# DNA polymorphisms and haplotype patterns of transcription factors involved in barley endosperm development are associated with key agronomic traits

**DOI:** 10.1186/1471-2229-10-5

**Published:** 2010-01-08

**Authors:** Grit Haseneyer, Silke Stracke, Hans-Peter Piepho, Sascha Sauer, Hartwig H Geiger, Andreas Graner

**Affiliations:** 1 Leibniz-Institute of Plant Genetics and Crop Plant Research (IPK), Corrensstr. 3, 06466 Gatersleben, Germany; 2 University of Hohenheim, Institute for Crop Production and Grassland Research (340), Bioinformatics, 70593 Stuttgart, Germany; 3 Max-Planck Institute for Molecular Genetics, Ihnestr. 73, D-14195 Berlin, Germany; 4 University of Hohenheim, Institute for Plant Breeding, Seed Science and Population Genetics (350), 70593 Stuttgart, Germany; 5 Plant Breeding, Centre of Life and Food Sciences Weihenstephan, Technische Universitaet Muenchen, Am Hochanger 4, 85350 Freising, Germany; 6 Department of Crop Sciences, Quality of Plant Products, University of Goettingen, Carl-Sprengel-Weg 1, 37075 Goettingen, Germany

## Abstract

**Background:**

Association mapping is receiving considerable attention in plant genetics for its potential to fine map quantitative trait loci (QTL), validate candidate genes, and identify alleles of interest. In the present study association mapping in barley (*Hordeum vulgare *L.) is investigated by associating DNA polymorphisms with variation in grain quality traits, plant height, and flowering time to gain further understanding of gene functions involved in the control of these traits. We focused on the four loci *BLZ1*, *BLZ2*, *BPBF *and *HvGAMYB *that play a role in the regulation of B-hordein expression, the major fraction of the barley storage protein. The association was tested in a collection of 224 spring barley accessions using a two-stage mixed model approach.

**Results:**

Within the sequenced fragments of four candidate genes we observed different levels of nucleotide diversity. The effect of selection on the candidate genes was tested by Tajima's D which revealed significant values for *BLZ1*, *BLZ2*, and *BPBF *in the subset of two-rowed barleys. Pair-wise LD estimates between the detected SNPs within each candidate gene revealed different intra-genic linkage patterns. On the basis of a more extensive examination of genomic regions surrounding the four candidate genes we found a sharp decrease of LD (*r*^2^<0.2 within 1 cM) in all but one flanking regions.

Significant marker-trait associations between SNP sites within *BLZ1 *and flowering time, *BPBF *and crude protein content and *BPBF *and starch content were detected. Most haplotypes occurred at frequencies <0.05 and therefore were rejected from the association analysis. Based on haplotype information, *BPBF *was associated to crude protein content and starch content, *BLZ2 *showed association to thousand-grain weight and *BLZ1 *was found to be associated with flowering time and plant height.

**Conclusions:**

Differences in nucleotide diversity and LD pattern within the candidate genes *BLZ1*, *BLZ2*, *BPBF*, and *HvGAMYB *reflect the impact of selection on the nucleotide sequence of the four candidate loci.

Despite significant associations, the analysed candidate genes only explained a minor part of the total genetic variation although they are known to be important factors influencing the expression of seed quality traits. Therefore, we assume that grain quality as well as plant height and flowering time are influenced by many factors each contributing a small part to the expression of the phenotype. A genome-wide association analysis could provide a more comprehensive picture of loci involved in the regulation of grain quality, thousand grain weight and the other agronomic traits that were analyzed in this study. However, despite available high-throughput genotyping arrays the marker density along the barely genome is still insufficient to cover all associations in a whole genome scan. Therefore, the candidate gene-based approach will further play an important role in barley association studies.

## Background

Association mapping is receiving considerable attention in plant genetics for its potential to fine map quantitative trait loci (QTL), validate candidate genes, and identify alleles of interest. Association mapping has several advantages over linkage mapping: First, a potentially larger number of alleles per locus can be surveyed simultaneously [1]. Second, results refer to a more representative genetic background. Third, the resolution of association mapping is increased because all recombination events accumulated in the population history are taken into consideration [2]. There are two ways to identify DNA-markers for QTL via association mapping: whole genome association mapping and re-sequencing of candidate genes. In whole genome association mapping populations are genotyped with a genome-wide set of closely linked and evenly distributed markers. This essentially requires a large number of markers and is therefore expensive and statistically complex [3]. The number of markers to be employed depends on the genome size and the extent of LD along the chromosomes. In a candidate gene-based approach, genotyping is targeted to functional and positional candidate genes for the trait under consideration [4]. This approach is assisted by (i) plant genomics resources such as expressed sequence tag (EST) databases, (ii) available knowledge on gene function in model organisms, and (iii) referenced information on physiology, biochemistry, and molecular genetics available for the trait of interest. In the present study we applied a candidate gene-based approach to find marker-trait associations for agronomic important traits in a spring barley collection.

The improvements of grain yield and quality, either for food or for feed, are paramount targets in any barley breeding program. It is known that transcription factors play an important role in controlling expression during seed development. Genetic differences in the synthesis of storage proteins can already be observed at the transcriptional level [5-7]. In barley, B-hordein represents the largest fraction of the storage protein. Functional analysis of the promoters of genes specifically expressed in the cereal endosperm, such as those encoding B-hordein (e.g. *Hor2*), has demonstrated the existence of *cis*-acting motifs capable of interacting with nuclear proteins that are putatively responsible for their tissue specificity and temporal regulation [8-10]. The endosperm box is a conserved *cis*-acting element, which contains two distinct protein binding sites: the prolamin-box (PB) and the GCN4-like motif (GLM). Four transcription factors (TFs) are the gibberellin-regulated Myb factor (*GAMYB*), the barley leucine zippers 1 and 2 (*BLZ1*, *BLZ2*), and the barley prolamin box binding factor (*BPBF*) that were shown to be involved in the transcription of B-hordeins encoded by the *Hor2 *locus.

**Figure 1:**
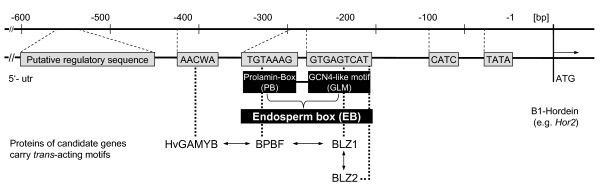
**Interplay between the candidate genes and the promoter region of a target gene (e.g. *Hor2*)**. Transcription start is displayed as ATG. Arrows to both sides show known interactions between the four transcription factors *BLZ1*, *BLZ2*, *BPBF *and *HvGAMYB*. Grey boxes indicate *cis*-regulatory motifs named as mentioned in the black boxes.

*BLZ1 *mRNA is detected during early endosperm development. The single copy gene is a transcriptional activator that interacts with endosperm-specific gene promoters (Figure 1). Vicente-Carbajosa et al. [11] demonstrated the involvement of BLZ1 in the regulation of hordein gene expression through binding to the GLM. BLZ1 protein functions as a transcriptional activator and is able to form either homodimers or heterodimers with BLZ2 [12]. The *BLZ2 *mRNA expression is restricted to the endosperm and its protein specifically binds to the GLM [12]. As indicated by its designation, the BPBF has been shown to activate hordein genes through binding to the PB [13,14]. Transient expression experiments in developing barley endosperms demonstrate that BPBF *trans*-activates transcription from the PB element of a native *Hor2 *promoter [14]. Positive regulatory interaction was observed between BPBF and HvGAMYB in the control of endosperm gene expression during seed development [13]. In developing seeds abundant expression of the transcription factor HvGAMYB is induced by gibberellic acid. Its mRNA can be detected in the starchy endosperm and other grain tissues [13]. The protein *trans*-actives transcription from the native *Hor2 *promoter through binding to a third motif (5'-AACA/TA-3') that is present in endosperm-specific genes. Thus, HvGAMYB represents a key regulator of genes specifically expressed in the endosperm during seed development [13]. In addition to seed tissue, HvGAMYB also plays a role in other aspects of plant growth and development [15] and *BLZ1 *expression was also detected in leaves and roots [11].

A phenotypically well characterized spring barley collection was recently established by Haseneyer et al. [16] as resource for this association study. Information about morphological properties of the accessions is available and population structure was determined with 45 EST-derived SSR markers. In the current paper we report on the analysis of nucleotide diversity parameters for the above mentioned candidate genes. Results are presented on the association between sequence polymorphisms within *BLZ1 *(chromosome 5 H), *BLZ2 *(chromosome 1 H), *BPBF *(chromosome 5 H), and *HvGAMYB *(chromosome 3 H) and the phenotypic variation of the five agronomic traits thousand-grain weight, starch content, protein content, plant height and flowering time.

## Methods

### Plant material and phenotypic analyses

The above mentioned collection of spring barleys selected from the Barley Core Collection (BCC) and the Federal *ex situ *Genebank (HOR) was used in this study (Additional file 1). The germplasm set consists of 128 two-rowed and 96 six-rowed accessions originating from Europe (N = 109), East Asia (N = 40), America (N = 30), and West Asia and North Africa (N = 45). Eighteen accessions were classified as "breeding/research material", 55 accessions as landraces/traditional cultivars while the remaining accessions represent advanced breeding lines and cultivars. Accessions were phenotypically evaluated at Stuttgart-Hohenheim (South Germany), Irlbach (South Germany) and Bergen-Wohlde (North Germany) in 2004 and 2005. Each trial was arranged in microplots in a 25 × 15 lattice design with three replicates. Thousand-grain weight (TGW), flowering time (FT), and plant height (PH) were recorded. Grain quality (crude protein content (CPC) and starch content (STR)) was assessed by near infrared reflectance spectroscopy (NIRS, for further details see [16]).

### Population structure

All 224 accessions were genotyped with 45 simple sequence repeat (SSR) markers that are evenly distributed across the barley genome [17]. A population structure with *K *= 2 subgroups was inferred from the SSR data by using the STRUCTURE 2.0 software package [18,19]. The individual steps of analysis were described in detail by Haseneyer et al. [16].

### Genotyping and genetic mapping

Eight seeds from each accession were grown in the greenhouse and leaves from 2-week-old seedlings were harvested and bulked for genomic DNA extraction using the method described in Stein et al. [20]. PCR-primers were designed using the software Primer3 [21]. Primer sequences and the fragment-specific PCR profile conditions are given in additional file 2. PCR for single nucleotide polymorphism (SNP) analysis by DNA sequencing was performed as described in full detail by [22]. In preparation for DNA sequencing, we purified the PCR amplicons in 384-well plates and adjusted to similar molarity. 10 ng PCR product was used as template for cycle sequencing. DNA sequences were determined using ABI BigDye Terminator 3.1 chemistry and 96-capillary sequencer systems (ABI 3730 × l). Forward and reverse PCR primers were used as sequencing primers (Additional file 2). DNA sequence ladders were processed for quality scoring using a software package based on the poly-phred system [23]. We applied the program Sequencher™ Version 4.5 (Gene Codes Cooperation) for sequence alignment and editing. All positions given in the text correspond to the positions in the haplotype sequence alignments related to the start codon (Additional file 3).

*BLZ1, BLZ2 *and *BPBF *were genetically mapped in the Oregon Wolfe Barley (OWB) mapping population developed by Costa et al. [24]. Positions were determined on an updated OWB map [25]. Therefore, we designed cleaved amplified polymorphic sequence (CAPS) markers that require the use of the restriction enzymes Nci I (*BLZ1*, SNP 1733), Ssp I (*BLZ2*, SNP 2161), and Sty I (*BPBF*, SNP -210). *HvGAMYB *was mapped earlier by Haseneyer et al. [26].

### Diversity and association analysis

The candidate genes' DNA fragments were sequenced for each accession of the collection. DnaSP Version 4.10 [27] was applied for the statistical sequence analysis. This software does not take into account the alignment gaps that may lead to underestimated diversity values. To avoid potential bias, insertion-deletion events (indels) were treated as single sites. Nucleotide diversity estimated as Pi (π) [28], haplotype diversity (Hd), and Tajima's D [29] were computed. Diversity values of gene fragments showing no sequence overlap were calculated fragment-wise and then the arithmetic average was computed.

LD between pairs of polymorphic sites (minor allele frequency, MAF ≥ 0.05) was estimated by TASSEL software, version 1.9.3 [30]. LD is expressed by *r*^2 ^[31] and the statistical significance (*P*-value) of the observed LD is estimated by Monte-Carlo approximation of Fisher's exact test [32], with 1,000 permutations. In order to estimate the local decay of LD, additional markers flanking the candidate genes at increasing distances were investigated in the entire collection. The expected value of *r*^2 ^is E(*r*^2^) = 1/(1+*C*), where *C *= 4 *Nc*, *N *is the effective population size, and *c *is the recombination fraction between sites [33]. This model was employed in nonlinear regression of *r*^2 ^on *c*, treating *N *as a parameter to be estimated, using PROC NLIN of the SAS System for Windows (Version 9.1.3.)

Combined analyses of phenotypic and genotypic data were performed using Version 9.1.3 of the SAS System for Windows. We followed a two-stage mixed model approach [34,35] where in the first stage adjusted entry means and weights were computed for each trial, which were then subjected to a mixed model analysis combined over trials in the second stage. Our analysis is based on the assumption that genotypes are a random sample from the world collection of barley genotypes. In order to compute adjusted means for single trials, however, we formally took genotypes as fixed in the first stage, fitting a linear model with fixed effects for genotypes and replicate and random effects for block and error. Thus, adjusted means were unbiased estimates of the genotypes' performances in the different environments, which allowed formulating a mixed model for adjusted means in the second stage. Note that taking genotypes random, and hence computing best linear unbiased predictors (BLUPs) of genotype performances, in the first stage would have caused biases that would have been difficult to account for in stage two [35]. In the second stage, the following model terms were fitted: overall mean (fixed), trial main effects (fixed), genotype main effect (random), genotype-by-trial interaction (random). In addition, spike morphology and geographic origin were modelled by fixed effects for 'row number', which had two levels, and 'origin', which had four levels. Population structure was modelled by fixed-effects regression on a Q matrix of membership probabilities of *N *genotypes in each of *K *subgroups. The Q matrix was computed using the Bayesian approach of Pritchard et al. [19]. Associations of haplotypes and SNP markers were tested by adding a haplotype or SNP marker covariate to the fixed part of the model. Tests of fixed effects were based on variance estimates using the restricted maximum likelihood (REML) method and denominator degrees of freedom approximated by the method of Kenward and Roger [36]. The genetic variance explained by a fixed effect was computed by the relative reduction in genetic variance when the fixed term was added. Weights to model the error variance of adjusted means in stage two were computed based on the diagonal elements of the inverse of the asymptotic variance-covariance matrix of adjusted means [35]. All variance components were estimated by the REML method. Adjusted means were compared by Wald *t*-tests [37]. As the haplotype means were not variance balanced, we used the method of Piepho [38] to generate a letter display showing the significance of comparisons. Type I error rate was controlled by the Bonferroni-Holm procedure [39].

## Results

### Sequence diversity and haplotype analysis

The polymorphism density ranged from 1 polymorphism/31 bp (*BLZ2*), 1 polymorphism/42 bp (*BPBF*), 1 polymorphism/55 bp (*BLZ1*) to 1 polymorphism/74 bp (*HvGAMYB*). Nucleotide diversities (π) were determined for *BLZ1 *(1,113 bp), *BLZ2 *(2,232 bp), *BPBF *(1,119 bp) and *HvGAMYB *(3,337 bp) for the entire germplasm set and the geographical and morphological subsets individually (Table 1). Diversity estimates for the entire collection ranged from π = 2.4 × 10^-3 ^(*HvGAMYB*) to π = 8.1 × 10^-3 ^(*BPBF*). In most cases individual subgroups showed a similar range of nucleotide diversities for all candidate genes. An exception was only noted for *BLZ2 *where the two-rowed subset displayed a highly reduced π-value, whereas a high diversity was observed for the East Asian accessions.

**Table 1:** Estimates of nucleotide and haplotype diversity for the candidate genes *BLZ1, BLZ2, BPBF *and *HvGAMYB*

Accession (sub)set^1^	No. of polymorphism	Nucleotide diversity (π × 10^-3^)	No. of haplotypes	Haplotype diversity (Hd)
	*BLZ1*			
Total	20	5.6	8	0.65
AM	20	5.8	6	0.68
EA	19	6.0	7	0.77
EU	20	5.2	8	0.66
WANA	20	5.2	8	0.73
2-rowed	19	5.4	7	0.63
6-rowed	20	5.8	8	0.70
	*BLZ2*			
Total	72	6.5	18	0.68
AM	27	6.0	4	0.65
EA	64	8.0	9	0.87
EU	67	4.0	9	0.38
WANA	56	6.3	9	0.80
2-rowed	58	1.8	9	0.23
6-rowed	68	5.9	16	0.86
	*BPBF*			
Total	26	8.1	21	0.68
AM	22	9.1	4	0.60
EA	25	7.6	11	0.73
EU	23	7.7	14	0.62
WANA	21	8.9	8	0.77
2-rowed	23	7.4	14	0.56
6-rowed	25	8.7	14	0.77
	*HvGAMYB*			
Total	45	2.4	18	0.74
AM	34	2.8	8	0.80
EA	30	1.8	8	0.64
EU	31	2.1	9	0.68
WANA	37	2.7	11	0.84
2-rowed	29	1.9	10	0.61
6-rowed	43	2.7	15	0.84

1: Diversity estimates for different geographic regions (AM: America, EA: East Asia, EU: Europe, WANA: West Asia and North Africa) and in two-rowed and six-rowed barleys are given

Haplotype analysis indicated a similar diversity at most gene loci and for all subpopulations, although the number of haplotypes per locus ranged from 8 (*BLZ1*) to 21 (*BPBF*). The haplotype diversity at *BLZ2*, *BPBF *and *HvGAMYB *was mainly caused by the six-rowed accessions that were particularly frequent in the American, East Asian and West Asian and North African subsets. The two-rowed subset, that primarily included European genotypes, revealed the lowest estimates for all loci considered, especially for the *BLZ2 *gene.

### Linkage disequilibrium

**Figure 2:**
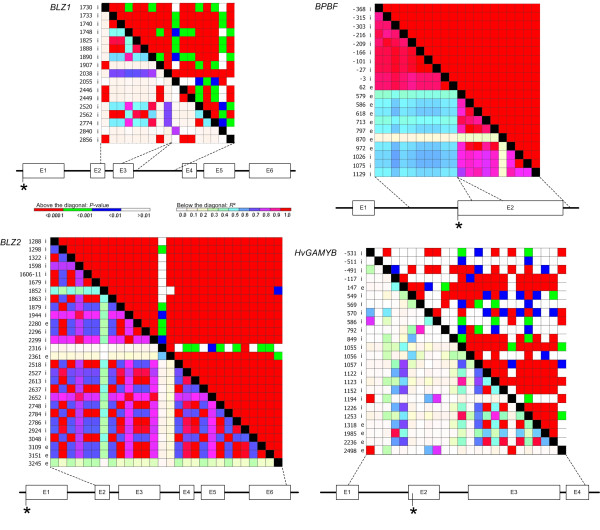
**Linkage disequilibrium between the polymorphic sites (MAF>0.05) within the candidate loci *BLZ1, BLZ2, BPBF, HvGAMYB***. Asterisk indicates transcription start, dashed lines indicate regions that were sequenced and "i" and "e" column indicates polymorphisms in introns and exons, respectively. MAF = minor allele frequency.

The pairwise LD values revealed different patterns for the genes studied (Figure 2). *BLZ1 *and *HvGAMYB *showed strong LD (*r*^2^>0.8, *P *< 0.0001) only between a few polymorphic sites. At the *BLZ1 *locus two blocks of polymorphism (positions 1740 to 1890 and 2520 to 2774) displayed significant LD estimates higher than *r*^2 ^= 0.5 (*P *< 0.0001). *BLZ2 *and *BPBF *showed significant LD across the entire sequence. Even beyond the gap of 482 bp between the two sequenced fragments of *BPBF *(positions -368 to 62 and 579 to 1129) LD persisted at a high level (*r*^2^>0.4, *P *< 0.0001). The sites 2316 and 2361 at the *BLZ2 *gene and 870 at the *BPBF *locus segregated separately from the remaining polymorphic sites.

**Figure 3:**
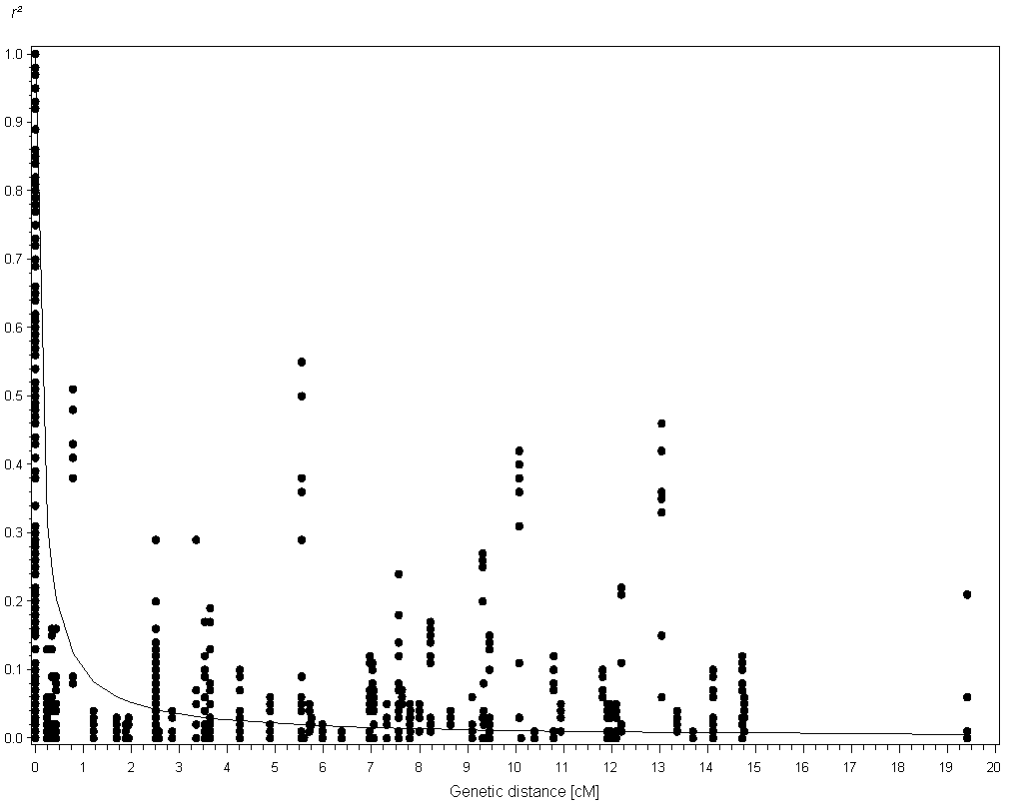
**LD decay plot in the surrounding regions of the four candidate genes as a function of genetic distance (in cM)**. Dots indicate pairwise comparisons between SNP alleles with minor allele frequency larger 0.05. The curve shows nonlinear regression of *r*^2 ^on genetic distance.

The results of the extended LD study of markers flanking the four candidate genes showed that LD remained significant at distances up to 19 cM. However, individual *r*^2 ^values sharply decreased to *r*^2^<0.1 within 1 cM in the surrounding regions of all four candidate genes (Figure 3). Only in the proximal region of *BLZ2 *sustained levels of LD were observed up to 10 cM (Additional file 4).

The impact of selection on the four candidate genes was tested by calculating Tajima's D. Significant deviations from the mutation-drift equilibrium were observed for *BLZ1 *and *BPBF *for the entire collection (Table 2). Within the two-rowed subset *BLZ1*, *BLZ2*, and *BPBF *were significant, while in the six-rowed subset only *BPBF *revealed a significant Tajima D-value. No significant values were observed for *HvGAMYB*.

**Table 2:** Tajima's D for the candidate genes *BLZ1, BLZ2, BPBF*, and *HvGAMYB*

Candidate gene	Total	2-rowed subset	6-rowed subset
*BLZ1*	2.22*	2.12*	ns
*BLZ2*	ns	-2.03*	ns
*BPBF*	2.86**	2.57*	2.74**
*HvGAMYB*	ns	ns	ns

**: significant at *P *< 0.01, *: significant at *P *< 0.05, ns: not significant

### Marker-trait association

For all association analyses the model including population structure (two subgroups referred to as '*K2*'), 'row number' and 'origin' was applied. Several SNPs within the candidate gene *BLZ1 *being in high LD with one another were significantly associated with flowering time (Table 3, Additional file 5). They explained between 6.5 to 7.5% of the genetic variation and phenotypic means of the respective SNP alleles revealed a significant two-day difference in flowering time.

**Table 3:** Percentage explained variance (%Var), phenotypic means of SNP alleles, and significant (*P *< 0.05) marker-trait associations

Candidate gene	Site position^1^	%Var	*P *-value	Means of SNP alleles^2^ (accession classes)	
*BLZ1*	Flowering time [days after sowing]				
	1733	6.46	0.0033	A: 67.96	C: 69.87
	1825	7.38	0.0031	G: 67.96	A: 69.91
	1888	6.46	0.0033	C: 67.96	T: 69.87
	1890	7.10	0.0017	G: 67.50	A: 69.54
	2038	7.52	0.0011	Del: 67.62	AT: 69.68
	2520	7.10	0.0017	G: 67.50	A: 69.54
	2562	6.46	0.0033	C: 67.96	T: 69.87
	2774	7.10	0.0017	T: 67.50	C: 69.54
*BPBF*	Crude protein content [%]				
	-368	6.65	0.0003	G: 14.85	A: 15.63
	-315	5.40	0.0003	T: 14.85	C: 15.63
	-303	6.50	0.0003	T: 14.87	A: 15.63
	-215	6.86	0.0002	C: 14.86	G: 15.63
	-209	6.19	0.0004	A: 14.88	G: 15.63
	-166	6.07	0.0003	A: 14.87	G: 15.64
	-101	5.38	0.0004	T: 14.88	C: 15.64
	-27	4.94	0.0003	C: 14.88	T: 15.65
	-3	6.76	0.0002	T: 14.82	C: 15.64
	62	4.02	<.0001	T: 14.79	C: 15.66
	579	12.40	0.0003	T: 14.90	C: 15.66
	586	3.34	0.0025	G: 14.93	A: 15.58
	618	3.42	0.0019	A: 14.91	G: 15.58
	713	5.45	0.0008	A: 14.88	G: 15.60
	797	4.06	0.0029	G: 14.94	T: 15.58
	972	4.40	0.0016	G: 14.91	A: 15.59
	1026	7.91	0.0019	T: 14.98	G: 15.62
	1075	3.51	0.0007	A: 14.91	G: 15.63
	1129	5.57	0.0007	C: 14.92	A: 15.63
	Starch content [%]				
	-368	4.44	0.0004	A: 55.58	G: 56.74
	-315	7.30	0.0007	C: 55.57	T: 56.66
	-303	5.39	0.0002	A: 55.56	T: 56.77
	-215	4.41	0.0004	G: 55.58	C: 56.73
	-209	4.84	0.0003	G: 55.57	A: 56.74
	-166	5.12	0.0001	G: 55.55	A: 56.78
	-101	4.79	0.0001	C: 55.53	T: 56.78
	-27	4.22	0.0001	T: 55.55	C: 56.79
	-3	3.27	0.0008	C: 55.61	T: 56.72
	62	0.16	0.0009	C: 55.56	T. 56.70

1: Positions refer to the sequence alignment given in additional file 3

2: All differences between classes for a given site position are significant at *P *= 0.05

Within the *BPBF *gene polymorphisms in the exonic and 5'- and 3'-untranslated regions were associated with crude protein content. Phenotypic means of the SNP alleles revealed a significant difference of 5.9% in crude protein content. One SNP (pos. 579) explained 12.4% of the genetic variation. Estimates for pairwise LD were significant for these sites with *r*^2^>0.5 (*P *< 0.0001). A portion (5'-untranslated region, and SNP at pos. 62) of these sites showed association to starch content revealing a significant difference between phenotypic means of the contrasting alleles.

### Haplotype-trait association

In accordance with the handling of SNP data, haplotype-trait associations were restricted to haplotype classes which were more frequent than 0.05. Applying this frequency threshold, three haplotype classes were detected for *BLZ1*, *BLZ2*, and *BPBF *and four haplotype classes for *HvGAMYB *(Additional file 5) that were entered in the association analysis. *BLZ1 *was significantly (*P*-value < 0.05) associated with flowering time and plant height, and explained 3.3% and 3.1% of the genetic variation, respectively (Table 4). A weak association of *BLZ1 *with crude protein content was observed explaining 2.7% of the genetic variation. *BLZ2 *haplotypes were associated with thousand-grain weight and explained 4.0% of the genetic variance (Table 4). Haplotypes of the candidate gene *BPBF *were significantly associated to crude protein content and starch content and explained 8.2% and 6.0% of the genetic variation, respectively.

**Table 4:** Haplotype-trait associations (*P *= 0.05) and percentage explained genetic variance (%Var) of the candidate genes' haplotypes

Candidate gene^1^	Crude protein content		Starch content		Thousand-grain weight		Flowering time		Plant height	
					
	%Var	Significance^2^	%Var	Significance	%Var	Significance	%Var	Significance	%Var	Significance
*BLZ1*	2.65	0.050	-	-	-	-	3.28	0.031	3.05	0.036
*BLZ2*	-	-	-	-	4.01	0.027	-	-	-	-
*BPBF*	8.25	0.001	6.02	0.006	-	-	-	-	-	-

1 Only haplotypes with a frequency greater than 0.05 are considered, no association was detected between haplotypes of *HvGAMYB *and any of the five traits

2 Significance level of Wald-*t *test

## Discussion

In this study, a worldwide collection of spring barley accessions was used to perform marker-trait association analyses based on a set of four candidate genes for grain quality.

Different patterns of sequence diversity, haplotype diversity and LD were observed for the candidate genes *BLZ1*, *BLZ2*, *BPBF*, and *HvGAMYB*. A similar variability of LD patterns was found for different members of the CBF (C-repeat binding factor) transcription factor family [40]. In the present study LD within genes was weak for *BLZ1 *and *HvGAMYB *but strong for the other two genes. The high number of sequence polymorphisms detected at the *BLZ2 *locus is in accordance with observations on the homologous gene *Opaque 2 *in maize [41]. Compared to the remaining members of the *bzip *class of regulatory genes, *BLZ2 *and its homologues seem to be characterized by exceptionally high levels of polymorphism. The high SNP frequency in *BLZ2 *is not reflected in a high nucleotide or haplotype diversity since diversity in this gene is caused by only few frequent and many rare SNPs. *HvGAMYB *showed the lowest and *BPBF *the highest values of nucleotide diversity, whereas the opposite was found for the haplotype diversity. This pattern is due to the high pairwise LD at the *BPBF *locus resulting in few frequent and many rare haplotypes. The low level of LD, which was observed at the *HvGAMYB *locus, might be due to a low selection pressure on this gene during its domestication and breeding history [26].

Malting barley is characterized by a low protein and high starch content [42]. In this regard, two-rowed barley is preferred by European brewing industry due to the favourable protein to starch relation. A strong selection for these two negatively correlated traits might have had a bearing on nucleotide diversity in the underlying candidate genes. This is apparent for the *BLZ2 *locus where the reduced diversity in the European subset corresponds with a high proportion of two-rowed genotypes in this geographic subset. The observed reduction in sequence variation might be a consequence of purifying selection [43]. The negative Tajima D value might indicate such kind of selection for *BLZ2 *in the two-rowed subset caused by the elimination of deleterious alleles and leaving only one major haplotype which is common to 95 of the 108 two-rowed accessions.

It is well known that selection in autogamous organisms leads to an increase in LD [44]. In this context, selection may affect the regulatory regions of genes, or target regulatory loci rather than the protein-coding region of genes [45]. In *Zea mays *L. the ear underwent dramatic morphological alteration upon domestication and has been a continuing target of selection for grain yield [46]. Therefore, Hufford et al. [46] hypothesize that genes targeted by selection are more likely to be expressed in tissues that experienced high levels of morphological divergence during crop improvement. One such tissue in barley is the endosperm since its characteristics are the determinants of malting quality [47]. Since expression of *BLZ2 *and *BPBF *is restricted to the endosperm [12,14] the selection and corresponding enrichment of only a few favourable alleles at these loci entails an increase in LD. Determining the nucleotide diversity of these two genes in wild barley would allow verification of this hypothesis.

The tentative appraisal about the impact of selection on the four candidate genes was investigated by calculating Tajima's D. A significant deviation from the mutation-drift-equilibrium, especially in the two-rowed subgroup, was observed for the three candidate genes that were found to be associated to the target traits. In Europe, two-rowed barley is the main target for the improvement of seed quality parameters. This is in accordance with the significant Tajima D values obtained for the three loci in this subgroup indicating footprints of selection on *BLZ1*, *BLZ2 *and *BPBF*. However, selection might act in different ways: In case of *BLZ2 *selection resulted in the accumulation of a large number of low frequency SNP alleles as 61% of the recorded SNPs have a MAF < 5%. In conjunction with the extended LD across this gene, this results in the presence of only one major haplotype for this gene which is present in 54% of the accessions. Within the subset of two-rowed barleys, this haplotype is even more dominant showing a frequency of 88% (see previous pragraph). In case of *BLZ1 *and *BPBF*, 11% and 23% of the SNPs show a MAF < 5%. Hence, selection was effective in the elimination of rare SNP alleles and the accumulation of moderate frequent SNP alleles was promoted. The indication that these two genes are targeted by balancing selection is supported by significant Tajima D values.

The detected marker-trait associations, even for polymorphisms explaining only a minor portion of the trait variation, are attributed to the high statistical power achieved by (i) extensive and precise phenotyping of the target traits as reflected by high heritability estimates [16], (ii) considering the population structure of the collection and (iii) the high phenotypic variability of the worldwide collection and the large nucleotide diversity within the selected candidate genes. However, the power to detect an association also depends on the number of accessions in the individual haplotype classes on which the analysis is based. In the analysed collection the high degree of diversity resulted in prevalence of rare haplotypes that occurred in less than 5% of accessions and thus were excluded from the analysis to avoid spurious associations. Interestingly, most of the phenotypic differences were found between those rare haplotype classes. Hence, a considerably larger collection size or the selective enrichment of haplotype classes would be needed to warrant a proper sample size for rare haplotypes as well.

The observed haplotype associations of *BLZ1 *with flowering time and plant height corroborate the hypothesis of Vicente-Carbajosa et al. [11] that this gene is involved in developmental processes and photoperiodic response. Pleiotropic effects of a single gene as observed for *BLZ1 *lead to overlapping QTL position estimates for different traits providing a basis for enhancing the effectiveness of marker-assisted selection [48]. Thus, candidate gene-based association studies for two or more traits might substantially contribute to cultivar improvement. However, in the present study, we could not identify an advantageous haplotype or SNP sites in the investigated candidate genes comparable to the ones found in the *sh4*-d gene in rice, the *Q-gene *in wheat and the *ppd-H1 *gene in barley [49-51]. As the present candidate genes were described as *trans*-active regulators for hordein encoding genes [11,12,14,52], we hypothesize that they influence both grain protein composition and protein content and thus are of importance not only for malting [53] but also for nutritional quality [54].

Both, marker-trait and haplotype-trait associations yielded comparable results. In both approaches significant associations of *BLZ1 *with flowering time and *BPBF *with crude protein and starch content were found. Using haplotypes instead of SNP alleles revealed a higher number of associations. This shows the higher sensitivity and statistical power of haplotype-trait associations [55,56] as here accessions are divided in several classes whereas in marker-trait association only two classes, representing the two SNP alleles, are considered. The portion of explained genetic variance by SNP sites was in reasonable agreement with the explained genetic variance by haplotypes. As would be expected for a quantitative trait, only a small part of the entire genetic variation could be explained by the variation occurring at the candidate loci. It follows that the remaining variation is due to additional loci that also influence the expression of crude protein content, starch content, thousand-grain weight, plant height, and flowering time.

With the increasing availability of high-throughput genotyping platforms for barley (DArT array [57], oligonucleotide pool assay [58]), estimation of genome-wide LD decay and whole genome association studies become a feasible alternative to the analysis of candidate genes. LD studies based on such genotyping data that were retrieved for a collection of genotypes resulted in a decay of intrachromosomal LD below *r*^2^<0.2 within 2.6 cM [59], *r*^2^<0.15 within 3.2 cM [60] and *r*^2^<0.5 within 3.9 cM [58], respectively. Complementary to the decrease in genetic diversity, LD has been shown to increase from wild barley via landraces to modern cultivars [58,61]. Notwithstanding this observation, LD within cultivated barley is also population dependent so that comparison of genome-wide LD between collections composed of accessions with different origins is difficult. In our world-wide collection the extent of genome-wide LD decreases more rapidly than in geographically restricted collections of domesticated barley germplasm [58-60]. The chromosomal regions surrounding the four candidate genes display a rapid LD decay. However, genome wide DNA fingerprinting of the present population would significantly increase the knowledge about LD structure in the present collection and facilitate comparisons to other mapping panels regarding local LD patterns and trait associations.

## Conclusions

Nucleotide diversity and LD patterns of *BLZ1*, *BLZ2*, *BPBF*, and *HvGAMYB *revealed differences between the candidate genes and between geographical and morphological subsets of the collection. This reflects the impact of selection on the nucleotide sequence of these four candidate loci.

According to literature, the four candidate genes represent transcriptional key regulators in barley. However, only three of the four selected candidate genes could be confirmed by haplotype-trait association studies. We conclude that there is still an incomplete knowledge about the expression and interaction of genes controlling the quantitative traits crude protein content, starch content, thousand-grain weight, plant height, and flowering time in barley. Additionally, both haplotypes and SNPs only explained a part of the genetic variation. Therefore, and in accordance with their quantitative inheritance, we assume that the investigated seed traits, plant height, and flowering time are influenced by many additional hitherto unknown factors each contributing a small part to the expression of the phenotype.

Although genome-wide association mapping could provide a more comprehensive picture of loci involved in the regulation of crude protein content, starch content, thousand-grain weight, flowering time, and plant height there is a risk of overlooking an association in genome-wide association studies. As has been demonstrated in the present study, a gene may contain SNPs that are associated and others that are not associated with the trait under consideration. If only one or two SNPs per locus (e.g. EST) would be interrogated as is presently the case with many SNP marker arrays used for whole genome scans, it is possible that the "right" SNP was not included in the array. On the other hand, a candidate gene-based approach might suffer from the limited knowledge about candidates for a given trait and hence only a part of the genetic variation for this trait is captured. Further verification of the observed associations is difficult owing to the quantitative nature of the target trait. Moreover, LD decay and hence genetic resolution of the present population is still insufficient to preclude that the observed association is not due to the presence of a physically linked gene being in LD with the candidate gene. Notwithstanding this fact, future candidate gene-based approaches will greatly benefit from the continuous accumulation of knowledge on gene function and regulation. Because of this and due to the still insufficient marker coverage of the barley genome, the candidate gene-based association mapping will continue to play an important role in barley.

## Authors' contributions

GH carried out the molecular genetic studies, the sequence alignment and analyses, the statistical association analyses, and drafted the manuscript. SSt participated in the design and coordination of the study. HPP developed the concept for the statistical analysis. SSa carried out sequencing of the candidate genes. HHG and AG participated in the design and coordination of the study, interpretation of the data and the development of the manuscript. All authors read and approved the final manuscript.

## Supplementary Material

Additional file 1 **Accessions under study**. Information about origin, row number, biological status and haplotypes observed for the candidate genes *BLZ1*, *BLZ2*, *BPBF *and *HvGAMYB *are given. Click here for file

Additional file 2 **Primer sequences for PCR and sequencing of the candidate genes, PCR conditions and fragment range**. 1: numbers indicate positions in the nucleotide sequence alignment of the candidate genes' haplotypes given in additional file 3. Click here for file

Additional file 3 **Nucleotide sequence alignments of the candidate gene fragments**. Description: Haplotype sequences of *BLZ1 *(reference = [GenBank:X80068.1]), *BLZ2 *(reference = [GenBank:Y10834.1]), *BPBF *(reference = [GenBank:AJ000991.1]) and *HvGAMYB *(reference = [GenBank:AY008692.1]). The alignment position is relative to the ATG and gaps are counted. Abbreviations: hpt = haplotype, cds = coding sequence, gene = genomic sequence (if available). Click here for file

Additional file 4 **Linkage disequilibrium in the surrounding region of the candidate genes *BLZ1 *(A), *BLZ2 *(B), *BPBF *(C), and *HvGAMYB *(D)**. The position 0.0 cM refers to the candidate gene. The symbols × and ◆ indicate significant (*P *= 0.05) and non significant pairwise comparisons, respectively. Click here for file

Additional file 5 **Haplotype sequence and marker-trait associations detected in the four candidate genes *BLZ1*, *BLZ2*, *BPBF*, *HvGAMYB***. Significant associations (*P *= 0.05) are indicated by 'x'. The traits crude protein content (CPC), starch content (STR), thousand-grain weight (TGW), plant height (PH), and flowering time (FT) were considered. Haplotype frequencies (in %) and minor allele frequencies (MAF, in %) are given. Click here for file
