# Supplementary material for: DNA polymorphisms and haplotype patterns of transcription factors involved in barley endosperm development are associated with key agronomic traits
Source: BMC Plant Biol. 2010 Jan 8;10:5. doi: 10.1186/1471-2229-10-5 (PMC2822787; doi:10.1186/1471-2229-10-5)
Supplement: Additional file 2 — Primer sequences for PCR and sequencing of the candidate genes, PCR conditions and fragment range. 1: numbers indicate positions in the nucleotide sequence alignment of the candidate genes' haplotypes given in additional file 3. [file 1471-2229-10-5-S2.PDF]

Table S2. Primer sequences for PCR and sequencing of the candidate genes, PCR conditions and fragment range are given

Candidate gene	Frag-ment	Primer sequences		PCR conditions		Range in alignment[1] [bp]
		forward	revers	Annaeling temperature [°C]	Elongation time [min:sec]	
BLZ1	B	5'- CCAGAACGTTTTGCAGCTAGTC -3'	5'- ATCAGTAGGGACCCCATTCC -3'	62	01:30	1587...2299
	C	5'- TGTGGTTCCACTTCAAAACAAG -3'	5'- TTCAGGTGAGCTGCCTTTCT -3'	60	01:30	2122...3055
BLZ2	2	5'- GGAATTTGCCGACAAAGCTC -3'	5'- TGTGCCTCCCCTTCCATATC -3'	59	01:00	1081...1502
	3	5'- CCTCATGGGAGCCATCACC -3'	5'- CTTCTAGGGTTTCAACATTTGCC -3'	59	01:00	1454...2437
	4	5'- CGCTCAAGGAGTAGAAAAGC -3'	5'- CTATTTCATTTGAATCCAATGTAGT -3'	59	01:00	2176...3407
BPBF	A	5'- CTACATACTACCCTTCGTTCACCTG -3'	5'- GAACTTGGTGTTACCAGACTTGC -3'	58	00:45	>-431...144
	B	5'- GTCCAAACAATGGCTACTACTATGG -3'	5'- ACACTCTCTCGAGTTTCACTCAAAT -3'	60	01:00	542...1178
HvGAMYB	1	5'- TGAAAATTCTCCGCATACTGG -3'	5'- GATGAGCCTCTCCTCCTCGG -3'	60	01:30	>-691...327
	2	5'- GAACGCGGTGCAGAAGAA -3'	5'- CTAGCAGGATATATTGGCAAACCG -3'	60	01:30	201...1389
	3	5'- AGTTGCCAGGGCGTACTGA -3'	5'- GGATCCAGTGAGTGAATTGCC -3'	60	01:30	1296...2248
	4	5'- AACCAAGCAGGGATGCTAAA -3'	5'- GTGAGGGATACCAAGAATCTCC -3'	58	02:00	1628...2715

1: numbers indicate positions in the haplotype alignments of the candidate genes given in the supplemental figure S1
