# Supplementary material for: DNA polymorphisms and haplotype patterns of transcription factors involved in barley endosperm development are associated with key agronomic traits
Source: BMC Plant Biol. 2010 Jan 8;10:5. doi: 10.1186/1471-2229-10-5 (PMC2822787; doi:10.1186/1471-2229-10-5)
Supplement: Additional file 3 — Nucleotide sequence alignments of the candidate gene fragments. Description: Haplotype sequences of BLZ1 (reference = [GenBank:X80068.1]), BLZ2 (reference = [GenBank:Y10834.1]), BPBF (reference = [GenBank:AJ000991.1]) and HvGAMYB (reference = [GenBank:AY008692.1]). The alignment position is relative to the ATG and gaps are counted. Abbreviations: hpt = haplotype, cds = coding sequence, gene = genomic sequence (if available). [file 1471-2229-10-5-S3.PDF]

***BLZ1***

```
                 10        20        30        40        50        60        70        80        90       100       110
         ....|....|....|....|....|....|....|....|....|....|....|....|....|....|....|....|....|....|....|....|....|....|
BLZ1 gene atggagcgcgtcttctccgtcgaggagatccccgacccgttctggggccagccgtcgccgcggcagcgcgggcgccggccgccggagggcgccatgaaccggtgcccgtc
BLZ1 cds  atggagcgcgtcttctccgtcgaggagatccccgacccgttctggggccagccgtcgccgcggcagcgcgggcgccggccgccggagggcgccatgaaccggtgcccgtc
hpt01     --------------------------------------------------------------------------------------------------------------
hpt02     --------------------------------------------------------------------------------------------------------------
hpt03     --------------------------------------------------------------------------------------------------------------
hpt04     --------------------------------------------------------------------------------------------------------------
hpt05     --------------------------------------------------------------------------------------------------------------
hpt06     --------------------------------------------------------------------------------------------------------------
hpt07     --------------------------------------------------------------------------------------------------------------
hpt08     --------------------------------------------------------------------------------------------------------------

                120       130       140       150       160       170       180       190       200       210       220
         ....|....|....|....|....|....|....|....|....|....|....|....|....|....|....|....|....|....|....|....|....|....|
BLZ1 gene cgagtggtacttccagaagttcctcgaggaggccgtgctcgacagccccgccgccgaccctagcccgatgtccggtgctagcggacgaggtcaagcggcctgccgtccgc
BLZ1 cds  cgagtggtacttccagaagttcctcgaggaggccgtgctcgacagccccgccgccgaccctagcccgatgtccggtgctagcggacgaggtcaagcggcctgccgtccgc
hpt01     --------------------------------------------------------------------------------------------------------------
hpt02     --------------------------------------------------------------------------------------------------------------
hpt03     --------------------------------------------------------------------------------------------------------------
hpt04     --------------------------------------------------------------------------------------------------------------
hpt05     --------------------------------------------------------------------------------------------------------------
hpt06     --------------------------------------------------------------------------------------------------------------
hpt07     --------------------------------------------------------------------------------------------------------------
hpt08     --------------------------------------------------------------------------------------------------------------

                230       240       250       260       270       280       290       300       310       320       330
         ....|....|....|....|....|....|....|....|....|....|....|....|....|....|....|....|....|....|....|....|....|....|
BLZ1 gene gtggcgtggcggggacggcgacgggcccggcggtggacccggtggagtacaacgccatgctcaagcagaagctcgagaaggacctcgccgccgtcgccatgtggagggta
BLZ1 cds  gtggcgtggcggggacggcgacgggcccggcggtggacccggtggagtacaacgccatgctcaagcagaagctcgagaaggacctcgccgccgtcgccatgtggagg---
hpt01     --------------------------------------------------------------------------------------------------------------
hpt02     --------------------------------------------------------------------------------------------------------------
hpt03     --------------------------------------------------------------------------------------------------------------
hpt04     --------------------------------------------------------------------------------------------------------------
hpt05     --------------------------------------------------------------------------------------------------------------
hpt06     --------------------------------------------------------------------------------------------------------------
hpt07     --------------------------------------------------------------------------------------------------------------
hpt08     --------------------------------------------------------------------------------------------------------------

                340       350       360       370       380       390       400       410       420       430       440
         ....|....|....|....|....|....|....|....|....|....|....|....|....|....|....|....|....|....|....|....|....|....|
BLZ1 gene cggccgtcgctctttataccatcttgttaggcgtttcggatgctacgcctgctccgaagtgtttagccgtttttagatcgtcatcgtgcttcgttatctactagtaatgt
BLZ1 cds  --------------------------------------------------------------------------------------------------------------
hpt01     --------------------------------------------------------------------------------------------------------------
hpt02     --------------------------------------------------------------------------------------------------------------
hpt03     --------------------------------------------------------------------------------------------------------------
hpt04     --------------------------------------------------------------------------------------------------------------
hpt05     --------------------------------------------------------------------------------------------------------------
hpt06     --------------------------------------------------------------------------------------------------------------
hpt07     --------------------------------------------------------------------------------------------------------------
hpt08     --------------------------------------------------------------------------------------------------------------
```

```
                   450       460       470       480       490       500       510       520       530       540       550
            ....|....|....|....|....|....|....|....|....|....|....|....|....|....|....|....|....|....|....|....|....|....|
BLZ1 gene   cttctcacggcgtctgtgatctgcagagccctgcattgcctgctcggttctagcaatctgtagatgatgtacaatacggctggttcagtccccgccttgcctatgatatt
BLZ1 cds    --------------------------------------------------------------------------------------------------------------
hpt01       --------------------------------------------------------------------------------------------------------------
hpt02       --------------------------------------------------------------------------------------------------------------
hpt03       --------------------------------------------------------------------------------------------------------------
hpt04       --------------------------------------------------------------------------------------------------------------
hpt05       --------------------------------------------------------------------------------------------------------------
hpt06       --------------------------------------------------------------------------------------------------------------
hpt07       --------------------------------------------------------------------------------------------------------------
hpt08       --------------------------------------------------------------------------------------------------------------

                   560       570       580       590       600       610       620       630       640       650       660
            ....|....|....|....|....|....|....|....|....|....|....|....|....|....|....|....|....|....|....|....|....|....|
BLZ1 gene   ctagcatctgccgccttggctatgatattctagcatctgcgatctggatggtttcgggcaaaatctgtcttcatagtgcatcatttgtccccaaaaaaaacaaaaaacaa
BLZ1 cds    --------------------------------------------------------------------------------------------------------------
hpt01       --------------------------------------------------------------------------------------------------------------
hpt02       --------------------------------------------------------------------------------------------------------------
hpt03       --------------------------------------------------------------------------------------------------------------
hpt04       --------------------------------------------------------------------------------------------------------------
hpt05       --------------------------------------------------------------------------------------------------------------
hpt06       --------------------------------------------------------------------------------------------------------------
hpt07       --------------------------------------------------------------------------------------------------------------
hpt08       --------------------------------------------------------------------------------------------------------------

                   670       680       690       700       710       720       730       740       750       760       770
            ....|....|....|....|....|....|....|....|....|....|....|....|....|....|....|....|....|....|....|....|....|....|
BLZ1 gene   aaaacaaaatggtttatccttatgtgtggacattggctagaatagagatcagagtgtgctgcgagtttgatgattctgtgatttctgagtatttgggtgtcaatagcgat
BLZ1 cds    --------------------------------------------------------------------------------------------------------------
hpt01       --------------------------------------------------------------------------------------------------------------
hpt02       --------------------------------------------------------------------------------------------------------------
hpt03       --------------------------------------------------------------------------------------------------------------
hpt04       --------------------------------------------------------------------------------------------------------------
hpt05       --------------------------------------------------------------------------------------------------------------
hpt06       --------------------------------------------------------------------------------------------------------------
hpt07       --------------------------------------------------------------------------------------------------------------
hpt08       --------------------------------------------------------------------------------------------------------------

                   780       790       800       810       820       830       840       850       860       870       880
            ....|....|....|....|....|....|....|....|....|....|....|....|....|....|....|....|....|....|....|....|....|....|
BLZ1 gene   acatttacactttggtgagcgttgggctcatctgatcctgtcataaagcggtgtgcttgtgcttagctggctgatgatgtcacctttattgtcttctgtctgtttccagt
BLZ1 cds    --------------------------------------------------------------------------------------------------------------
hpt01       --------------------------------------------------------------------------------------------------------------
hpt02       --------------------------------------------------------------------------------------------------------------
hpt03       --------------------------------------------------------------------------------------------------------------
hpt04       --------------------------------------------------------------------------------------------------------------
hpt05       --------------------------------------------------------------------------------------------------------------
hpt06       --------------------------------------------------------------------------------------------------------------
hpt07       --------------------------------------------------------------------------------------------------------------
hpt08       --------------------------------------------------------------------------------------------------------------
```

```
                 890       900       910       920       930       940       950       960       970       980       990
          ....|....|....|....|....|....|....|....|....|....|....|....|....|....|....|....|....|....|....|....|....|....|
BLZ1 gene ttttagaccaatgaaattagcttgtcttgtactccctccatcccataatatagatcttcttacatccaatatgtgagtaaactggatgtaatagatcttatattatggga
BLZ1 cds  --------------------------------------------------------------------------------------------------------------
hpt01     --------------------------------------------------------------------------------------------------------------
hpt02     --------------------------------------------------------------------------------------------------------------
hpt03     --------------------------------------------------------------------------------------------------------------
hpt04     --------------------------------------------------------------------------------------------------------------
hpt05     --------------------------------------------------------------------------------------------------------------
hpt06     --------------------------------------------------------------------------------------------------------------
hpt07     --------------------------------------------------------------------------------------------------------------
hpt08     --------------------------------------------------------------------------------------------------------------

                1000      1010      1020      1030      1040      1050      1060      1070      1080      1090      1100
          ....|....|....|....|....|....|....|....|....|....|....|....|....|....|....|....|....|....|....|....|....|....|
BLZ1 gene cggagggagtatattctagaaatcatatgagcttttgatttttgactccagcacatcaataatttgttttgtttcgttaatcagaacttctctccttgagtttccccact
BLZ1 cds  --------------------------------------------------------------------------------------------------------------
hpt01     --------------------------------------------------------------------------------------------------------------
hpt02     --------------------------------------------------------------------------------------------------------------
hpt03     --------------------------------------------------------------------------------------------------------------
hpt04     --------------------------------------------------------------------------------------------------------------
hpt05     --------------------------------------------------------------------------------------------------------------
hpt06     --------------------------------------------------------------------------------------------------------------
hpt07     --------------------------------------------------------------------------------------------------------------
hpt08     --------------------------------------------------------------------------------------------------------------

                1110      1120      1130      1140      1150      1160      1170      1180      1190      1200      1210
          ....|....|....|....|....|....|....|....|....|....|....|....|....|....|....|....|....|....|....|....|....|....|
BLZ1 gene aaagatgtgttgttaagttgtttgtcttttcctcttactgttatgatattgcaactgtagccttttcaaagaactaaccctaaacactcaatcggggggaggggggagac
BLZ1 cds  --------------------------------------------------------------------------------------------------------------
hpt01     --------------------------------------------------------------------------------------------------------------
hpt02     --------------------------------------------------------------------------------------------------------------
hpt03     --------------------------------------------------------------------------------------------------------------
hpt04     --------------------------------------------------------------------------------------------------------------
hpt05     --------------------------------------------------------------------------------------------------------------
hpt06     --------------------------------------------------------------------------------------------------------------
hpt07     --------------------------------------------------------------------------------------------------------------
hpt08     --------------------------------------------------------------------------------------------------------------

                1220      1230      1240      1250      1260      1270      1280      1290      1300      1310      1320
          ....|....|....|....|....|....|....|....|....|....|....|....|....|....|....|....|....|....|....|....|....|....|
BLZ1 gene agcccctggggggaaatttagaaggtaagggaactgcatcaaacacgctcatgtggggagtcgcactacaggtgccagtgaggggctttgtatgtgagacccaggcattg
BLZ1 cds  --------------------------------------------------------------------------------------------------------------
hpt01     --------------------------------------------------------------------------------------------------------------
hpt02     --------------------------------------------------------------------------------------------------------------
hpt03     --------------------------------------------------------------------------------------------------------------
hpt04     --------------------------------------------------------------------------------------------------------------
hpt05     --------------------------------------------------------------------------------------------------------------
hpt06     --------------------------------------------------------------------------------------------------------------
hpt07     --------------------------------------------------------------------------------------------------------------
hpt08     --------------------------------------------------------------------------------------------------------------
```

```
                   1330      1340      1350      1360      1370      1380      1390      1400      1410      1420      1430
           ....|....|....|....|....|....|....|....|....|....|....|....|....|....|....|....|....|....|....|....|....|....|
BLZ1 gene  aacccgggttggccgaccctctataggaggcgttacctggagctggatagtgtgttcgcttctaagaactaaccctatatttaaaaggacaaattcatgtgcatttttat
BLZ1 cds   --------------------------------------------------------------------------------------------------------------
hpt01      --------------------------------------------------------------------------------------------------------------
hpt02      --------------------------------------------------------------------------------------------------------------
hpt03      --------------------------------------------------------------------------------------------------------------
hpt04      --------------------------------------------------------------------------------------------------------------
hpt05      --------------------------------------------------------------------------------------------------------------
hpt06      --------------------------------------------------------------------------------------------------------------
hpt07      --------------------------------------------------------------------------------------------------------------
hpt08      --------------------------------------------------------------------------------------------------------------

                   1440      1450      1460      1470      1480      1490      1500      1510      1520      1530      1540
           ....|....|....|....|....|....|....|....|....|....|....|....|....|....|....|....|....|....|....|....|....|....|
BLZ1 gene  tttatttttaatgcatttctgttattctgctcatttgtcatactagctcgtgtcatttccgtccgcattttcattttataatgcatttctgttattctgctcatttgtaa
BLZ1 cds   --------------------------------------------------------------------------------------------------------------
hpt01      --------------------------------------------------------------------------------------------------------------
hpt02      --------------------------------------------------------------------------------------------------------------
hpt03      --------------------------------------------------------------------------------------------------------------
hpt04      --------------------------------------------------------------------------------------------------------------
hpt05      --------------------------------------------------------------------------------------------------------------
hpt06      --------------------------------------------------------------------------------------------------------------
hpt07      --------------------------------------------------------------------------------------------------------------
hpt08      --------------------------------------------------------------------------------------------------------------

                   1550      1560      1570      1580      1590      1600      1610      1620      1630      1640      1650
           ....|....|....|....|....|....|....|....|....|....|....|....|....|....|....|....|....|....|....|....|....|....|
BLZ1 gene  tactagctcatgtcattttcgtccgcaggcctctggtgccatgcctccagaacgttttgcagctagtccgtcatgcccaaatgcagatggtcagcatataggcactatta
BLZ1 cds   ----------------------------gcctctggtgccatgcctccagaacgttttgcagctagtccgtcatgcccaaatgcagatggtcagcatataggcactatta
hpt01      --------------------------------------------------------------------------------------------------------CTATTA
hpt02      --------------------------------------------------------------------------------------------------------CTATTA
hpt03      --------------------------------------------------------------------------------------------------------CTATTA
hpt04      --------------------------------------------------------------------------------------------------------CTATTA
hpt05      --------------------------------------------------------------------------------------------------------CTATTA
hpt06      --------------------------------------------------------------------------------------------------------CTATTA
hpt07      --------------------------------------------------------------------------------------------------------CTATTA
hpt08      --------------------------------------------------------------------------------------------------------CTATTA

                   1660      1670      1680      1690      1700      1710      1720      1730      1740      1750      1760
           ....|....|....|....|....|....|....|....|....|....|....|....|....|....|....|....|....|....|....|....|....|....|
BLZ1 gene  atcccataggaggtacttatcttctggtaacatctttgaactagttagga--ctgcggtagggaatgaactaccgtgcatacaatatttgcttaataattcttaaatctt
BLZ1 cds   atcccataggaggt------------------------------------------------------------------------------------------------
hpt01      ATCCCATAGGAGGTACTTATCTTCTGGTAACATCTTTGAACTAGTTAGGAACCTGCGGTAGGGAATGAACTACCGTGCATACCATATTTTCTTAATAATTCTTAAATCTT
hpt02      ATCCCATAGGAGGTACTTATCTTCTGGTAACATCTTTGAACTAGTTAGGAACCTGCGGTAGGGAATGAACTACCGTGCATACAATATTTGCTTAATAGTTCTTAAATCTT
hpt03      ATCCCATAGGAGGTACTTATCTTCTGGTAACATCTTTGAACTAGTTAGGAACCTGCGGTAGGGAATGAACTACCGTGCATACAATATTTGCTTAATAATTCTTAAATCTT
hpt04      ATCCCATAGGAGGTACTTATCTTCTGGTAACATCTTTGAACTAGTTAGGAACCTGCGGTAGGGAATGAACTACCGTGCATACAATATTTGCTTAATAATTCTTAAATCTT
hpt05      ATCCCATAGGAGGTACTTATCTTCTGGTAACATCTTTGAACTAGTTAGGAACCTGCGGTAGGGAATGAACTACCGTGCATACAATATTTGCTTAATAATTCTTAAATCTT
hpt06      ATCCCATAGGAGGTACTTATCTTCTGGTAACATCTTTGAACTAGTTAGGAACCTGCGGTAGGGAATGAACTACCGTGCACACAATATTTGCTTAATAATTCTTAAATCTT
hpt07      ATCCCATAGGAGGTACTTATCTTCTGGTAACATCTTTGAACTAGTTAGGAACCTGCGGTAGGGAATGAACTACCGTGCATACAATATTTGCTTAATAATTCTTAAATCTT
hpt08      ATCCCATAGGAGGTACTTATCTTCTGGTAACATCTTTGAACTAGTTAGGAACCTGCGGTAGGGAATGAACTACCGTGCACACAATATTTGCTTAATAATTCTTAAATCTT
```

```
                   1770      1780      1790      1800      1810      1820      1830      1840      1850      1860      1870
           ....|....|....|....|....|....|....|....|....|....|....|....|....|....|....|....|....|....|....|....|....|....|
BLZ1 gene  tgatttgttacatcttttccctgattctcataaatctggcaatattctttttgaagtgaacggtgttaaattacatttcttggaatcttgttaaaacatttcgt-gtttg
BLZ1 cds   --------------------------------------------------------------------------------------------------------------
hpt01      TGATTTGTTACATCTTTTCCCTGATTCTCATAAATCTGGCAATATTCTTTTTGAAGTGAACGGTATTAAATTACATTTCTTGGAATCTTGTTAAAACATTTCTTTGCTTG
hpt02      TGATTTGTTACATCTTTTCCCTGATTCTCATAAATCTGGCAATATTCTTTTTGAAGTGAACGGTGTTAAATTACATTTCTTGGAATCTTGTTAAAACATTTCTTTGCTTG
hpt03      TGATTTGTTACATCTTTTCCCTGATTCTCATAAATCTGGCAATATTCTTTTTGAAGTGAACGGTGTTAAATTACATTTCTTGGAATCTTGTTAAAACATTTCTTTGCTTG
hpt04      TGATTTGTTACATCTTTTCCCTGATTCTCATAAATCTGGCAATATTCTTTTTGAAGTGAACGGTGTTAAATTACATTTCTTGGAATCTTGTTAAAACATTTCTTTGCTTG
hpt05      TGATTTGTTACATCTTTTCCCTGATTCTCATAAATCTGGCAATATTCTTTTTGAAGTGAACGGTGTTAAATTACATTTCTTGGAATCTTGTTAAAACATTTCTTTGCTTG
hpt06      TGATTTGTTACATCTTTTCCCTGATTCTCATAAATCTGGCAATATTCTTTTTGAAGTGAACGGTGTTAAATTACATTTCTTGGAATCTTGTTAAAACATTTCTTTGCTTG
hpt07      TGATTTGTTACATCTTTTCCCTGATTCTCATAAATCTGGCAATATTCTTTTTGAAGTGAACGGTGTTAAATTACATTTCTTGGAATCTTGTTAAAACATTTCTTTGCTTG
hpt08      TGATTTGTTACATCTTTTCCCTGATTCTCATAAATCTGGCAATATTCTTTTTGAAGTGAACGGTGTTAAATTACATTTCTTGGAATCTTGTTAAAACATTTATTTGCTTG

                   1880      1890      1900      1910      1920      1930      1940      1950      1960      1970      1980
           ....|....|....|....|....|....|....|....|....|....|....|....|....|....|....|....|....|....|....|....|....|....|
BLZ1 gene  c-tgctggcaaccgtgtctgtggctatcctttttcgcagcctaggggggcacactcagtatcctgttttgagttctcagaagaaatcatcctgttttttccttgagcag
BLZ1 cds   --------------------------------------------------------------------------------------------------------------
hpt01      CCTGCTGGCAAGCGTGTTTATGGCTATCCTTTTTTCGCAGCCTAGGGGGGCACACTCAGTATCCTGTTTTGAGTTCTCAGAAGAAATCATCCTGTTTTTTCCTTGAGCAG
hpt02      CCTGCTGGCAAGCGTGTCTGTGGCTATCCTTTTTTCGCAGCCTAGGGGGGCACACTCAGTATCCTGTTTTGAGTTCTCAGAAGAAATCATCCTGTTTTTTCCTTGAGCAG
hpt03      CCTGCTGGCAAGCGTGTCTATGGCTATCCTTTTTTCGCAGCCTAGGGGGGCACACTCAGTATCCTGTTTTGAGTTCTCAGAAGAAATCATCCTGTTTTTTCCTTGAGCAG
hpt04      CCTGCTGGCAAGCGTGTCTGTGGCTATCCTTTTTTCGCAGCCTAGGGGGGCACACTCAGTATCCTGTTTTGAGTTCTCAGAAGAAATCATCCTGTTTTTTCCTTGAGCAG
hpt05      CCTGCTGGCAAGCGTGTCTATGGCTATCCTTTTTTCGCAGCCTAGGGGGGCACACTCAGTATCCTGTTTTGAGTTCTCAGAAGAAATCATCCTGTTTTTTCCTTGAGCAG
hpt06      CCTGCTGGCAAGCGTGTCTATGGCTATCCTTTTTTCACAGCCTAGGGGGGCACACTCAGTATCCTGTTTTGAGTTCTCAGAAGAAATCATCCTGTTTTTTCCTTGAGCAG
hpt07      CCTGCTGGCAAGCGTGTCTATGGCTATCCTTTTTTCGCAGCCTAGGGGGGCACACTCAGTATCCTGTTTTGAGTTCTCAGAAGAAATCATCCTGTTTTTTCCTTGAGCAG
hpt08      CCTGCTGGCAAGCGTGTCTATGGCTATCCTTTTTTCACAGCCTAGGGGGGCACACTCAGTATCCTGTTTTGAGTTCTCAGAAGAAATCATCCTGTTTTTTCCTTGAGCAG

                   1990      2000      2010      2020      2030      2040      2050      2060      2070      2080      2090
           ....|....|....|....|....|....|....|....|....|....|....|....|....|....|....|....|....|....|....|....|....|....|
BLZ1 gene  atagacttcacagtttaactgagtagtgtaaaccatagttccctggaatcgtgttat--gccatgttgtgaaacataaacagaaaagcatattctgtgatgctgacatga
BLZ1 cds   --------------------------------------------------------------------------------------------------------------
hpt01      ATAGACTTCACAGTTTAACTGAGTAGTGTAAACCATAGTTCCCTGGAATCGTGTTATATGCCATGTTGTGAAACATAAACAGAAAAGCATATTCTGTGATGCTGACATGA
hpt02      ATAGACTTCACAGTTTAACTGAGTAGTGTAAACCATAGTTCCCTGGAATCGTGTTAT--GCCATGTTGTGAAACATAAACAGAAAAGCATATTCTGTGATGCTGACATGA
hpt03      ATAGACTTCACAGTTTAACTGAGTAGTGTAAACCATAGTTCCCTGAAATCGTGTTAT--GCCATGTTGTGAAACCTAAACAGAAAAGCATATTCTGTGATGCTGACATGA
hpt04      ATAGACTTCACAGTTTAACTGAGTAGTGTAAACCATAGTTCCCTGGAATCGTGTTAT--GCCATGTTGTGAAACATAAACAGAAAAGCATATTCTGTGATGCTGACATGA
hpt05      ATAGACTTCACAGTTTAACTGAGTAGTGTAAACCATAGTTCCCTGGAATCGTGTTAT--GCCATGTTGTGAAACCTAAACAGAAAAGCATATTCTGTGATGCTGACATGA
hpt06      ATAGACTTCACAGTTTAACTGAGTAGTGTAAACCATAGTTCCCTGGAATCGTGTTATATGCCATGTTGTGAAACATAAACAGAAAAGCATATTCTGTGATGCTGACATGA
hpt07      ATAGACTTCACAGTTTAACTGAGTAGTGTAAACCATAGTTCCCTGGAATCGTGTTAT--GCCATGTTGTGAAACCTAAACAGAAAAGCATATTCTGTGATGCTGACATGA
hpt08      ATAGACTTCACAGTTTAACTGAGTAGTGTAAACCATAGTTCCCTGGAATCGTGTTATATGCCATGTTGTGAAACATAAACAGAAAAGCATATTCTGTGATGCTGACATGA

                   2100      2110      2120      2130      2140      2150      2160      2170      2180      2190      2200
           ....|....|....|....|....|....|....|....|....|....|....|....|....|....|....|....|....|....|....|....|....|....|
BLZ1 gene  catgcctggcttttggacacatatcaggtaatgtggttccacttcaaaacaagctagctggtggcgcaagcggggtgtcgggtccacatctggtacaaaatgctgatgcc
BLZ1 cds   -----------------------------aatgtggttccacttcaaaacaagctagctggtggcgcaagcggggtgtcgggtccacatctggtacaaaatgctgatgcc
hpt01      CATGCCTGGCTTTTGGACACATATCAGGTAATGTGGTTCCACTTCAAAACAAGCTAGCTGGTGGCGCAAGCGGGGTGTCGGGTCCACATCTGGTACAAAATGCTGATGCC
hpt02      CATGCCTGGCTTTTGGACACATATCAGGTAATGTGGTTCCACTTCAAAACAAGCTAGCTGGTGGCGCAAGCGGGGTGTCGGGTCCACATCTGGTACAAAATGCTGATGCC
hpt03      CATGCCTGGCTTTTGGACACATATCAGGTAATGTGGTTCCACTTCAAAACAAGCTAGCTGGTGGCGCAAGCGGGGTGTCGGGTCCACATCTGGTACAAAATGCTGATGCC
hpt04      CATGCCTGGCTTTTGGACACATATCAGGTAATGTGGTTCCACTTCAAAACAAGCTAGCTGGTGGCGCAAGCGGGGTGTCGGGTCCACATCTGGTACAAAATGCTGATGCC
hpt05      CATGCCTGGCTTTTGGACACATATCAGGTAATGTGGTTCCACTTCAAAACAAGCTAGCTGGTGGCGCAAGCGGGGTGTCGGGTCCACATCTGGTACAAAATGCTGATGCC
hpt06      CATGCCTGGCTTTTGGACACATATCAGGTAATGTGGTTCCACTTCAAAACAAGCTAGCTGGTGGCGCAAGCGGGGTGTCGGGTCCACATCTGGTACAAAATGCTGATGCC
hpt07      CATGCCTGGCTTTTGGACACATATCAGGTAATGTGGTTCCACTTCAAAACAAGCTAGCTGGTGGCGCAAGCGGGGTGTCGGGTCCACATCTGGTACAAAATGCTGATGCC
hpt08      CATGCCTGGCTTTTGGACACATATCAGGTAATGTGGTTCCACTTCAAAACAAGCTAGCTGGTGGCGCAAGCGGGGTGTCGGGTCCACATCTGGTACAAAATGCTGATGCC
```

```
                 2210      2220      2230      2240      2250      2260      2270      2280      2290      2300      2310
            ....|....|....|....|....|....|....|....|....|....|....|....|....|....|....|....|....|....|....|....|....|....|
BLZ1 gene   cttgtaaagcaagccgctagctcttcttcgcgggagcagtcagaagatgatgatatggaaggagaagatgagatcactgggaatggggtccctactgatcaaaggctgcg
BLZ1 cds    cttgtaaagcaagccgctagctcttcttcgcgggagcagtcagaagatgatgatatggaaggagaagatgagatcactgggaatggggtccctactgatcaaaggctgcg
hpt01       CTTGTAAAGCAAGCCGCTAG------------------------------------------------------------------------------------------
hpt02       CTTGTAAAGCAAGCCGCTAG------------------------------------------------------------------------------------------
hpt03       CTTGTAAAGCAAGCCGCTAG------------------------------------------------------------------------------------------
hpt04       CTTGTAAAGCAAGCCGCTAG------------------------------------------------------------------------------------------
hpt05       CTTGTAAAGCAAGCCGCTAG------------------------------------------------------------------------------------------
hpt06       CTTGTAAAGCAAGCCGCTAG------------------------------------------------------------------------------------------
hpt07       CTTGTAAAGCAAGCCGCTAG------------------------------------------------------------------------------------------
hpt08       CTTGTAAAGCAAGCCGCTAG------------------------------------------------------------------------------------------

                 2320      2330      2340      2350      2360      2370      2380      2390      2400      2410      2420
            ....|....|....|....|....|....|....|....|....|....|....|....|....|....|....|....|....|....|....|....|....|....|
BLZ1 gene   gaggaggtaatcatttgtaccttcttgtgattatatgcaccatcatgttgtctcatcatgcataatgccccttttcctcaagttagcccttcctttagaatcccccctca
BLZ1 cds    gaggag--------------------------------------------------------------------------------------------------------
hpt01       ------------------------------------------------------------------------------------------------------------CA
hpt02       ------------------------------------------------------------------------------------------------------------CA
hpt03       ------------------------------------------------------------------------------------------------------------CA
hpt04       ------------------------------------------------------------------------------------------------------------CA
hpt05       ------------------------------------------------------------------------------------------------------------CA
hpt06       ------------------------------------------------------------------------------------------------------------CA
hpt07       ------------------------------------------------------------------------------------------------------------CA
hpt08       ------------------------------------------------------------------------------------------------------------CA

                 2430      2440      2450      2460      2470      2480      2490      2500      2510      2520      2530
            ....|....|....|....|....|....|....|....|....|....|....|....|....|....|....|....|....|....|....|....|....|....|
BLZ1 gene   ttcatccctttgccatgttacacag-ag--taattcgtatgggtgttcttatcttggctataggcatagctcgttgcttatatgatttatgattgttcagtggacaagtt
BLZ1 cds    --------------------------------------------------------------------------------------------------------------
hpt01       TTCATCCCTTTGCCATGTTACACAG-AG--TAATTCGTATGGGTGTTCTTATCTTGGCTATAGGCATAGCTCGTTGCTTATATGATTTATGATTGTTCAATGGACAAGTT
hpt02       TTCATCCCTTTGCCATGTTACACAG-AG--TAATTCGTATGGGTGTTCTTATCTTGGCTATAGGCATAGCTCGTTGCTTATATGATTTATGATTGTTCAGTGGACAAGTT
hpt03       TTCATCCCTTTGCCATGTTACACAG-AG--TAATTCGTATGGGTGTTCTTATCTTGGCTATAGGCATAGCTCGTTGCTTATATGATTTATGATTGTTCAATGGACAAGTT
hpt04       TTCATCCCTTTGCCATGTTACACAG-AGAGTAATTCGTATGGGTGTTCTTATCTTGGCTATAGGCATAGCTCGTTGCTTATATGATTTATGATTGTTCAGTGGACAAGTT
hpt05       TTCATCCCTTTGCCATGTTACACAG-AG--TAATTCGTATGGGTGTTCTTATCTTGGCTATAGGCATAGCTCGTTGCTTATATGATTTATGATTGTTCAATGGACAAGTT
hpt06       TTCATCCCTTTGCCATGTTACACAGTAGAGTAATTCGTATGGGTGTTCTTATCTTGGCTATAGGCATAGCTCGTTGCTTATATGATTTATGATTGTTCAATGGACAAGTT
hpt07       TTCATCCCTTTGCCATGTTACACAG-AG--TAATTCGTATGGGTGTTCTTATCTTGGCTATAGGCATAGCTCGTTGCTTATATGATTTATGATTGTTCAATGGACAAGTT
hpt08       TTCATCCCTTTGCCATGTTACACAGTAGAGTAATTCGTATGGGTGTTCTTATCTTGGCTATAGGCATAGCTCGTTGCTTATATGATTTATGATTGTTCAATGGACAAGTT

                 2540      2550      2560      2570      2580      2590      2600      2610      2620      2630      2640
            ....|....|....|....|....|....|....|....|....|....|....|....|....|....|....|....|....|....|....|....|....|....|
BLZ1 gene   aatggttaaaaaaaagtatccatatcatattcgttagatgacttgtctgatactgacggagtgatggtatcaagtagaataaaaaagatcagaatggcctgcttagtaca
BLZ1 cds    --------------------------------------------------------------------------------------------------------------
hpt01       AATGGTTAAAAAAA-GTATCCATATCATATTTGTTAGATGACTTGTCTGATACTGACGGAGTGATGGTATCAAGTAGAATAAAAAAGATCAGAATGGCCTGCTTAGTACA
hpt02       AATGGTTAAAAAAA-GTATCCATATCATATTCGTTAGATGACTTGTCTGATACTGACGGAGTGATGGTATCAAGTAGAATAAAAAAGATCAGAATGGCCTGCTTAGTACA
hpt03       AATGGTTAAAAAAA-GTATCCATATCATATTCGTTAGATGACTTGTCTGATACTGACGGAGTGATGGTATCAAGTAGAATAAAAAAGATCAGAATGGCCTGCTTAGTACA
hpt04       AATGGTTAAAAAAA-GTATCCATATCATATTCGTTAGATGACTTGTCTGATACTGACGGAGTGATGGTATCAAGTAGAATAAAAAAGATCAGAATGGCCTGCTTAGTACA
hpt05       AATGGTTAAAAAAA-GTATCCATATCATATTCGTTAGATGACTTGTCTGATACTGACGGAGTGATGGTATCAAGTAGAATAAAAAAGATCAGAATGGCCTGCTTAGTACA
hpt06       AATGGTTAAAAAAA-GTATCCATATCATATTCGTTAGATGACTTGTCTGATACTGACGGAGTGATGGTATCAAGTAGAATAAAAAAGATCAGAATGGCCTGCTTAGTACA
hpt07       AATGGTTAAAAAAA-GTATCCATATCATATTCGTTAGATGACTTGTCTGATACTGACCGAGTGATGGTATCAAGTAGAATAAAAAAGATCAGAATGGCCTGCTTAGTACA
hpt08       AATGGTTAAAAAAA-GTATCCATATCATATTCGTTAGATGACTTGTCTGATACTGACGGAGTGATGGTATCAAGTAGAATAAAAAAGATCAGAATGGCCTGCTTAGTACA
```

```
                  2650      2660      2670      2680      2690      2700      2710      2720      2730      2740      2750
          ....|....|....|....|....|....|....|....|....|....|....|....|....|....|....|....|....|....|....|....|....|....|
BLZ1 gene tgactgaaaatgataccacggttagatacaagtacaagtatgcacagcgcacaaggctatatggttgcacaatcccatttgggtctttagtaatgtcaaattattgaagt
BLZ1 cds  --------------------------------------------------------------------------------------------------------------
hpt01     TGACTGAAAATGATACCACGGTTAGATACAAGTA------TGCACAGCGCACAAGGCATATAGGTTGCACAATGGCATTTGGGTCTTTAGTAATGTCAAATTATTGAAGT
hpt02     TGACTGAAAATGATACCACGGTTAGATACAAGTA------TGCACAGCGCACAAGGCATATAGGTTGCACAATGGCATTTGGGTCTTTAGTAATGTCAAATTATTGAAGT
hpt03     TGACTGAAAATGATACCACGGTTAGATACAAGTA------TGCACAGCGCACAAGGCATATAGGTTGCACAATGGCATTTGGGTCTTTAGTAATGTCAAATTATTGAAGT
hpt04     TGACTGAAAATGATACCACGGTTAGATACAAGTA------TGCACAGCGCACAAGGCATATAGGTTGCACAATGGCATTTGGGTCTTTAGTAATGTCAAATTATTGAAGT
hpt05     TGACTGAAAATGATACCACGGTTAGATACAAGTA------TGCACAGCGCACAAGGCATATAGGTTGCACAATGGCATTTGGGTCTTTAGTAATGTCAAATTATTGAAGT
hpt06     TGACTGAAAATGATACCACGGTTAGATACAAGTA------TGCACAGCGCACAAGGCATATAGGTTGCACAATGGCATTTGGGTCTTTAGTAATGTCAAATTATTGAAGT
hpt07     TGACTGAAAATGATACCACGGTTAGATACAAGTA------TGCACAGCGCACAAGGCATATAGGTTGCACAATGGCATTTGGGTCTTTAGTAATGTCAAATTATTGAAGT
hpt08     TGACTGAAAATGATACCACGGTTAGATACAAGTA------TGCACAGCGCACAAGGCATATAGGTTGCACAATGGCATTTGGGTCTTTAGTAATGTCAAATTATTGAAGT

                  2760      2770      2780      2790      2800      2810      2820      2830      2840      2850      2860
          ....|....|....|....|....|....|....|....|....|....|....|....|....|....|....|....|....|....|....|....|....|....|
BLZ1 gene gtacatcatggatagctggtttgtgctttggtactccctccgtcccaaaaagcttgttttagatttagtgttagtctgttagatacatccgtatctagataaatctaagg
BLZ1 cds  --------------------------------------------------------------------------------------------------------------
hpt01     GTACATCATGGTATGCTGGTTTGCGCTTTGGTACTCCCTCCGTCCCAAAAAGCTTGTTTTAGATTTAGTGTTAGTCTGTTAGATACATCCGTATCTAGATAAATCTAAGG
hpt02     GTACATCATGGTATGCTGGTTTGTGCTTTGGTACTCCCTCCGTCCCAAAAAGCTTGTTTTAGATTTAGTGTTAGTCTGTTAGATACATCCGTATCTAGATAAATCTAAGG
hpt03     GTACATCATGGTATGCTGGTTTGCGCTTTGGTACTCCCTCCGTCCCAAAAAGCTTGTTTTAGATTTAGTGTTAGTCTGTTAGATACATCCGTATCTAGATAAATCTAAGG
hpt04     GTACATCATGGTATGCTGGTTTGTGCTTTGGTACTCCCTCCGTCCCAAAAAGCTTGTTTTAGATTTAGTGTTAGTCTGTTAGATACATCCGTATCTAGATAAATCTAAGG
hpt05     GTACATCATGGTATGCTGGTTTGCGCTTTGGTACTCCCTCCGTCCCAAAAAGCTTGTTTTAGATTTAGTGTTAGTCTGTTAGATACATCTGTATCTAGATAAATCTAAGG
hpt06     GTACATCATGGTATGCTGGTTTGCGCTTTGGTACTCCCTCCGTCCCAAAAAGCTTGTTTTAGATTTAGTGTTAGTCTGTTAGATACATCCGTATCTAGATAAATCCAAGG
hpt07     GTACATCATGGTATGCTGGTTTGCGCTTTGGTACTCCCTCCGTCCCAAAAAGCTTGTTTTAGATTTAGTGTTAGTCTGTTAGATACATCTGTATCTAGATAAATCTAAGG
hpt08     GTACATCATGGTATGCTGGTTTGCGCTTTGGTACTCCCTCCGTCCCAAAAAGCTTGTTTTAGATTTAGTGTTAGTCTGTTAGATACATCCGTATCTAGATAAATCCAAGG

                  2870      2880      2890      2900      2910      2920      2930      2940      2950      2960      2970
          ....|....|....|....|....|....|....|....|....|....|....|....|....|....|....|....|....|....|....|....|....|....|
BLZ1 gene caagctt---gggatggaggtaatatattttttatcctgaatagtgtaactcctgatgttgtgccggggtctctctctatgacgctattgagcgtccacttcttggatatt
BLZ1 cds  --------------------------------------------------------------------------------------------------------------
hpt01     CAAGCTTTTTGGGATGGAGGTAATATATTTTTTATCCTGAATAGTGTAACTCCTGATGTTGTGCCGGGGTCTCTCTCTATGACGCTATTGA--------------------
hpt02     CAAGCTTTTTGGGATGGAGGTAATATATTTTTTATCCTGAATAGTGTAACTCCTGATGTTGTGCCGGGGTCTCTCTCTATGACGCTATTGA--------------------
hpt03     CAAGCTTTTTGGGATGGAGGTAATATATTTTTTATCCTGAATAGTGTAACTCCTGATGTTGTGCCGGGGTCTCTCTCTATGACGCTATTGA--------------------
hpt04     CAAGCTTTTTGGGATGGAGGTAATATATTTTTTATCCTGAATAGTGTAACTCCTGATGTTGTGCCGGGGTCTCTCTCTATGACGCTATTGA--------------------
hpt05     CAAGCTTTTTGGGATGGAGGTAATATATTTTTTATCCTGAATAGTGTAACTCCTGATGTTGTGCCGGGGTCTCTCTCTATGACGCTATTGA--------------------
hpt06     CAAGCTTTTTGGGATGGAGGTAATATATTTTTTATCCTGAATAGTGTAACTCCTGATGTTGTGCCGGGGTCTCTCTCTATGACGCTATTGA--------------------
hpt07     CAAGCTTTTTGGGATGGAGGTAATATATTTTTTATCCTGAATAGTGTAACTCCTGATGTTGTGCCGGGGTCTCTCTCTATGACGCTATTGA--------------------
hpt08     CAAGCTTTTTGGGATGGAGGTAATATATTTTTTATCCTGAATAGTGTAACTCCTGATGTTGTGCCGGGGTCTCTCTCTATGACGCTATTGA--------------------

                  2980      2990      3000      3010      3020      3030      3040      3050      3060      3070      3080
          ....|....|....|....|....|....|....|....|....|....|....|....|....|....|....|....|....|....|....|....|....|....|
BLZ1 gene atgctagcttcttgactggttccaggaagcaatccaatcgggaatcggccaggcgttcaagaagcagaaaggcagctcacctgaatgaactcgaagcacaggtttgacaa
BLZ1 cds  -------------------------gaagcaatccaatcgggaatcggccaggcgttcaagaagcagaaaggcagctcacctgaatgaactcgaagcacag---------
hpt01     --------------------------------------------------------------------------------------------------------------
hpt02     --------------------------------------------------------------------------------------------------------------
hpt03     --------------------------------------------------------------------------------------------------------------
hpt04     --------------------------------------------------------------------------------------------------------------
hpt05     --------------------------------------------------------------------------------------------------------------
hpt06     --------------------------------------------------------------------------------------------------------------
hpt07     --------------------------------------------------------------------------------------------------------------
hpt08     --------------------------------------------------------------------------------------------------------------
```

```
                3090      3100      3110      3120      3130      3140      3150      3160      3170      3180      3190
          ....|....|....|....|....|....|....|....|....|....|....|....|....|....|....|....|....|....|....|....|....|....|
BLZ1 gene tttacattgttgtttcttgactggcagtggattctgaaaaaggcgctaatgagtttttgttgtggatggctcgttacaggtatcacagttaagagttgaaaactcctcgc
BLZ1 cds  -------------------------------------------------------------------------------gtatcacagttaagagttgaaaactcctcgc
hpt01     --------------------------------------------------------------------------------------------------------------
hpt02     --------------------------------------------------------------------------------------------------------------
hpt03     --------------------------------------------------------------------------------------------------------------
hpt04     --------------------------------------------------------------------------------------------------------------
hpt05     --------------------------------------------------------------------------------------------------------------
hpt06     --------------------------------------------------------------------------------------------------------------
hpt07     --------------------------------------------------------------------------------------------------------------
hpt08     --------------------------------------------------------------------------------------------------------------

                3200      3210      3220      3230      3240      3250      3260      3270      3280      3290      3300
          ....|....|....|....|....|....|....|....|....|....|....|....|....|....|....|....|....|....|....|....|....|....|
BLZ1 gene tgttaaggcggcttgctgatgttaatcagaaatacaatggtgctgctgttgacaatagggtgttaaaggcggatgttgaaaccttaagagcaaaggtacgctcctttatt
BLZ1 cds  tgttaaggcggcttgctgatgttaatcagaaatacaatggtgctgctgttgacaatagggtgttaaaggcggatgttgaaaccttaagagcaaag---------------
hpt01     --------------------------------------------------------------------------------------------------------------
hpt02     --------------------------------------------------------------------------------------------------------------
hpt03     --------------------------------------------------------------------------------------------------------------
hpt04     --------------------------------------------------------------------------------------------------------------
hpt05     --------------------------------------------------------------------------------------------------------------
hpt06     --------------------------------------------------------------------------------------------------------------
hpt07     --------------------------------------------------------------------------------------------------------------
hpt08     --------------------------------------------------------------------------------------------------------------

                3310      3320      3330      3340      3350      3360      3370      3380      3390      3400      3410
          ....|....|....|....|....|....|....|....|....|....|....|....|....|....|....|....|....|....|....|....|....|....|
BLZ1 gene atatgccttatctgctatgcatctgcgtctgttggattggccactagtcttgttccaaatggcatgcccacctcagaccgcacaatacctagtttttagagaaagaatac
BLZ1 cds  --------------------------------------------------------------------------------------------------------------
hpt01     --------------------------------------------------------------------------------------------------------------
hpt02     --------------------------------------------------------------------------------------------------------------
hpt03     --------------------------------------------------------------------------------------------------------------
hpt04     --------------------------------------------------------------------------------------------------------------
hpt05     --------------------------------------------------------------------------------------------------------------
hpt06     --------------------------------------------------------------------------------------------------------------
hpt07     --------------------------------------------------------------------------------------------------------------
hpt08     --------------------------------------------------------------------------------------------------------------

                3420      3430      3440      3450      3460      3470      3480      3490      3500      3510      3520
          ....|....|....|....|....|....|....|....|....|....|....|....|....|....|....|....|....|....|....|....|....|....|
BLZ1 gene gctgcctagtatcctatagtctctatgttggccattcattgtctgctctgaacatacttccacaggaaattttgcaggtgaagatggccgaggactcggtgaagcgggtg
BLZ1 cds  -----------------------------------------------------------------------------gtgaagatggccgaggactcggtgaagcgggtg
hpt01     --------------------------------------------------------------------------------------------------------------
hpt02     --------------------------------------------------------------------------------------------------------------
hpt03     --------------------------------------------------------------------------------------------------------------
hpt04     --------------------------------------------------------------------------------------------------------------
hpt05     --------------------------------------------------------------------------------------------------------------
hpt06     --------------------------------------------------------------------------------------------------------------
hpt07     --------------------------------------------------------------------------------------------------------------
hpt08     --------------------------------------------------------------------------------------------------------------
```

```
                  3530      3540      3550      3560      3570      3580      3590      3600      3610      3620      3630
            ....|....|....|....|....|....|....|....|....|....|....|....|....|....|....|....|....|....|....|....|....|....|
BLZ1 gene   acaggcatgagcgctctgttccctgcagggtccgacatgtcatctctcagcatgcccttcactggctctccatcagaagccacctccgacgctgcgttcccggacgacct
BLZ1 cds    acaggcatgagcgctctgttccctgcagggtccgacatgtcatctctcagcatgcccttcactggctctccatcagaagccacctccgacgctgcgttcccggacgacct
hpt01       --------------------------------------------------------------------------------------------------------------
hpt02       --------------------------------------------------------------------------------------------------------------
hpt03       --------------------------------------------------------------------------------------------------------------
hpt04       --------------------------------------------------------------------------------------------------------------
hpt05       --------------------------------------------------------------------------------------------------------------
hpt06       --------------------------------------------------------------------------------------------------------------
hpt07       --------------------------------------------------------------------------------------------------------------
hpt08       --------------------------------------------------------------------------------------------------------------

                  3640      3650      3660      3670      3680      3690      3700      3710      3720      3730      3740
            ....|....|....|....|....|....|....|....|....|....|....|....|....|....|....|....|....|....|....|....|....|....|
BLZ1 gene   gagcgcttacttctccacaagcgaggctggaggtaacaacggatacatgcccgagatggcttcctcggcgcaagaggatgacaacttcctcaacgagaccatggatacca
BLZ1 cds    gagcgcttacttctccacaagcgaggctggaggtaacaacggatacatgcccgagatggcttcctcggcgcaagaggatgacaacttcctcaacgagaccatggatacca
hpt01       --------------------------------------------------------------------------------------------------------------
hpt02       --------------------------------------------------------------------------------------------------------------
hpt03       --------------------------------------------------------------------------------------------------------------
hpt04       --------------------------------------------------------------------------------------------------------------
hpt05       --------------------------------------------------------------------------------------------------------------
hpt06       --------------------------------------------------------------------------------------------------------------
hpt07       --------------------------------------------------------------------------------------------------------------
hpt08       --------------------------------------------------------------------------------------------------------------

                  3750      3760      3770      3780      3790      3800      3810      3820      3830      3840
            ....|....|....|....|....|....|....|....|....|....|....|....|....|....|....|....|....|....|....|....|.
BLZ1 gene   gcaagatgggcagacccgactcgctgcatcgtgtggcgagcctggagcacctccagaagaggatgtgcggcgggccagcttcgtccggatcgacctcatag
BLZ1 cds    gcaagatgggcagacccgactcgctgcatcgtgtggcgagcctggagcacctccagaagaggatgtgcggcgggccagcttcgtccggatcgacctcatag
hpt01       -----------------------------------------------------------------------------------------------------
hpt02       -----------------------------------------------------------------------------------------------------
hpt03       -----------------------------------------------------------------------------------------------------
hpt04       -----------------------------------------------------------------------------------------------------
hpt05       -----------------------------------------------------------------------------------------------------
hpt06       -----------------------------------------------------------------------------------------------------
hpt07       -----------------------------------------------------------------------------------------------------
hpt08       -----------------------------------------------------------------------------------------------------
```

***BLZ2***

```
           -190      -180      -170      -160      -150      -140      -130      -120      -110      -100      -90
           |....|....|....|....|....|....|....|....|....|....|....|....|....|....|....|....|....|....|....|....|....|....
BLZ2 gene  CCCTCAACCCATCTCATTCCATACACAACCCATGGGTTCGCCATAGCCAAAACCCATGCTGTGCTCGCCGCGTGATTGAGCCTCTCCACCGTCCTCTCTTCGCTGGTTCC
BLZ2 CDS   --------------------------------------------------------------------------------------------------------------
hpt01      --------------------------------------------------------------------------------------------------------------
hpt02      --------------------------------------------------------------------------------------------------------------
hpt03      --------------------------------------------------------------------------------------------------------------
hpt04      --------------------------------------------------------------------------------------------------------------
hpt05      --------------------------------------------------------------------------------------------------------------
hpt06      --------------------------------------------------------------------------------------------------------------
hpt07      --------------------------------------------------------------------------------------------------------------
hpt08      --------------------------------------------------------------------------------------------------------------
hpt09      --------------------------------------------------------------------------------------------------------------
hpt10      --------------------------------------------------------------------------------------------------------------
hpt11      --------------------------------------------------------------------------------------------------------------
hpt12      --------------------------------------------------------------------------------------------------------------
hpt13      --------------------------------------------------------------------------------------------------------------
hpt14      --------------------------------------------------------------------------------------------------------------
hpt15      --------------------------------------------------------------------------------------------------------------
hpt16      --------------------------------------------------------------------------------------------------------------
hpt17      --------------------------------------------------------------------------------------------------------------
hpt18      --------------------------------------------------------------------------------------------------------------
```

```
           -80       -70       -60       -50       -40       -30       -20       -10       -1        10        20
           |....|....|....|....|....|....|....|....|....|....|....|....|....|....|....|...|1...|....|....|....|....|.....
BLZ2 gene  CACGGGGTTAACCATACGATTTCTCGCTCAATTTTGTTCTAATCAATCACCTTGCCCAAATTCCATTCCGGAAACGTGCAATGGAGCCCGTGTTCTCACTGCTGGAGGAG
BLZ2 CDS   --------------------------------------------------------------------------------atggagcccgtgttctcactgctggaggag
hpt01      --------------------------------------------------------------------------------------------------------------
hpt02      --------------------------------------------------------------------------------------------------------------
hpt03      --------------------------------------------------------------------------------------------------------------
hpt04      --------------------------------------------------------------------------------------------------------------
hpt05      --------------------------------------------------------------------------------------------------------------
hpt06      --------------------------------------------------------------------------------------------------------------
hpt07      --------------------------------------------------------------------------------------------------------------
hpt08      --------------------------------------------------------------------------------------------------------------
hpt09      --------------------------------------------------------------------------------------------------------------
hpt10      --------------------------------------------------------------------------------------------------------------
hpt11      --------------------------------------------------------------------------------------------------------------
hpt12      --------------------------------------------------------------------------------------------------------------
hpt13      --------------------------------------------------------------------------------------------------------------
hpt14      --------------------------------------------------------------------------------------------------------------
hpt15      --------------------------------------------------------------------------------------------------------------
hpt16      --------------------------------------------------------------------------------------------------------------
hpt17      --------------------------------------------------------------------------------------------------------------
hpt18      --------------------------------------------------------------------------------------------------------------
```

```
                   40        50        60        70        80        90       100       110       120       130       140
           ....|....|....|....|....|....|....|....|....|....|....|....|....|....|....|....|....|....|....|....|....|....|
BLZ2 gene  GCGATGCCCGAGCCCGACTCTAACCCCGGTCGGACCTCGCCGCCGCAGCTGCAGGCACACGTGCTCGCCGGAGGAGTCAGAGGAGCAGGAGGAGTGGGCGTCGGTGAGAT
BLZ2 CDS   gcgatgcccgagcccgactctaaccccggtcggacctcgccgccgcagctgcaggcacacgtgctcgccggaggagtcagaggagcaggaggagtgggcgtcggtgagat
hpt01      --------------------------------------------------------------------------------------------------------------
hpt02      --------------------------------------------------------------------------------------------------------------
hpt03      --------------------------------------------------------------------------------------------------------------
hpt04      --------------------------------------------------------------------------------------------------------------
hpt05      --------------------------------------------------------------------------------------------------------------
hpt06      --------------------------------------------------------------------------------------------------------------
hpt07      --------------------------------------------------------------------------------------------------------------
hpt08      --------------------------------------------------------------------------------------------------------------
hpt09      --------------------------------------------------------------------------------------------------------------
hpt10      --------------------------------------------------------------------------------------------------------------
hpt11      --------------------------------------------------------------------------------------------------------------
hpt12      --------------------------------------------------------------------------------------------------------------
hpt13      --------------------------------------------------------------------------------------------------------------
hpt14      --------------------------------------------------------------------------------------------------------------
hpt15      --------------------------------------------------------------------------------------------------------------
hpt16      --------------------------------------------------------------------------------------------------------------
hpt17      --------------------------------------------------------------------------------------------------------------
hpt18      --------------------------------------------------------------------------------------------------------------

                  150       160       170       180       190       200       210       220       230       240       250
           ....|....|....|....|....|....|....|....|....|....|....|....|....|....|....|....|....|....|....|....|....|....|
BLZ2 gene  CGTTGGCGATGGCGCGACAGAATTGTGCTTCGACAAGTCCATGGAGGAGCCGTCGCTGCTCAACGTCCCCACGGAGCCAGTGGCGAACCCGGACGCCTCGACGCTTCACC
BLZ2 CDS   cgttggcgatggcgcgacagaattgtgcttcgacaagtccatggaggagccgtcgctgctcaacgtccccacggagccagtggcgaacccggacgcctcgacgcttcacc
hpt01      --------------------------------------------------------------------------------------------------------------
hpt02      --------------------------------------------------------------------------------------------------------------
hpt03      --------------------------------------------------------------------------------------------------------------
hpt04      --------------------------------------------------------------------------------------------------------------
hpt05      --------------------------------------------------------------------------------------------------------------
hpt06      --------------------------------------------------------------------------------------------------------------
hpt07      --------------------------------------------------------------------------------------------------------------
hpt08      --------------------------------------------------------------------------------------------------------------
hpt09      --------------------------------------------------------------------------------------------------------------
hpt10      --------------------------------------------------------------------------------------------------------------
hpt11      --------------------------------------------------------------------------------------------------------------
hpt12      --------------------------------------------------------------------------------------------------------------
hpt13      --------------------------------------------------------------------------------------------------------------
hpt14      --------------------------------------------------------------------------------------------------------------
hpt15      --------------------------------------------------------------------------------------------------------------
hpt16      --------------------------------------------------------------------------------------------------------------
hpt17      --------------------------------------------------------------------------------------------------------------
hpt18      --------------------------------------------------------------------------------------------------------------
```

```
                     260       270       280       290       300       310       320       330       340       350       360
           ....|....|....|....|....|....|....|....|....|....|....|....|....|....|....|....|....|....|....|....|....|....|
BLZ2 gene  CTAATCCCACGGCGGAGGTGAGCCGCAAGAGGCGGTACGACGTTCATGAGGAGGAGGAGGTGGTGGGGGTCATCCCCACGCCGCCTGCGGCGGGCGCGGTGCTGGACCCC
BLZ2 CDS   ctaatcccacggcggaggtgagccgcaagaggcggtacgacgttcatgaggaggaggaggtggtgggggtcatccccacgccgcctgcggcgggcgcggtgctggacccc
hpt01      --------------------------------------------------------------------------------------------------------------
hpt02      --------------------------------------------------------------------------------------------------------------
hpt03      --------------------------------------------------------------------------------------------------------------
hpt04      --------------------------------------------------------------------------------------------------------------
hpt05      --------------------------------------------------------------------------------------------------------------
hpt06      --------------------------------------------------------------------------------------------------------------
hpt07      --------------------------------------------------------------------------------------------------------------
hpt08      --------------------------------------------------------------------------------------------------------------
hpt09      --------------------------------------------------------------------------------------------------------------
hpt10      --------------------------------------------------------------------------------------------------------------
hpt11      --------------------------------------------------------------------------------------------------------------
hpt12      --------------------------------------------------------------------------------------------------------------
hpt13      --------------------------------------------------------------------------------------------------------------
hpt14      --------------------------------------------------------------------------------------------------------------
hpt15      --------------------------------------------------------------------------------------------------------------
hpt16      --------------------------------------------------------------------------------------------------------------
hpt17      --------------------------------------------------------------------------------------------------------------
hpt18      --------------------------------------------------------------------------------------------------------------

                     370       380       390       400       410       420       430       440       450       460       470
           ....|....|....|....|....|....|....|....|....|....|....|....|....|....|....|....|....|....|....|....|....|....|
BLZ2 gene  GTGGGCTACAACGCGATGCTGAGACGGAAGTTGGACGCGCATCTCGCCGCCGTCGCCATGTGGAGGGTACGCTACTGCCCCTACCCCCACCCACCCCTCCTCTACTCGAC
BLZ2 CDS   gtgggctacaacgcgatgctgagacggaagttggacgcgcatctcgccgccgtcgccatgtggagg--------------------------------------------
hpt01      --------------------------------------------------------------------------------------------------------------
hpt02      --------------------------------------------------------------------------------------------------------------
hpt03      --------------------------------------------------------------------------------------------------------------
hpt04      --------------------------------------------------------------------------------------------------------------
hpt05      --------------------------------------------------------------------------------------------------------------
hpt06      --------------------------------------------------------------------------------------------------------------
hpt07      --------------------------------------------------------------------------------------------------------------
hpt08      --------------------------------------------------------------------------------------------------------------
hpt09      --------------------------------------------------------------------------------------------------------------
hpt10      --------------------------------------------------------------------------------------------------------------
hpt11      --------------------------------------------------------------------------------------------------------------
hpt12      --------------------------------------------------------------------------------------------------------------
hpt13      --------------------------------------------------------------------------------------------------------------
hpt14      --------------------------------------------------------------------------------------------------------------
hpt15      --------------------------------------------------------------------------------------------------------------
hpt16      --------------------------------------------------------------------------------------------------------------
hpt17      --------------------------------------------------------------------------------------------------------------
hpt18      --------------------------------------------------------------------------------------------------------------
```

```
                   480       490       500       510       520       530       540       550       560       570       580
           ....|....|....|....|....|....|....|....|....|....|....|....|....|....|....|....|....|....|....|....|....|....|
BLZ2 gene  TCCTTCCTAAGTTCCTAGGCTTGTTTCGATCGTAGTGACTAGGTCATCAGGTGCAAACGTGCGTGGATGGGAGTTCGATGGGAACGATTTGTGATGGAAATATTTTTCGA
BLZ2 CDS   --------------------------------------------------------------------------------------------------------------
hpt01      --------------------------------------------------------------------------------------------------------------
hpt02      --------------------------------------------------------------------------------------------------------------
hpt03      --------------------------------------------------------------------------------------------------------------
hpt04      --------------------------------------------------------------------------------------------------------------
hpt05      --------------------------------------------------------------------------------------------------------------
hpt06      --------------------------------------------------------------------------------------------------------------
hpt07      --------------------------------------------------------------------------------------------------------------
hpt08      --------------------------------------------------------------------------------------------------------------
hpt09      --------------------------------------------------------------------------------------------------------------
hpt10      --------------------------------------------------------------------------------------------------------------
hpt11      --------------------------------------------------------------------------------------------------------------
hpt12      --------------------------------------------------------------------------------------------------------------
hpt13      --------------------------------------------------------------------------------------------------------------
hpt14      --------------------------------------------------------------------------------------------------------------
hpt15      --------------------------------------------------------------------------------------------------------------
hpt16      --------------------------------------------------------------------------------------------------------------
hpt17      --------------------------------------------------------------------------------------------------------------
hpt18      --------------------------------------------------------------------------------------------------------------

                   590       600       610       620       630       640       650       660       670       680       690
           ....|....|....|....|....|....|....|....|....|....|....|....|....|....|....|....|....|....|....|....|....|....|
BLZ2 gene  ACCATAACCATCAGTTGACTAAAATGTTTTCAAGGAAAAAGGTCGAATTTAGCTCAACGAATTAATATGCATGAAATAATTCAAGATTTGCTCAGCRTCACATGTGTAAA
BLZ2 CDS   --------------------------------------------------------------------------------------------------------------
hpt01      --------------------------------------------------------------------------------------------------------------
hpt02      --------------------------------------------------------------------------------------------------------------
hpt03      --------------------------------------------------------------------------------------------------------------
hpt04      --------------------------------------------------------------------------------------------------------------
hpt05      --------------------------------------------------------------------------------------------------------------
hpt06      --------------------------------------------------------------------------------------------------------------
hpt07      --------------------------------------------------------------------------------------------------------------
hpt08      --------------------------------------------------------------------------------------------------------------
hpt09      --------------------------------------------------------------------------------------------------------------
hpt10      --------------------------------------------------------------------------------------------------------------
hpt11      --------------------------------------------------------------------------------------------------------------
hpt12      --------------------------------------------------------------------------------------------------------------
hpt13      --------------------------------------------------------------------------------------------------------------
hpt14      --------------------------------------------------------------------------------------------------------------
hpt15      --------------------------------------------------------------------------------------------------------------
hpt16      --------------------------------------------------------------------------------------------------------------
hpt17      --------------------------------------------------------------------------------------------------------------
hpt18      --------------------------------------------------------------------------------------------------------------
```

```
                    700       710       720       730       740       750       760       770       780       790       800
           ....|....|....|....|....|....|....|....|....|....|....|....|....|....|....|....|....|....|....|....|....|....|
BLZ2 gene  CCAGCCTATCCATGTTGTGTCCATTGGTGGTCTGGCGTCATCTCTAACGAGAACAGCATGCACGTTGCACAAGAGATTCATACTTGGTTAAAGTTTCAAATTGGGTATCT
BLZ2 CDS   --------------------------------------------------------------------------------------------------------------
hpt01      --------------------------------------------------------------------------------------------------------------
hpt02      --------------------------------------------------------------------------------------------------------------
hpt03      --------------------------------------------------------------------------------------------------------------
hpt04      --------------------------------------------------------------------------------------------------------------
hpt05      --------------------------------------------------------------------------------------------------------------
hpt06      --------------------------------------------------------------------------------------------------------------
hpt07      --------------------------------------------------------------------------------------------------------------
hpt08      --------------------------------------------------------------------------------------------------------------
hpt09      --------------------------------------------------------------------------------------------------------------
hpt10      --------------------------------------------------------------------------------------------------------------
hpt11      --------------------------------------------------------------------------------------------------------------
hpt12      --------------------------------------------------------------------------------------------------------------
hpt13      --------------------------------------------------------------------------------------------------------------
hpt14      --------------------------------------------------------------------------------------------------------------
hpt15      --------------------------------------------------------------------------------------------------------------
hpt16      --------------------------------------------------------------------------------------------------------------
hpt17      --------------------------------------------------------------------------------------------------------------
hpt18      --------------------------------------------------------------------------------------------------------------

                    810       820       830       840       850       860       870       880       890       900       910
           ....|....|....|....|....|....|....|....|....|....|....|....|....|....|....|....|....|....|....|....|....|....|
BLZ2 gene  TTTTTACTAAGTTTTTWAAAGAGGATCAGTTATCACGAGCTGGAAAGTCATCGATTAGGAGGGTTGTGGCGACAACAGGGAGAGAGACAATGGAGTTAGCGTTAAGATGT
BLZ2 CDS   --------------------------------------------------------------------------------------------------------------
hpt01      --------------------------------------------------------------------------------------------------------------
hpt02      --------------------------------------------------------------------------------------------------------------
hpt03      --------------------------------------------------------------------------------------------------------------
hpt04      --------------------------------------------------------------------------------------------------------------
hpt05      --------------------------------------------------------------------------------------------------------------
hpt06      --------------------------------------------------------------------------------------------------------------
hpt07      --------------------------------------------------------------------------------------------------------------
hpt08      --------------------------------------------------------------------------------------------------------------
hpt09      --------------------------------------------------------------------------------------------------------------
hpt10      --------------------------------------------------------------------------------------------------------------
hpt11      --------------------------------------------------------------------------------------------------------------
hpt12      --------------------------------------------------------------------------------------------------------------
hpt13      --------------------------------------------------------------------------------------------------------------
hpt14      --------------------------------------------------------------------------------------------------------------
hpt15      --------------------------------------------------------------------------------------------------------------
hpt16      --------------------------------------------------------------------------------------------------------------
hpt17      --------------------------------------------------------------------------------------------------------------
hpt18      --------------------------------------------------------------------------------------------------------------
```

```
                   920       930       940       950       960       970       980       990       1000      1010      1020
           ....|....|....|....|....|....|....|....|....|....|....|....|....|....|....|....|....|....|....|....|....|....|
BLZ2 gene  GTGGAATTTTACATACGGAGAGGACAGATACTTTTTTCACTTTATTTAAATATATATATTTCTCTCGAGAGATTCTTTGTTTCACATATGAATATGGTGCCCCTAAAATA
BLZ2 CDS   --------------------------------------------------------------------------------------------------------------
hpt01      --------------------------------------------------------------------------------------------------------------
hpt02      --------------------------------------------------------------------------------------------------------------
hpt03      --------------------------------------------------------------------------------------------------------------
hpt04      --------------------------------------------------------------------------------------------------------------
hpt05      --------------------------------------------------------------------------------------------------------------
hpt06      --------------------------------------------------------------------------------------------------------------
hpt07      --------------------------------------------------------------------------------------------------------------
hpt08      --------------------------------------------------------------------------------------------------------------
hpt09      --------------------------------------------------------------------------------------------------------------
hpt10      --------------------------------------------------------------------------------------------------------------
hpt11      --------------------------------------------------------------------------------------------------------------
hpt12      --------------------------------------------------------------------------------------------------------------
hpt13      --------------------------------------------------------------------------------------------------------------
hpt14      --------------------------------------------------------------------------------------------------------------
hpt15      --------------------------------------------------------------------------------------------------------------
hpt16      --------------------------------------------------------------------------------------------------------------
hpt17      --------------------------------------------------------------------------------------------------------------
hpt18      --------------------------------------------------------------------------------------------------------------

                   1030      1040      1050      1060      1070      1080      1090      1100      1110      1120      1130
           ....|....|....|....|....|....|....|....|....|....|....|....|....|....|....|....|....|....|....|....|....|....|
BLZ2 gene  TTAACCGTGTATCTTGTTCCACTTTCAGTGGACAAGCACTTCTYATGTTACACTACTCGAGGAATTTGCCGACAAAGCTCCCATGACAATAGAGCATCACAAAATCCAGA
BLZ2 CDS   ---------------------------------------------------actactcgaggaatttgccgacaaagctcccatgacaatagagcatcacaaaatccaga
hpt01      ---------------------------------------------------------------------CGACAAAGCTCCCATGACAATAGAGCATCACAAAATCCAGA
hpt02      ---------------------------------------------------------------------CGACAAAGCTCCCATGACAATAGAGCATCACAAAATCCAGA
hpt03      ---------------------------------------------------------------------CGACAAAGCTCCCATGACAATAGAGCATCACAAAATCCAGA
hpt04      ---------------------------------------------------------------------CGACAAAGCTCCCATGACAATAGAGCATCACAAAATCCAGA
hpt05      ---------------------------------------------------------------------CGACAAAGCTCCCATGACAATAGAGCATCACAAAATCCAGA
hpt06      ---------------------------------------------------------------------CGACAAAGCTCCCATGACAATAGAGCATCACAAAATCCAGA
hpt07      ---------------------------------------------------------------------CGACAAAGCTCCCATGACAATAGAGCATCACAAAATCCAGA
hpt08      ---------------------------------------------------------------------CGACAAAGCTCCCATGACAATAGAGCATCACAAAATCCAGA
hpt09      ---------------------------------------------------------------------CGACAAAGCTCCCATGACAATAGAGCATCACAAAATCCAGA
hpt10      ---------------------------------------------------------------------CGACAAAGCTCCCATGACAATAGAGCATCACAAAATCCAGA
hpt11      ---------------------------------------------------------------------CGACAAAGCTCCCATGACAATAGAGCATCACAAAATCCAGA
hpt12      ---------------------------------------------------------------------CGACAAAGCTCCCATGACAATAGAGCATCACAAAATCCAGA
hpt13      ---------------------------------------------------------------------CGACAAAGCTCCCATGACAATAGAGCATCACAAAATCCAGA
hpt14      ---------------------------------------------------------------------CGACAAAGCTCCCATGACAATAGAGCATCACAAAATCCAGA
hpt15      ---------------------------------------------------------------------CGACAAAGCTCCCATGACAATAGAGCATCACAAAATCCAGA
hpt16      ---------------------------------------------------------------------CGACAAAGCTCCCATGACAATAGAGCATCACAAAATCCAGA
hpt17      ---------------------------------------------------------------------CGACAAAGCTCCCATGACAATAGAGCATCACAAAATCCAGA
hpt18      ---------------------------------------------------------------------CGACAAAGCTCCCATGACAATAGAGCATCACAAAATCCAGA
```

```
                    1140      1150      1160      1170      1180      1190      1200      1210      1220      1230      1240
           ....|....|....|....|....|....|....|....|....|....|....|....|....|....|....|....|....|....|....|....|....|....|
BLZ2 gene  TTCTATCCAAGGCTCAGAAAATCACACTGGAGGTACTCAATTGTGCCTAGTTRCATCTTTCACTACTTATGAAATGATATATGCTAAAAGCTAAGTTGACTTATATACTC
BLZ2 CDS   ttctatccaaggctcagaaaatcacactggag------------------------------------------------------------------------------
hpt01      TTCTATCCAAGGCTCAGAAAATCACACTGGAGGTACTCAATTGTGCCTAGTTACATCTTTCACTACTTATGAAATGATATATGCTAAAAGCTAAGTTGACTTATATACTC
hpt02      TTCTATCCAAGGCTCAGAAAATCACACTGGAGGTACTCAATTGTGCCTAGTTACATCTTTCACTACTTATGAAATGATATATGCTAAAAGCTAAGTTGACTTATATACTC
hpt03      TTCTATCCAAGGCTCAGAAAATCACACTGGAGGTACTCAATTGTGCCTAGTTACATCTTTCACTACTTATGAAATGATATATGCTAAAAGCTAAGTTGACTTATATACTC
hpt04      TTCTATCCAAGGCTCAGAAAATCACACTGGAGGTACTCAATTGTGCCTAGTTACATCTTTCACTACTTATGARATGATATATGCTAAAAGCTAAGTTGACTTATATACTC
hpt05      TTCTATCCAAGGCTCAGAAAATCACACTGGAGGTACTCAATTGTGCCTAGTTACATCTTTCACTACTTATGAAATGATATATGCTAAAAGCTAAGTTGACTTATATACTC
hpt06      TTCTATCCAAGGCTCAGAAAATCACACTGGAGGTACTCAATTGTGCCTAGTTACATCTTTCACTACTTATGAAATGATATATGCTAAAAGCTAAGTTGACTTATATACTC
hpt07      TTCTATCCAAGGCTCAGAAAATCACACTGGAGGTACTCAATTGTGCCTAGTTACATCTTTCACTACTTATGAAATGATATATGCTAAAAGCTAAGTTGACTTATATACTC
hpt08      TTCTATCCAAGGCTCAGAAAATCACACTGGAGGTACTCAATTGTGCCTAGTTGCATCTTTCACTACTTATGAAATGATATATGCTAAAAGCTAAGTTGACTTATATACTC
hpt09      TTCTATCCAAGGCTCAGAAAATCACACTGGAGGTACTCAATTGTGCCTAGTTGCATCTTTCACTACTTATGAAATGATATATGCTAAAAGCTAAGTTGACTTATATACTC
hpt10      TTCTATCCAAGGCTCAGAAAATCACACTGGAGGTACTCAATTGTGCCTAGTTACATCTTTCACTACTTATGAAATGATATATGCTAAAAGCTAAGTTGACTTATATACTC
hpt11      TTCTATCCAAGGCTCAGAAAATCACACTGGAGGTACTCAATTGTGCCTAGTTACATCTTTCACTACTTATGAAATGATATATGCTAAAAGCTAAGTTGACTTATATACTC
hpt12      TTCTATCCAAGGCTCAGAAAATCACACTGGAGGTACTCAATTGTGCCTAGTTACATCTTTCACTACTTATGAAATGATATATGCTAAAAGCTAAGTTGACTTATATACTC
hpt13      TTCTATCCAAGGCTCAGAAAATCACACTGGAGGTACTCAATTGTGCCTAGTTACATCTTTCACTACTTATGAAATGATATATGCTAAAAGCTAAGTTGACTTATATACTC
hpt14      TTCTATCCAAGGCTCAGAAAATCACACTGGAGGTACTCAATTGTGCCTAGTTACATCTTTCACTACTTATGAAATGATATATGCTAAAAGCTAAGTTGACTTATATACTC
hpt15      TTCTATCCAAGGCTCAGAAAATCACACTGGAGGTACTCAATTGTGCCTAGTTACATCTTTCACTACTTATGAAATGATATATGCTAAAAGCTAAGTTGACTTATATACTC
hpt16      TTCTATCCAAGGCTCAGAAAATCACACTGGAGGTACTCAATTGTGCCTAGTTACATCTTTCACTACTTATGAAATGATATATGCTAAAAGCTAAGTTGACTTATATACTC
hpt17      TTCTATCCAAGGCTCAGAAAATCACACTGGAGGTACTCAATTGTGCCTAGTTACATCTTTCACTACTTATGAAATGATATATGCTAAAAGCTAAGTTGACTTATATACTC
hpt18      TTCTATCCAAGGCTCAGAAAATCACACTGGAGGTACTCAATTGTGCCTAGTTACATCTTTCACTACTTATGAAATGATATATGCTAAAAGCTAAGTTGACTTATATACTC

                    1250      1260      1270      1280      1290      1300      1310      1320      1330      1340      1350
           ....|....|....|....|....|....|....|....|....|....|....|....|....|....|....|....|....|....|....|....|....|....|
BLZ2 gene  CCTCCGTCCGGAAATACTTGTCCTAAAGATGAATGTATCTAGACTTAATTTTAGTTAYAGATACATCCATTATATTCATTTYTAGGACAAGTATTTTCGGACGRAGGGAG
BLZ2 CDS   --------------------------------------------------------------------------------------------------------------
hpt01      CCTCCGTCCGGAAATACTTGTCCTAAAGATGAATGTATCTAGACTTA-TTTTAGTTACAGATACATCCATTATATTCATTTCTAGGACAAGTATTTTCGGACGGAGGGAG
hpt02      CCTCCGTCCGGAAATACTTGTCCTAAAGATGAATGTATCTAGACTTAATTTTAGTTATAGATACATCCATTATATTCATTTTTAGGACAAGTATTTTCGGACGGAGGGAG
hpt03      CCTCCGTCCAGAAATACTTGTCCTAAAGATGAATGTATCTAGACTTA-TTTTAGTTATAGATACATCCATTATATTCATTTCTAGGACAAGTATTTTTGGACGGAGGGAG
hpt04      CCTCCGTCCGGAAATACTTGTCCTAAAGATGAATGTATCTAGACTTA-TTTTAGTTATAGATACATCCATTATATTCATTTCTAGGACAAGTATTTTTGGACGAAGGGAG
hpt05      CCTCCGTCCGGAAATACTTGTCCTAAAGATGAATGTATCTAGACTTAATTTTAGTTATAGATACATCCATTATATTCATTTTTAGGACAAGTATTTTCGGACGGAGGGAG
hpt06      CCTCCGTCCGGAAATACTTGTCCTAAAGATGAATGTATCTAGACTTAATTTTAGTTATAGATACATCCATTATATTCATTTTTAGGACAAGTATTTTCGGACGGAGGGAG
hpt07      CCTCCGTCCGGAAATACTTGTCCTAAAGATGAATGTATCTAGACTTA-TTTTAGTTATAGATACATCCATTATATTCATTTCTAAGACAAGTATTTTCGGACGGAGGGAG
hpt08      CCTCCGTCCGGAAATACTTGTCCTAAAGATGAATATATCTAGACTTA-TTTTAGTTATAGATACATCCATTATATTCATTTCTAGGACAAGTATTTTCGGACGAAAGGAG
hpt09      CCTCCGTCCGGAAATACTTGTCCTAAAGATGAATATATCTAGACTTA-TTTTAGTTATAGATACATCCATTATATTCATTTCTAGGACAAGTATTTTCGGACGAAAGGAG
hpt10      CCTCCGTCCAGAAATACTTGTCCTAAAGATGAATGTATCTAGACTTA-TTTTAGTTATAGATACATCCATTATATTCATTTCTAGGACAAGTATTTTTGGACGGAGGGAG
hpt11      CCTCCGTCCGGAAATACTTGTCCTAAAGATGAATGTATCTAGACTTA-TTTTAGTTACAGATACATCCATTATATTCATTTCTAGGACAAGTATTTTCGGACGGAGGGAG
hpt12      CCTCCGTCCGGAAATACTTGTCCTAAAGATGAATGTATCTAGACTTAATTTTAGTTATAGATACATCCATTATATTCATTTTTAGGACAAGTATTTTCGGACGGAGGGAG
hpt13      CCTCCGTCCGGAAATACTTGTCCTAAAGATGAATGTATCTAGACTTAATTTTAGTTATAGATACATCCATTATATTCATTTTTAGGACAAGTATTTTCGGACGGAGGGAG
hpt14      CCTCCGTCCGGAAATACTTGTCCTAAAGATGAATGTATCTAGACTTAATTTTAGTTATAGATACATCCATTATATTCATTTTTAGGACAAGTATTTTCGGACGGAGGGAG
hpt15      CCTCCGTCCGGAAATACTTGTCCTAAAGATGAATGTATCTAGACTTAATTTTAGTTATAGATACATCCATTATATTCATTTTTAGGACAAGTATTTTCGGACGGAGGGAG
hpt16      CCTCCGTCCGGAAATACTTGTCCTAAAGATGAATGTATCTAGACTTA-TTTTAGTTACAGATACATCCATTATATTCATTTCTAGGACAAGTATTTTCGGACGGAGGGAG
hpt17      CCTCCGTCCGGAAATACTTGTCCTAAAGATGAATGTATCTAGACTTAATTTTAGTTATAGATACATCCATTATATTCATTTTTAGAACAAGTATTTTCGGACGGAGGGAG
hpt18      CCTCCGTCCGGAAATACTTGTCCTAAAGATGAATGTATCTAGACTTA-TTTTAGTTATAGATACATCCATTATATTCATTTCTAAGACAAGTATTTTCGGACGGAGGGAG
```

```
                 1360      1370      1380      1390      1400      1410      1420      1430      1440      1450      1460
            ....|....|....|....|....|....|....|....|....|....|....|....|....|....|....|....|....|....|....|....|....|....|
BLZ2 gene   TAGTTGTTTCGTTTTACTATTTGTGTGCTTATGGGTAATGAGGTTCACATGCAACAAATATCACGTGGTGGTGCAGATGCTAGTGTGCAACAACTTAGCTCTTCCTCATG
BLZ2 CDS   ----------------------------------------------------------------------------atgctagtgtgcaacaacttagctcttcctcatg
hpt01       TAGTTGTTTCGTTTTACTATTTGTGTGCTTATGGGTAATGAGGTTCACATGCAACAAATATCACGTGGTGGTGCAGATGCTAGTGTGCAACAACTTAGCTCTTCCTCATG
hpt02       TAGTTGTTTCGTTTTACTATTTGTGTGCTTATGGGTAATGAGGTTCACATGCAACAAATATCACGTGGTGGTGCAGATGCTAGTGTGCAACAACTTAGCTCTTCCTCATG
hpt03       TAGTTGTTTCGTTTTACTATTTGTGTGCTTATGGGTAATGAGGTTCACATGCAACAAATATCACGTGGTGGTGCAGATGCTAGTGTGCAACAACTTAGCTCTTCCTCATG
hpt04       TAGTTGTTTCGTTTTACTATTTGTGTGCTTATGGGTAATGAGGTTCACATGCAACAAATATCACGTGGTGGTGCAGATGCTAGTGTGCAACAACTTAGCTCTTCCTCATG
hpt05       TAGTTGTTTCGTTTTACTATTTGTGTGCTTATGGGTAATGAGGTTCACATGCAACAAATATCACGTGGTGGTGCAGATGCTAGTGTGCAACAACTTAGCTCTTCCTCATG
hpt06       TAGTTGTTTCGTTTTACTATTTGTGTGCTTATGGGTAATGAGGTTCACATGCAACAAATATCACGTGGTGGTGCAGATGCTAGTGTGCAACAACTTAGCTCTTCCTCATG
hpt07       TAGCTGTTTCGTTTTACTATTTGTGTGCTTATGGGTAATGAGGTTCACATGCAACAAATATCACGTGGTGGTGCAGATGCTAGTGTGCAACAACTTAGCTCTTCCTCATG
hpt08       TAGTTGTTTCGTTTTACTATTTGTGTGCTTATGGGTAATGAGGTTCACATGCAACAAATATCACGTGGTGGTGCAGATGCTAGTGTGCAACAACTTAGCTCTTCCTCATG
hpt09       TAGTTGTTTCGTTTTACTATTTGTGTGCTTATGGGTAATGAGGTTCACATGCAACAAATATCACGTGGTGGTGCAGATGCTAGTGTGCAACAACTTAGCTCTTCCTCATG
hpt10       TAGTTGTTTCGTTTTACTATTTGTGTGCTTATGGGTAATGAGGTTCACATGCAACAAATATCACGTGGTGGTGCAGATGCTAGTGTGCAACAACTTAGCTCTTCCTCATG
hpt11       TAGTTGTTTCGTTTTACTATTTGTGTGCTTATGGGTAATGAGGTTCACATGCAACAAATATCACGTGGTGGTGCAGATGCTAGTGTGCAACAACTTAGCTCTTCCTCATG
hpt12       TAGTTGTTTCGTTTTACTATTTGTGTGCTTATGGGTAATGAGGTTCACATGCAACAAATATCACGTGGTGGTGCAGATGCTAGTGTGCAACAACTTAGCTCTTCCTCATG
hpt13       TAGTTGTTTCGTTTTACTATTTGTGTGCTTATGGGTAATGAGGTTCACATGCAACAAATATCACGTGGTGGTGCAGATGCTAGTGTGCAACAACTTAGCTCTTCCTCATG
hpt14       TAGTTGTTTCGTTTTACTATTTGTGTGCTTATGGGTAATGAGGTTCACATGCAACAAATATCACGTGGTGGTGCAGATGCTAGTGTGCAACAACTTAGCTCTTCCTCATG
hpt15       TAGTTGTTTCGTTTTACTATTTGTGTGCTTATGGGTAATGAGGTTCACATGCAACAAATATCACGTGGTGGTGCAGATGCTAGTGTGCAACAACTTAGCTCTTCCTCATG
hpt16       TAGTTGTTTCGTTTTACTATTTGTGTGCTTATGGGTAATGAGGTTCACATGCAACAAATATCACGTGGTGGTGCAGATGCTAGTGTGCAACAACTTAGCTCTTCCTCATG
hpt17       TAGTTGTTTCGTTTTACTATTTGTGTGCTTATGGGTAATGAGGTTCACATGCAACAAATATCACGTGGTGGTGCAGATGCTAGTGTGCAACAACTTAGCTCTTCCTCATG
hpt18       TAGCTGTTTCGTTTTACTATTTGTGTGCTTATGGGTAATGAGGTTCACATGCAACAAATATCACGTGGTGGTGCAGATGCTAGTGTGCAACAACTTAGCTCTTCCTCATG
```

```
                 1470      1480      1490      1500      1510      1520      1530      1540      1550      1560      1570
            ....|....|....|....|....|....|....|....|....|....|....|....|....|....|....|....|....|....|....|....|....|....|
BLZ2 gene   GGAGCCATCACCATCGGATGATGATATGGAAGGGGAGGCACAAACAATTGGAACTATGAATATTAGTGCAGAGAAAGTGAACAAAAGGTAACATATGTTGTATATTTATT
BLZ2 CDS    ggagccatcaccatcggatgatgatatggaaggggaggcacaaacaattggaactatgaatattagtgcagagaaagtgaacaaaag-----------------------
hpt01       GGAGCCATCACCATCGGATGATGATATGGAAGGGGAG---------------ACTATGAATATTAGTGCAGAGAAAGTGAACAAAAGGTAACATATGTTGTATATTTATT
hpt02       GGAGCCATCACCATCGGATGATGATATGGAAGGGGAG---------------ACTATGAATATTAGTGCAGAGAAAGTGAACAAAAGGTAACATATGTTGTATATTTATT
hpt03       GGAGCCATCACCATCGGATGATGATATGGAAGGGGAG---------------ACTATGAATATTAGTGCAGAGAAAGTGAACAAAAGGTAACATATGTTGTATATTTATT
hpt04       GGAGCCATCACCATCGGATGATGATATGGAAGGGGAG---------------ACTATGAATATTAGTGCAGAGAAAGTGAACAAAAGGTAACATATGTTGTATATTTATT
hpt05       GGAGCCATCACCATCGGATGATGATATGGAAGGGGAG---------------ACTATGAATATTAGTGCAGAGAAAGTGAACAAAAGGTAACATATGTTGTATATTTATT
hpt06       GGAGCCATCACCATCGGATGATGATATGGAAGGGGAG---------------ACTATGAATATTAGTGCAGAGAAAGTGAACAAAAGGTAACATATGTTGTATATTTATT
hpt07       GGAGCCATCACCATCGGATGATGATATGGAAGGGGAG---------------ACTATGAATATTAGTGCAGAGAAAGTGAACAAAAGGTAACATATGTTGTATATTTATT
hpt08       GGAGCCATCACCATCGGATGATGATATGGAAGGGGAG---------------ACTATGAATATTAGTGCAGAGAAAGTGAACAAAAGGTAACATATGTTGTATATTTATT
hpt09       GGAGCCATCACCATCGGATGATGATATGGAAGGGGAG---------------ACTATGAATATTAGTGCAGAGAAAGTGAACAAAAGGTAACATATGTTGTATATTTATT
hpt10       GGAGCCATCACCATCGGATGATGATATGGAAGGGGAG---------------ACTATGAATATTAGTGCAGAGAAAGTGAACAAAAGGTAACATATGTTGTATATTTATT
hpt11       GGAGCCATCACCATCGGATGATGATATGGAAGGGGAG---------------ACTATGAATATTAGTGCAGAGAAAGTGAACAAAAGGTAACATATGTTGTATATTTATT
hpt12       GGAGCCATCACCATCGGATGATGATATGGAAGGGGAG---------------ACTATGAATATTAGTGCAGAGAAAGTGAACAAAAGGTAACATATGTTGTATATTTATT
hpt13       GGAGCCATCACCATCGGATGATGATATGGAAGGGGAG---------------ACTATGAATATTAGTGCAGAGAAAGTGAACAAAAGGTAACATATGTTGTATATTTATT
hpt14       GGAGCCATCACCATCGGATGATGATATGGAAGGGGAG---------------ACTATGAATATTAGTGCAGAGAAAGTGAACAAAAGGTAACATATGTTGTATATTTATT
hpt15       GGAGCCATCACCATCGGATGATGATATGGAAGGGGAG---------------ACTATGAATATTAGTGCAGAGAAAGTGAACAAAAGGTAACATATGTTGTATATTTATT
hpt16       GGAGCCATCACCATCGGATGATGATATGGAAGGGGAG---------------ACTATGAATATTAGTGCAGAGAAAGTGAACAAAAGGTAACATATGTTGTATATTTATT
hpt17       GGAGCCATCACCATCGGATGATGATATGGAAGGGGAG---------------ACTATGAATATTAGTGCAGAGAAAGTGAACAAAAGGTAACATATGTTGTATATTTATT
hpt18       GGAGCCATCACCATCGGATGATGATATGGAAGGGGAG---------------ACTATGAATATTAGTGCAGAGAAAGTGAACAAAAGGTAACATATGTTGTATATTTATT
```

```
                   1580      1590      1600      1610      1620      1630      1640      1650      1660      1670      1680
           ....|....|....|....|....|....|....|....|....|....|....|....|....|....|....|....|....|....|....|....|....|....|
BLZ2 gene  TTGTGGTCCTTTCATTCAATCCTCAATKTTGTACTATAAAATTGATGCCTGGGCATTAGAATTATAGACCAAAACTTGTGTGTCCATGTTTTTCTCCTTATGCGGAGAYA
BLZ2 CDS   --------------------------------------------------------------------------------------------------------------
hpt01      TTGTGGTCCTTTCATTCAATCCTCAATGTTGTACTATAAAATTGATGCCTGGGCATTAGAATTATAGACCAAAACTTGTGTGTCCATGTTTTTCTCCTTATGCGGAGATA
hpt02      TTGTGGTCCTTTCATTCAATCCTCAATTTTGTACT------TTGATGCCTGGGCATTAGAATTATAGACCAAAACTTGTGTGTCCATGTTTTTCTCCTTATGCGGAGACA
hpt03      TTGTGGTCCTTTCATTGAATCCTCAATGTTGTACTATAAAATTGATGCCTGGGCATTAGAATTATAGACCAAAACTTGTGTGTCCATGTTTTTCTCCTTATGCGGAGATA
hpt04      TTGTGGTCCTTTCATTCAATCCTCAATGTTGTACTATAAAATTGATGCCTGGGCATTAGAATTATAGACCAAAACTTGTGTGTCCATGTTTTTCTCCTTATGCGGAGATA
hpt05      TTGTGGTCCTTTCATTCAATCCTCAATTTTGTACT------TTGATGCCTGGGCATTAGAATTATAGACCAAAACTTGTGTGTCCATGTTTTTCTCCTTATGCGGAGACA
hpt06      TTGTGGTCCTTTCATTCAATCCTCAATTTTGTACT------TTGATGCCTGGGCATTAGAATTATAGACCAAAACTTGTGTGTCCATGTTTTTCTCCTTATGCGGAGACA
hpt07      TTGTGGTCCTTTCATTCAATCCTCAATTTTGTACTATAAAATTGATGCCTGGGCATTAGAATTATAGACCAAAACTTGTGTGTCCATGTTTTTCTCCTTATGCGGAGATA
hpt08      TTGTGGTCCTTTCATTCAATCCTCAATTTTGTACTATAAAATTGATGCCTGGGCATTAGAATTATAGACCAAAACTTGTGTGTCCATGTTTTTCTCCTTATGCGGAGATA
hpt09      TTGTGGTCCTTTCATTCAATCCTCAATTTTGTACTATAAAATTGATGCCTGGGCATTAGAATTATAGACCAAAACTTGTGTGTCCATGTTTTTCTCCTTATGCGGAGATA
hpt10      TTGTGGTCCTTTCATTGAATCCTCAATGTTGTACTATAAAATTGATGCCTGGGCATTAGAATTATAGACCAAAACTTGTGTGTCCATGTTTTTCTCCTTATGCGGAGATA
hpt11      TTGTGGTCCTTTCATTCAATCCTCAATGTTGTACTATAAAATTGATGCCTGGGCATTAGAATTATAGACCAAAACTTGTGTGTCCATGTTTTTCTCCTTATGCGGAGATA
hpt12      TTGTGGTCCTTTCATTCAATCCTCAATTTTGTACT------TTGATGCCTGGGCATTAGAATTATAGACCAAAACTTGTGTGTCCATGTTTTTCTCCTTATGCGGAGACA
hpt13      TTGTGGTCCTTTCATTCAATCCTCAATTTTGTACT------TTGATGCCTGGGCATTAGAATTATAGACCAAAACTTGTGTGTCCATGTTTTTCTCCTTATGCGGAGACA
hpt14      TTGTGGTCCGTTCATTCAATCCTCAATTTTGTACT------TTGATGCCTGGGCATTAGAATTATAGACCAAAACTTGTGTGTCCATGTTTTTCTCCTTATGCGGAGACA
hpt15      TTGTGGTCCTTTCATTCAATCCTCAATTTTGTACT------TTGATGCCTGGGCATTAGAATTATAGACCAAAACTTGTGTGTCCATGTTTTTCTCCTTATGCGGAGACA
hpt16      TTGTGGTCCTTTCATTCAATCCTCAATGTTGTACTATAAAATTGATGCCTGGGCATTAGAATTATAGACCAAAACTTGTGTGTCCATGTTTTTCTCCTTATGCGGAGATA
hpt17      TTGTGGTCCTTTCATTCAATCCTCAATTTTGTACT------TTGATGCCTGGGCATTAGAATTATAGACCAAAACTTGTGTGTCCATGTTTTTCTCCTTATGCGGAGACA
hpt18      TTGTGGTCCTTTCATTCAATCCTCAATTTTGTACTATAAAATTGATGCCTGGGCATTAGAATTATAGACCAAAACTTGTGTGTCCATGTTTTTCTCCTTATGCGGAGATA

                   1690      1700      1710      1720      1730      1740      1750      1760      1770      1780      1790
           ....|....|....|....|....|....|....|....|....|....|....|....|....|....|....|....|....|....|....|....|....|....|
BLZ2 gene  TTTGTATTGTTTTATCTTTAGTTTTCCCTACATCGATTTTGCTAATTTCTTTCTGGCTTTGTACTGGATGGACTTGGCATATACCTTACATTCAATCCAAACTTTCRATT
BLZ2 CDS   --------------------------------------------------------------------------------------------------------------
hpt01      TTTGTATTGTTTTATCTTTAGTTTTCCCTACATCGATTTTGCTAATTTCTTTCTGGCTTTGTACTGGATGGACTTGGCATATACCTTACATTCAATCCAAACTTTCAATT
hpt02      TTTGTATTGTTTTATCTTTAGTTTTCCCTACATCGATTTTGCTAATTTCTTTCTGGCTTTGTACTGGATGGACTTGGCATATACCTTACATTCAATCCAAACTTTCAATT
hpt03      TTTGTATTGTTTTATCTTTAGTTTTCCCTACATCGATTTTGCTAATTTATTTCTGGCTTTGTACGGGATGGACTTGGCATATACCTTACATTCAATCCAAACTTTCAATT
hpt04      TTTGTATTGTTTTATCTTTAGTTTTCCCTACATCGATTTTGCTAATTTCTTTCTGGCTTTGTACTGGATGGACTTGGCATATACCTTACATTCAATCCAAACTTTCAATT
hpt05      TTTGTATTGTTTTATCTTTAGTTTTCCCTACATCGATTTTGCTAATTTCTTTCTGGCTTTGTACTGGATGGACTTGGCATATACCTTACATTCAATCCAAACTTTCAATT
hpt06      TTTGTATTGTTTTATCTTTAGTTTTCCCTACATCGATTTTGCTAATTTCTTTCTGGCTTTGTACTGGATGGACTTGGCATATACCTTACATTCAATCCAAACTTTCAATT
hpt07      TTTGTATTGTTTTATCTTTAGTTTTCCCTACATCGATTTTGCTAATTTCTTTCTGGCTTTGTACTGGATGGACTTGGCATATACCTTACATTCAATCCAAACGTTCGATT
hpt08      TTTGTATTGTTTTATCTTTAGTTTTCCCTACATCGATTTTGCTAATTTCTTTCTGGCTTTGTACTGGATGGACTTGGCATATACCTTACATTCAATCCAAACTTTCGATT
hpt09      TTTGTATTGTTTTATCTTTAGTTTTCCCTACATCGATTTTGCTAATTTCTTTCTGGCTTTGTACTGGATGGACTTGGCATATACCTTACATTCAATCCAAACTTTCGATT
hpt10      TTTGTATTGTTTTATCTTTAGTTTTCCCTACATCGATTTTGCTAATTTATTTCTGGCTTTGTACGGGATGGACTTGGCATATACCTTACATTCAATCCAAACTTTCAATT
hpt11      TTTGTATTGTTTTATCTTTAGTTTTCCCTACATCGATTTTGCTAATTTCTTTCTGGCTTTGTACTGGATGGACTTGGCATATACCTTACATTCAATCCAAACTTTCAATT
hpt12      TTTGTATTGTTTTATCTTTAGTTTTCCCTACATCGATTTTGCTAATTTCTTTCTGGCTTTGTACTGGATGGACTTGGCATATACCTTACATTCAATCCAAACTTTCAATT
hpt13      TTTGTATTGTTTTATCTTTAGTTTTCCCTACATCGATTTTGCTAATTTCTTTCTGGCTTTGTACTGGATGGACTTGGCATATACCTTACATTCAATCCAAACTTTCAATT
hpt14      TTTGTATTGTTTTATCTTTAGTTTTCCCTACATCGATTTTGCTAATTTCTTTCTGGCTTTGTACTGGATGGACTTGGCATATACCTTACATTCAATCCAAACTTTCAATT
hpt15      TTTGTATTGTTTTATCTTTAGTTTTCCCTACATCGATTTTGCTAATTTCTTTCTGGCTTTGTACTGGATGGACTTGGCATATACCTTACATTCAATCCAAACTTTCAATT
hpt16      TTTGTATTGTTTTATCTTTAGTTTTCCCTACATCGATTTTGCTAATTTCTTTCTGGCTTTGTACTGGATGGACTTGGCATATACCTTACATTCAATCCAAACTTTCAATT
hpt17      TTTGTATTGTTTTATCTTTAGTTTTCCCTACATCGATTTTGCTAATTTCTTTCTGGCTTTGTACTGGATGGACTTGGCATATACCTTACATTCAATCCAAACTTTCAATT
hpt18      TTTGTATTGTTTTATCTTTAGTTTTCCCTACATCGATTTTGCTAATTTCTTTCTGGCTTTGTACTGGATGGACTTGGCATATACCTTACATTCAATCCAAACGTTCGATT
```

```
                   1800      1810      1820      1830      1840      1850      1860      1870      1880      1890      1900
           ....|....|....|....|....|....|....|....|....|....|....|....|....|....|....|....|....|....|....|....|....|....|
BLZ2 gene  AATCTTCATGCACCTTGGCACAAACTTGAGAGCACATTTATGAATTGGTGGAGTGTTTCTAYAGACTGATATRTGTACCACATGATGTRCAGCCTGATTTAGCATGGTTA
BLZ2 CDS   --------------------------------------------------------------------------------------------------------------
hpt01      AATCTTCATGCACCTTGGCACAAACTTGAGAGCACATTTATGAATTGGTGGAGTGTTTCTACAGACTGATATATGTACCACATGATGTACAGCCTGATTTAGCATGGTTA
hpt02      AATCTTCATGCACCTTGGCACAAACTTGAGAGCACATTTATGAATTGGTGGAGTGTTTCTACAGACTGATATGTGTACCACATGATGTGCAGCCTGATTTAGCATGGTTA
hpt03      AATCTTCATGCACCTTGGCACAAACTTGAGAGCACATTTATGAATTGGTGGAGTGTTTCTACAGACTGATATATGTACCACATGATGTGCAGCCTGATTTAGCATGGTTA
hpt04      AATCTTCATGCACCTTGGCACAAACTTGAGAGCACATTTATGAATTGGTGGAGTGTTTCTACAGACTGATATATGTACCACATGATGTGCAGCCTGATTTAGCATGGTTA
hpt05      AATCTTCATGCACCTTGGCACAAACTTGAGAGCACATTTATGAATTGGTGGAGTGTTTCTATAGACTGATATGTGTACCACATGATGTGCAGCCTGATTTAGCATGGTTA
hpt06      AATCTTCATGCACCTTGGCACAAACTTGAGAGCACATTTATGAATTGGTGGAGTGTTTCTACAGACTGATATGTGTACCACATGATGTGCAGCCTGATTTAGCATGGTTA
hpt07      AATCTTCATGCACCTTGGCACAAACTTGAGAGCACATTTATGAATTGGTGGAGTGTTTCTACAGACTGATATATGTACCACATGATGTGCAGCCTGATTTARCATGGTTA
hpt08      AATCTCCATGCACCTTGGCACAAACTTGAGAGCACATTTATGAATTGGTGGAGTGTTTCTACAGACTGATATATGTACCACATGATGTGCAGCCTGATTTAGCATGGTTA
hpt09      AATCTCCATGCACCTTGGCACAAACTTGAGAGCACATTTATGAATTGGTGGAGTGTTTCTACAGACTGATATATGTACCACATGATGTGCAGCCTGATTTAGCATGGTTA
hpt10      AATCTTCATGCACCTTGGCACAAACTTGAGAGCACATTTATGAATTGGTGGAGTGTTTCTACAGACTGATATATGTACCACATGATGTGCAGCCTGATTTAGCATGGTTA
hpt11      AATCTTCATGCACCTTGGCACAAACTTGAGAGCACATTTATGAATTGGTGGAGTGTTTCTACAGACTGATATATGTACCACATGATGTACAGCCTGATTTAGCATGGTTA
hpt12      AATCTTCATGCACCTTGGCACAAACTTGAGAGCACATTTATGAATTGGTGGAGTGTTTCTACAGACTGATATGTGTACCACATGATGTGCAGCCTGATTTAGCATGGTTA
hpt13      AATCTTCATGCACCTTGGCACAAACTTGAGAGCACATTTATGAATTGGTGGAGTGTTTCTATAGACTGATATGTGTACCACATGATGTGCAGCCTGATTTAGCATGGTTA
hpt14      AATCTTCATGCACCTTGGCACAAACTTGAGAGCACATTTATGAATTGGTGGAGTGTTTCTACAGACTGATATGTGTACCACATGATGTGCAGCCTGATTTAGCATGGTTA
hpt15      AATCTTCATGCACCTTGGCACAAACTTGAGAGCACATTTATGAATTGGTGGAGTGTTTCTATAGACTGATATGTGTACCACATGATGTGCAGCCTGATTTAGCATGGTTA
hpt16      AATCTTCATGCACCTTGGCACAAACTTGAGAGCACATTTATGAATTGGTGGAGTGTTTCTACAGACTGATATATGTACCACATGATGTACAGCCTGATTTAGCATGGTTA
hpt17      AATCTTCATGCACCTTGGCACAAACTTGAGAGCACATTTATGAATTGGTGGAGTGTTTCTATAGACTGATATGTGTACCACATGATGTGCAGCCTGATTTAGCATGGTTA
hpt18      AATCTTCATGCACCTTGGCACAAACTTGAGAGCACATTTATGAATTGGTGGAGTGTTTCTACAGACTGATATATGTACCACATGATGTGCAGCCTGATTTAACATGGTTA

                   1910      1920      1930      1940      1950      1960      1970      1980      1990      2000      2010
           ....|....|....|....|....|....|....|....|....|....|....|....|....|....|....|....|....|....|....|....|....|....|
BLZ2 gene  GAATTTTTGGTGGAGCTTGGCATGATCACAAACTACATTTTTTTGTCTCGTGCAGTTTTACCCATCTTCTTATTGAAACSGGTGTGCATTCTCATGGGCATTCATGAAAA
BLZ2 CDS   --------------------------------------------------------------------------------------------------------------
hpt01      GAATTTTTGGTGGAGCTTGGCATGATCACAAACTACATTTTTT-GTCTCGTGCAGTTTTACCCATCTTCTTATTGAAACGGGTGTGCATTCTCATGGGCATTCATGAAAA
hpt02      GAATTTTTGGTGGAGCTTGGCATGATCACAAACTACATTTTTTTGTCTCGTGCAGTTTTACCCATCTTCTTATTGAAACGGGTGTGCATTCTCATGGGCATTCATGAAAA
hpt03      GAATTTTTGGTGGAGCTTGGCATGATCACAAACTACATTTTTT-CTCTCGTGCAGTTTTACCCATCTTCTTATTGAAACGGGTGTGCATTCTCATGGGGATTCATGAAAA
hpt04      GAATTTTTGGTGGAGCTTGGCATGATCACAAACTACATTTTTT-GTCTCGTGCAGTTTTACCCATCTTCTTATTGAAACGGGTGTGCATTCTCATGGGCATTCATGAAAA
hpt05      GAATTTTTGGTGGAGCTTGGCATGATCACAAACTACATTTTTTTGTCTCGTGCAGTTTTACCCATCTTCTTATTGAAACGGGTGTGCATTCTCATGGGCATTCATGAAAA
hpt06      GAATTTTTGGTGGAGCTTGGCATGATCACAAACTACATTTTTTTGTCTCGTGCAGTTTTACCCATCTTCTTATTGAAACGGGTGTGCATTCTCATGGGCATTCATGAAAA
hpt07      GAATTTTTGGTGGAGCTTGGCATGATCACAAACTACATTTTTTTGTCTCGTGCAGTTTTACCCATCTTCTTATTGAAACGGGTGTGCATTCTCTTGGGCATTCATGAAAA
hpt08      GAATTTTTGGTGGAGCTTGGCATGATCACAAACTACATTTTTTTGTCTCGTGCAATTTTACCCATCTTCTTATTGAAACSGGTGTGCATTCTCATGGGCATTCATGAAAA
hpt09      GAATTTTTGGTGGAGCTTGGCATGATCACAAACTACATTTTTTTGTCTCGTGCARTTTTACCCATCTTCTTATTGAAACCGGTGTGCATTCTCATGGGCATTCATGAAAA
hpt10      GAATTTTTGGTGGAGCTTGGCATGATCACAAACTACATTTTTT-CTCTCGTGCAGTTTTACCCATCTTCTTATTGAAACGGGTGTGCATTCTCATGGGGATTCATGAAAA
hpt11      GAATTTTTGGTGGAGCTTGGCATGATCACAAACTACATTTTTT-GTCTCGTGCAGTTTTACCCATCTTCTTATTGAAACGGGTGTGCATTCTCATGGGCATTCATGAAAA
hpt12      GAATTTTTGGTGGAGCTTGGCATGATCACAAACTACATTTTTTTGTCTCGTGCAGTTTTACCCATCTTCTTATTGAAACGGGTGTGCATTCTCATGGGCATTCATGAAAA
hpt13      GAATTTTTGGTGGAGCTTGGCATGATCACAAACTACATTTTTTTGTCTCGTGCAGTTTTACCCATCTTCTTATTGAAACGGGTGTGCATTCTCATGGGCATTCATGAAAA
hpt14      GAATTTTTGGTGGAGCTTGGCATGATCACAAACTACATTTTTTTGTCTCGTGCAGTTTTACCCATCTTCTTATTGAAACGGGTGTGCATTCTCATGGGCATTCATGAAAA
hpt15      GAATTTTTGGTGGAGCTTGGCATGATCACAAACTACATTTTTTTGTCTCGTGCAGTTTTACCCATCTTCTTATTGAAACGGGTGTGCATTCTCATGGGCATTCATGAAAA
hpt16      GAATTTTTGGTGGAGCTTGGCATGATCACAAACTACATTTTTT-GTCTCGTGCAGTTTTACCCATCTTCTTATTGAAACGGGTGTGCATTCTCATGGGCATTCATGAAAA
hpt17      GAATTTTTGGTGGAGCTTGGCATGATCACAAACTACATTTTTTTGTCTCGTGCAGTTTTACCCATCTTCTTATTGAAACGGGTGTGCATTCTCATGGGCATTCATGAAAA
hpt18      GAATTTTTGGTGGAGCTTGGCATGATCACAAACTACATTTTTTTGTCTCGTGCAGTTTTACCCATCTTCTTATTGAAACGGGTGTGCATTCTCTTGGGCATTCATGAAAA
```

```
                2020      2030      2040      2050      2060      2070      2080      2090      2100      2110      2120
           ....|....|....|....|....|....|....|....|....|....|....|....|....|....|....|....|....|....|....|....|....|....|
BLZ2 gene  TGTTACACTGAATTACATCGTATGCCAAATACACCAATACAAATTATCATATTTGGAAAACATTTATGAACATAAATTTTTGTTGTCTTCTAGCACTCAAGTCCTAACTT
BLZ2 CDS   --------------------------------------------------------------------------------------------------------------
hpt01      TGTTACACTGAATTACATCGTATGCCAAATACACCAATACAAATTATCATATTTGGAAAACATTTATGAACATAAATTTTTGTTGTCTTCTAGCACTCAAGTCCTAACTT
hpt02      TGTTACACTGAATTACATCGTATGCCAAATACACCAATACAAATTATCATATTTGGAAAACATTTATGAACATAAATTTTTGTTGTCTTCTAGCACTCAAGTCCTAACTT
hpt03      TGTTACACTGAATTACATCGTATGCCAAATACACCAATACAAATTATCATATTTGGAAAACATTTATGAACATAAATTTTTGTTGTCTTCTAGCACTCAAGTCCTAACTT
hpt04      TGTTACACTGAATTACATCGTATGCCAAATACACCAATACAAATTATCATATTTGGAAAACATTTATGAACATAAATTTTTGTTGTCTTCTAGCACTCAAGTCCTAACTT
hpt05      TGTTACACTGAATTACATCGTATGCCAAATACACCAATACAAATTATCATATTTGGAAAACATTTATGAACATAAATTTTTGTTGTCTTCTAGCACTCAAGTCCTAACTT
hpt06      TGTTACACTGAATTACATCGTATGCCAAATACACCAATACAAATTATCATATTTGGAAAACATTTATGAACATAAATTTTTGTTGTCTTCTAGCACTCAAGTCCTAACTT
hpt07      TGTTACACTGAATTACATCGTATGCCAAATACACCAATACAAATTATCATATTTGGAAAACATTTATGAACATAAATTTTTGTTGTCTTCTAGCACTCAAGTCCTAACTT
hpt08      TGTTACACTGAATTACATCGTATGCCAAATACACCAATACAAATTATCATATTTGGAAAACATTTATGAACATAAATTTTTGTTGTCTTCTATCACTCAAGTCCTAACTT
hpt09      TGTTACACTGAATTACATCGTATGCCAAATACACCAATACAAATTATCATATTTGGAAAACATTTATGAACATAAATTTTTGTTGTCTTCTATCACTCAAGTCCTAACTT
hpt10      TGTTACACTGAATTACATCGTATGCCAAATACACCAATACAAATTATCATATTTGGAAAACATTTATGAACATAAATTTTTGTTGTCTTCTAGCACTCAAGTCCTAACTT
hpt11      TGTTACACTGAATTACATCGTATGCCAAATACACCAATACAAATTATCATATTTGGAAAACATTTATGAACATAAATTTTTGTTGTCTTCTAGCACTCAAGTCCTAACTT
hpt12      TGTTACACTGAATTACATCGTATGCCAAATACACCAATACAAATTATCATATTTGGAAAACATTTATGAACATAAATTTTTGTTGTCTTCTAGCACTCAAGTCCTAACTT
hpt13      TGTTACACTGAATTACATCGTATGCCAAATACACCAATACAAATTATCATATTTGGAAAACATTTATGAACATAAATTTTTGTTGTCTTCTAGCACTCAAGTCCTAACTT
hpt14      TGTTACACTGAATTACATCGTATGCCAAATACACCAATACAAATTATCATATTTGGAAAACATTTATGAACATAAATTTTTGTTGTCTTCTAGCACTCAAGTCCTAACTT
hpt15      TGTTACACTGAATTACATCGTATGCCAAATACACCAATACAAATTATCATATTTGGAAAACATTTATGAACATAAATTTTTGTTGTCTTCTAGCACTCAAGTCCTAACTT
hpt16      TGTTACACTGAATTACATCGTATGCCAAATACACCAATACAAATTATCATATTTGGAAAACATTTATGAACATAAATTTTTGTTGTCTTCTAGCACTCAAGTCCTAACTT
hpt17      TGTTACACTGAATTACATCGTATGCCAAATACACCAATACAAATTATCATATTTGGAAAACATTTATGAACATAAATTTTTGTTGTCTTCTAGCACTCAAGTCCTAACTT
hpt18      TGTTACACTGAATTACATCGTATGCCAAATACACCAATACAAATTATCATATTTGGAAAACATTTATGAACATAAATTTTTGTTGTCTTCTAGCACTCAAGTCCTAACTT
```

```
                2130      2140      2150      2160      2170      2180      2190      2200      2210      2220      2230
           ....|....|....|....|....|....|....|....|....|....|....|....|....|....|....|....|....|....|....|....|....|....|
BLZ2 gene  TTGCGCTCTTTCCTTATATGATTTCAGAAAAGAATCTAACCGGGATTCGGCGAGACGCTCAAGGAGTAGAAAAGCAGCTCATACGAAGGAACTAGAGGAGCAAGTATGAC
BLZ2 CDS   ---------------------------aaaagaatctaaccgggattcggcgagacgctcaaggagtagaaaagcagctcatacgaaggaactagaggagcaa-------
hpt01      TTGCGCTCTTTCCTTATATGATTTCAGAAAAGAATCTAACCGGGATTCGGCGAGACGCTCAAGGAGTAGAAAAGCAGCTCATACGAAGGAACTAGAGGAGCAAGTATGAC
hpt02      TTGCGCTCTTTCCTTATATGATTTCAGAAAAGAATCTAACCGGGATTCGGCGAGACGCTCAAGGAGTAGAAAAGCAGCTCATACGAAGGAACTAGAGGAGCAAGTATGAC
hpt03      TTGCGTTCTTTCCTTATATGATTTCAGAAAAGAATCTAACCGGGATTCGGCGAGACGCTCAAGGAGTAGAAAAGCAGCTCATACGAAGGAACTAGAGGAGCAAGTATGAC
hpt04      TTGCGCTCTTTCCTTATATGATTTCAGAAAAGAATCTAACCGGGATTCGGCGAGACGCTCAAGGAGTAGAAAAGCAGCTCATACGAAGGAACTAGAGGAGCAAGTATGAC
hpt05      TTGCGCTCTTTCCTTATATGATTTCAGAAAAGAATCTAACCGGGATTCGGCGAGACGCTCAAGGAGTAGAAAAGCAGCTCATACGAAGGAACTAGAGGAGCAAGTATGAC
hpt06      TTGCGCTCTTTCCTTATATGATTTCAGAAAAGAATCTAACCGGGATTCGGCGAGACGCTCAAGGAGTAGAAAAGCAGCTCATACGAAGGAACTAGAGGAGCAAGTATGAC
hpt07      TTGCGCTCTTTCCTTATATGATTTCAGAAAAGAATCTAACCGGGATTCAGCGAGACGCTCAAGGAGTAGAAAAGCAGCTCATACGAAGGAACTAGAGGAGCAAGTATGAC
hpt08      TTGCGCTCTTTCCTTATATGATTTCAGAAAAGAATCTAACCGGGATTCGGCGAGACGCTCAAGGAGTAGAAAAGCAGCTCATACGAAGGAACTAGAGGAGCAAGTATGAC
hpt09      TTGCGCTCTTTCCTTATATGATTTCAGAAAAGAATCTAACCGGGATTCGGCGAGACGCTCAAGGAGTAGAAAAGCAGCTCATACGAAGGAACTAGAGGAGCAAGTATGAC
hpt10      TTGCGTTCTTTCCTTATATGATTTCAGAAAAGAATCTAACCGGGATTCGGCGAGACGCTCAAGGAGTAGAAAAGCAGCTCATACGAAGGAACTAGAGGAGCAAGTATGAC
hpt11      TTGCGCTCTTTCCTTATATGATTTCAGAAAAGAATCTAACCGGGATTCGGCGAGACGCTCAAGGAGTAGAAAAGCAGCTCATACGAAGGAACTAGAGGAGCAAGTATGAC
hpt12      TTGCGCTCTTTCCTTATATGATTTCAGAAAAGAATCTAACCGGGATTCGGCGAGACGCTCAAGGAGTAGAAAAGCAGCTCATACGAAGGAACTAGAGGAGCAAGTATGAC
hpt13      TTGCGCTCTTTCCTTATATGATTTCAGAAAAGAATCTAACCGGGATTCGGCGAGACGCTCAAGGAGTAGAAAAGCAGCTCATACGAAGGAACTAGAGGAGCAAGTATGAC
hpt14      TTGCGCTCTTTCCTTATATGATTTCAGAAAAGAATCTAACCGGGATTCGGCGAGACGCTCAAGGAGTAGAAAAGCAGCTCATACGAAGGAACTAGAGGAGCAAGTATGAC
hpt15      TTGCGCTCTTTCCTTATATGATTTCAGAAAAGAATCTAACCGGGATTCGGCGAGACGCTCAAGGAGTAGAAAAGCAGCTCATACGAAGGAACTAGAGGAGCAAGTATGAC
hpt16      TTGCGCTCTTTCCTTATATGATTTCAGAAAAGAATCTAACCGGGATTCGGCGAGACGCTCAAGGAGTAGAAAAGCAGCTCATACGAAGGAACTAGAGGAGCAAGTATGAC
hpt17      TTGCGCTCTTTCCTTATATGATTTCAGAAAAGAATCTAACCGGGATTCGGCGAGACGCTCAAGGAGTAGAAAAGCAGCTCATACGAAGGAACTAGAGGAGCAAGTATGAC
hpt18      TTGCGCTCTTTCCTTATATGATTTCAGAAAAGAATCTAACCGGGATTCAGCGAGACGCTCAAGGAGTAGAAAAGCAGCTCATACGAAGGAACTAGAGGAGCAAGTATGAC
```

```
                   2240      2250      2260      2270      2280      2290      2300      2310      2320      2330      2340
          ....|....|....|....|....|....|....|....|....|....|....|....|....|....|....|....|....|....|....|....|....|....|
BLZ2 gene ATTTTAAACAATATTTTACTTCTCGAGATCAAAATTGCATACTATGGGAGCAAGGAATCTGATGACTACTGCTAAAAAA-TGCA--GTCTCACTATTAAGAGTTGCAAAT
BLZ2 CDS  --------------------------------------------------------------------------------------gtctcactattaagagttgcaaat
hpt01     ATTTTAAACAATATTTTACTTCTCGAGATCAAAATTGCATACTATGGGAACAAGGAATCTGATGATTAGTGCTAAAAAA-TGCAGAGTCTCACTATTAAGAGTTGCAAAT
hpt02     ATTTTAAACAATATTTTACTTCTCGAGATCAAAATTGCATACTATGGGAGCAAGGAATCTGATGACTACTGCTAAAAAA-TGCAGAGTCTCACTATTAAGAGTTGCAAAT
hpt03     ATTTTAAACAATATTTTACT---CGAGATCAAAATTGCATACTATGGGAGCAAGGAATCTGATGACTAGTGCTAAAAAA-TGCAGAGTCTCACTATTAAGAGTTGCAAAT
hpt04     ATTTTAAACAATATTTTACTTCTCGAGATCAAAATTGCATACTATGGGAGCAAGGAATCTGATGACTAGTGCTAAAAAA-TGCAGAGTCTCACTATTAAGAGTTGCAAAT
hpt05     ATTTTAAACAATATTTTACTTCTCGAGATCAAAATTGCATACTATGGGAGCAAGGAATCTGATGACTACTGCTAAAAAA-TGCAGAGTCTCACTATTAAGAGTTGCAAAT
hpt06     ATTTTAAACAATATTTTACTTCTCGAGATCAAAATTGCATACTATGGGAGCAAGGAATCTGATGACTACTGCTAAAAAA-TGCAGAGTCTCACTATTAAGAGTTGCAAAT
hpt07     ATTTTAAACAATATTTTACTTCTCGAGATCAAAATTGCATACTATGGGAGCAAGGAATCTGATGACTACTGCTAAAAAAATGCAGAGTCTCACTATTAAGAGTTGCAAAT
hpt08     ATTTTAAACAATATTTTACTTCTCGAGATCAAAATTGCATACTATGGGAGCAAGGAATCTGATGACTACTGCTAAAAAA-TGCAGAGTCTCACTATTAAGAGTTGCAAAT
hpt09     ATTTTAAACAATATTTTACTTCTCGAGATCAAAATTGCATACTATGGGAGCAAGGAATCTGATGACTACTGCTAAAAAA-TGCAG-GTCTCACTATTAAGAGTTGCAAAT
hpt10     ATTTTAAACAATATTTTACT---CGAGATCAAAATTGCATACTATGGGAGCAAGGAATCTGATGACTAGTGCTAAAAAA-TGCAG-GTCTCACTATTAAGAGTTGCAAAT
hpt11     ATTTTAAACAATATTTTACTTCTCGAGATCAAAATTGCATACTATGGGAACAAGGAATCTGATGATTAGTGCTAAAAAA-TGCAG-GTCTCACTATTAAGAGTTGCAAAT
hpt12     ATTTTAAACAATATTTTACTTCTCGAGATCAAAATTGCATACTATGGGAGCAAGGAATCTGATGACTACTGCTAAAAAA-TGCAG-GTCTCACTATTAAGAGTTGCAAAT
hpt13     ATTTTAAACAATATTTTACTTCTCGAGATCAAAATTGCATACTATGGGAGCAAGGAATCTGATGACTACTGCTAAAAAA-TGCAG-GTCTCACTATTAAGAGTTGCAAAT
hpt14     ATTTTAAACAATATTTTACTTCTCGAGATCAAAATTGCATACTATGGGAGCAAGGAATCTGATGACTACTGCTAAAAAA-TGCAG-GTCTCACTATTAAGAGTTGCAAAT
hpt15     ATTTTAAACAATATTTTACTTCTCGAGATCAAAATTGCATACTATGGGAGCAAGGAATCTGATGACTACTGCTAAAAAA-TGCAG-GTCTCACTATTAAGAGTTGCAAAT
hpt16     ATTTTAAACAATATTTTACTTCTCGAGATCAAAATTGCATACTATGGGAACAAGGAATCTGATGATTAGTGCTAAAAAA-TGCAG-GTCTCACTATTAAGAGTTGCAAAT
hpt17     ATTTTAAACAATATTTTACTTCTCGAGATCAAAATTGCATACTATGGGAGCAAGGAATCTGATGACTACTGCTAAAAAA-TGCAG-GTCTCACTATTAAGAGTTGCAAAT
hpt18     ATTTTAAACAATATTTTACTTCTCGAGATCAAAATTGCATACTATGGGAGCAAGGAATCTGATGACTACTGCTAAAAAAATGCAG-GTCTCACTATTAAGAGTTGCAAAT

                   2350      2360      2370      2380      2390      2400      2410      2420      2430      2440      2450
          ....|....|....|....|....|....|....|....|....|....|....|....|....|....|....|....|....|....|....|....|....|....|
BLZ2 gene AACTCCTTGATGAGACATCTAGCAGATGTAAGTCACAGATACGTCAATACCGCTATTGACAATAGGGTATTGAAGGCAAATGTTGAAACCCTAGAAGCAAAGGTATGTCT
BLZ2 CDS  aactccttgatgagacatctagcagatgtaagtcacagatacgtcaataccgctattgacaatagggtattgaaggcaaatgttgaaaccctagaagcaaag--------
hpt01     AACTCCTTGATGAGACATCTAGCAGATGTAAGTCACAGATACGTCAATACCGCTATTGACAATAGGGTATTGAAGGCAAATGTTGAAACCCTAGAAGCAAAGGTATGTCT
hpt02     AACTCCTTGATGAGACATCTTGCAGATGTAAGTCACAGATACGTCAATACCGCTATTGACAATAGGGTATTGAAGGCAAATGTTGAAACCCTAGAAGCAAAGGTATGTCT
hpt03     AACTCCTTGATGAGACATCTAGCAGATGTAAGTCACAGATACGTCAATACCGCTATTGACAATAGGGTATTGAAGGCAAATGTTGAAACCCTAGAAGCAAAGGTATGTCT
hpt04     AACTCCTTGATGAGACATCTWGCAGATGTAAGTCACAGATACGTCAATACCGCTATTGACAATAGGGTATTGAAGGCAAATGTTGAAACCCTAGAAGCAAAGGTATGTCT
hpt05     AACTCCTTGATGAGACATCTTGCAGATGTAAGTCACAGATACGTCAATACCGCTATTGACAATAGGGTATTGAAGGCAAATGTTGAAACCCTAGAAGCAAAGGTATGTCT
hpt06     AACTCCTTGATGAGACATCTTGCAGATGTAAGTCACAGATACGTCAATACCGCTATTGACAATAGGGTATTGAAGGCAAATGTTGAAACCCTAGAAGCAAAGGTATGTCT
hpt07     AACTCCTTGATGAGACATCTTGCAGATGTAAGTCACAGATACGTCAATACCGCTATTGACAATAGGGTATTGAAGGCAAATGTTGAAACCCTAGAAGCAAAGGTATGTCT
hpt08     AACTCCTTGATGAGACATCTTGCAGATGTAAGTCACAGATACGTCAATACCGCTATTGACAATAGGGTATTGAAGGCAAATGTTGAAACCCTAGAAGCAAAGGTATGTCT
hpt09     AACTCCTTGATGAGACATCTWGCAGATGTAAGTCACAGATACGTCAATACCGCTATTGACAATAGGGTATTGAAGGCAAATGTTGAAACCCTAGAAGCAAAGGTATGTCT
hpt10     AACTCCTTGATGAGACATCTAGCAGATGTAAGTCACAGATACGTCAATACCGCTATTGACAATAGGGTATTGAAGGCAAATGTTGAAACCCTAGAAGCAAAGGTATGTCT
hpt11     AACTCCTTGATGAGACATCTAGCAGATGTAAGTCACAGATACGTCAATACCGCTATTGACAATAGGGTATTGAAGGCAAATGTTGAAACCCTAGAAGCAAAGGTATGTCT
hpt12     AACTCCTTGATGAGACATCTWGCAGATGTAAGTCACAGATACGTCAATACCGCTATTGACAATAGGGTATTGAAGGCAAATGTTGAAACCCTAGAAGCAAAGGTATGTCT
hpt13     AACTCCTTGATGAGACATCTWGCAGATGTAAGTCACAGATACGTCAATACCGCTATTGACAATAGGGTATTGAAGGCAAATGTTGAAACCCTAGAAGCAAAGGTATGTCT
hpt14     AACTCCTTGATGAGACATCTWGCAGATGTAAGTCACAGATACGTCAATACCGCTATTGACAATAGGGTATTGAAGGCAAATGTTGAAACCCTAGAAGCAAAGGTATGTCT
hpt15     AACTCCTTGATGAGACATCTWGCAGATGTAAGTCACAGATACGTCAATACCGCTATTGACAATAGGGTATTGAAGGCAAATGTTGAAACCCTAGAAGCAAAGGTATGTCT
hpt16     AACTCCTTGATGAGACATCTAGCAGATGTAAGTCACAGATACGTCAATACCGCTATTGACAATAGGGTATTGAAGGCAAATGTTGAAACCCTAGAAGCAAAGGTATGTCT
hpt17     AACTCCTTGATGAGACATCTWGCAGATGTAAGTCACAGATACGTCAATACCGCTATTGACAATAGGGTATTGAAGGCAAATGTTGAAACCCTAGAAGCAAAGGTATGTCT
hpt18     AACTCCTTGATGAGACATCTWGCAGATGTAAGTCACAGATACGTCAATACCGCTATTGACAATAGGGTATTGAAGGCAAATGTTGAAACCCTAGAAGCAAAGGTATGTCT
```

```
                    2460      2470      2480      2490      2500      2510      2520      2530      2540      2550      2560
           ....|....|....|....|....|....|....|....|....|....|....|....|....|....|....|....|....|....|....|....|....|....|
BLZ2 gene  TGGTTCTACGCTATTGTGTTACATATTGAATTATGAACATGTGTCAATGCGCATAGCTGCAATGAGTYTCTTTATCYCAATTAAATATTGTGAGAATTTGAAGGATCAAT
BLZ2 CDS   --------------------------------------------------------------------------------------------------------------
hpt01      TGGTTCTACGCTATTGTGTTACATATTGAATTATGAACATGTGTCAATGCGCATAGCTGCAATGAGTTTCTTTATCTCAATTAAATATTGTGAGAATTTGAAGGATCAAT
hpt02      TGGTTCTACGCTATTGTGTTACATATTGAATTATGAACATGTGTCAATGCGCATAGCTGCAATGAGTCTCTTTATCCCAATTAAATATTGTGAGAATTTGAAGGATCAAT
hpt03      TGGTTCTACGCTATTGTGTTACATATTGAATTATGAACATGTGTCAATGCGCATAGCTGCAATGAGTTTCTTTATCCCAATTAAATATTGTGAGAATTTGAAGGATCAAT
hpt04      TGGTTCTACGCTATTGTGTTACATATTGTATTATGAACATGTGTCAATGCGCATAGCTGCAATGAGTTTCTTTATCCCAATTAAATATTGTGAGAATTTGAAGGATCAAT
hpt05      TGGTTCTACGCTATTGTGTTACATATTGAATTATGAACATGTGTCAATGCGCATAGCTGCAATGAGTCTCTTTATCCCAATTAAATATTGTGAGAATTTGAAGGATCAAT
hpt06      TGGTTCTACGCTATTGTGTTACATATTGAATTATGAACATGTGTCAATGCGCATAGCTGCAATGAGTCTCTTTATCCCAATTAAATATTGTGAGAATTTGAAGGATCAAT
hpt07      TGGTTCTACGCTATTGTGTTACATATTCAATTATGAACATGTGTCAATGCGCATAGCTGCAATGAGTTTCTTTATCTCAATTAAATATTGTGAGAATTTGAAGGATCAAT
hpt08      TGGTTCTACGCTATTGTGTTACATATTGAATTATGAACATGTGTCAATGCGCATAGCTGCAATGAGTTTCTTTATCCCAATTAAATATTGTGAGAATTTGAAGGATCAAT
hpt09      TGGTTCTACGCTATTGTGTTACATATTGAATTATGAACATGTGTCAATGCGCATAGCTGCAATGAGTTTCTTTATCCCAATTAAATATTGTGAGAATTTGAAGGATCAAT
hpt10      TGGTTCTACGCTATTGTGTTACATATTGAATTATGAACATGTGTCAATGCGCATAGCTGCAATGAGTTTCTTTATCCCAATTAAATATTGTGAGAATTTGAAGGATCAAT
hpt11      TGGTTCTACGCTATTGTGTTACATATTGAATTATGAACATGTGTCAATGCGCATAGCTGCAATGAGTTTCTTTATCTCAATTAAATATTGTGAGAATTTGAAGGATCAAT
hpt12      TGGTTCTACGCTATTGTGTTACATATTGAATTATGAACATGTGTCAATGCGCATAGCTGCAATGAGTCTCTTTATCCCAATTAAATATTGTGAGAATTTGAAGGATCAAT
hpt13      TGGTTCTACGCTATTGTGTTACATATTGAATTATGAACATGTGTCAATGCGCATAGCTGCAATGAGTCTCTTTATCCCAATTAAATATTGTGAGAATTTGAAGGATCAAT
hpt14      TGGTTCTACGCTATTGTGTTACATATTGAATTATGAACATGTGTCAATGCGCATAGCTGCAATGAGTCTCTTTATCCCAATTAAATATTGTGAGAATTTGAAGGATCAAT
hpt15      TGGTTCTACGCTATTGTGTTACATATTGAATTATGAACATGTGTCAATGCGCATAGCTGCAATGAGTCTCTTTATCCCAATTAAATATTGTGAGAATTTGAAGGATCAAT
hpt16      TGGTTCTACGCTATTGTGTTACATATTGAATTATGAACATGTGTCAATGCGCATAGCTGCAATGAGTTTCTTTATCTCAATTAAATATTGTGAGAATTTGAAGGATCAAT
hpt17      TGGTTCTACGCTATTGTGTTACATATTGAATTATGAACATGTGTCAATGCGCATAGCTGCAATGAGTCTCTTTATCCCAATTAAATATTGTGAGAATTTGAAGGATCAAT
hpt18      TGGTTCTACGCTATTGTGTTACATATTCAATTATGAACATGTGTCAATGCGCATAGCTGCAATGAGTTTCTTTATCTCAATTAAATATTGTGAGAATTTGAAGGATCAAT

                    2570      2580      2590      2600      2610      2620      2630      2640      2650      2660      2670
           ....|....|....|....|....|....|....|....|....|....|....|....|....|....|....|....|....|....|....|....|....|....|
BLZ2 gene  AGTTCACTAGATGTAGGGGTGGATAGGTGACTAACGACGTATATAAACGATATACCCTTTTATGATATTGTTAAAAYGAAAAGGTCGTTTARTGTAGTTCTATATTAACA
BLZ2 CDS   --------------------------------------------------------------------------------------------------------------
hpt01      AGTTCACTAGATGTAGGGGTGGATAGGTGACTAACGACGTATATAAACGATA--CCCTTTTATGATATTGTTAAAATGAAAAGGTCGTTTAATGTAGTTCTATATTAACA
hpt02      AGTTCACTAGATGTAGGGGTGGATAGGTGACTAACGACGTATATAAACGATATACCCTTTTATGATATTGTTAAAACGAAAAGGTCGTTTAGTGTAGTTCTATATTAACA
hpt03      AGTTCACTAGATGTAGGGGTGGATAGGTGACTAACGACGCATATAAACGATATACCCTTTTATGATATTGTTAAAATGAAAAGGTCGTTTAATGTAGTTCTATATTAACA
hpt04      AGTTCACTAGATGTAGGGGTGGATAGGTGACTAACGACGTATATAAACGATATACCCTTTTATGATATTGTTAA--TGAAAAGGTCGTTTAGTGTAGTTCTATATTAACA
hpt05      AGTTCACTAGATGTAGGGGTGGATAGGTGACTAACGACGTATATAAACGATATACCCTTTTATGATATTGTTAAAACGAAAAGGTCGTTTAGTGTAGTTCTATATTAACA
hpt06      AGTTCACTAGATGTAGGGGTGGATAGGTGACTAACGACGTATATAAACGATATACCCTTTTATGATATTGTTAAAACGAAAAGGTCGTTTAGTGTAGTTCTATATTAACA
hpt07      AGTTGACTAGATGTAGGGGTGGATAGGTGACTAACGACGTATATAAACAATATACCCTTTTATGATATTGTTAAAATGAAAAGGTCGTTTAGTGTAGTTCTATATTAACA
hpt08      AGTTCACTAGATGTAGGGGTGGATAGGTGACTAACGACGTATATAAACGATATACCCTTTTATGATATTGTTAAAATGAAAAGGTCGTTTAGTGTAGTTCTATATTAACA
hpt09      AGTTCACTAGATGTAGGGGTGGATAGGTGACTAACGACGTATATAAACGATATACCCTTTTATGATATTGTTAAAATGAAAAGGTCGTTTAGTGTAGTTCTATATTAACA
hpt10      AGTTCACTAGATGTAGGGGTGGATAGGTGACTAACGACGCATATAAACGATATACCCTTTTATGATATTGTTAAAATGAAAAGGTCGTTTAATGTAGTTCTATATTAACA
hpt11      AGTTCACTAGATGTAGGGGTGGATAGGTGACTAACGACGTATATAAACGATA--CCCTTTTATGATATTGTTAAAATGAAAAGGTCGTTTAATGTAGTTCTATATTAACA
hpt12      AGTTCACTAGATGTAGGGGTGGATAGGTGACTAACGACGTATATAAACGATATACCCTTTTATGATATTGTTAAAACGAAAAGGTCGTTTAGTGTAGTTCTATATTAACA
hpt13      AGTTCACTAGATGTAGGGGTGGATAGGTGACTAACGACGTATATAAACGATATACCCTTTTATGATATTGTTAAAACGAAAAGGTCGTTTAGTGTAGTTCTATATTAACA
hpt14      AGTTCACTAGATGTAGGGGTGGATAGGTGACTAACGACGTATATAAACGATATACCCTTTTATGATATTGTTAAAACGAAAAGGTCGTTTAGTGTAGTTCTATATTAACA
hpt15      AGTTCACTAGATGTAGGGGTGGATAGGTGACTAACGACGTATATAAACGATATACCCTTTTATGATATTGTTAAAACGAAAAGGTCGTTTAGTGTAGTTCTA-ATTAACA
hpt16      AGTTCACTAGATGTAGGGGTGGATAGGTGACTAACGACGTATATAAACGATA--CCCTTTTATGATATTGTTAAAATGAAAAGGTCGTTTAATGTAGTTCTATATTAACA
hpt17      AGTTCACTAGATGTAGGGGTGGATAGGTGACTAACGACGTATATAAACGATATACCCTTTTATGATATTGTTAAAACGAAAAGGTCGTTTAGTGTAGTTCTATATTAACA
hpt18      AGTTGACTAGATGTAGGGGTGGATAGGTGACTAACGACGTATATAAACAATATACCCTTTTATGATATTGTTAAAATGAAAAGGTCGTTTAGTGTAGTTCTATATTAACA
```

```
                  2680      2690      2700      2710      2720      2730      2740      2750      2760      2770      2780
            ....|....|....|....|....|....|....|....|....|....|....|....|....|....|....|....|....|....|....|....|....|....|
BLZ2 gene   AATTAGTGATGAGAAAGCTGCAATAACAACACAAACAATTCAGGCAATTGTGCAAGTGAAGTATGAAAAAAATGTAAYATACCGAGAAAATAGGTGTGAAATGTTGATGT
BLZ2 CDS    --------------------------------------------------------------------------------------------------------------
hpt01       AATTAGTGATGAGAAAGCTGCAATAACAACACAAACAATTCAGGCAATTGTGCAAGTGAAGTATGAAAAAAATGTAATATACCGAGAAAATAGGTGTGAAATGTTGATGT
hpt02       AATTAGTGATGAGAAAGCTGCAATAACAACACAAACAATTCAGGCAATTGTGCAAGTGAAGTATGAAAAAAATGTAACATACCGAGAAAATAGGTGTGAAATGTTGATGT
hpt03       AATTAGTGATGAGAAAGCTGCAATAACAACACAAACAATTCAGGCAATTGTGCAAGTGAAGTATGAAAAAAAAGTAACATACCGAGAAAATAGGTGTGAAATGTTGATGT
hpt04       AATTAGTGATGAGAAAGCTGCAATAACAACACAAACAATTCAGGCAATTGTGCAAGTGAAGTATGAAAAAAATGTAACATACCGAGAAAATAGGTGTGAAATGTTGATGT
hpt05       AATTAGTGATGAGAAAGCTGCAATAACAACACAAACAATTCAGGCAATTGTGCAAGTGAAGTATGAAAAAAATGTAACATACCGAGAAAATAGGTGTGAAATGTTGATGT
hpt06       AATTAGTGATGAGAAAGCTGCAATAACAACACAAACAATTCAGGCAATTGTGCAAGTGAAGTATGAAAAAAATGTAACATACCGAGAAAATAGGTGTGAAATGTTGATGT
hpt07       AATTAGTGATGAGAAAGCTGCAATAACAACACAAACAATTCAGGCAATTGTGCAAGTGAAGTATGAAAAAAATGTAACATACCGAGAAAATAGGTGTGAAATGTTGATGT
hpt08       AATTAGTGATGAGAAAGCTGCAATAACAACACAAACAATTCAGGCAATTGTGCAAGTGAAGTATGAAAAAAATGTAACATACCGAGAAAATAGGTGTGAAATGTTGATGT
hpt09       AATTAGTGATGAGAAAGCTGCAATAACAACACAAACAATTCAGGCAATTGTGCAAGTGAAGTATGAAAAAAATGTAACATACCGAGAAAATAGGTGTGAAATGTTGATGT
hpt10       AATTAGTGATGAGAAAGCTGCAATAACAACACAAACAATTCAGGCAATTGTGCAAGTGAAGTATGAAAAAAAAGTAACATACCGAGAAAATAGGTGTGAAATGTTGATGT
hpt11       AATTAGTGATGAGAAAGCTGCAATAACAACACAAACAATTCAGGCAATTGTGCAAGTGAAGTATGAAAAAAATGTAATATACCGAGAAAATAGGTGTGAAATGTTGATGT
hpt12       AATTAGTGATGAGAAAGCTGCAATAACAACACAAACAATTCAGGCAATTGTGCAAGTGAAGTATGAAAAAAATGTAACATACCGAGAAAATAGGTGTGAAATGTTGATGT
hpt13       AATTAGTGATGAGAAAGCTGCAATAACAACACAAACAATTCAGGCAATTGTGCAAGTGAAGTATGAAAAAAATGTAACATACCGAGAAAATAGGTGTGAAATGTTGATGT
hpt14       AATTAGTGATGAGAAAGCTGCAATAACAACACAAACAATTCAGGCAATTGTGCAAGTGAAGTATGAAAAAAATGTAACATACCGAGAAAATAGGTGTGAAATGTTGATGT
hpt15       AATTAGTGATGAGAAAGCTGCAATAACAACACAAACAATTCAGGCAATTGTGCAAGTGAAGTATGAAAAAAATGTAACATACCGAGAAAATAGGTGTGAAATGTTGATGT
hpt16       AATTAGTGATGAGAAAGCTGCAATAACAACACAAACAATTCAGGCAATTGTGCAAGTGAAGTATGAAAAAAATGTAATATACCGAGAAAATAGGTGTGAAATGTTGATGT
hpt17       AATTAGTGATGAGAAAGCTGCAATAACAACACAAACAATTCAGGCAATTGTGCAAGTGAAGTATGAAAAAAATGTAACATACCGAGAAAATAGGTGTGAAATGTTGATGT
hpt18       AATTAGTGATGAGAAAGCTGCAATAACAACACAAACAATTCAGGCAATTGTGCAAGTGAAGTATGAAAAAAATGTAACATACCGAGAAAATAGGTGTGAAATGTTGATGT

                  2790      2800      2810      2820      2830      2840      2850      2860      2870      2880      2890
            ....|....|....|....|....|....|....|....|....|....|....|....|....|....|....|....|....|....|....|....|....|....|
BLZ2 gene   AGASCYGAAGTTCAAATCCTTGGGTGATTCCATGCATCCAATGGAGAGGCATGCATACAAAGTCTTTATGGCCACAAATTTCCCTTCTTCTCCTTGAGATTCTTGCATTA
BLZ2 CDS    --------------------------------------------------------------------------------------------------------------
hpt01       AGACCCGAAGTTCAAATCCTTGGGTGATTCCATGCATCCAATGGAGAGGCATGCATACAAAGTCTTTATGGCCACAAATTTCCCTTCTTCTCCTTGAGATTCTTGCATTA
hpt02       AGAGCTGAAGTTCAAATCCTTGGGTGATTCCATGCATCCAATGGAGAGGCATGCATACAAAGTCTTTATGGCCACAAATTTCCCTTCTTCTCCTTGAGATTCTTGCATTA
hpt03       AGACCTGAAGTTCAAATCCTTGGGTGATTCCATGCATCCAATGGAGAGGCATGCATACAAAGTCTTTATGGCCACAAATTTCCCTTCTTCTCCTTGAGATTCTTGCATTA
hpt04       AGACCTGAAGTTCAAATCCTTGGGTGATTCCATGCATCCAATGGAGAGGCATGCATACAAAGTCTTTATGGCCACAAATTTCCCTTCTTCTCCTTGAGATTCTTGCATTA
hpt05       AGAGCTGAAGTTCAAATCCTTGGGTGATTCCATGCATCCAATGGAGAGGCATGCATACAAAGTCTTTATGGCCACAAATTTCCCTTCTTCTCCTTGAGATTCTTGCATTA
hpt06       AGAGCTGAAGTTCAAATCCTTGGGTGATTCCATGCATCCAATGGAGAGGCATGCATACAAAGTCTTTATGGCCACAAATTTCCCTTCTTCTCCTTGAGATTCTTGCATTA
hpt07       AGACCTGAAGTTCAAATCCTTGGGTGATTCCATGCATCCAATGGAGAGGCATGCATACAAAGTCTTTATGGCCACAAATTTCCCTTCTTCTCCTTGAGATTCTTGCATTA
hpt08       AGACCTGAAGTTCAAATCCTTGGGTGATTCCATGCATCCAATGGAGAGACATGCATACAAAGTCTTTATGGCCACAAATTTCCCTTCTTCTCCTTGAGATTCTTGCATTA
hpt09       AGACCTGAAGTTCAAATCCTTGGGTGATTCCATGCATCCAATGGAGAGRCATGCATACAAAGTCTTTATGGCCACAAATTTCCCTTCTTCTCCTTGAGATTCTTGCATTA
hpt10       AGACCTGAAGTTCAAATCCTTGGGTGATTCCATGCATCCAATGGAGAGGCATGCATACAAAGTCTTTATGGCCACAAATTTCCCTTCTTCTCCTTGAGATTCTTGCATTA
hpt11       AGACCCGAAGTTCAAATCCTTGGGTGATTCCATGCATCCAATGGAGAGGCATGCATACAAAGTCTTTATGGCCACAAATTTCCCTTCTTCTCCTTGAGATTCTTGCATTA
hpt12       AGAGCTGAAGTTCAAATCCTTGGGTGATTCCATGCATCCAATGGAGAGGCATGCATACAAAGTCTTTATGGCCACAAATTTCCCTTCTTCTCCTTGAGATTCTTGCATTA
hpt13       AGAGCTGAAGTTCAAATCCTTGGGTGATTCCATGCATCCAATGGAGAGGCATGCATACAAAGTCTTTATGGCCACAAATTTCCCTTCTTCTCCTTGAGATTCTTGCATTA
hpt14       AGAGCTGAAGTTCAAATCCTTGGGTGATTCCATGCATCCAATGGAGAGGCATGCATACAAAGTCTTTATGGCCACAAATTTCCCTTCTTCTCCTTGAGATTCTTGCATTA
hpt15       AGAGCTGAAGTTCAAATCCTTGGGTGATTCCATGCATCCAATGGAGAGGCATGCATACAAAGTCTTTATGGCCACAAATTTCCCTTCTTCTCCTTGAGATTCTTGCATTA
hpt16       AGACCCGAAGTTCAAATCCTTGGGTGATTCCATGCATCCAATGGAGAGGCATGCATACAAAGTCTTTATGGCCACAAATTTCCCTTCTTCTCCTTGAGATTCTTGCATTA
hpt17       AGAGCTGAAGTTCAAATCCTTGGGTGATTCCATGCATCCAATGGAGAGGCATGCATACAAAGTCTTTATGGCCACAAATTTCCCTTCTTCTCCTTGAGATTCTTGCATTA
hpt18       AGACCTGAAGTTCAAATCCTTGGGTGATTCCATGCATCCAATGGAGAGGCATGCATACAAAGTCTTTATGGCCACAAATTTCCCTTCTTCTCCTTGAGATTCTTGCATTA
```

```
               2900      2910      2920      2930      2940      2950      2960      2970      2980      2990      3000
          ....|....|....|....|....|....|....|....|....|....|....|....|....|....|....|....|....|....|....|....|....|....|
BLZ2 gene AGTGTAACTAATCAACTCGTGAGTGGTGACAACMAATGCCACACAATAGATTGAATTCAGCTCTAAAGAAAGAAAGAAACATGGTTACATTGTGACCAATATATTAGCTT
BLZ2 CDS  --------------------------------------------------------------------------------------------------------------
hpt01     AGTGTAACTAATCAACTCGTGAGTGGTGACAACAAATGCCACACAATAGATTGAATTCAGCTCTAAAGAAAGAAAGAAACATGGTTACATTGTGACCAATATATTAGCTT
hpt02     AGTGTAACTAATCAACTCGTGAGTGGTGACAACCAATGCCACACAATAGATTGAATTCAGCTCTAAAGAAAGAAAGAAACATGGTTACATTGTGACCAATATATTAGCTT
hpt03     AGTGTAACTAATCAACTCGTGAGTGGTGACAACCAATGCCACACAATAGATTGAATTCAGCTCTAAAGAAAGAAAGAAACATGGTTACATTGTGACCAATATATTAGCTT
hpt04     AGTGTAACTAATCAACTCGTGAGTGGTGACAACCAATGCCACACAATAGATTGAATTCAGCTCTAAAGAAAGAAAGAAACATGGTTACATTGTGACCAATATATTAGCTT
hpt05     AGTGTAACTAATCAACTCGTGAGTGGTGACAACCAATGCCACACAATAGATTGAATTCAGCTCTAAAGAAAGAAAGAAACATGGTTACATTGTGACCAATATATTAGCTT
hpt06     AGTGTAACTAATCAACTCGTGAGTGGTGACAACCAATGCCACACAATAGATTGAATTCAGCTCTAAAGAAAGAAAGAAACATGGTTACATTGTGACCAATATATTAGCTT
hpt07     AGTGTAACTAATCAACTCGTGAGTGGTGACAACCAATGCCACACAATAGATTGAATTCAGCTCTAAAGAAAGAAAGAAACATGGTTACATTGTGACCAATATATTAGCTT
hpt08     AGTGTAACTAATCAACTCGTGAGTGGTGACAACCAATGCCACACAATAGATTGAATTCAGCTCTAAAGAAAGAAAGAAACATGGTTACATTGTGACCAATATATTAGCTT
hpt09     AGTGTAACTAATCAACTCGTGAGTGGTGACAACCAATGCCACACAATAGATTGAATTCAGCTCTAAAGAAAGAAAGAAACATGGTTACATTGTGACCAATATATTAGCTT
hpt10     AGTGTAACTAATCAACTCGTGAGTGGTGACAACCAATGCCACACAATAGATTGAATTCAGCTCTAAAGAAAGAAAGAAACATGGTTACATTGTGACCAATATATTAGCTT
hpt11     AGTGTAACTAATCAACTCGTGAGTGGTGACAACAAATGCCACACAATAGATTGAATTCAGCTCTAAAGAAAGAAAGAAACATGGTTACATTGTGACCAATATATTAGCTT
hpt12     AGTGTAACTAATCAACTCGTGAGTGGTGACAACCAATGCCACACAATAGATTGAATTCAGCTCTAAAGAAAGAAAGAAACATGGTTACATTGTGACCAATATATTAGCTT
hpt13     AGTGTAACTAATCAACTCGTGAGTGGTGACAACCAATGCCACACAATAGATTGAATTCAGCTCTAAAGAAAGAAAGAAACATGGTTACATTGTGACCAATATATTAGCTT
hpt14     AGTGTAACTAATCAACTCGTGAGTGGTGACAACCAATGCCACACAATAGATTGAATTCAGCTCTAAAGAAAGAAAGAAACATGGTTACATTGTGACCAATATATTAGCTT
hpt15     AGTGTAACTAATCAACTCGTGAGTGGTGACAACCAATGCCACACAATAGATTGAATTCAGCTCTAAAGAAAGAAAGAAACATGGTTACATTGTGACCAATATATTAGCTT
hpt16     AGTGTAACTAATCAACTCGTGAGTGGTGACAACAAATGCCACACAATAGATTGAATTCAGCTCTAAAGAAAGAAAGAAACATGGTTACATTGTGACCAATATATTAGCTT
hpt17     AGTGTAACTAATCAACTCGTGAGTGGTGACAACCAATGCCACACAATAGATTGAATTCAGCTCTAAAGAAAGAAAGAAACATGGTTACATTGTGACCAATATATTAGCTT
hpt18     AGTGTAACTAATCAACTCGTGAGTGGTGACAACCAATGCCACACAATAGATTGAATTCAGCTCTAAAGAAAGAAAGAAACATGGTTACATTGTGACCAATATATTAGCTT
```

```
               3010      3020      3030      3040      3050      3060      3070      3080      3090      3100      3110
          ....|....|....|....|....|....|....|....|....|....|....|....|....|....|....|....|....|....|....|....|....|....|
BLZ2 gene ATGTAGGTGTTTAACATTTAATATGGTTGCATTGCAGGTAAAGATGGYCGAGGAAACTATGAAGAGAATTACATCCACCAACAATTTCCCCCAAGCAATATCTGGCATRT
BLZ2 CDS  -------------------------------------gtaaagatggccgaggaaactatgaagagaattacatccaccaacaatttcccccaagcaatatctggcatgt
hpt01     ATGTAGGTGTTTAACATTTAATATGGTTGCATTGCAGGTAAAGATGGCCGAGGAAACTATGAAGAGAATTACATCCACCAACAATTTCCCCCAAGCAATATCTGGCATGT
hpt02     ATGTAGGTGTTTAACATTTAATATGGTTGCATTGCAGGTAAAGATGGTCGAGGAAACTATGAAGAGAATTACATCCACCAACAATTTCCCCCAAGCAATATCTGGCATAT
hpt03     ATGTAGGTGTTTAACATTTAATATGGTTGCATTGCAGGTAAAGATGGCCGAGGAAACTATGAAGAGAATTACATCCACCAACAATTTCCCCCAAGCAATATCTGGCATAT
hpt04     ATGTAGGTGTTTAACATTTAATATGGTTGCATTGCAGGTAAAGATGGCCGAGGAAACTATGAAGAGAATTACATCCACCAACAATTTCCCCCAAGCAATATCTGGCATAT
hpt05     ATGTAGGTGTTTAACATTTAATATGGTTGCATTGCAGGTAAAGATGGTCGAGGAAACTATGAAGAGAATTACATCCACCAACAATTTCCCCCAAGCAATATCTGGCATAT
hpt06     ATGTAGGTGTTTAACATTTAATATGGTTGCATTGCAGGTAAAGATGGTCGAGGAAACTATGAAGAGAATTACATCCACCAACAATTTCCCCCAAGCAATATCTGGCATAT
hpt07     ATGTAGGTGTTTAACATTTAATATGGTTGCATTGCAGGTAAAGATGGCCGAGGAAACTATGAAGAGAATTACATCCACCAACAATTTCCCGCAAGCAATATCTGGCATAT
hpt08     ATGTAGATGTTTAACATTTAATATGGTTGCATTGCAGGTAAAGATGGCCGAGGAAACTATGAAGAGAATTACATCCACCAACAATTTCCCCCAAGCAATATCTGGCATAT
hpt09     ATGTAGATGTTTAACATTTAATATGGTTGCATTGCAGGTAAAGATGGCCGAGGAAACTATGAAGAGAATTACATCCACCAACAATTTCCCCCAAGCAATATCTGGCATAT
hpt10     ATGTAGGTGTTTAACATTTAATATGGTTGCATTGCAGGTAAAGATGGCCGAGGAAACTATGAAGAGAATTACATCCACCAACAATTTCCCCCAAGCAATATCTGGCATAT
hpt11     ATGTAGGTGTTTAACATTTAATATGGTTGCATTGCAGGTAAAGATGGCCGAGGAAACTATGAAGAGAATTACATCCACCAACAATTTCCCCCAAGCAATATCTGGCATGT
hpt12     ATGTAGGTGTTTAACATTTAATATGGTTGCATTGCAGGTAAAGATGGTCGAGGAAACTATGAAGAGAATTACATCCACCAACAATTTCCCCCAAGCAATATCTGGCATAT
hpt13     ATGTAGGTGTTTAACATTTAATATGGTTGCATTGCAGGTAAAGATGGTCGAGGAAACTATGAAGAGAATTACATCCACCAACAATTTCCCCCAAGCAATATCTGGCATAT
hpt14     ATGTAGGTGTTTAACATTTAATATGGTTGCATTGCAGGTAAAGATGGTCGAGGAAACTATGAAGAGAATTACATCCACCAACAATTTCCCCCAAGCAATATCTGGCATAT
hpt15     ATGTAGGTGTTTAACATTTAATATGGTTGCATTGCAGGTAAAGATGGTCGAGGAAACTATGAAGAGAATTACATCCACCAACAATTTCCCCCAAGCAATATCTGGCATAT
hpt16     ATGTAGGTGTTTAACATTTAATATGGTTGCATTGCAGGTAAAGATGGCCGAGGAAACTATGAAGAGAATTACATCCACCAACAATTTCCCCCAAGCAATATCTGGCATGT
hpt17     ATGTAGGTGTTTAACATTTAATATGGTTGCATTGCAGGTAAAGATGGTCGAGGAAACTATGAAGAGAATTACATCCACCAACAATTTCCCCCAAGCAATATCTGGCATAT
hpt18     ATGTAGGTGTTTAACATTTAATATGGTTGCATTGCAGGTAAAGATGGCCGAGGAAACTATGAAGAGAATTACATCCACCAACAATTTCCCGCAAGCAATATCTGGCATAT
```

```
                 3120      3130      3140      3150      3160      3170      3180      3190      3200      3210      3220
            ....|....|....|....|....|....|....|....|....|....|....|....|....|....|....|....|....|....|....|....|....|....|
BLZ2 gene   CATCTCTCAGGACCCATTTCAGTGGTTCCCAATTGGATGGYATCTTTGATACTACATTGCCAACCCAAAACATGTCACTTAACCATTTTTCCACTACAGCAACAAATTTT
BLZ2 CDS    catctctcaggacccatttcagtggttcccaattggatggcatctttgatactacattgccaacccaaaacatgtcacttaaccatttttccactacagcaacaaatttt
hpt01       CATCTCTCAGGACCCATTTCAGTGGTTCCCAATTGGATGGCATCTTTGATACTACATTGCCAACCCAAAACATGTCACTTAACCATTTTTCCACTACAGCAACAAATTTT
hpt02       CATCTCTCAGGACCCATTTCAGTGGTTCCCAATTGGATGGTATCTTTGATACTACATTGCCAACCCAAAACATGTCACTTAACCATTTTTCCACTACAGCAACAAATTTT
hpt03       CATCTCTCAGGACCCATTTCAGTGGTTCCCAATTGGATGGTATCTTTGATACTACATTGCCAACCCAAAACATGTCAATTAACCATATTTCCACTACAGCAACAAATTTT
hpt04       CATCTCTCAGGACCCATTTCAGTGGTTCCCAATTGGATGGTATCTTTGATACTACATTGCCAACCCAAAACATGTCACTTAACCATTTTTCCACTACAGCAACAAATTTT
hpt05       CATCTCTCAGGACCCATTTCAGTGGTTCCCAATTGGATGGTATCTTTGATACTACATTGCCAACCCAAAACATGTCACTTAACCATTTTTCCACTACAGCAACAAATTTT
hpt06       CATCTCTCAGGACCCATTTCAGTGGTTCCCAATTGGATGGTATCTTTGATACTACATTGCCAACCCAAAACATGTCACTTAACCATTTTTCCACTACAGCAACAAATTTT
hpt07       CATCTCTCAGGACCCATTTCAGTGGTTCCCAATTGGATGGTATCTTTGATACTACATTGCCAACCCAAAACATGTCACTTAACCATTTTTCCACTACAGCAACAAATTTT
hpt08       CATCTCTCAGGACCCATTTCAGTGGTTCCCAATTGGATGGTATCTTTGATACTACATTGCCAACCCAAAACATGTCACTTAACCATTTTTCCACTACAGCAACAAATTTT
hpt09       CATCTCTCAGGACCCATTTCAGTGGTTCCCAATTGGATGGTATCTTTGATACTACATTGCCAACCCAAAACATGTCACTTAACCATTTTTCCACTACAGCAACAAATTTT
hpt10       CATCTCTCAGGACCCATTTCAGTGGTTCCCAATTGGATGGTATCTTTGATACTACATTGCCAACCCAAAACATGTCAATTAACCATATTTCCACTACAGCAACAAATTTT
hpt11       CATCTCTCAGGACCCATTTCAGTGGTTCCCAATTGGATGGCATCTTTGATACTACATTGCCAACCCAAAACATGTCACTTAACCATTTTTCCACTACAGCAACAAATTTT
hpt12       CATCTCTCAGGACCCATTTCAGTGGTTCCCAATTGGATGGTATCTTTGATACTACATTGCCAACCCAAAACATGTCACTTAACCATTTTTCCACTACAGCAACAAATTTT
hpt13       CATCTCTCAGGACCCATTTCAGTGGTTCCCAATTGGATGGTATCTTTGATACTACATTGCCAACCCAAAACATGTCACTTAACCATTTTTCCACTACAGCAACAAATTTT
hpt14       CATCTCTCAGGACCCATTTCAGTGGTTCCCAATTGGATGGTATCTTTGATACTACATTGCCAACCCAAAACATGTCACTTAACCATTTTTCCACTACAGCAACAAATTTT
hpt15       CATCTCTCAGGACCCATTTCAGTGGTTCCCAATTGGATGGTATCTTTGATACTACATTGCCAACCCAAAACATGTCACTTAACCATTTTTCCACTACAGCAACAAATTTT
hpt16       CATCTCTCAGGACCCATTTCAGTGGTTCCCAATTGGATGGCATCTTTGATAGTACATTGCCAACCCAAAACATGTCACTTAACCATTTTTCCACTACAGCAACAAATTTT
hpt17       CATCTCTTAGGACCCATTTCAGTGGTTCCCAATTGGATGGTATCTTTGATACTACATTGCCAACCCAAAACATGTCACTTAACCATTTTTCCACTACAGCAACAAATTTT
hpt18       CATCTCTCAGGACCCATTTCAGTGGTTCCCAATTGGATGGTATCTTTGATACTACATTGCCAACCCAAAACATGTCACTTAACCATTTTTCCACTACAGCAACAAATTTT


                 3230      3240      3250      3260      3270      3280      3290      3300      3310      3320      3330
            ....|....|....|....|....|....|....|....|....|....|....|....|....|....|....|....|....|....|....|....|....|....|
BLZ2 gene   GATGTGAGCAGCAACTACATCCCCGAGCTAGCTCCGGCATACCAGATCCATGATCAAATATCTTCGCTACATACGCAACCTATGCCATGCTTGGATCATCACCCACGGAG
BLZ2 CDS    gatgtgagcagcaactacatccccgagctagctccggcataccagatccatgatcaaatatcttcgctacatacgcaacctatgccatgcttggatcatcacccacggag
hpt01       GATGTGAGCAGCAACTACATCCCCGAGCTAGCTCCGGCATACCAGATCCATGATCAAATATCTTCGCTACATACGCAACCTATGCCATGCTTGGATCATCA
hpt02       GATGTGAGCAGCAACTACATCCCCGAGCTAGCTCCGGCATACCAGATCCATGATCAAATATCTTCGCTACATACGCAACCTATGCCATGCTTGGATCATCA
hpt03       GATGTGAGCAGCAACTACATCCCCGAGCTAGCTCCGGCATACCAGATCCATGATCAAATATCTTCGCTACATACGCAACCTATGCCATGCTTGGATCATCA
hpt04       GATGTGAGCAGCAACTACATCCCCGAGCTAGCTCCGGCATACCAGATCCATGATCAAATATCTTCGCTACATACGCAACCTATGCCATGATTGGATCATCA
hpt05       GATGTGAGCAGCAACTACATCCCCGAGCTAGCTCCGGCATACCAGATCCATGATCAAATATCTTCGCTACATACGCAACCTATGCCATGCTTGGATCATCA
hpt06       GATGTGAGCAGCAACTACATCCCCAAGCTAGCTCCGGCATACCAGATCCATGATCAAATATCTTCGCTACATACGCAACCTATGCCATGCTTGGATCATCA
hpt07       GATGTGAGCAGCAACTACATCCCCGAGCTAGCTCCGGCATACCAGATCCATGATCAAATATCTTCGCTACATACGCAACCTATGCCATGCTTGGATCATCA
hpt08       GATGTGAGCAGCAACTACATCCCCGAGCTAGCTCCGGCATACCAGATCCATGATCAAATATCTTCGCTACATACGCAACCTATGCCATGCTTGGATCGTCA
hpt09       GATGTGAGCAGCAACTACATCCCCGAGCTAGCTCCGGCATACCAGATCCATGATCAAATATCTTCGCTACATACGCAACCTATGCCATGCTTGGATCGTCA
hpt10       GATGTGAGCAGCAACTACATCCCCGAGCTAGCTCCGGCATACCAGATCCATGATCAAATATCTTCGCTACATACGCAACCTATGCCATGCTTGGATCATCA
hpt11       GATGTGAGCAGCAACTACATCCCCGAGCTAGCTCCGGCATACCAGATCCATGATCAAATATCTTCGCTACATACGCAACCTATGCCATGCTTGGATCATCA
hpt12       GATGTGAGCAGCAACTACATCCCCAAGCTAGCTCCGGCATACCAGATCCATGATCAAATATCTTCGCTACATACGCAACCTATGCCATGCTTGGATCATCA
hpt13       GATGTGAGCAGCAACTACATCCCCGAGCTAGCTCCGGCATACCAGATCCATGATCAAATATCTTCGCTACATACGCAACCTATGCCATGCTTGGATCATCA
hpt14       GATGTGAGCAGCAACTACATCCCCAAGCTAGCTCCGGCATACCAGATCCATGATCAAATATCTTCGCTACATACGCAACCTATGCCATGCTTGGATCATCA
hpt15       GATGTGAGCAGCAACTACATCCCCGAGCTAGCTCCGGCATACCAGATCCATGATCAAATATCTTCGCTACATACGCAACCTATGCCATGCTTGGATCATCA
hpt16       GATGTGAGCAGCAACTACATCCCCGAGCTAGCTCCGGCATACCAGATCCATGATCAAATATCTTCGCTACATACGCAACCTATGCCATGCTTGGATCATCA
hpt17       GATGTGAGCAGCAACTACATCCCCGAGCTAGCTCCGGCATACCAGATCCATGATCAAATATCTTCGCTACATACGCAACCTATGCCATGCTTGGATCATCA
hpt18       GATGTGAGCAGCAACTACATCCCCGAGCTAGCTCCGGCATACCAGATCCATGATCAAATATCTTCGCTACATACGCAACCTATGCCATGCTTGGATCATCA
```

```
                     3340      3350      3360      3370      3380      3390      3400      3410      3420      3430      3440
           ....|....|....|....|....|....|....|....|....|....|....|....|....|....|....|....|....|....|....|....|....|....|
BLZ2 gene  GATGCCCTTTGGTATTCCAAGTACATTGGTGCCTACCCCACAACGGGAATCCACTACATTGGATTCAAATGAAATAGGCAACATGGTGATGCAGTAGGAATTATGTGAGA
BLZ2 CDS   gatgccctttggtattccaagtacattggtgcctaccccacaacgggaatccactacattggattcaaatgaaataggcaacatggtgatgcagtag
hpt01
hpt02
hpt03
hpt04
hpt05
hpt06
hpt07
hpt08
hpt09
hpt10
hpt11
hpt12
hpt13
hpt14
hpt15
hpt16
hpt17
hpt18
```

```
                     3450      3460      3470      3480      3490      3500      3510      3520      3530      3540      3550
           ....|....|....|....|....|....|....|....|....|....|....|....|....|....|....|....|....|....|....|....|....|....|
BLZ2 gene  GACCTGAGCCGGAATTATTATTTTAAAATAAATATTGTCGTTGTTCTTGGTGGCCATTGGGGGTATTTGTAATGGTTTCTCACCTTGTTAAGCTCAGACTTGTTTAAGGA
BLZ2 CDS
hpt01
hpt02
hpt03
hpt04
hpt05
hpt06
hpt07
hpt08
hpt09
hpt10
hpt11
hpt12
hpt13
hpt14
hpt15
hpt16
hpt17
hpt18
```

```
                3560      3570      3580      3590      3600      3610      3620      3630      3640      3650
            ....|....|....|....|....|....|....|....|....|....|....|....|....|....|....|....|....|....|....|....|....
BLZ2 gene   ATATCTATCATTGTTGTTTCAGAAAGAACCTATACATTGTTGGTATCCCATAACTAATAAAATATATCAAAGGTTATTGGACAATAAAGTCATGTGGTTGAGTT
BLZ2 CDS
hpt01
hpt02
hpt03
hpt04
hpt05
hpt06
hpt07
hpt08
hpt09
hpt10
hpt11
hpt12
hpt13
hpt14
hpt15
hpt16
hpt17
hpt18
```

***BPBF***

```
              -430      -420      -410      -400      -390      -380      -370      -360      -350      -340      -330
              .|....|....|....|....|....|....|....|....|....|....|....|....|....|....|....|....|....|....|....|....|....|...
BPBF mRNA     --------------------------------------------------------------------------------------------------------------
BPBF_CDS      --------------------------------------------------------------------------------------------------------------
hpt01         GCATGCCGGATTCACTACTTTTGTACGCAAGGTTTTATTTATTTTTTGTCTCGATTTACAAAAAGTTGGATCTGATTTTGTAGTTCTTTTTTATCCATGCCGGATTCACT
hpt02         GCATGCCGGATTCACTACTTTTGTACGCAAGGTTTTATTTATTTTTTGTCTCGATTTACAAAAGGTTGGATCTGATTTTGTAGTTCTTTTTTATCCATGCCGGATTCACT
hpt03         GCATGCCGGATTCACTACTTTTGTACGCAAGGTTTTATTTATTTTTTGTCTCGATTTACAAAAAGTTGGATCTGATTTTGTAGTTCTTTTTTATCCATGCCGGATTCACT
hpt04         GCATGCCGGATTCACTACTTTTGTACGCAAGGTTTTATTTATTTTTTGTCTCGATTTACAAAAGGTTGGATCTGATTTTGTAGTTCTTTTTTATCCATGCCGGATTCACT
hpt05         GCATGCCGGATTCACTACTTTTGTACGCAAGGTTTTATTTATTTTTTGTCTCGATTTACAAAAGGTTGGATCTGATTTTGTAGTTCTTTTTTATCCATGCCGGATTCACT
hpt06         GCATGCCGGATTCACTACTTTTGTACGCAAGGTTTTATTTATTTTTTGTCTCGATTTACAAAAGGTTGGATCTGATTTTGTAGTTCTTTTTTATCCATGCCGGATTCACT
hpt07         GCATGCCGGATTCACTACTTTTGTACACAAGGTTTTATTTATTTTTTGTCTCGATTTACAAAAAGTTGGATCTGATTTTGTAGTTCTTTTTTATCCATGCCGGATTCACT
hpt08         GCATGCCGGATTCACTACTTTTGTACGCAAGGTTTTATTTATTTTTTGTCTCGATTTACAAAAGGTTGGATCTGATTTTGTAGTTCTTTTTTATCCATGCCGGATTCACT
hpt09         GCATGCCGGATTCACTACTTTTGTACGCAAGGTTTTATTTATTTTTTGTCTCGATTTACAAAAAGTTGGATCTGATTTTGTAGTTCTTTTTTATCCATGCCGGATTCACT
hpt10         GCATGCCGGATTCACTACTTTTGTACACAAGGTTTTATTTATTTTTTGTCTCGATTTACAAAAAGTTGGATCTGATTTTGTAGTTCTTTTTTATCCATGCCGGATTCACT
hpt11         GCATGCCGGATTCACTACTTTTGTACGCAAGGTTTTATTTATTTTTTGTCTCGATTTACAAAAAGTTGGATCTGATTTTGTAGTTCTTTTTTATCCATGCCGGATTCACT
hpt12         GCATGCCGGATTCACTACTTTTGTACACAAGGTTTTATTTATTTTTTGTCTCGATTTACAAAAAGTTGGATCTGATTTTGTAGTTCTTTTTTATCCATGCCGGATTCACT
hpt13         GCATGCCGGATTCACTACTTTTGTACGCAAGGTTTTATTTATTTTTTGTCTCGATTTACAAAAAGTTGGATCTGATTTTGTAGTTCTTTTTTATCCATGCCGGATTCACT
hpt14         GCATGCCGGATTCACTACTTTTGTACGCAAGGTTTTATTTATTTTTTGTCTCGATTTACAAAAGGTTGGATCTGATTTTGTAGTTCTTTTTTATCCATGCCGGATTCACT
hpt15         GCATGCCGGATTCACTACTTTTGTACGCAAGGTTTTATTTATTTTTTGTCTCGATTTACAAAAGGTTGGATCTGATTTTGTAGTTCTTTTTTATCCATGCCGGATTCACT
hpt16         GCATGCCGGATTCACTACTTTTGTACGCAAGGTTTTATTTATTTTTTGTCTCGATTTACAAAAAGTTGGATCTGATTTTGTAGTTCTTTTTTATCCATGCCGGATTCACT
hpt17         GCATGCCGGATTCACTACTTTTGTACGCAAGGTTTTATTTATTTTTTGTCTCGATTTACAAAAGGTTGGATCTGATTTTGTAGTTCTTTTTTATCCATGCCGGATTCACT
hpt18         GCATGCCGGATTCACTACTTTTGTACGCAAGGTTTTATTTATTTTTTGTCTCGATTTACAAAANGTTGGATCTGATTTTGTAGTTCTTTTTTATCCATGCCGGATTCACT
hpt19         GCATGCCGGATTCACTACTTTTGTACGCAAGGTTTTATTTATTTTTTGTCTCGATTTACAAAAGGTTGGATCTGATTTTGTAGTTCTTTTTTATCCATGCCGGATTCACT
hpt20         GCATGCCGGATTCACTACTTTTGTACGCAAGGTTTTATTTATTTTTTGTCTCGATTTACAAAAGGTTGGATCTGATTTTGTAGTTCTTTTTTATCCATGCCGGATTCACT
hpt21         GCATGCCGGATTCACTACTTTTGTACGCAAGGTTTTATTTATTTTTTGTCTCGATTTACAAAAAGTTGGATCTGATTTTGTAGTTCTTTTTTATCCATGCCGGATTCACT

              -320      -310      -300      -290      -280      -270      -260      -250      -240      -230      -220
              .|....|....|....|....|....|....|....|....|....|....|....|....|....|....|....|....|....|....|....|....|...
BPBF mRNA     --------------------------------------------------------------------------------------------------------------
BPBF_CDS      --------------------------------------------------------------------------------------------------------------
hpt01         ACTTTGCATCCAAGATTTAATTTTATTTTTATCTCGAATTCTTACATGTCTGGTTAGCTAATTAGGAGTGCTTGGGATTAGGCTTTCAATGAATACTAATACCAGGGAGC
hpt02         ACTTTGTATCCAAGATTTTATTTTATTTTTATCTCGAATTCTTACATGTCTGGTTAGCTAATTAGGAGTGCTTGGGATTAGGCTTTCAATGAATACTAATACCAGGCAGC
hpt03         ACTTTGCATCCAAGATTTAATTTTATTTTTATCTCGAATTCTTACATGTCTGGTTAGCTAATTAGGAGTGCTTGGGATTAGGCTTTCAATGAATACTAATACCAGGGAGC
hpt04         ACTTTGTATCCAAGATTTTATTTTATTTTTATCTCGAATTCTTACATGTCTGGTTAGCTAATTAGGAGTGCTTGGGATTAGGCTTTCAATGAATACTAATACCAGGCAGC
hpt05         ACTTTGTATCCAAGATTTTATTTTATTTTTATCTCGAATTCTTACATGTCTGGTTAGCTAATTAGGAGTGCTTGGGATTAGGCTTTCAATGAATACTAATACCAGGCAGC
hpt06         ACTTTGTATCCAAGATTTTATTTTATTTTTATCTCGAATTCTTACATGTCTGGTTAGCTAATTAGGAGTGCTTGGGATTAGGCTTTCAATGAATACTAATACCAGGCAGC
hpt07         ACTTTGCATCCAAGATTTAATTTTATTTTTATCTCGAATTCTTACATGTCTGGTTAGCTAATTAGGAGTGCTTGGGATTAGGCTTTCAATGAATACTAATACCAGGGAGC
hpt08         ACTTTGTATCCAAGATTTTATTTTATTTTTATCTCGAATTCTTACATGTCTGGTTAGCTAATTAGGAGTGCTTGGGATTAGGCTTTCAATGAATACTAATACCAGGCAGC
hpt09         ACTTTGCATCCAAGATTTAATTTTATTTTTATCTCGAATTCTTACATGTCTGGTTAGCTAATTAGGAGTGCTTGGGATTAGGCTTTCAATGAATACTAATACCAGGGAGC
hpt10         ACTTTGCATCCAAGATTTAATTTTATTTTTATCTCGAATTCTTACATGTCTGGTTAGCTAATTAGGAGTGCTTGGGATTAGGCTTTCAATGAATACTAATACCAGGGAGC
hpt11         ACTTTGCATCCAAGATTTAATTTTATTTTTATCTCGAATTCTTACATGTCTGGTTAGCTAATTAGGAGTGCTTGGGATTAGGCTTTCAATGAATACTAATACCAGGGAGC
hpt12         ACTTTGCATCCAAGATTTAATTCTATTTTTATCTCGAATTCTTACATGTCTGGTTAGCTAATTAGGAGTGCTTGGGATTAGGCTTTCAATGAATACTAATACCAGGGAGC
hpt13         ACTTTGTATCCAAGATTTTATTTTATTTTTATCTCGAATTCTTACATGTCTGGTTAGCTAATTAGGAGTGCTTGGGATTAGGCTTTCAATGAATACTAATACCAGGCAGC
hpt14         ACTTTGCATCCAAGATTTNATTTTATTTTTATCTCGAATTCTTACATGTCTGGTTAGCTAATTAGGAGTGCTTGGGATTAGGCTTTCAATGAATACTAATACCAGGCAGC
hpt15         ACTTTGTATCCAAGATTTTATTTTATTTTTATCTCGAATTCTTACATGTCTGGTTAGCTAATTAGGAGTGCTTGGGATTAGGCTTTCAATGAATACTAATACCAGGCAGC
hpt16         ACTTTGCATCCAAGATTTAATTTTATTTTTATCTCGAATTCTTACATGTCTGGTTAGCTAATTAGGAGTGCTTGGGATTAGGCTTTCAATGAATACTAATACCAGGGAGC
hpt17         ACTTTGTATCCAAGATTTTATTTTATTTTTATCTCGAATTCTTACATGTCTGGTTAGCTAATTAGGAGTGCTTGGGATTAGGCTTTCAATGAATACTAATACCAGGCAGC
hpt18         ACTTTGCATCCAAGATTTAATTTTATTTTTATCTCGAATTCTTACATGTCTGGTTAGCTAATTAGGAGTGCTTGGGATTAGGCTTTCAATGAATACTAATACCAGGNAGC
hpt19         ACTTTGTATCCAAGATTTAATTTTATTTTTATCTCGAATTCTTACATGTCTGGTTAGCTAATTAGGAGTGCTTGGGATTAGGCTTTCAATGAATACTAATACCAGGCAGC
hpt20         ACTTTGCATCCAAGATTTTATTTTATTTTTATCTCGAATTCTTACATGTCTGGTTAGCTAATTAGGAGTGCTTGGGATTAGGCTTTCAATGAATACTAATACCAGGCAGC
hpt21         ACTTTGTATCCAAGATTTTATTTTATTTTTATCTCGAATTCTTACATGTCTGGTTAGCTAATTAGGAGTGCTTGGGATTAGGCTTTCAATGAATACTAATACCAGGGAGC
```

```
                -210      -200      -190      -180      -170      -160      -150      -140      -130      -120      -110
                .|....|....|....|....|....|....|....|....|....|....|....|....|....|....|....|....|....|....|....|....|....|...
BPBF mRNA       --------------------------------------------------------------------------------------------------------------
BPBF_CDS        --------------------------------------------------------------------------------------------------------------
hpt01           CTGGGAAGAGACAACTTATTTTTTAAGGCACTCAAAAGAACTAACGCTTAGATTTGTAGCGGATTAATTAAATCTATTGTATGGATCTTATAATTTGAATTATGCACCCA
hpt02           CTAGGAAGAGACAACTTATTTTTTAAGGCACTCAAAAGAACTAACACTTAGATTTGTAGCGGATTAATTAAATCTATTGTATGGATCTTATAATTTGAATTATGCACCCA
hpt03           CTGGGAAGAGACAACTTATTTTTTAAGGCACTCAAAAGAACTAACGCTTAGATTTGTAGCGGATTAATTAAATCTATTGTATGGATCTTATAATTTGAATTATGCACCCA
hpt04           CTAGGAAGAGACAACTTATTTTTTAAGGCACTCAAAAGAACTAACACTTAGATTTGTAGCGGATTAATTAAATCTATTGTATGGATCTTATAATTTGAATTATGCACCCA
hpt05           CTAGGAAGAGACAACTTATTTTTTAAGGCACTCAAAAGAACTAACACTTAGATTTGTAGCGGATTAATTAAATCTATTGTATGGATCTTATAATTTGAATTATGCACCCA
hpt06           CTAGGAAGAGACAACTTATTTTTTAAGGCACTCAAAAGAACTAACACTTAGATTTGTAGCGGATTAATTAAATCTATTGTATGGATCTTATAATTTGAATTATGCACCCA
hpt07           CTGGGAAGAGACAACTTATTTTTTAAGGCACTCAAAAGAACTAACGCTTAGATTTGTAGCGGATTAATTAAATCTATTGTATGGATCTTATAATTTGAATTATGCACCCA
hpt08           CTAGGAAGAGACAACTTATTTTTTAAGGCACTCAAAAGAACTAACACTTAGATTTGTAGCGGATTAATTAAATCTATTGTATGGATCTTATAATTTGAATTATGCACCCA
hpt09           CTGGGAAGAGACAACTTATTTTTTAAGGCACTCAAAAGAACTAACGCTTAGATTTGTAGCGGATTAATTAAATCTATTGTATGGATCTTATAATTTGAATTATGCACCCA
hpt10           CTGGGAAGAGACAACTTATTTTTTAAGGCACTCAAAAGAACTAACGCTTAGATTTGTAGCGGATTAATTAAATCTATTGTATGGATCTTATAATTTGAATTATGCACCCA
hpt11           CTGGGAAGAGACAACTTATTTTTTAAGGCACTCAAAAGAACTAACGCTTAGATTTGTAGCGGATTAATTAAATCTATTGTATGGATCTTATAATTTGAATTATGCACCCA
hpt12           CTGGGAAGAGACAACTTATTTTTTAAGGCACTCAAAAGAACTAACGCTTAGATTTGTAGCGGATTAATTAAATCTATTGTATGGATCTTATAATTTGAATTATGCACCCA
hpt13           CTAGGAAGAGACAACTTATTTTTTAAGGCACTCAAAAGAACTAACACTTAGATTTGTAGCGGATTAATTAAATCTATTGTATGGATCTTATAATTTGAATTATGCACCCA
hpt14           CTNGGAAGAGACAACTTATTTTTTAAGGCACTCAAAAGAACTAACACTTAGATTTGTAGCGGATTAATTAAATCTATTGTATGGATCTTATAATTTGAATTATGCACCCA
hpt15           CTAGGAAGAGACAACTTATTTTTTAAGGCACTCAAAAGAACTAACACTTAGATTTGTAGCGGATTAATTAAATCTATTGTATGGATCTTATAATTTGAATTATGCACCCA
hpt16           CTGGGAAGAGACAACTTATTTTTTAAGGCACTCAAAAGAACTAACGCTTAGATTTGTAGCGGATTAATTAAATCTATTGTATGGATCTTATAATTTGAATTATGCACCCA
hpt17           CTAGGAAGAGACAACTTATTTTTTAAGGCACTCAAAAGAACTAACACTTAGATTTGTAGCGGATTAATTAAATCTATTGTATGGATCTTATAATTTGAATTATGCACCCA
hpt18           CTGGGAAGAGACAACTTATTTTTTAAGGCACTCAAAAGAACTAACGCTTAGATTTGTAGCGGATTAATTAAATCTATTGTATGGATCTTATAATTTGAATTATGCACCCA
hpt19           CTAGGAAGAGACAACTTATTTTTTAAGGCACTCAAAAGAACTAACACTTAGATTTGTAGCGGATTAATTAAATCTATTGTATGGATCTTATAATTTGAATTATGCACCCA
hpt20           CTAGGAAGAGACAACTTATTTTTTAAGGCACTCAAAAGAACTAACACTTAGATTTGTAGCGGATTAATTAAATCTATTGTATGGATCTTATAATTTGAATTATGCACCCA
hpt21           CTAGGAAGAGACAACTTATTTTTTAAGGCACTCAAAAGAACTAACACTTAGATTTGTAGCGGATTAATTAAATCTATTGTATGGATCTTATAATTTGAATTATGCACCCA
```

```
                -100      -90       -80       -70       -60       -50       -40       -30       -20       -10       -1
                .|....|....|....|....|....|....|....|....|....|....|....|....|....|....|....|....|....|....|....|...|1...|....
BPBF mRNA       -----------------------------------------------------------------------------------------------------atggaggaa
BPBF_CDS        -----------------------------------------------------------------------------------------------------ATGGAGGAA
hpt01           CCATGACATTTTGAGTTAACCAATGATTCGACATGTCTGACACATTAGGATTAGCAACTTAACTTAATTTAATTTGATATGGTTGTATACATTTGTGGCAGATGGAGGAA
hpt02           TCATGACATTTTGAGTTAACCAATGATTCGACATGTCTGACACATTAGGATTAGCAACTTAACTTAATTTAATTCGATATGGTTGTATACATTTGTGGTAGATGGAGGAA
hpt03           CCATGACATTTTGAGTTAACCAATGATTCGACATGTCTGACACATTAGGATTAGCAACTTAACTTAATTTAATTTGATATGGTTGTATACATTTGTGGCAGATGGAGGAA
hpt04           TCATGACATTTTGAGTTAACCAATGATTCGACATGTCTGACACATTAGGATTAGCAACTTAACTTAATTTAATTCGATATGGTTGTATACATTTGTGGTAGATGGAGGAA
hpt05           TCATGACATTTTGAGTTAACCAATGATTCGACATGTCTGACACATTAGGATTAGCAACTTAACTTAATTTAATTCGATATGGTTGTATACATTTGTGGTAGATGGAGGAA
hpt06           TCATGACATTTTGAGTTAACCAATGATTCGACATGTCTGACACATTAGGATTAGCAACTTAACTTAATTTAATTCGATATGGTTGTATACATTTGTGGTAGATGGAGGAA
hpt07           CCATGACATTTTGAGTTAACCAATGATTCGACATGTCTGACACATTAGGATTAGCAACTTAACTTAATTTAATTTGATATGGTTGTATACATTTGTGGCAGATGGAGGAA
hpt08           TCATGACATTTTGAGTTAACCAATGATTCGACATGTCTGACACATTAGGATTAGCAACTTAACTTAATTTAATTCGATATGGTTGTATACATTTGTGGTAGATGGAGGAA
hpt09           CCATGACATTTTGAGTTAACCAATGATTCGACATGTCTGACACATTAGGATTAGCAACTTAACTTAATTTAATTTGATGTGGTTGTATACATTTGTGGCAGATGGAGGAA
hpt10           CCATGACATTTTGAGTTAACCAATGATTCGACATGTCTGACACATTAGGATTAGCAACTTAACTTAATTTAATTTGATATGGTTGTATACATTTGTGGCAGATGGAGGAA
hpt11           CCATGACATTTTGAGTTAACCAATGATTCGACATGTCTGACACATTAGGATTAGCAACTTAACTTAATTTAATTTGATATGGTTGTATACATTTGTGACAGATGGAGGAA
hpt12           CCATGACATTTTGAGTTAACCAATGATTCGACATGTCTGACACATTAGGATTAGCAACTTAACTTAATTTAATTTGATATGGTTGTATACATTTGTGGCAGATGGAGGAA
hpt13           TCATGACATTTTGAGTTAACCAATGATTCGACATGTCTGACACATTAGGATTAGCAACTTAACTTAATTTAATTCGATATGGTTGTATACATTTGTGGTAGATGGAGGAA
hpt14           NCATGACATTTTGAGTTAACCAATGATTCGACATGTCTGACACATTAGGATTAGCAACTTAACTTAATTTAATTCGATATGGTTGTATACATTTGTGGNAGATGGAGGAA
hpt15           TCATGACATTTTGAGTTAACCAATGATTCGACATGTCTGACACATTAGGATTAGCAACTTAACTTAATTTAATTCGATATGGTTGTATACATTTGTGGTAGATGGAGGAA
hpt16           CCATGACATTTTGAGTTAACCAATGATTCGACATGTCTGACACATTAGGATTAGCAACTTAACTTAATTTAATTTGATATGGTTGTATACATTTGTGGCAGATGGAGGAA
hpt17           TCATGACATTTTGAGTTAACCAATGATTCGACATGTCTGACACATTAGGATTAGCAACTTAACTTAATTTAATTCGATATGGTTGTATACATTTGTGGTAGATGGAGGAA
hpt18           CCATGACATTTTGAGTTAACCAATGATTCGACATGTCTGACACATTAGGATTAGCAACTTAACTTAATTTAATTTGATATGGTTGTATACATTTGTGGCAGATGGAGGAA
hpt19           TCATGACATTTTGAGTTAACCAATGATTCGACATGTCTGACACATTAGGATTAGCAACTTAACTTAATTTAATTCGATATGGTTGTATACATTTGTGGCAGATGGAGGAA
hpt20           TCATGACATTTTGAGTTAACCAATGATTCGACATGTCTGACACATTAGGATTAGCAACTTAACTTAATTTAATTCGATATGGTTGTATACATTTGTGGCAGATGGAGGAA
hpt21           TCATGACATTTTGAGTTAACCAATGATTCGACATGTCTGACACATTAGGATTAGCAACTTAACTTAATTTAATTCGATATGGTTGTATACATTTGTGGCAGATGGAGGAA
```

```
           10        20        30        40        50        60        70        80        90        100       110
           |....|....|....|....|....|....|....|....|....|....|....|....|....|....|....|....|....|....|....|....|....|....
BPBF mRNA  gtgttttcgtccaactccaagagcaaggccggtcagatggcgggagaggcggcggcggcggccgagaagaagtctcggccgaagccagagcagaaggtggagtgccctcg
BPBF_CDS   GTGTTTTCGTCCAACTCCAAGAGCAAGGCCGGTCAGATGGCGGGAGAGGCGGCGGCGGCGGCCGAGAAGAAGTCTCGGCCGAAGCCAGAGCAGAAGGTGGAGTGCCCTCG
hpt01      GTGTTTTCGTCCAACTCCAAGAGCAAGGCCGGTCAGATGGCGGGAGAGGCGGCGGCGGCGGCCGAGAAGAA--------------------------------------
hpt02      GTGTTTTCGTCCAACTCCAAGAGCAAGGCCGGTCAGATGGCGGGAGAGGCGGTGGCGGCGGCCGAGAAGAA--------------------------------------
hpt03      GTGTTTTCGTCCAACTCCAAGAGCAAGGCCGGTCAGATGGCGGGAGAGGCGGCGGCGGCGGCCGAGAAGAA--------------------------------------
hpt04      GTGTTTTCGTCCAACTCCAAGAGCAAGGCCGGTCAGATGGCGGGAGAGGCGGTGGCGGCGGCCGAGAAGAA--------------------------------------
hpt05      GTGTTTTCGTCCAACTCCAAGAGCAAGGCCGGTCAGATGGCGGGAGAGGCGGTGGCGGCGGCCGAGAAGAA--------------------------------------
hpt06      GTGTTTTCGTCCAACTCCAAGAGCAAGGCCGGTCAGATGGCGGGAGAGGCGGTGGCGGCGGCCGAGAAGAA--------------------------------------
hpt07      GTGTTTTCGTCCAACTCCAAGAGCAAGGCCGGTCAGATGGCGGGAGAGGCGGCGGCGGCGGCCGAGAAGAA--------------------------------------
hpt08      GTGTTTTCGTCCAACTCCAAGAGCAAGGCCGGTCAGATGGCGGGAGAGGCGGTGGCGGCGGCCGAGAAGAA--------------------------------------
hpt09      GTGTTTTCGTCCAACTCCAAGAGCAAGGCCGGTCAGATGGCGGGAGAGGCGGCGGCGGCGGCCGAGAAGAA--------------------------------------
hpt10      GTGTTTTCGTCCAACTCCAAGAGCAAGGCCGGTCAGATGGCGGGAGAGGCGGCGGCGGCGGCCGAGAAGAA--------------------------------------
hpt11      GTGTTTTCGTCCAACTCCAAGAGCAAGGCCGGTCAGATGGCGGGAGAGGCGGCGGCGGCGGCCGAGAAGAA--------------------------------------
hpt12      GTGTTTTCGTCCAACTCCAAGAGCAAGGCCGGTCAGATGGCGGGAGAGGCGGCGGCGGCGGCCGAGAAGAA--------------------------------------
hpt13      GTGTTTTCGTCCAACTCCAAGAGCAAGGCCGGTCAGATGGCGGGAGAGGCGGTGGCGGCGGCCGAGAAGAA--------------------------------------
hpt14      GTGTTTTCGTCCAACTCCAAGAGCAAGGCCGGTCAGATGGCGGGAGAGGCGGNGGCGGCGGCCGAGAAGAA--------------------------------------
hpt15      GTGTTTTCGTCCAACTCCAAGAGCAAGGCCGGTCAGATGGCGGGAGAGGCGGTGGCGGCGGCCGAGAAGAA--------------------------------------
hpt16      GTGTTTTCGTCCAACTCCAAGAGCAAGGCCGGTCAGATGGCGGGAGAGGCGGCGGCGGCGGCCGAGAAGAA--------------------------------------
hpt17      GTGTTTTCGTCCAACTCCAAGAGCAAGGCCGGTCAGATGGCGGGAGAGGCGGTGGCGGCGGCCGAGAAGAA--------------------------------------
hpt18      GTGTTTTCGTCCAACTCCAAGAGCAAGGCCGGTCAGATGGCGGGAGAGGCGGCGGCGGCGGCCGAGAAGAA--------------------------------------
hpt19      GTGTTTTCGTCCAACTCCAAGAGCAAGGCCGGTCAGATGGCGGGAGAGGCGGCGGCGGCGGCCGAGAAGAA--------------------------------------
hpt20      GTGTTTTCGTCCAACTCCAAGAGCAAGGCCGGTCAGATGGCGGGAGAGGCGGCGGCGGCGGCCGAGAAGAA--------------------------------------
hpt21      GTGTTTTCGTCCAACTCCAAGAGCAAGGCCGGTCAGATGGCGGGAGAGGCGGCGGCGGCGGCCGAGAAGAA--------------------------------------

           120       130       140       150       160       170       180       190       200       210       220
           |....|....|....|....|....|....|....|....|....|....|....|....|....|....|....|....|....|....|....|....|....
BPBF mRNA  gtgcaagtctggtaacaccaagttctgctactacaacaactacagcatgtcccagccgcgctacttctgcaaggcctgccgccgctactggacccatggtggctccctcc
BPBF_CDS   GTGCAAGTCTGGTAACACCAAGTTCTGCTACTACAACAACTACAGCATGTCCCAGCCGCGCTACTTCTGCAAGGCCTGCCGCCGCTACTGGACCCATGGTGGCTCCCTCC
hpt01      --------------------------------------------------------------------------------------------------------------
hpt02      --------------------------------------------------------------------------------------------------------------
hpt03      --------------------------------------------------------------------------------------------------------------
hpt04      --------------------------------------------------------------------------------------------------------------
hpt05      --------------------------------------------------------------------------------------------------------------
hpt06      --------------------------------------------------------------------------------------------------------------
hpt07      --------------------------------------------------------------------------------------------------------------
hpt08      --------------------------------------------------------------------------------------------------------------
hpt09      --------------------------------------------------------------------------------------------------------------
hpt10      --------------------------------------------------------------------------------------------------------------
hpt11      --------------------------------------------------------------------------------------------------------------
hpt12      --------------------------------------------------------------------------------------------------------------
hpt13      --------------------------------------------------------------------------------------------------------------
hpt14      --------------------------------------------------------------------------------------------------------------
hpt15      --------------------------------------------------------------------------------------------------------------
hpt16      --------------------------------------------------------------------------------------------------------------
hpt17      --------------------------------------------------------------------------------------------------------------
hpt18      --------------------------------------------------------------------------------------------------------------
hpt19      --------------------------------------------------------------------------------------------------------------
hpt20      --------------------------------------------------------------------------------------------------------------
hpt21      --------------------------------------------------------------------------------------------------------------
```

```
              230       240       250       260       270       280       290       300       310       320       330
              |....|....|....|....|....|....|....|....|....|....|....|....|....|....|....|....|....|....|....|....|....|....
BPBF mRNA     gcaacgtccccatcggtggtggttgccgcaagcccaaacgcccggggacctctgacgcccacaagctcggcatggcctcctcgtcggaacccacgggtgtcgtgcccccc
BPBF_CDS      GCAACGTCCCCATCGGTGGTGGTTGCCGCAAGCCCAAACGCCCGGGGACCTCTGACGCCCACAAGCTCGGCATGGCCTCCTCGTCGGAACCCACGGGTGTCGTGCCCCCC
hpt01         --------------------------------------------------------------------------------------------------------------
hpt02         --------------------------------------------------------------------------------------------------------------
hpt03         --------------------------------------------------------------------------------------------------------------
hpt04         --------------------------------------------------------------------------------------------------------------
hpt05         --------------------------------------------------------------------------------------------------------------
hpt06         --------------------------------------------------------------------------------------------------------------
hpt07         --------------------------------------------------------------------------------------------------------------
hpt08         --------------------------------------------------------------------------------------------------------------
hpt09         --------------------------------------------------------------------------------------------------------------
hpt10         --------------------------------------------------------------------------------------------------------------
hpt11         --------------------------------------------------------------------------------------------------------------
hpt12         --------------------------------------------------------------------------------------------------------------
hpt13         --------------------------------------------------------------------------------------------------------------
hpt14         --------------------------------------------------------------------------------------------------------------
hpt15         --------------------------------------------------------------------------------------------------------------
hpt16         --------------------------------------------------------------------------------------------------------------
hpt17         --------------------------------------------------------------------------------------------------------------
hpt18         --------------------------------------------------------------------------------------------------------------
hpt19         --------------------------------------------------------------------------------------------------------------
hpt20         --------------------------------------------------------------------------------------------------------------
hpt21         --------------------------------------------------------------------------------------------------------------

              340       350       360       370       380       390       400       410       420       430       440
              |....|....|....|....|....|....|....|....|....|....|....|....|....|....|....|....|....|....|....|....|....|....
BPBF mRNA     tcgaactgcacagggatgaactttgctaacgtcctcccgacgtttatgtctggtggctttgacattcaaagcagcctctccctgacaacttttgggtcatcatcctcatc
BPBF_CDS      TCGAACTGCACAGGGATGAACTTTGCTAACGTCCTCCCGACGTTTATGTCTGGTGGCTTTGACATTCAAAGCAGCCTCTCCCTGACAACTTTTGGGTCATCATCCTCATC
hpt01         --------------------------------------------------------------------------------------------------------------
hpt02         --------------------------------------------------------------------------------------------------------------
hpt03         --------------------------------------------------------------------------------------------------------------
hpt04         --------------------------------------------------------------------------------------------------------------
hpt05         --------------------------------------------------------------------------------------------------------------
hpt06         --------------------------------------------------------------------------------------------------------------
hpt07         --------------------------------------------------------------------------------------------------------------
hpt08         --------------------------------------------------------------------------------------------------------------
hpt09         --------------------------------------------------------------------------------------------------------------
hpt10         --------------------------------------------------------------------------------------------------------------
hpt11         --------------------------------------------------------------------------------------------------------------
hpt12         --------------------------------------------------------------------------------------------------------------
hpt13         --------------------------------------------------------------------------------------------------------------
hpt14         --------------------------------------------------------------------------------------------------------------
hpt15         --------------------------------------------------------------------------------------------------------------
hpt16         --------------------------------------------------------------------------------------------------------------
hpt17         --------------------------------------------------------------------------------------------------------------
hpt18         --------------------------------------------------------------------------------------------------------------
hpt19         --------------------------------------------------------------------------------------------------------------
hpt20         --------------------------------------------------------------------------------------------------------------
hpt21         --------------------------------------------------------------------------------------------------------------
```

```
              450       460       470       480       490       500       510       520       530       540       550
              |....|....|....|....|....|....|....|....|....|....|....|....|....|....|....|....|....|....|....|....|....|....
BPBF mRNA     caacccgacggcgttgatgtcccccggtgggacgacttcatttctggatgtgctgagaggtggtgcaggagggcttcttgatggcagcctcggtccaaacaatggctact
BPBF_CDS      CAACCCGACGGCGTTGATGTCCCCCGGTGGGACGACTTCATTTCTGGATGTGCTGAGAGGTGGTGCAGGAGGGCTTCTTGATGGCAGCCTCGGTCCAAACAATGGCTACT
hpt01         --------------------------------------------------------------------------------------------------------------
hpt02         --------------------------------------------------------------------------------------------------------------
hpt03         --------------------------------------------------------------------------------------------------------------
hpt04         --------------------------------------------------------------------------------------------------------------
hpt05         --------------------------------------------------------------------------------------------------------------
hpt06         --------------------------------------------------------------------------------------------------------------
hpt07         --------------------------------------------------------------------------------------------------------------
hpt08         --------------------------------------------------------------------------------------------------------------
hpt09         --------------------------------------------------------------------------------------------------------------
hpt10         --------------------------------------------------------------------------------------------------------------
hpt11         --------------------------------------------------------------------------------------------------------------
hpt12         --------------------------------------------------------------------------------------------------------------
hpt13         --------------------------------------------------------------------------------------------------------------
hpt14         --------------------------------------------------------------------------------------------------------------
hpt15         --------------------------------------------------------------------------------------------------------------
hpt16         --------------------------------------------------------------------------------------------------------------
hpt17         --------------------------------------------------------------------------------------------------------------
hpt18         --------------------------------------------------------------------------------------------------------------
hpt19         --------------------------------------------------------------------------------------------------------------
hpt20         --------------------------------------------------------------------------------------------------------------
hpt21         --------------------------------------------------------------------------------------------------------------

              560       570       580       590       600       610       620       630       640       650       660
              |....|....|....|....|....|....|....|....|....|....|....|....|....|....|....|....|....|....|....|....|....|....
BPBF mRNA     actatggtgggcatgccaacggatcaagcattgggatgttgatgactccgccaacggtgtcgtttggcattccaagtccgatgcaacaacatggcggtctcgtggttggt
BPBF_CDS      ACTATGGTGGGCATGCCAACGGATCAAGCATTGGGATGTTGATGACTCCGCCAACGGTGTCGTTTGGCATTCCAAGTCCGATGCAACAACATGGCGGTCTCGTGGTTGGT
hpt01         --TATGGTGGGCATGCCAACGGATCAAGCATTGGGATGTTGATGACTCCGCCAACGGTGTCGTTTGGCATTCCAAGTCCGATGCAACAACATGGCGGTCTCGTGGTTGGT
hpt02         --TATGGTGGGCATGCCAATGGATCAGGCATTGGGATGTTGATGACTCCGCCAACGGTATCGTTTGGCATTCCAAGTCCGATGCAACAACATGGCGGTCTCGTGGTTGGT
hpt03         --TATGGTGGGCATGCCAATGGATCANGCATTGGGATGTTGATGACTCCGCCAACGGTATCGTTTGGCATTCCAAGTCCGATGCAACAACATGGCGGTCTCGTGGTTGGT
hpt04         --TATGGTGGGCATGCCAACGGATCAAGCATTGGGATGTTGATGACTCCGCCAACGGTGTCGTTTGGCATTCCAAGTCCGATGCAACAACATGGCGGTCTCGTGGTTGGT
hpt05         --TATGGTGGGCATGCCAATGGATCAGGCATTGGGATGTTGATGACTCCGCCAACGGTATCGTTTGGCATTCCAAGTCCGATGCAACAACATGGCGGTCTCGTGGTTGGT
hpt06         --TATGGTGGGCATGCCAANGGATCAAGCATTGGGATGTTGATGACTCCGCCAACGGTGTCGTTTGGCATTCCAAGTCCGATGCAACAACATGGCGGTCTCGTGGTTGGT
hpt07         --TATGGTGGGCATGCCAATGGATCAGGCATTGGGATGTTGATGACTCCGCCAACGGTATCGTTTGGCATTCCAAGTCCGATGCAACAACATGGCGGTCTCGTGGTTGGT
hpt08         --TATGGTGGGCATGCCAANGGATCAAGCATTGGGATGTTGATGACTCCGCCAACGGTNTCGTTTGGCATTCCAAGTCCGATGCAACAACATGGCGGTCTCGTGGTTGGT
hpt09         --TATGGTGGGCATGCCAACGGATCAAGCATTGGGATGTTGATGACTCCGCCAACGGTGTCGTTTGGCATTCCAAGTCCGATGCAACAACATGGCGGTCTCGTGGTTGGT
hpt10         --TATGGTGGGCATGCCAACGGATCAAGCATTGGGATGTTGATGACTCCGCCAACGGTGTCGTTTGGCATTCCAAGTCCGATGCAACAACATGGCGGTCTCGTGGTTGGT
hpt11         --TATGGTGGGCATGCCAACGGATCAAGCATTGGGATGTTGATGACTCCGCCAACGGTGTCGTTTGGCATTCCAAGTCCGATGCAACAACATGGCGGTCTCGTGGTTGGT
hpt12         --TATGGTGGGCATGCCAACGGATCAAGCATTGGGATGTTGATGACTCCGCCAACGGTGTCGTTTGGCATTCCAAGTCCGATGCAACAACATGGCGGTCTCGTGGTTGGT
hpt13         --TATGGTGGGCATGCCAATGGATCAGGCATTGGGATGTTGATGACTCCGCCAACGGTATCGTTTGGCATTCCAAGTCCGATGCAACAACATGGCGGTCTCGTGGTTGGT
hpt14         --TATGGTGGGCATGCCAATGGATCAGGCATTGGGATGTTGATGACTCCGCCAACGGTATCGTTTGGCATTCCAAGTCCGATGCAACAACATGGCGGTCTCGTGGTTGGT
hpt15         --TATGGTGGGCATGCCAACGGATCANGCATTGGGATGTTGATGACTCCGCCAACGGTNTCGTTTGGCATTCCAAGTCCGATGCAACAACATGGCGGTCTCGTGGTTGGT
hpt16         --TATGGTGGGCATGCCAANGGATCANGCATTGGGATGTTGATGACTCCGCCAACGGTNTCGTTTGGCATTCCAAGTCCGATGCAACAACATGGCGGTCTCGTGGTTGGT
hpt17         --TATGGTGGGCATGCCAACGGATCAAGCATTGGGATGTTGATGACTCCGCCAACGGTGTCGTTTGGCATTCCAAGTCCGATGCAACAACATGGCGGTCTCGTGGTTGGT
hpt18         --TATGGTGGGCATGCCAANGGATCANGCATTGGGATGTTGATGACTCCGCCAACGGTNTCGTTTGGCATTCCAAGTCCGATGCAACAACATGGCGGTCTCGTGGTTGGT
hpt19         --TATGGTGGGCATGCCAACGGATCAAGCATTGGGATGTTGATGACTCCGCCAACGGTGTCGTTTGGCATTCCAAGTCCGATGCAACAACATGGCGGTCTCGTGGTTGGT
hpt20         --TATGGTGGGCATGCCAATGGATCAGGCATTGGGATGTTGATGACTCCGCCAACGGTATCGTTTGGCATTCCAAGTCCGATGCAACAACATGGCGGTCTCGTGGTTGGT
hpt21         --TATGGTGGGCATGCCAATGGATCAGGCATTGGGATGTTGATGACTCCGCCAACGGTATCGTTTGGCATTCCAAGTCCGATGCAACAACATGGCGGTCTCGTGGTTGGT
```

```
            670       680       690       700       710       720       730       740       750       760       770
            |....|....|....|....|....|....|....|....|....|....|....|....|....|....|....|....|....|....|....|....|....|....
BPBF mRNA   ggaaatggaataggtggcacaacttcttcaacatttcagggcagcgctggcgaggaaggagacgatggtacggggtccattatggggctccagtggcagccacatgttgg
BPBF_CDS    GGAAATGGAATAGGTGGCACAACTTCTTCAACATTTCAGGGCAGCGCTGGCGAGGAAGGAGACGATGGTACGGGGTCCATTATGGGGCTCCAGTGGCAGCCACATGTTGG
hpt01       GGAAATGGAATAGGTGGCACAACTTCTTCAACATTTCAGGGCAGCGCTGGCGAGGAAGGAGACGATGGTACGGGGTCCATTATGGGGCTCCAGTGGCAGCCACATGTTGG
hpt02       GGAAATGGAATAGGTGGCACAACTTCTTCAACATTTCAGGGCAACGCTGGCGAGGAAGGAGACGATGGTACGGGGTCCATTATGGGGCTCCAGTGGCAGCCACATGTTGG
hpt03       GGAAATGGAATAGGTGGCACAACTTCTTCAACATTTCAGGGCAACGCTGGCGAGGAAGGAGACGATGGTACGGGGTCCATTATGGGGCTCCAGTGGCAGCCACATGTTGG
hpt04       GGAAATGGAATAGGTGGCACAACTTCTTCAACATTTCAGGGCAGCGCTGGCGAGGAAGGAGACGATGGTACGGGGTCCATTATGGGGCTCCAGTGGCAGCCACATGTTGG
hpt05       GGAAATGGAATAGGTGGCACAACTTCTTCAACATTTCAGGGCAACGCTGGCGAGGAAGGAGACGATGGTACGGGGTCCATTATGGGGCTCCAGTGGCAGCCACATGTTGG
hpt06       GGAAATGGAATAGGTGGCACAACTTCTTCAACATTTCAGGGCAGCGCTGGCGAGGAAGGAGACGATGGTACGGGGTCCATTATGGGGCTCCAGTGGCAGCCACATGTTGG
hpt07       GGAAATGGAATAGGTGGCACAACTTCTTCAACATTTCAGGGCAACGCTGGCGAGGAAGGAGACGATGGTACGGGGTCCATTATGGGGCTCCAGTGGCAGCCACATGTTGG
hpt08       GGAAATGGAATAGGTGGCACAACTTCTTCAACATTTCAGGGCARCGCTGGCGAGGAAGGAGACGATGGTACGGGGTCCATTATGGGGCTCCAGTGGCAGCCACATGTTGG
hpt09       GGAAATGGAATAGGTGGCACAACTTCTTCAACATTTCAGGGCAGCGCTGGCGAGGAAGGAGACGATGGTACGGGGTCCATTATGGGGCTCCAGTGGCAGCCACATGTTGG
hpt10       GGAAATGGAATAGGTGGCACAACTTCTTCAACATTTCAGGGCAGCGCTGGCGAGGAAGGAGACGATGGTACGGGGTCCATTATGGGGCTCCAGTGGCAGCCACATGTTGG
hpt11       GGAAATGGAATAGGTGGCACAACTTCTTCAACATTTCAGGGCAGCGCTGGCGAGGAAGGAGACGATGGTACGGGGTCCATTATGGGGCTCCAGTGGCAGCCACATGTTGG
hpt12       GGAAATGGAATAGGTGGCACAACTTCTTCAACATTTCAGGGCAGCGCTGGCGAGGAAGGAGACGATGGTACGGGGTCCATTATGGGGCTCCAGTGGCAGCCACATGTTGG
hpt13       GGAAATGGAATAGGTGGCACAACTTCTTCAACATTTCAGGGCAACGCTGGCGAGGAAGGAGACGATGGTACGGGGTCCATTATGGGGCTCCAGTGGCAGCCACATGTTGG
hpt14       GGAAATGGAATAGGTGGCACAACTTCTTCAACATTTCAGGGCAACGCTGGCGAGGAAGGAGACGATGGTACGGGGTCCATTATGGGGCTCCAGTGGCAGCCACATGTTGG
hpt15       GGAAATGGAATAGGTGGCACAACTTCTTCAACATTTCAGGGCAGCGCTGGCGAGGAAGGAGACGATGGTACGGGGTCCATTATGGGGCTCCAGTGGCAGCCACATGTTGG
hpt16       GGAAATGGAATAGGTGGCACAACTTCTTCAACATTTCAGGGCARCGCTGGCGAGGAAGGAGACGATGGTACGGGGTCCATTATGGGGCTCCAGTGGCAGCCACATGTTGG
hpt17       GGAAATGGAATAGGTGGCACAACTTCTTCAACATTTCAGGGCAGCGCTGGCGAGGAAGGAGACGATGGTACGGGGTCCATTATGGGGCTCCAGTGGCAGCCACATGTTGG
hpt18       GGAAATGGAATAGGTGGCACAACTTCTTCAACATTTCAGGGCAACGCTGGCGAGGAAGGAGACGATGGTACGGGGTCCATTATGGGGCTCCAGTGGCAGCCACATGTTGG
hpt19       GGAAATGGAATAGGTGGCACAACTTCTTCAACATTTCAGGGCAGCGCTGGCGAGGAAGGAGACGATGGTACGGGGTCCATTATGGGGCTCCAGTGGCAGCCACATGTTGG
hpt20       GGAAATGGAATAGGTGGCACAACTTCTTCAACATTTCAGGGCAACGCTGGCGAGGAAGGAGACGATGGTACGGGGTCCATTATGGGGCTCCAGTGGCAGCCACATGTTGG
hpt21       GGAAATGGAATAGGTGGCACAACTTCTTCAACATTTCAGGGCAACGCTGGCGAGGAAGGAGACGATGGTACGGGGTCCATTATGGGGCTCCAGTGGCAGCCACATGTTGG

            780       790       800       810       820       830       840       850       860       870       880
            |....|....|....|....|....|....|....|....|....|....|....|....|....|....|....|....|....|....|....|....|....
BPBF mRNA   taatggtggcggtggtgttgttggattaggaggcgcgcatcatcttgggactgggaacaatgtgacgatgggcaacaacaacaataataacaaccagaacaacaataacg
BPBF_CDS    TAATGGTGGCGGTGGTGTTGTTGGATTAGGAGGCGCGCATCATCTTGGGACTGGGAACAATGTGACGATGGGCAACAACAACAATAATAACAACCAGAACAACAATAACG
hpt01       TAATGGTGGCGGTGGTGTTGTTGGATTAGGAGGCGCGCATCATCTTGGGACTGGGAACAATGTGACGATGGGCAACAACAACAATAATAACAACCAGAACAACAATAACG
hpt02       TAATGGTGGCGGTGGTGGTGTTGGATTAGGAGGCGCGCATCATCTTGGGACTGGGAACAATGTGACGATGGGCAACAACAACAATAATAATAACCAGAACAACAATAACG
hpt03       TAATGGTGGCGGTGGTGNTGTTGGATTAGGAGGCGCGCATCATCTTGGGACTGGGAACAATGTGACGATGGGCAACAACAACAATAATAACAACCAGAACAACAATAACG
hpt04       TAATGGTGGCGGTGGTGTTGTTGGATTAGGAGGCGCGCATCATCTTGGGACTGGGAACAATGTGACGATGGGCAACAACAACAATAATAACAACCAGAACAACAATAACG
hpt05       TAATGGTGGCGGTGGTGGTGTTGGATTAGGAGGCGCGCATCATCTTGGGACTGGGAACAATGTGACGATGGGCAACAACAACAATAATAACAACCAGAACAACAATAACG
hpt06       TAATGGTGGCGGTGGTGTTGTTGGATTAGGAGGCGCGCATCATCTTGGGACTGGGAACAATGTGACGATGGGCAACAACAACAATAATAACAACCAGAACAACAATAACG
hpt07       TAATGGTGGCGGTGGTGGTGTTGGATTAGGAGGCGCGCATCATCTTGGGACTGGGAACAATGTGACGATGGGCAACAACAACAATAATAATAACCAGAACAACAATAACG
hpt08       TAATGGTGGCGGTGGTGTTGTTGGATTAGGAGGCGCGCATCATCTTGGGACTGGGAACAATGTGACGATGGGCAACAACAACAATAATAACAACCAGAACAACAATAACG
hpt09       TAATGGTGGCGGTGGTGTTGTTGGATTAGGAGGCGCGCATCATCTTGGGACTGGGAACAATGTGACGATGGGCAACAACAACAATAATAACAACCAGAACAACAATAACG
hpt10       TAATGGTGGCGGTGGTGTTGTTGGATTAGGAGGCGCGCATCATCTTGGGACTGGGAACAATGTGACGATGGGCAACAACAACAATAATAACAACCAGAACAACAATAACG
hpt11       TAATGGTGGCGGTGGTGTTGTTGGATTAGGAGGCGCGCATCATCTTGGGACTGGGAACAATGTGACGATGGGCAACAACAACAATAATAACAACCAGAACAACAATAACG
hpt12       TAATGGTGGCGGTGGTGTTGTTGGATTAGGAGGCGCGCATCATCTTGGGACTGGGAACAATGTGACGATGGGCAACAACAACAATAATAACAACCAGAACAACAATAACG
hpt13       TAATGGTGGCGGTGGTGGTGTTGGATTAGGAGGCGCGCATCATCTTGGGACTGGGAACAATGTGACGATGGGCAACAACAACAATAATAACAACCAGAACAACAATAACG
hpt14       TAATGGTGGCGGTGGTGGTGTTGGATTAGGAGGCGCGCATCATCTTGGGACTGGGAACAATGTGACGATGGGCAACAACAACAATAATAATAACCAGAACAACAATAACG
hpt15       TAATGGTGGCGGTGGTGGTGTTGGATTAGGAGGCGCGCATCATCTTGGGACTGGGAACAATGTGACGATGGGCAACAACAACAATAATAACAACCAGAACAACAATAACG
hpt16       TAATGGTGGCGGTGGTGTTGTTGGATTAGGAGGCGCGCATCATCTTGGGACTGGGAACAATGTGACGATGGGCAACAACAACAATAATAACAACCAGAACAACAATAACG
hpt17       TAATGGTGGCGGTGGTGTTGTTGGATTAGGAGGCGCGCATCATCTTGGGACTGGGAACAATGTGACGATGGGCAACAACAACAATAATAACAACCAGAACAACAATAACG
hpt18       TAATGGTGGCGGTGGTGNTGTTGGATTAGGAGGCGCGCATCATCTTGGGACTGGGAACAATGTGACGATGGGCAACAACAACAATAATAACAACCAGAACAACAATAACG
hpt19       TAATGGTGGCGGTGGTGTTGTTGGATTAGGAGGCGCGCATCATCTTGGGACTGGGAACAATGTGACGATGGGCAACAACAACAATAATAACAACCAGAACAACAATAACG
hpt20       TAATGGTGGCGGTGGTGGTGTTGGATTAGGAGGCGCGCATCATCTTGGGACTGGGAACAATGTGACGATGGGCAACAACAACAATAATAACAACCAGAACAACAATAACG
hpt21       TAATGGTGGCGGTGGTGGTGTTGGATTAGGAGGCGCGCATCATCTTGGGACTGGGAACAATGTGACGATGGGCAACAACAACAATAATAATAACCAGAACAACAATAACG
```

```
                890       900       910       920       930       940       950       960       970       980       990
                |....|....|....|....|....|....|....|....|....|....|....|....|....|....|....|....|....|....|....|....|....|....
BPBF mRNA       gcggcggtgctggtgatgacgacgatggtgggtcatcgagggattgctactggatcaacaatggaggatcgaacccatggcaaagcctcctcaacagcacctccctgatg
BPBF_CDS        GCGGCGGTGCTGGTGATGACGACGATGGTGGGTCATCGAGGGATTGCTACTGGATCAACAATGGAGGATCGAACCCATGGCAAAGCCTCCTCAACAGCACCTCCCTGATG
hpt01           GCGGCGGTGCTGGTGATGACGACGATGGTGGGTCATCGAGGGATTGCTACTGGATCAACAATGGAGGATCGAACCCATGGCAAAGCCTCCTCAACAGCACCTCCCTGATG
hpt02           GCGGCGGTGCTGGTGATGACGACGATGGTGGGTCATCGAGGGATTGCTACTGGATCAACAATGGAGGATCGAACCCATGGCAGAGCCTCCTCAACAGCACCTCCCTGATG
hpt03           GCGGCGGTGCTGGTGATGACGACGATGGTGGGTCATCGAGGGATTGCTACTGGATCAACAATGGAGGATCGAACCCATGGCANAGCCTCCTCAACAGCACCTCCCTGATG
hpt04           GCGGCGGTGCTGGTGATGACGACGATGGTGGGTCATCGAGGGATTGCTACTGGATCAACAATGGAGGATCGAACCCATGGCAAAGCCTCCTCAACAGCACCTCCCTGATG
hpt05           GCGGCGGTGCTGGTGATGACGACGATGGTGGGTCATCGAGGGATTGCTACTGGATCAACAATGGAGGATCGAACCCATGGCAGAGCCTCCTCAACAGCACCTCCCTGATG
hpt06           GCGGCGGTGCTGGTGATGACGACGATGATGGGTCATCGAGGGATTGCTACTGGATCAACAATGGAGGATCGAACCCATGGCAAAGCCTCCTCAACAGCACCTCCCTGATG
hpt07           GCGGCGGTGCTGGTGATGACGACGATGGTGGGTCATCGAGGGATTGCTACTGGATCAACAATGGAGGATCGAACCCATGGCAGAGCCTCCTCAACAGCACCTCCCTGATG
hpt08           GCGGCGGTGCTGGTGATGACGACGATGGTGGGTCATCGAGGGATTGCTACTGGATCAACAATGGAGGATCGAACCCATGGCAAAGCCTCCTCAACAGCACCTCCCTGATG
hpt09           GCGGCGGTGCTGGTGATGACGACGATGATGGGTCATCGAGGGATTGCTACTGGATCAACAATGGAGGATCGAACCCATGGCAAAGCCTCCTCAACAGCACCTCCCTGATG
hpt10           GCGGCGGTGCTGGTGATGACGACGATGGTGGGTCATCGAGGGATTGCTACTGGATCAACAATGGAGGATCGAACCCATGGCAAAGCCTCCTCAACAGCACCTCCCTGATG
hpt11           GCGGCGGTGCTGGTGATGACGACGATGGTGGGTCATCGAGGGATTGCTACTGGATCAACAATGGAGGATCGAACCCATGGCAAAGCCTCCTCAACAGCACCTCCCTGATG
hpt12           GCGGCGGTGCTGGTGATGACGACGATGGTGGGTCATCGAGGGATTGCTACTGGATCAACAATGGAGGATCGAACCCATGGCAAAGCCTCCTCAACAGCACCTCCCTGATG
hpt13           GCGGCGGTGCTGGTGATGACGACGATGGTGGGTCATCGAGGGATTGCTACTGGATCAACAATGGAGGATCGAACCCATGGCAGAGCCTCCTCAACAGCACCTCCCTGATG
hpt14           GCGGCGGTGCTGGTGATGACGACGATGGTGGGTCATCGAGGGATTGCTACTGGATCAACAATGGAGGATCGAACCCATGGCAGAGCCTCCTCAACAGCACCTCCCTGATG
hpt15           GCGGCGGTGCTGGTGATGACGACGATGGTGGGTCATCGAGGGATTGCTACTGGATCAACAATGGAGGATCGAACCCATGGCARAGCCTCCTCAACAGCACCTCCCTGATG
hpt16           GCGGCGGTGCTGGTGATGACGACGATGGTGGGTCATCGAGGGATTGCTACTGGATCAACAATGGAGGATCGAACCCATGGCAAAGCCTCCTCAACAGCACCTCCCTGATG
hpt17           GCGGCGGTGCTAGTGATGACGACGATGGTGGGTCATCGAGGGATTGCTACTGGATCAACAATGGAGGATCGAACCCATGGCAAAGCCTCCTCAACAGCACCTCCCTGATG
hpt18           GCGGCGGTGCTGGTGATGACGACGATGGTGGGTCATCGAGGGATTGCTACTGGATCAACAATGGAGGATCGAACCCATGGCAAAGCCTCCTCAACAGCACCTCCCTGATG
hpt19           GCGGCGGTGCTGGTGATGACGACGATGGTGGGTCATCGAGGGATTGCTACTGGATCAACAATGGAGGATCGAACCCATGGCAAAGCCTCCTCAACAGCACCTCCCTGATG
hpt20           GCGGCGGTGCTGGTGATGACGACGATGGTGGGTCATCGAGGGATTGCTACTGGATCAACAATGGAGGATCGAACCCATGGCAGAGCCTCCTCAACAGCACCTCCCTGATG
hpt21           GCGGCGGTGCTGGTGATGACGACGATGGTGGGTCATCGAGGGATTGCTACTGGATCAACAATGGAGGATCGAACCCATGGCAGAGCCTCCTCAACAGCACCTCCCTGATG

                1000      1010      1020      1030      1040      1050      1060      1070      1080      1090      1100
                |....|....|....|....|....|....|....|....|....|....|....|....|....|....|....|....|....|....|....|....|....
BPBF mRNA       taagtggaagaagaaaatgggaaatagaggccattgaattggagaattgggtcgatcattttcttattactgtcggtgcttcttttttgttacttgcttgaagtatttcg
BPBF_CDS        TAA-----------------------------------------------------------------------------------------------------------
hpt01           TAAGTGGAAGAAGAAAATGGGAAATAGAGGCCATTGAATTGGAGAATTGGGTCGATCATTTTCTTATTACTGTCGGTGCTTCTTTTTTGTTACTTGCTTGAAGTATTTCG
hpt02           TAAGTGGAAGAAGAAAATGGGAAATATAGGCCATTGAATTGGAGAATTGGGTCGATCATTTTCTTATTACTGTCGATGCTTCTTTTTTGTTACTTGCTTGAAGTATTTCG
hpt03           TAAGTGGAAGAAGAAAATGGGAAATATAGGCCATTGAATTGGAGAATTGGGTCGATCATTTTCTTATTACTGTCGATGCTTCTTTTTTGTTACTTGCTTGAAGTATTTCG
hpt04           TAAGTGGAAGAAGAAAATGGGAAATAGAGGCCATTGAATTGGAGAATTGGGTCGATCATTTTCTTATTACTGTCGGTGCTTCTTTTTTGTTACTTGCTTGAAGTATTTCG
hpt05           TAAGTGGAAGAAGAAAATGGGAAATATAGGCCATTGAATTGGAGAATTGGGTCGATCATTTTCTTATTACTGTCGATGCTTCTTTTTTGTTACTTGCTTGAAGTATTTCG
hpt06           TAAGTGGAAGAAGAAAATGGGAAATAKAGGCCATTGAATTGGAGAATTGGGTCGATCATTTTCTTATTACTGTCGNTGCTTCTTTTTTGTTACTTGCTTGAAGTATTTCG
hpt07           TAAGTGGAAGAAGAAAATGGGAAATATAGGCCATTGAATTGGAGAATTGGGTCGATCATTTTCTTATTACTGTCGATGCTTCTTTTTTGTTACTTGCTTGAAGTATTTCG
hpt08           TAAGTGGAAGAAGAAAATGGGAAATATAGGCCATTGAATTGGAGAATTGGGTCGATCATTTTCTTATTACTGTCGNTGCTTCTTTTTTGTTACTTGCTTGAAGTATTTCG
hpt09           TAAGTGGAAGAAGAAAATGGGAAATAGAGGCCATTGAATTGGAGAATTGGGTCGATCATTTTCTTATTACTGTCGGTGCTTCTTTTTTGTTACTTGCTTGAAGTATTTCG
hpt10           TAAGTGGAAGAAGAAAATGGGAAATAGAGGCCATTGAATTGGAGAATTGGGTCGATCATTTTCTTATTACTGTCGGTGCTTCTTTTTTGTTACTTGCTTGAAGTATTTCG
hpt11           TAAGTGGAAGAAGAAAATGGGAAATAGAGGCCATTGAATTGGAGAATTGGGTCGATCATTTTCTTATTACTGTCGGTGCTTCTTTTTTGTTACTTGCTTGAAGTATTTCG
hpt12           TAAGTGGAAGAAGAAAATGGGAAATAGAGGCCATTGAATTGGAGAATTGGGTCGATCATTTTCTTATTACTGTCGGTGCTTCTTTTTTGTTACTTGCTTGAAGTATTTCG
hpt13           TAAGTGGAAGAAGAAAATGGGAAATATAGGCCATTGAATTGGAGAATTGGGTCGATCATTTTCTTATTACTGTCGATGCTTCTTTTTTGTTACTTGCTTGAAGTATTTCG
hpt14           TAAGTGGAAGAAGAAAATGGGAAATATAGGCCATTGAATTGGAGAATTGGGTCGATCATTTTCTTATTACTGTCGATGCTTCTTTTTTGTTACTTGCTTGAAGTATTTCG
hpt15           TAAGTGGAAGAAGAAAATGGGAAATATAGGCCATTGAATTGGAGAATTGGGTCGATCATTTTCTTATTACTGTCGATGCTTCTTTTTTGTTACTTGCTTGAAGTATTTCG
hpt16           TAAGTGGAAGAAGAAAATGGGAAATATAGGCCATTGAATTGGAGAATTGGGTCGATCATTTTCTTATTACTGTCGATGCTTCTTTTTTGTTACTTGCTTGAAGTATTTCG
hpt17           TAAGTGGAAGAAGAAAATGGGAAATAGAGGCCATTGAATTGGAGAATTGGGTCGATCATTTTCTTATTACTGTCGGTGCTTCTTTTTTGTTACTTGCTTGAAGTATTTCG
hpt18           TAAGTGGAAGAAGAAAATGGGAAATATAGGCCATTGAATTGGAGAATTGGGTCGATCATTTTCTTATTACTGTCGATGCTTCTTTTTTGTTACTTGCTTGAAGTATTTCG
hpt19           TAAGTGGAAGAAGAAAATGGGAAATAGAGGCCATTGAATTGGAGAATTGGGTCGATCATTTTCTTATTACTGTCGGTGCTTCTTTTTTGTTACTTGCTTGAAGTATTTCG
hpt20           TAAGTGGAAGAAGAAAATGGGAAATATAGGCCATTGAATTGGAGAATTGGGTCGATCATTTTCTTATTACTGTCGATGCTTCTTTTTTGTTACTTGCTTGAAGTATTTCG
hpt21           TAAGTGGAAGAAGAAAATGGGAAATATAGGCCATTGAATTGGAGAATTGGGTCGATCATTTTCTTATTACTGTCGATGCTTCTTTTTTGTTACTTGCTTGAAGTATTTCG
```

```
            1110      1120      1130      1140      1150      1160      1170      1180      1190      1200      1210
            |....|....|....|....|....|....|....|....|....|....|....|....|....|....|....|....|....|....|....|....|....|....
BPBF mRNA   ctcggtcattttcatgtgtattttaagaagggattattttgtttatttgagtgaaactcgagagagtgtgtcacattgggatgcttaatttaagtttatttgtttcttca
BPBF_CDS    --------------------------------------------------------------------------------------------------------------
hpt01       CTCGGTCATTTTCATGTGTATTTTAAGAAGGGATTATTTTGTTTATTTGAGTGAA-------------------------------------------------------
hpt02       CTCGGTCATTTTCATGTGTCTTTTAAGAAGGGATTATTTTGTTTATTTGAGTGAA-------------------------------------------------------
hpt03       CTCGGTCATTTTCATGTGTCTTTTAAGAAGGGATTATTTTGTTTATTTGAGTGAA-------------------------------------------------------
hpt04       CTCGGTCATTTTCATGTGTATTTTAAGAAGGGATTATTTTGTTTATTTGAGTGAA-------------------------------------------------------
hpt05       CTCGGTCATTTTCATGTGTCTTTTAAGAAGGGATTATTTTGTTTATTTGAGTGAA-------------------------------------------------------
hpt06       CTCGGTCATTTTCATGTGTCTTTTAAGAAGGGATTATTTTGTTTATTTGAGTGAA-------------------------------------------------------
hpt07       CTCGGTCATTTTCATGTGTCTTTTAAGAAGGGATTATTTTGTTTATTTGAGTGAA-------------------------------------------------------
hpt08       CTCGGTCATTTTCATGTGTNTTTTAAGAAGGGATTATTTTGTTTATTTGAGTGAA-------------------------------------------------------
hpt09       CTCGGTCATTTTCATGTGTCTTTTAAGAAGGGATTATTTTGTTTATTTGAGTGAA-------------------------------------------------------
hpt10       CTCGGTCATTTTCATGTGTATTTTAAGAAGGGATTATTTTGTTTATTTGAGTGAA-------------------------------------------------------
hpt11       CTCGGTCATTTTCATGTGTATTTTAAGAAGGGATTATTTTGTTTATTTGAGTGAA-------------------------------------------------------
hpt12       CTCGGTCATTTTCATGTGTATTTTAAGAAGGGATTATTTTGTTTATTTGAGTGAA-------------------------------------------------------
hpt13       CTCGGTCATTTTCATGTGTCTTTTAAGAAGGGATTATTTTGTTTATTTGAGTGAA-------------------------------------------------------
hpt14       CTCGGTCATTTTCATGTGTCTTTTAAGAAGGGATTATTTTGTTTATTTGAGTGAA-------------------------------------------------------
hpt15       CTCGGTCATTTTCATGTGTCTTTTAAGAAGGGATTATTTTGTTTATTTGAGTGAA-------------------------------------------------------
hpt16       CTCGGTCATTTTCATGTGTNTTTTAAGAAGGGATTATTTTGTTTATTTGAGTGAA-------------------------------------------------------
hpt17       CTCGGTCATTTTCATGTGTATTTTAAGAAGGGATTATTTTGTTTATTTGAGTGAA-------------------------------------------------------
hpt18       CTCGGTCATTTTCATGTGTNTTTTAAGAAGGGATTATTTTGTTTATTTGAGTGAA-------------------------------------------------------
hpt19       CTCGGTCATTTTCATGTGTATTTTAAGAAGGGATTATTTTGTTTATTTGAGTGAA-------------------------------------------------------
hpt20       CTCGGTCATTTTCATGTGTCTTTTAAGAAGGGATTATTTTGTTTATTTGAGTGAA-------------------------------------------------------
hpt21       CTCGGTCATTTTCATGTGTCTTTTAAGAAGGGATTATTTTGTTTATTTGAGTGAA-------------------------------------------------------

            1220      1230      1240      1250      1260      1270      1280
            |....|....|....|....|....|....|....|....|....|....|....|....|....|...
BPBF mRNA   cattaattatttaaggatttatggtttaatctttataaaagttatgttgtgtcaaaaaaaaaaaaaaaa
BPBF_CDS    ---------------------------------------------------------------------
hpt01       ---------------------------------------------------------------------
hpt02       ---------------------------------------------------------------------
hpt03       ---------------------------------------------------------------------
hpt04       ---------------------------------------------------------------------
hpt05       ---------------------------------------------------------------------
hpt06       ---------------------------------------------------------------------
hpt07       ---------------------------------------------------------------------
hpt08       ---------------------------------------------------------------------
hpt09       ---------------------------------------------------------------------
hpt10       ---------------------------------------------------------------------
hpt11       ---------------------------------------------------------------------
hpt12       ---------------------------------------------------------------------
hpt13       ---------------------------------------------------------------------
hpt14       ---------------------------------------------------------------------
hpt15       ---------------------------------------------------------------------
hpt16       ---------------------------------------------------------------------
hpt17       ---------------------------------------------------------------------
hpt18       ---------------------------------------------------------------------
hpt19       ---------------------------------------------------------------------
hpt20       ---------------------------------------------------------------------
hpt21       ---------------------------------------------------------------------
```

***HvGAMYB***

```
              -690      -680      -670      -660      -650      -640      -630      -620      -610      -600      -590
              |....|....|....|....|....|....|....|....|....|....|....|....|....|....|....|....|....|....|....|....|....|....
HvGAMYB mRNA  cgggacgaggaggaggaggagctgcagctcagctccgccccagctcagcccccggagcccgagctgatcgacccgccggcagcgagattcggccagg-------------
HvGAMYB_CDS   --------------------------------------------------------------------------------------------------------------
hpt01         CGGGACGAGGAGGAGGAGGAGCTGCAGCTCAGCTCCGCCCCAGCTCAGCCCCCGGAGCCCGAGCTGATCGACCCGCCGGCAGCGAGATTCGGCCAGGTAGCCCCCCTCTC
hpt02         CGGGACGAGGAGGAGGAGGAGCTGCAGCTCAGCTCCGCCCCAGCTCAGCCCCCGGAGCCCGAGCTGATCGACCCGCCGGCAGCGAGATTCGGCCAGGTAGCCCCCCTCTC
hpt03         CGGGACGAGGAGGAGGAGGAGCTGCAGCTCAGCTCCGCCCCAGCTCAGCCCCCGGAGCCCGAGCTGATCGACCCGCCGGCAGCGAGATTCGGCCAGGTAGCCCCCCTCTC
hpt04         CGGGACGAGGAGGAGGAGGAGCTGCAGCTCAGCTCCGCCCCAGCTCAGCCCCCGGAGCCCGAGCTGATCGACCCGCCGGCAGCGAGATTCGGCCAGGTAGCCCCCCTCTC
hpt05         CGGGACGAGGAGGAGGAGGAGCTGCAGCTCAGCTCCGCCCCAGCTCAGCCCCCGGAGCCCGAGCTGATCGACCCGCCGGCAGCGAGATTCGGCCAGGTAGCCCCCCTCTC
hpt06         CGGGACGAGGAGGAGGAGGAGCTGCAGCTCAGCTCCGCCCCAGCTCAGCCCCCGGAGCCCGAGCTGATCGACCCGCCGGCAGCGAGATTCGGCCAGGTAGCCCCCCTCTC
hpt07         CGGGACGAGGAGGAGGAGGAGCTGCAGCTCAGCTCCGCCCCAGCTCAGCCCCCGGAGCCCGAGCTGATCGACCCGCCGGCAGCGAGATTCGGCCAGGTAGCCCCCCTCTC
hpt08         CGGGACGAGGAGGAGGAGGAGCTGCAGCTCAGCTCCGCCCCAGCTCAGCCCCCGGAGCCCGAGCTGATCGACCCGCCGGCAGCGAGATTCGGCCAGGTAGCCCCCCTCTC
hpt09         CGGGACGAGGAGGAGGAGGAGCTGCAGCTCAGCTCCGCCCCAGCTCAGCCCCCGGAGCCCGAGCTGATCGACCCGCCGGCAGCGAGATTCGGCCAGGTAGCCCCCCTCTC
hpt10         CGGGACGAGGAGGAGGAGGAGCTGCAGCTCAGCTCCGCCCCAGCTCAGCCCCCGGAGCCCGAGCTGATCGACCCGCCGGCAGCGAGATTCGGCCAGGTAGCCCCCCTCTC
hpt14         CGGGACGAGGAGGAGGAGGAGCTGCAGCTCAGCTCCGCCCCAGCTCAGCCCCCGGAGCCCGAGCTGATCGACCCGCCGGCAGCGAGATTCGGCCAGGTAGCCCCCCTCTC
hpt11         CGGGACGAGGAGGAGGAGGAGCTGCAGCTCAGCTCCGCCCCAGCTCAGCCCCCGGAGCCCGAGCTGATCGACCCGCCGGCAGCGAGATTCGGCCAGGTAGCCCCCCTCTC
hpt12         CGGGACGAGGAGGAGGAGGAGCTGCAGCTCAGCTCCGCCCCAGCTCAGCCCCCGGAGCCCGAGCTGATCGACCCGCCGGCAGCGAGATTCGGCCAGGTAGCCCCCCTCTC
hpt13         CGGGACGAGGAGGAGGAGGAGCTGCAGCTCAGCTCCGCCCCAGCTCAGCCCCCGGAGCCCGAGCTGATCGACCCGCCGGCAGCGAGATTCGGCCAGGTAGCCCCCCTCTC
hpt15         CGGGACGAGGAGGAGGAGGAGCTGCAGCTCAGCTCCGCCCCAGCTCAGCCCCCGGAGCCCGAGCTGATCGACCCGCCGGCAGCGAGATTCGGCCAGGTAGCCCCCCTCTC
hpt16         CGGGACGAGGAGGAGGAGGAGCTGCAGCTCAGCTCCGCCCCAGCTCAGCCCCCGGAGCCCGAGCTGATCGACCCGCCGGCAGCGAGATTCGGCCAGGTAGCCCCCCTCTC
hpt17         CGGGACGAGGAGGAGGAGGAGCTGCAGCTCAGCTCCGCCCCAGCTCAGCCCCCGGAGCCCGAGCTGATCGACCCGCCGGCAGCGAGATTCGGCCAGGTAGCCCCCCTCTC
hpt18         CGGGACGAGGAGGAGGAGGAGCTGCAGCTCAGCTCCGCCCCAGCTCAGCCCCCGGAGCCCGAGCTGATCGACCCGCCGGCAGCGAGATTCGGCCAGGTAGCCCCCCTCTC
```

```
              -580      -570      -560      -550      -540      -530      -520      -510      -500      -490      -480
              |....|....|....|....|....|....|....|....|....|....|....|....|....|....|....|....|....|....|....|....|....
HvGAMYB mRNA  --------------------------------------------------------------------------------------------------------------
HvGAMYB_CDS   --------------------------------------------------------------------------------------------------------------
hpt01         CCCGCAGAATTCCGGGTTCCTTTCGGGTGGGCGCGGGGATGCTGATCCGTGGCTGCTCGGGTGCTGGAATTGCGTGCTTGGCCTTTTTT-CTTTCTTCGGCCCCGTGGGA
hpt02         CCCGCAGAATTCCGGGTTCCTTTCGGGTGGGCGCGGGGATGCTGATCCGTGGCTGCTCGGGTGCTGGAATTGCGTGCTTGGCCTTTTTTTCTTTCTTCGGCCCCGTGGGA
hpt03         CCCGCAGAATTCCGGGTTCCTTTCGGGTGGGCGCGGGGATGCTGATCCGTGGCTGCTCGGGTGCTGGAATTGCGTGCTTGGCCTTTTTT-CTTTCTTCGGCCCCGTGGGA
hpt04         CCCGCAGAATTCCGGGTTCCTTTCGGGTGGGCGCGGGGATGCTGATCCGTGGCTGCTCGGGTGCTGGAATTGCGTGCTTGGCCTTTTTT-CTTTCTTCGGCCCCGTGGGA
hpt05         CCCGCAGAATTCCGGGTTCCTTTCGGGTGGGCGCGGGGATGCTGATCCGTGGCTGCTCGGGTGCTGGAATTGCGTGCTTGGCCTTTTTTTCTTTCTTCGGCCCCGTGGGA
hpt06         CCCGCAGAATTCCGGGTTCCTTTCGGGTGGGCGCGGGGATGCTGATCCGTGGCTGCTCGGGTGCTGGAATTGCGTGCTTGGCCTTTTTTTCTTTCTTCGGCCCCGTGGGA
hpt07         CCCGCAGAATTCCGGGTTCCTTTCGGGTGGGCGCGGGGATGCTGATCCGTGGCTGCTCGGGTGCTGGAATTGCGTGCTTGGCCTTTTTTTCTTTCTTCGGCCCCGTGGGA
hpt08         CCCGCAGAATTCCGGGTTCCTTTCGGGTGGGCGCGGGGATGCTGATCCGTGTCTGCTCGGGTGCTGGAATTGCGTGCTTGGCCTTTTTT-CTTTCTTCGGCCCCGTGGGA
hpt09         CCCGCAGAATTCCGGGTTCCTTTCGGGTGGGCGCGGGGATGCTGATCCGTGGCTGCTCGGGTGCTGGAATTGCGTGCTTGGCCTTTTTTTCTTTCTTCGGCCCCGTGGGA
hpt10         CCCGCAGAATTCCGGGTTCCTTTCGGGTGGGCGCGGGGATGCTGATCCGTGGCTGCTCGGGTGCTGGAATTGCGTGCTTGGCCTTTTTT-CTTTCTTCGGCCCCGTGGGA
hpt14         CCCGCAGAATTCCGGGTTCCTTTCGGGTGGGCGCGGGGATGCTGATCCGTGGCTGCTCGGGTGCTGGAATTGCGTGCTTGGCCTTTTTTTCTTTCTTCGGCCCCGTGGGA
hpt11         CCCGCAGAATTCCGGGTTCCTTTCGGGTGGGCGCGGGGATGCTGATCCGTGTCTGCTCGGGTGCTGGAATTGCGTGCTTGGCCTTTTTT-CTTTCTTCGGCCCCGTGGGA
hpt12         CCCGCAGAATTCCGGGTTCCTTTCGGGTGGGCGCGGGGATGCTGATCCGTGGCTGCTCGGGTGCTGGAATTGCGTGCTTGGCCTTTTTT-CTTTCTTCGGCCCCGTGGGA
hpt13         CCCGCAGAATTCCGGGTTCCTTTCGGGTGGGCGCGGGGATGCTGATCCGTGGCTGCTCGGGTGCTGGAATTGCGTGCTTGGCCTTTTTT-CTTTCTTCGGCCCCGTGGGA
hpt15         CCCGCAGAATTCCGGGTTCCTTTCGGGTGGGCGCGGGGATGCTGATCCGTGGCTGCTCGGGTGCTGGAACTGCGTGCTTGGCCTTTTTTTCTTTCTTCGGCCCCGTGGGA
hpt16         CCCGCAGAATTCCGGGTTCCTTTCGGGTGGGCGCGGGGATGCTGATCCGTGGCTGCTCGGGTGCTGGAATTGCGTGCTTGGCCTTTTTTTCTTTCTTCGGCCCCGTGGGA
hpt17         CCCGCAGAATTCCGGGTTCCTTTCGGGTGGGCGCGGGGATGCTGATCCGTGGCTGCTCGGGTGCTGGAATTGCGTGCTTGGCCTTTTTTTCTTTCTTCGGCCCCGTGGGA
hpt18         CCCGCAGAATTCCGGGTTCCTTTCGGGTGGGCGCGGGGATGCTGATCCGTGGCTGCTCGGGTGCTGGAATTGCGTGCTTGGCCTTTTTT-CTTTCTTCGGCCCCGTGGGA
```

```
             -470      -460      -450      -440      -430      -420      -410      -400      -390      -380      -370
             |....|....|....|....|....|....|....|....|....|....|....|....|....|....|....|....|....|....|....|....|....|....
HvGAMYB mRNA --------------------------------------------------------------------------------------------------------------
HvGAMYB_CDS  --------------------------------------------------------------------------------------------------------------
hpt01        ATTTTCCATTCTGGGGGCACGAGGAGTGGTGTTTACGCCTTTTTGTCCGCTTGTCTAGTGGTTTGATCTGTCGATTGCGAGGACACCCTTTCCTCTTTTTCCCACCTAAA
hpt02        ATTTTCCATTCTGGGGGCACGAGGAGTGGTGTTTACGCCTTTTTGTCCGCTTGTCTAGTGGTTTGATCTGTCGATTGCGAGGACACCCTTTCCTCTTTTTCCCACCTAAA
hpt03        ATTTTCCATTCTGGGGGCACGAGGAGTGGTGTTTACGCCTTTTTGTCCGCTTGTCTAGTGGTTTGATCTGTCGATTGCGAGGACACCCTTTCCTCTTTTTCCCACCTAAA
hpt04        ATTTTCCATTCTGGGGGCACGAGGAGTGGTGTTTACGCCTTTTTGTCCGCTTGTCTAGTGGTTTGATCTGTCGATTGCGAGGACACCCTTTCCTCTTTTTCCCACCTAAA
hpt05        ATTTTCCATTCTGGGGGCACGAGGAGTGGTGTTTACGCCTTTTTGTCCGCTTGTCTAGTGGTTTGATCTGTCGATTGCGAGGACACCCTTTCCTCTTTTTCCCACCTAAA
hpt06        ATTTTCCATTCTGGGGGCACGAGGAGTGGTGTTTACGCCTTTTTGTCCGCTTGTCTAGTGGTTTGATCTGTCGATTGCGAGGACACCCTTTCCTCTTTTTCCCACCTAAA
hpt07        ATTTTCCATTCTGGGGGCACGAGGAGTGGTGTTTACGCCTTTTTGTCCGCTTGTCTAGTGGTTTGATCTGTCGATTGCGAGGACACCCTTTCCTCTTTTTCCCACCTAAA
hpt08        ATTTTCCATTCTGGGGGCACGAGGAGTGGTGTTTACGCCTTTTTGTCCGCTTGTCTAGTGGTTTGATCTGTCGATTGCGAGGACACCCTTTCCTCTTTTTCCCACCTAAA
hpt09        ATTTTCCATTCTGGGGGCACGAGGAGTGGTGTTTACGCCTTTTTGTCCGCTTGTCTAGTGGTTTGATCTGTCGATTGCGAGGACACCCTTTCCTCTTTTTCCCACCTAAA
hpt10        ATTTTCCATTCTGGGGGCACGAGGAGTGGTGTTTACGCCTTTTTGTCCGCTTGTCTAGTGGTTTGATCTGTCGATTGCGAGGACACCCTTTCCTCTTTTTCCCACCTAAA
hpt14        ATTTTCCATTCTGGGGGCACGAGGAGTGGTGTTTACGCCTTTTTGTCCGCTTGTCTAGTGGTTTGATCTGTCGATTGCGAGGACACCCTTTCCTCTTTTTCCCACCTAAA
hpt11        ATTTTCCATTCTGGGGGCACGAGGAGTGGTGTTTACGCCTTTTTGTCCGCTTGTCTAGTGGTTTGATCTGTCGATTGCGAGGACACCCTTTCCTCTTTTTCCCACCTAAA
hpt12        ATTTTCCATTCTGGGGGCACGAGGAGTGGTGTTTACGCCTTTTTGTCCGCTTGTCTAGTGGTTTGATCTGTCGATTGCGAGGACACCCTTTCCTCTTTTTCCCACCTAAA
hpt13        ATTTTCCATTCTGGGGGCACGAGGAGTGGTGTTTACGCCTTTTTGTCCGCTTGTCTAGTGGTTTGATCTGTCGATTGCGAGGACACCCTTTCCTCTTTTTCCCACCTAAA
hpt15        ATTTTCCATTCTGGGGGCACGAGGAGTGGTGTTTACGCCTTTTTGTCCGCTTGTCTAGTGGTTTGATCTGTCGATTGCGAGGACACCCTTTCCTCTTTTTCCCACCTAAA
hpt16        ATTTTCCATTCTGGGGGCACGAGGAGTGGTGTTTACGCCTTTTTGTCCGCTTGTCTAGTGGTTTGATCTGTCGATTGCGAGGACACCCTTTCCTCTTTTTCCCACCTAAA
hpt17        ATTTTCCATTCTGGGGGCACGAGGAGTGGTGTTTACGCCTTTTTGTCCGCTTGTCTAGTGGTTTGATCTGTCGATTGCGAGGACACCCTTTCCTCTTTTTCCCACCTAAA
hpt18        ATTTTCCATTCTGGGGGCACGAGGAGTGGTGTTTACGCCTTTTTGTCCGCTTGTCTAGTGGTTTGATCTGTCGATTGCGAGGACACCCTTTCCTCTTTTTCCCACCTAAA
```

```
             -360      -350      -340      -330      -320      -310      -300      -290      -280      -270      -260
             |....|....|....|....|....|....|....|....|....|....|....|....|....|....|....|....|....|....|....|....|....
HvGAMYB mRNA --------------------------------------------------------------------------------------------------------------
HvGAMYB_CDS  --------------------------------------------------------------------------------------------------------------
hpt01        TCCTTGGCTAAAACTGCTGTGCTTGAGCTCAAACATGTTCAATTCAATTCTTAACCGGGTATTAAATGCCCCTGCCCTGCTGAATTTGGGAAGGAAGTGATTCCAGTCTG
hpt02        TCCTTGGCTAAAACTGCTGTGCTTGAGCTCAAACATGTTCAATTCAATTCTTAACCGGGTATTAAATGCCCCTGCCCTGCTGAATTTGGGAAGGAAGTGATTCCAGTCTG
hpt03        TCCTTGGCTAAAACTGCTGTGCTTGAGCTCAAACATGTTCAATTCAATTCTTAACCGGGTATTAAATGCCCCTGCCCTGCTGAATTTGGGAAGGAAGTGATTCCAGTCTG
hpt04        TCCTTGGCTAAAACTGCTGTGCTTGAGCTCAAACATGTTCAATTCAATTCTTAACCGGGTATTAAATGCCCCTGCCCTGCTGAATTTGGGAAGGAAGTGATTCCAGTCTG
hpt05        TCCTTGGCTAAAACTGCTGTGCTTGAGCTCAAACATGTTCAATTCAATTCTTAACCGGGTATTAAATGCCCCTGCCCTGCTGAATTTGGGAAGGAAGTGATTCCAGTCTG
hpt06        TCCTTGGCTAAAACTGCTGTGCTTGAGCTCAAACATGTTCAATTCAATTCTTAACCGGGTATTAAATGCCCCTGCCCTGCTGAATTTGGGAAGGAAGTGATTCCAGTCTG
hpt07        TCCTTGGCTAAAACTGCTGTGCTTGAGCTCAAACATGTTCAATTCAATTCTTAACCGGGTATTAAATGCCCCTGCCCTGCTGAATTTGGGAAGGAAGTGATTCCAGTCTG
hpt08        TCCTTGGCTAAAACTGCTGTGCTTGAGCTCAAACATGTTCAATTCAATTCTTAACCGGGTATTAAATGCCCCTGCCCTGCTGAATTTGGGAAGGAAGTGATTCCAGTCTG
hpt09        TCCTTGGCTAAAACTGCTGTGCTTGAGCTCAAACATGTTCAATTCAATTCTTAACCGGGTATTAAATGCCCCTGCCCTGCTGAATTTGGGAAGGAAGTGATTCCAGTCTG
hpt10        TCCTTGGCTAAAACTGCTGTGCTTGAGCTCAAACATGTTCAATTCAATTCTTAACCGGGTATTAAATGCCCCTGCCCTGCTGAATTTGGGAAGGAAGTGATTCCAGTCTG
hpt14        TCCTTGGCTAAAACTGCTGTGCTTGAGCTCAAACATGTTCAATTCAATTCTTAACCGGGTATTAAATGCCCCTGCCCTGCTGAATTTGGGAAGGAAGTGATTCCAGTCTG
hpt11        TCCTTGGCTAAAACTGCTGTGCTTGAGCTCAAACATGTTCAATTCAATTCTTAACCGGGTATTAAATGCCCCTGCCCTGCTGAATTTGGGAAGGAAGTGATTCCAGTCTG
hpt12        TCCTTGGCTAAAACTGCTGTGCTTGAGCTCAAACATGTTCAATTCAATTCTTAACCGGGTATTAAATGCCCCTGCCCTGCTGAATTTGGGAAGGAAGTGATTCCAGTCTG
hpt13        TCCTTGGCTAAAACTGCTGTGCTTGAGCTCAAACATGTTCAATTCAATTCTTAACCGGGTATTAAATGCCCCTGCCCTGCTGAATTTGGGAAGGAAGTGATTCCAGTCTG
hpt15        TCCTTGGCTAAAACTGCTGTGCTTGAGCTCAAACATGTTCAATTCAATTCTTAACCGGGTATTAAATGCCCCTGCCCTGCTGAATTTGGGAAGGAAGTGATTCCAGTCTG
hpt16        TCCTTGGCTAAAACTGCTGTGCTTGAGCTCAAACATGTTCAATTCAATTCTTAACCGGGTATTAAATGCCCCTGCCCTGCTGAATTTGGGAAGGAAGTGATTCCAGTCTG
hpt17        TCCTTGGCTAAAACTGCTGTGCTTGAGCTCAAACATGTTCAATTCAATTCTTAACCGGGTATTAAATGCCCCTGCCCTGCTGAATTTGGGAAGGAAGTGATTCCAGTCTG
hpt18        TCCTTGGCTAAAACTGCTGTGCTTGAGCTCAAACATGTTCAATTCAATTCTTAACCGGGTATTAAATGCCCCTGCCCTGCTGAATTTGGGAAGGAAGTGATTCCAGTCTG
```

```
                -250      -240      -230      -220      -210      -200      -190      -180      -170      -160      -150
                |....|....|....|....|....|....|....|....|....|....|....|....|....|....|....|....|....|....|....|....|....|....
HvGAMYB mRNA    --------------------------------------------------------------------------------------------------------------
HvGAMYB_CDS     --------------------------------------------------------------------------------------------------------------
hpt01           GTGGCTTCTCCTGGTTCTTGAGTTGCCGCGTAATAAATACATGCTTTGTTCTCGTGATTTTCTAAACAAGTTTGATCGGCTTCTGGTGCTGTTTGATTGCATAATTCCAA
hpt02           GTGGCTTCTCCTGGTTCTTGAGTTGCCGCGTAATAAATACATGCTTTGTTCTCGTGATTTTCTAAACAAGTTTGATCGGCTTCTGGTGCTGTTTGATTGCATAATTCCAA
hpt03           GTGGCTTCTCCTGGTTCTTGAGTTGCCGCGTAATAAATACATGCTTTGTTCTCGTGATTTTCTAAACAAGTTTGATCGGCTTCTGGTGCTGTTTGATTGCATAATTCCAA
hpt04           GTGGCTTCTCCTGGTTCTTGAGTTGCCGCGTAATAAATACATGCTTTGTTCTCGTGATTTTCTAAACAAGTTTGATCGGCTTCTGGTGCTGTTTGATTGCATAATTCCAA
hpt05           GTGGCTTCTCCTGGTTCTTGAGTTGCCGCGTAATAAATACATGCTTTGTTCTCGTGATTTTCTAAACAAGTTTGATCGGCTTCTGGTGCTGTTTGATTGCATAATTCCAA
hpt06           GTGGCTTCTCCTGGTTCTTGAGTTGCCGCGTAATAAATACATGCTTTGTTCTCGTGATTTTCTAAACAAGTTTGATCGGCTTCTGGTGCTGTTTGATTGCATAATTCCAA
hpt07           GTGGCTTCTCCTGGTTCTTGAGTTGCCGCGTAATAAATACATGCTTTGTTCTCGTGATTTTCTAAACAAGTTTGATCGGCTTCTGGTGCTGTTTGATTGCATAATTCCAA
hpt08           GTGGCTTCTCCTGGTTCTTGAGTTGCCGCGTAATAAATACATGCTTTGTTCTCGTGATTTTCTAAACAAGTTTGATCGGCTTCTGGTGCTGTTTGATTGCATAATTCCAA
hpt09           GTGGCTTCTCCTGGTTCTTGAGTTGCCGCGTAATAAATACATGCTTTGTTCTCGTGATTTTCTAAACAAGTTTGATCGGCTTCTGGTGCTGTTTGATTGCATAATTCCAA
hpt10           GTGGCTTCTCCTGGTTCTTGAGTTGCCGCGTAATAAATACATGCTTTGTTCTCGTGATTTTCTAAACAAGTTTGATCGGCTTCTGGTGCTGTTTGATTGCATAATTCCAA
hpt14           GTGGCTTCTCCTGGTTCTTGAGTTGCCGCGTAATAAATACATGCTTTGTTCTCGTGATTTTCTAAACAAGTTTGATCGGCTTCTGGTGCTGTTTGATTGCATAATTCCAA
hpt11           GTGGCTTCTCCTGGTTCTTGAGTTGCCGCGTAATAAATACATGCTTTGTTCTCGTGATTTTCTAAACAAGTTTGATCGGCTTCTGGTGCTGTTTGATTGCATAATTCCAA
hpt12           GTGGCTTCTCCTGGTTCTTGAGTTGCCGCGTAATAAATACATGCTTTGTTCTCGTGATTTTCTAAACAAGTTTGATCGGCTTCTGGTGCTGTTTGATTGCATAATTCCAA
hpt13           GTGGCTTCTCCTGGTTCTTGAGTTGCCGCGTAATAAATACATGCTTTGTTCTCGTGATTTTCTAAACAAGTTTGATCGGCTTCTGGTGCTGTTTGATTGCATAATTCCAA
hpt15           GTGGCTTCTCCTGGTTCTTGAGTTGCCGCGTAATAAATACATGCTTTGTTCTCGTGATTTTCTAAACAAGTTTGATCGGCTTCTGGTGCTGTTTGATTGCATAATTCCAA
hpt16           GTGGCTTCTCCTGGTTCTTGAGTTGCCGCGTAATAAATACATGCTTTGTTCTCGTGATTTTCTAAACAAGTTTGATCGGCTTCTGGTGCTGTTTGATTGCATAATTCCAA
hpt17           GTGGCTTCTCCTGGTTCTTGAGTTGCCGCGTAATAAATACATGCTTTGTTCTCGTGATTTTCTAAACAAGTTTGATCGGCTTCTGGTGCTGTTTGATTGCATAATTCCAA
hpt18           GTGGCTTCTCCTGGTTCTTGAGTTGCCGCGTAATAAATACATGCTTTGTTCTCGTGATTTTCTAAACAAGTTTGATCGGCTTCTGGTGCTGTTTGATTGCATAATTCCAA

                -140      -130      -120      -110      -100      -90       -80       -70       -60       -50       -40
                |....|....|....|....|....|....|....|....|....|....|....|....|....|....|....|....|....|....|....|....|....
HvGAMYB mRNA    --------------------------------------------------------------------------------------------------------------
HvGAMYB_CDS     --------------------------------------------------------------------------------------------------------------
hpt01           CAGTCCTGTGTGATTCGAGCAGTGGTAGAACTCGATTTTCTCCACAAAAAAGGTACTAGTTGTGGTCAATTCGTAATCTAACAAAAAATTCTTTCTTTTTCTTTGTTTCT
hpt02           CAGTCCTGTGTGATTCGAGCAGTGGTAGAACTCGATTTTCTCCACAAAAAAGGTACTAGTTGTGGTCAATTCGTAATCTAACAAAAAATTCTTTCTTTTTCTTTGTTTCT
hpt03           CAGTCCTGTGTGATTCGAGCAGTGGTAGAACTCGATTTTCTCCACAAAAAAGGTACTAGTTGTGGTCAATTCGTAATCTAACAAAAAATTCTTTCTTTTTCTTTGTTTCT
hpt04           CAGTCCTGTGTGATTCGAGCAGTGGTAGAACTCGATTTTCTCCACAAAAAAGGTACTAGTTGTGGTCAATTCGTAATCTAACAAAAAATTCTTTCTTTTTCTTTGTTTCT
hpt05           CAGTCCTGTGTGATTCGAGCAGTGGTAGAACTCGATTTTCTCCACAAAAAAGGTACTAGTTGTGGTCAATTCGTAATCTAACAAAAAATTCTTTCTTTTTCTTTGTTTCT
hpt06           CAGTCCTGTGTGATTCGAGCAGTGGTAGARCTCGATTTTCTCCACAAAAAAGGTACTAGTTGTGGTCAATTCGTAATCTAACAAAAAATTCTTTCTTTTTCTTTGTTTCT
hpt07           CAGTCCTGTGTGATTCGAGCAGTGGTAGAACTCGATTTTCTCCACAAAAAAGGTACTAGTTGTGGTCAATTCGTAATCTAACAAAAAATTCTTTCTTTTTCTTTGTTTCT
hpt08           CAGTCCTGTGTGATTCGAGCAGTGGTAGAACTCGATTTTCTCCACAAAAAAGGTACTAGTTGTGGTCAATTCGTAATCTAACAAAAAATTCTTTCTTTTTCTTTGTTTCT
hpt09           CAGTCCTGTGTGATTCGAGCAGTGGTAGAACTCGATTTTCTCCACAAAAAAGGTACTAGTTGTGGTCAATTCGTAATCTAACAAAAAATTCTTTCTTTTTCTTTGTTTCT
hpt10           CAGTCCTGTGTGATTCGAGCAGTGGTAGAACTCGATTTTCTCCACAAAAAAGGTACTAGTTGTGGTCAATTCGTAATCTAACAAAAAATTCTTTCTTTTTCTTTGTTTCT
hpt14           CAGTCCTGTGTGATTCGAGCAGTCGTAGAACTCGATTTTCTCCACAAAAAAGGTACTAGTTGTGGTCAATTCGTAATCTAACAAAAAATTCTTTCTTTTTCTTTGTTTCT
hpt11           CAGTCCTGTGTGATTCGAGCAGTGGTAGAACTCGATTTTCTCCACAAAAAAGGTACTAGTTGTGGTCAATTCGTAATCTAACAAAAAATTCTTTCTTTTTCTTTGTTTCT
hpt12           CAGTCCTGTGTGATTCGAGCAGTGGTAGAACTCGATTTTCTCCACAAAAAAGGTACTAGTTGTGGTCAATTCGTAATCTAACAAAAAATTCTTTCTTTTTCTTTGTTTCT
hpt13           CAGTCCTGTGTGATTCGAGCAGTGGTAGAACTCGATTTTCTCCACAAAAAAGGTACTAGTTGTGGTCAATTCGTAATCTAACAAAAAATTCTTTCTTTTTCTTTGTTTCT
hpt15           CAGTCCTGTGTGATTCGAGCAGTGGTAGAACTCGATTTTCTCCACAAAAAAGGTACTAGTTGTGGTCAATTCGTAATCTAACAAAAAATTCTTTCTTTTTCTTTGTTTCT
hpt16           CAGTCCTGTGTGATTCGAGCAGTCGTAGAACTCGATTTTCTCCACAAAAAAGGTACTAGTTGTGGTCAATTCGTAATCTAACAAAAAATTCTTTCTTTTTCTTTGTTTCT
hpt17           CAGTCCTGTGTGATTCGAGCAGTGGTAGAGCTCGATTTTCTCCACAAAAAAGGTACTAGTTGTGGTCAATTCGTAATCTAACAAAAAATTCTTTCTTTTTCTTTGTTTCT
hpt18           CAGTCCTGTGTGATTCGAGCAGTGGTAGAACTCGATTTTCTCCACAAAAAAGGTACTAGTTGTGGTCAATTCGTAATCTAACAAAAAATTCTTTCTTTTTCTTTGTTTCT
```

```
              -30        -20        -10        -1        10         20         30         40         50         60         70         80
              |....|....|....|....|....|....|...|1...|....|....|....|....|....|....|....|....|....|....|....|....|....|....|....|
HvGAMYB mRNA  -------------gcacgatcgacggagagatgtaccgggtgaagagcgagagcgactgcgagatgatgcaccaggaggaccagatggactcgccggtgggcgacgacgg
HvGAMYB_CDS   ------------------------------ATGTACCGGGTGAAGAGCGAGAGCGACTGCGAGATGATGCACCAGGAGGACCAGATGGACTCGCCGGTGGGCGACGACGG
hpt01         GGGGTAATTCAGGGCACGATCGACGGAGAGATGTACCGGGTGAAGAGCGAGAGCGACTGCGAGATGATGCACCAGGAGGACCAGATGGACTCGCCGGTGGGCGACGACGG
hpt02         GGGGTAATTCAGGGCACGATCGACGGAGAGATGTACCGGGTGAAGAGCGAGAGCGACTGCGAGATGATGCACCAGGAGGACCAGATGGACTCGCCGGTGGGCGACGACGG
hpt03         GGGGTAATTCAGGGCACGATCGACGGAGAGATGTACCGGGTGAAGAGCGAGAGCGACTGCGAGATGATGCACCAGGAGGACCAGATGGACTCGCCGGTGGGCGACGACGG
hpt04         GGGATAATTCAGGGCACGATCAACGGAGAGATGTACCGGGTGAAGAGCGAGAGCGACTGCGAGATGATGCACCAGGAGGACCAGATGGACTCGCCGGTGGGCGACGACGG
hpt05         GGGGTAATTCAGGGCACGATCGACGGAGAGATGTACCGGGTGAAGAGCGAGAGCGACTGCGAGATGATGCACCAGGAGGACCAGATGGACTCGCCGGTGGGCGACGACGG
hpt06         GGGGTAATTCAGGGCACGATCGACGGAGAGATGTACCGGGTGAAGAGCGAGAGCGACTGCGAGATGATGCACCAGGAGGACCAGATGGACTCGCCGGTGGGCGACGACGG
hpt07         GGGGTAATTCAGGGCACGATCGACGGAGAGATGTACCGGGTGAAGAGCGAGAGCGACTGCGAGATGATGCACCAGGAGGACCAGATGGACTCGCCGGTGGGCGACGACGG
hpt08         GGGGTAATTCAGGGCACGATCGACGGAGAGATGTACCGGGTGAAGAGCGAGAGCGACTGCGAGATGATGCACCAGGAGGACCAGATGGACTCGCCGGTGGGCGACGACGG
hpt09         GGGGTAATTCAGGGCACGATCGACGGAGAGATGTACCGGGTGAAGAGCGAGAGCGACTGCGAGATGATGCACCAGGAGGACCAGATGGACTCGCCGGTGGGCGACGACGG
hpt10         GGGATAATTCAGGGCACGATCAACGGAGAGATGTACCGGGTGAAGAGCGAGAGCGACTGCGAGATGATGCACCAGGAGGACCAGATGGACTCGCCGGTGGGCGACGACGG
hpt14         GGGGTAATTCAGGGCACGATCGACGGAGAGATGTACCGGGTGAAGAGCGAGAGCGACTGCGAGATGATGCACCAGGAGGACCAGATGGACTCGCCGGTGGGCGACGACGG
hpt11         GGGGTAATTCAGGGCACGATCGACGGAGAGATGTACCGGGTGAAGAGCGAGAGCGACTGCGAGATGATGCACCAGGAGGACCAGATGGACTCGCCGGTGGGCGACGACGG
hpt12         GGGATAATTCAGGGCACGATCAACGGAGAGATGTACCGGGTGAAGAGCGAGAGCGACTGCGAGATGATGCACCAGGAGGACCAGATGGACTCGCCGGTGGGCGACGACGG
hpt13         GGGGTAATTCAGGGCACGATCGACGGAGAGATGTACCGGGTGAAGAGCGAGAGCGACTGCGAGATGATGCACCAGGAGGACCAGATGGACTCGCCGGTGGGCGACGACGG
hpt15         GGGGTAATTCAGGGCACGATCGACGGAGAGATGTACCGGGTGAAGAGCGAGAGCGACTGCGAGATGATGCACCAGGAGGACCAGATGGACTCGCCGGTGGGCGACGACGG
hpt16         GGGGTAATTCAGGGCACGATCGACGGAGAGATGTACCGGGTGAAGAGCGAGAGCGACTGCGAGATGATGCACCAGGAGGACCAGATGGACTCGCCGGTGGGCGACGACGG
hpt17         GGGGTAATTCAGGGCACGATCGACGGAGAGATGTACCGGGTGAAGAGCGAGAGCGACTGCGAGATGATGCACCAGGAGGACCAGATGGACTCGCCGGTGGGCGACGACGG
hpt18         GGGGTAATTCAGGGCACGATCGACGGAGAGATGTACCGGGTGAAGAGCGAGAGCGACTGCGAGATGATGCACCAGGAGGACCAGATGGACTCGCCGGTGGGCGACGACGG

                  90        100        110        120        130        140        150        160        170        180        190
              ....|....|....|....|....|....|....|....|....|....|....|....|....|....|....|....|....|....|....|....|....|....|
HvGAMYB mRNA  cagcagcggcggagggtcgcctcacaggggcggcgggccgcctctgaagaaggggccctggacgtccgcggaggacgccatcctggtggactacgtgaagaagcacggcg
HvGAMYB_CDS   CAGCAGCGGCGGAGGGTCGCCTCACAGGGGCGGCGGGCCGCCTCTGAAGAAGGGGCCCTGGACGTCGGCGGAGGACGCCATCCTGGTGGACTACGTGAAGAAGCACGGCG
hpt01         CAGCAGCGGCGGAGGGTCGCCTCACAGGGGCGGCGGGCCGCCTCTGAAGAAGGGGCCCTGGACGTCGGCGGAGGACGCCATCCTGGTGGACTACGTGAAGAAGCACGGCG
hpt02         CAGCAGCGGCGGAGGGTCGCCTCACAGGGGCGGCGGGCCGCCTCTGAAGAAGGGGCCCTGGACGTCCGCGGAGGACGCCATCCTGGTGGACTACGTGAAGAAGCACGGCG
hpt03         CAGCAGCGGCGGAGGGTCGCCTCACAGGGGCGGCGGGCCGCCTCTGAAGAAGGGGCCCTGGACGTCGGCGGAGGACGCCATCCTGGTGGACTACGTGAAGAAGCACGGCG
hpt04         CAGCAGCGGCGGAGGGTCGCCTCACAGGGGCGGCGGGCCGCCTCTGAAGAAGGGGCCCTGGACGTCCGCGGAGGACGCCATCCTGGTGGACTACGTGAAGAAGCACGGCG
hpt05         CAGCAGCGGCGGAGGGTCGCCTCACAGGGGCGGCGGGCCGCCTCTGAAGAAGGGGCCCTGGACGTCGGCGGAGGACGCCATCCTGGTGGACTACGTGAAGAAGCACGGCG
hpt06         CAGCAGCGGCGGAGGGTCGCCTCACAGGGGCGGCGGGCCGCCTCTGAAGAAGGGGCCCTGGACGTCCGCGGAGGACGCCATCCTGGTGGACTACGTGAAGAAGCACGGCG
hpt07         CAGCAGCGGCGGAGGGTCGCCTCACAGGGGCGGCGGGCCGCCTCTGAAGAAGGGGCCCTGGACGTCCGCGGAGGACGCCATCCTGGTGGACTACGTGAAGAAGCACGGCG
hpt08         CAGCAGCGGCGGAGGGTCGCCTCACAGGGGCGGCGGGCCGCCTCTGAAGAAGGGGCCCTGGACGTCCGCGGAGGACGCCATCCTGGTGGACTACGTGAAGAAGCACGGCG
hpt09         CAGCAGCGGCGGAGGGTCGCCTCACAGGGGCGGCGGGCCGCCTCTGAAGAAGGGGCCCTGGACGTCCGCGGAGGACGCCATCCTGGTGGACTACGTGAAGAAGCACGGCG
hpt10         CAGCAGCGGCGGAGGGTCGCCTCACAGGGGCGGCGGGCCGCCTCTGAAGAAGGGGCCCTGGACGTCCGCGGAGGACGCCATCCTGGTGGACTACGTGAAGAAGCACGGCG
hpt14         CAGCAGCGGCGGAGGGTCGCCTCACAGGGGCGGCGGGCCGCCTCTGAAGAAGGGGCCCTGGACGTCGGCGGAGGACGCCATCCTGGTGGACTACGTGAAGAAGCACGGCG
hpt11         CAGCAGCGGCGGAGGGTCGCCTCACAGGGGCGGCGGGCCGCCTCTGAAGAAGGGGCCCTGGACGTCCGCGGAGGACGCCATCCTGGTGGACTACGTGAAGAAGCACGGCG
hpt12         CAGCAGCGGCGGAGGGTCGCCTCACAGGGGCGGCGGGCCGCCTCTGAAGAAGGGGCCCTGGACGTCCGCGGAGGACGCCATCCTGGTGGACTACGTGAAGAAGCACGGCG
hpt13         CAGCAGCGGCGGAGGGTCGCCTCACAGGGGCGGCGGGCCGCCTCTGAAGAAGGGGCCCTGGACGTCGGCGGAGGACGCCATCCTGGTGGACTACGTGAAGAAGCACGGCG
hpt15         CAGCAGCGGCGGAGGGTCGCCTCACAGGGGCGGCGGGCCGCCTCTGAAGAAGGGGCCCTGGACGTCCGCGGAGGACGCCATCCTGGTGGACTACGTGAAGAAGCACGGCG
hpt16         CAGCAGCGGCGGAGGGTCGCCTCACAGGGGCGGCGGGCCGCCTCTGAAGAAGGGGCCCTGGACGTCGGCGGAGGACGCCATCCTGGTGGACTACGTGAAGAAGCACGGCG
hpt17         CAGCAGCGGCGGAGGGTCGCCTCACAGGGGCGGCGGGCCGCCTCTGAAGAAGGGGCCCTGGACGTCCGCGGAGGACGCCATCCTGGTGGACTACGTGAAGAAGCACGGCG
hpt18         CAGCAGCGGCGGAGGGTCGCCTCACAGGGGCGGCGGGCCGCCTCTGAAGAAGGGGCCCTGGACGTCGGCGGAGGACGCCATCCTGGTGGACTACGTGAAGAAGCACGGCG
```

```
                         200       210       220       230       240       250       260       270       280       290       300
                  ....|....|....|....|....|....|....|....|....|....|....|....|....|....|....|....|....|....|....|....|....|....|
HvGAMYB mRNA      aggggaactggaacgcggtgcagaagaacaccgggctgttccggtgcggcaagagctgccgcctccggtgggcgaaccacctcaggcccaacctcaagaagggggccttc
HvGAMYB_CDS       AGGGGAACTGGAACGCGGTGCAGAAGAACACCGGGCTGTTCCGGTGCGGCAAGAGCTGCCGCCTCCGGTGGGCGAACCACCTCAGGCCCAACCTCAAGAAGGGGGCCTTC
hpt01             AGGGGAACTGGAACGCGGTGCAGAAGAACACCGGGCTGTTCCGGTGCGGCAAGAGCTGCCGCCTCCGGTGGGCGAACCACCTCAGGCCCAACCTCAAGAAGGGGGCCTTC
hpt02             AGGGGAACTGGAACGCGGTGCAGAAGAACACCGGGCTGTTCCGGTGCGGCAAGAGCTGCCGCCTCCGGTGGGCGAACCACCTCAGGCCCAACCTCAAGAAGGGGGCCTTC
hpt03             AGGGGAACTGGAACGCGGTGCAGAAGAACACCGGGCTGTTCCGGTGCGGCAAGAGCTGCCGCCTCCGGTGGGCGAACCACCTCAGGCCCAACCTCAAGAAGGGGGCCTTC
hpt04             AGGGGAACTGGAACGCGGTGCAGAAGAACACCGGGCTGTTCCGGTGCGGCAAGAGCTGCCGCCTCCGGTGGGCGAACCACCTCAGGCCCAACCTCAAGAAGGGGGCCTTC
hpt05             AGGGGAACTGGAACGCGGTGCAGAAGAACACCGGGCTGTTCCGGTGCGGCAAGAGCTGCCGCCTCCGGTGGGCGAACCACCTCAGGCCCAACCTCAAGAAGGGGGCCTTC
hpt06             AGGGGAACTGGAACGCGGTGCAGAAGAACACCGGGCTGTTCCGGTGCGGCAAGAGCTGCCGCCTCCGGTGGGCGAACCACCTCAGGCCCAACCTCAAGAAGGGGGCCTTC
hpt07             AGGGGAACTGGAACGCGGTGCAGAAGAACACCGGGCTGTTCCGGTGCGGCAAGAGCTGCCGCCTCCGGTGGGCGAACCACCTCAGGCCCAACCTCAAGAAGGGGGCCTTC
hpt08             AGGGGAACTGGAACGCGGTGCAGAAGAACACCGGGCTGTTCCGGTGCGGCAAGAGCTGCCGCCTCCGGTGGGCGAACCACCTCAGGCCCAACCTCAAGAAGGGGGCCTTC
hpt09             AGGGGAACTGGAACGCGGTGCAGAAGAACACCGGGCTGTTCCGGTGCGGCAAGAGCTGCCGCCTCCGGTGGGCGAACCACCTCAGGCCCAACCTCAAGAAGGGGGCCTTC
hpt10             AGGGGAACTGGAACGCGGTGCAGAAGAACACCGGGCTGTTCCGGTGCGGCAAGAGCTGCCGCCTCCGGTGGGCGAACCACCTCAGGCCCAACCTCAAGAAGGGGGCCTTC
hpt14             AGGGGAACTGGAACGCGGTGCAGAAGAACACCGGGCTGTTCCGGTGCGGCAAGAGCTGCCGCCTCCGGTGGGCGAACCACCTCAGGCCCAACCTCAAGAAGGGGGCCTTC
hpt11             AGGGGAACTGGAACGCGGTGCAGAAGAACACCGGGCTGTTCCGGTGCGGCAAGAGCTGCCGCCTCCGGTGGGCGAACCACCTCAGGCCCAACCTCAAGAAGGGGGCCTTC
hpt12             AGGGGAACTGGAACGCGGTGCAGAAGAACACCGGGCTGTTCCGGTGCGGCAAGAGCTGCCGCCTCCGGTGGGCGAACCACCTCAGGCCCAACCTCAAGAAGGGGGCCTTC
hpt13             AGGGGAACTGGAACGCGGTGCAGAAGAACACCGGGCTGTTCCGGTGCGGCAAGAGCTGCCGCCTCCGGTGGGCGAACCACCTCAGGCCCAACCTCAAGAAGGGGGCCTTC
hpt15             AGGGGAACTGGAACGCGGTGCAGAAGAACACCGGGCTGTTCCGGTGCGGCAAGAGCTGCCGCCTCCGGTGGGCGAACCACCTCAGGCCCAACCTCAAGAAGGGGGCCTTC
hpt16             AGGGGAACTGGAACGCGGTGCAGAAGAACACCGGGCTGTTCCGGTGCGGCAAGAGCTGCCGCCTCCGGTGGGCGAACCACCTCAGGCCCAACCTCAAGAAGGGGGCCTTC
hpt17             AGGGGAACTGGAACGCGGTGCAGAAGAACACCGGGCTGTTCCGGTGCGGCAAGAGCTGCCGCCTCCGGTGGGCGAACCACCTCAGGCCCAACCTCAAGAAGGGGGCCTTC
hpt18             AGGGGAACTGGAACGCGGTGCAGAAGAACACCGGGCTGTTCCGGTGCGGCAAGAGCTGCCGCCTCCGGTGGGCGAACCACCTCAGGCCCAACCTCAAGAAGGGGGCCTTC

                         310       320       330       340       350       360       370       380       390       400       410
                  ....|....|....|....|....|....|....|....|....|....|....|....|....|....|....|....|....|....|....|....|....|....|
HvGAMYB mRNA      acccccgaggaggagaggctcatcatccagctccactccaagatgggcaacaagtgggctcggatggccgctcat-----------------------------------
HvGAMYB_CDS       ACCCCCGAGGAGGAGAGGCTCATCATCCAGCTCCACTCCAAGATGGGCAACAAGTGGGCTCGGATGGCCGCTCAT-----------------------------------
hpt01             ACCCCCGAGGAGGAGAGGCTCATCATCCAGCTCCACTCCAAGATGGGCAACAAGTGGGCTCGGATGGCCGCTCATGTAAGTGCGATGCCACGCTGATTCACTGCTTTACT
hpt02             ACCCCCGAGGAGGAGAGGCTCATCATCCAGCTCCACTCCAAGATGGGCAACAAGTGGGCTCGGATGGCCGCTCATGTAAGTGCGATGCCACGCTGATTCACTGCTTTACT
hpt03             ACCCCCGAGGAGGAGAGGCTCATCATCCAGCTCCACTCCAAGATGGGCAACAAGTGGGCTCGGATGGCCGCTCATGTAAGTGCGATGCCACGCTGATTCACTGCTTTACT
hpt04             ACCCCCGAGGAGGAGAGGCTCATCATCCAGCTCCACTCCAAGATGGGCAACAAGTGGGCTCGGATGGCCGCTCATGTAAGTGCGATGCCACGCTGATTCACTGCTTTACT
hpt05             ACCCCCGAGGAGGAGAGGCTCATCATCCAGCTCCACTCCAAGATGGGCAACAAGTGGGCTCGGATGGCCGCTCATGTAAGTGCGATGCCACGCTGATTCACTGCTTTACT
hpt06             ACCCCCGAGGAGGAGAGGCTCATCATCCAGCTCCACTCCAAGATGGGCAACAAGTGGGCTCGGATGGCCGCTCATGTAAGTGCGATGCCACGCTGATTCACTGCTTTACT
hpt07             ACCCCCGAGGAGGAGAGGCTCATCATCCAGCTCCACTCCAAGATGGGCAACAAGTGGGCTCGGATGGCCGCTCATGTAAGTGCGATGCCACGCTGATTCACTGCTTTACT
hpt08             ACCCCCGAGGAGGAGAGGCTCATCATCCAGCTCCACTCCAAGATGGGCAACAAGTGGGCTCGGATGGCCGCTCATGTAAGTGCGATGCCACGCTGATTCACTGCTTTACT
hpt09             ACCCCCGAGGAGGAGAGGCTCATCATCCAGCTCCACTCCAAGATGGGCAACAAGTGGGCTCGGATGGCCGCTCATGTAAGTGCGATGCCACGCTGATTCACTGCTTTACT
hpt10             ACCCCCGAGGAGGAGAGGCTCATCATCCAGCTCCACTCCAAGATGGGCAACAAGTGGGCTCGGATGGCCGCTCATGTAAGTGCGATGCCACGCTGATTCACTGCTTTACT
hpt14             ACCCCCGAGGAGGAGAGGCTCATCATCCAGCTCCACTCCAAGATGGGCAACAAGTGGGCTCGGATGGCCGCTCATGTAAGTGCGATGCCACGCTGATTCACTGCTTTACT
hpt11             ACCCCCGAGGAGGAGAGGCTCATCATCCAGCTCCACTCCAAGATGGGCAACAAGTGGGCTCGGATGGCCGCTCATGTAAGTGCGATGCCACGCTGATTCACTGCTTTACT
hpt12             ACCCCCGAGGAGGAGAGGCTCATCATCCAGCTCCACTCCAAGATGGGCAACAAGTGGGCTCGGATGGCCGCTCATGTAAGTGCGATGCCACGCTGATTCACTGCTTTACT
hpt13             ACCCCCGAGGAGGAGAGGCTCATCATCCAGCTCCACTCCAAGATGGGCAACAAGTGGGCTCGGATGGCCGCTCATGTAAGTGCGATGCCACGCTGATTCACTGCTTTACT
hpt15             ACCCCCGAGGAGGAGAGGCTCATCATCCAGCTCCACTCCAAGATGGGCAACAAGTGGGCTCGGATGGCCGCTCATGTAAGTGCGATGCCACGCTGATTCACTGCTTTACT
hpt16             ACCCCCGAGGAGGAGAGGCTCATCATCCAGCTCCACTCCAAGATGGGCAACAAGTGGGCTCGGATGGCCGCTCATGTAAGTGCGATGCCACGCTGATTCACTGCTTTACT
hpt17             ACCCCCGAGGAGGAGAGGCTCATCATCCAGCTCCACTCCAAGATGGGCAACAAGTGGGCTCGGATGGCCGCTCATGTAAGTGCGATGCCACGCTGATTCACTGCTTTACT
hpt18             ACCCCCGAGGAGGAGAGGCTCATCATCCAGCTCCACTCCAAGATGGGCAACAAGTGGGCTCGGATGGCCGCTCATGTAAGTGCGATGCCACGCTGATTCACTGCTTTACT
```

```
                   420       430       440       450       460       470       480       490       500       510       520
                ....|....|....|....|....|....|....|....|....|....|....|....|....|....|....|....|....|....|....|....|....|....|
HvGAMYB mRNA    --------------------------------------------------------------------------------------------------------------
HvGAMYB_CDS     --------------------------------------------------------------------------------------------------------------
hpt01           CCATGTACTAGTACTAGATGTTTACCAGTAGCAAGTCATGCTCTTTGTGTGCTGTTCTTGAGCCTTGCATGATTTCCTCTTACGTCATGGTCACATGGGGTTGAAAGCTA
hpt02           CCATGTACTAGTACTAGATGTTTACCAGTAGCAAGTCATGCTCTTTGTGTGCTGTTCTTGAGCCTTGCATGATTTCCTCTTACGTCATGGTCACATGGGGTTGAAAGCTA
hpt03           CCATGTACTAGTACTAGATGTTTACCAGTAGCAAGTCATGCTCTTTGTGTGCTGTTCTTGAGCCTTGCATGATTTCCTCTTACGTCATGGTCACATGGGGTTGAAAGCTA
hpt04           CCATGTACTAGTACTAGATGTTTACCAGTAGCAAGTCATGCTCTTTGTGTGCTGTTCTTGAGCCTTGCATGATTTCCTCTTACGTCATGGTCACATGGGGTTGAAAGCTA
hpt05           CCATGTACTAGTACTAGATGTTTACCAGTAGCAAGTCATGCTCTTTGTGTGCTGTTCTTGAGCCTTGCATGATTTCCTCTTACGTCATGGTCACATGGGGTTGAAAGCTA
hpt06           CCATGTACTAGTACTAGATGTTTACCAGTAGCAAGTCATGCTCTTTGTGTGCTGTTCTTGAGCCTTGCATGATTTCCTCTTACGTCATGGTCACATGGGGTTGAAAGCTA
hpt07           CCATGTACTAGTACTAGATGTTTACCAGTAGCAAGTCATGCTCTTTGTGTGCTGTTCTTGAGCCTTGCATGATTTCCTCTTACGTCATGGTCACATGGG-TTGAAAGCTA
hpt08           CCATGTACTAGTACTAGATGTTTACCAGTAGCAAGTCATGCTCTTTGTGTGCTGTTCTTGAGCCTTGCATGATTTCCTCTTACGTCATGGTCACATGGGGTTGAAAGCTA
hpt09           CCATGTACTAGTACTAGATGTTTACCAGTAGCAAGTCATGCTCTTTGTGTGCTGTTCTTGAGCCTTGCATGATTTCCTCTTACGTCATGGTCACATGGGGTTGAAAGCTA
hpt10           CCATGTACTAGTACTAGATGTTTACCAGTAGCAAGTCATGCTCTTTGTGTGCTGTTCTTGAGCCTTGCATGATTTCCTCTTACGTCATGGTCACATGGGGTTGAAAGCTA
hpt14           CCATGTACTAGTACTAGATGTTTACCAGTAGCAAGTCATGCTCTTTGTGTGCTGTTCTTGAGCCTTGCATGATTTCCTCTTACGTCATGGTCACATGGGGTTGAAAGCTA
hpt11           CCATGTACTAGTACTAGATGTTTACCAGTAGCAAGTCATGCTCTTTGTGTGCTGTTCTTGAGCCTTGCATGATTTCCTCTTACGTCATGGTCACATGGGGTTGAAAGCTA
hpt12           CCATGTACTAGTACTAGATGTTTACCAGTAGCAAGTCATGCTCTTTGTGTGCTGTTCTTGAGCCTTGCATGATTTCCTCTTACGTCATGGTCACATGGGGTTGAAAGCTA
hpt13           CCATGTACTAGTACTAGATGTTTACCAGTAGCAAGTCATGCTCTTTGTGTGCTGTTCTTGAGCCTTGCATGATTTCCTCTTACGTCATGGTCACATGGGGTTGAAAGCTA
hpt15           CCATGTACTAGTACTAGATGTTTACCAGTAGCAAGTCATGCTCTTTGTGTGCTGTTCTTGAGCCTTGCATGATTTCCTCTTACGTCATGGTCACATGGGGTTGAAAGCTA
hpt16           CCATGTACTAGTACTAGATGTTTACCAGTAGCAAGTCATGCTCTTTGTGTGCTGTTCTTGAGCCTTGCATGATTTCCTCTTACGTCATGGTCACATGGGGTTGAAAGCTA
hpt17           CCATGTACTAGTACTAGATGTTTACCAGTAGCAAGTCATGCTCTTTGTGTGCTGTTCTTGAGCCTTGCATGATTTCCTCTTACGTCATGGTCACATGGGGTTGAAAGCTA
hpt18           CCATGTACTAGTACTAGATGTTTACCAGTAGCAAGTCATGCTCTTTGTGTGCTGTTCTTGAGCCTTGCATGATTTCCTCTTACGTCATGGTCACATGGGGTTGAAAGCTA


                   530       540       550       560       570       580       590       600       610       620       630
                ....|....|....|....|....|....|....|....|....|....|....|....|....|....|....|....|....|....|....|....|....|....|
HvGAMYB mRNA    --------------------------------------------------------------------------------------------------------------
HvGAMYB_CDS     --------------------------------------------------------------------------------------------------------------
hpt01           CCGATTTCTATAGATTTATTATTGTCCTCAAGACTCTCTATTTTTTTTT--ATGTAGTAGAGTACGTAGTAATGAATTGTTCTGTTTGATTTATACTGTATGTTGGAACT
hpt02           CCGATTTCTATAGATTTATTATTGTCCTGAAGACTCTCTATTTTTTTTT--ATGTAGTAGAGTACGTAGTAATGAATTGTTCTGTTTGATTTATACTGTATGTTGGAACT
hpt03           CCGATTTCTATAGATTTATTATTGTCCTGAAGACTCTCTATTTTTTTT---ATGTAGTAGAGTACGTAGTAATGAATTGTTCTGTTTGATTTATACTGTATGTTGGAACT
hpt04           CCGATTTCTATAGATTTATTATTGTCCTGAAGACTCTCTATTTTTTTTTTTATGTAGTAGAGTACGTAGTAATGAATTGTTCTGTTTGATTTATACTGTATGTTGGAACT
hpt05           CCGATTTCTATAGATTTATTATTGTCCTCAAGACTCTCTATTTTTTTT---ATGTAGTAGAGTACGTAGTAATGAATTGTTCTGTTTGATTTATACTGTATGTTGGAACT
hpt06           CCGATTTCTATAGATTTATTATTGTCCTGAAGACTCTCTATTTTTTTTT--ATGTAGTAGAGTACGTAGTAATGAATTGTTCTGTTTGATTTATACTGTATGTTGGAACT
hpt07           CCGATTTCTATAGATTTATTATTGTCCTGAAGACTCTCTATTTTTTTTT--ATGTAGTAGAGTACGTAGTAATGAATTGTTCTGTTTGATTTATACTGTATGTTGGAACT
hpt08           CCGATTTCTATAGATTTATTATTGTCCTGAAGACTCTCTATTTTTTTTTT-ATGTAGTAGAGTACGTAGTAATGAATTGTTCTGTTTGATTTATACTGTATGTTGGAACT
hpt09           CCGATTTCTATAGATTTATTATTGTCCTGAAGACTCTCTATTTTTTTTT--ATGTAGTAGAGTACGTAGTAATGAATTGTTCTGTTTGATTTATACTGTATGTTGGAACT
hpt10           CCGATTTCTATAGATTTATTATTGTCCTGAAGACTCTCTATTTTTTTTTTTATGTAGTAGAGTACGTAGTAATGAATTGTTCTGTTTGATTTATACTGTATGTTGGAACT
hpt14           CCGATTTCTATAGATTTATTATTGTCCTGAAGACTCTCTATTTTTTTTT--ATGTAGTAGAGTACGTAGTAATGAATTGTTCTGTTTGATTTATACTGTATGTTGGAACT
hpt11           CCGATTTCTATAGATTTATTATTGTCCTGAAGACTCTCTATTTTTTTTTT-ATGTAGTANAGTACATAGTAATGAATTGTTCTGTTTGATTTATACTGTATGTTGGAACT
hpt12           CCGATTTCTATAGATTTATTATTGTCCTGAAGACTCTCTATTTTTTTTTTTATGTAGTAGAGTACGTAGTAATGAATTGTTCTGTTTGATTTATACTGTATGTTGGAACT
hpt13           CCGATTTCTATAGATTTATTATTGTCCTCAAGACTCTCTATTTTTTTTT--ATGTAGTAGAGTACGTAGTAATGAATTGTTCTGTTTGATTTATACTGTATGTTGGAACT
hpt15           CCGATTTCTATAGATTTATTATTGTCCTGAAGACTCTCTATTTTTTTTT--ATGTAGTAGAGTACGTAGTAATGAATTGTTCTGTTTGATTTATACTGTATGTTGGAACT
hpt16           CCGATTTCTATAGATTTATTATTGTCCTGAAGACTCTCTATTTTTTTTT--ATGTAGTAGAGTACGTAGTAATGAATTGTTCTGTTTGATTTATACTGTATGTTGGAACT
hpt17           CCGATTTCTATAGATTTATTATTGTCCTGAAGACTCTCTATTTTTTTTT--ATGTAGTAGAGTACGTAGTAATGAATTGTTCTGTTTGATTTATACTGTATGTTGGAACT
hpt18           CCGATTTCTATAGATTTATTATTGTCCTGAAGACTCTCTATTTTTTTTT--ATGTAGTAGAGTACGTAGTAATGAATTGTTCTGTTTGATTTATACTGTATGTTGGAACT
```

```
                       640       650       660       670       680       690       700       710       720       730       740
                ....|....|....|....|....|....|....|....|....|....|....|....|....|....|....|....|....|....|....|....|....|....|
HvGAMYB mRNA    --------------------------------------------------------------------------------------------------------------
HvGAMYB_CDS     --------------------------------------------------------------------------------------------------------------
hpt01           GTGTTAATATTTGTTCCACTTCCTGTTGCTTACTGTTGT---TGTTGTTGTTCTGGACGAAAGATTAACTTAGATGATTATCTACTAGTGCTTTTCAGAAAGTAATAATC
hpt02           GTGTTAATATTTGTTCCACTTCCTGTTGCTTACTGTTGT---TGTTGTTGTTCTGGACGAAAGATTAACTTAGATGATTATCTACTAGTGCTTTTCAGAAAGTAATAATC
hpt03           GTGTTAATATTTGTTCCCCTTCCTGTTGCTTACTGTTGTGTTTGTTGTTGTTCTGGACGAAAGATTAACTTAGATGATTATCTACTAGTGCTTTTCAGAAAGTAATAATC
hpt04           GTGTTAATATTTGTTCCACTTCCTGTTGCTTACTGTTGT---TGTTGTTGTTCTGGACGAAAGATTAACTTAGATGATTATCTACTAGTGCTTTTCAGAAAGTAATAATC
hpt05           GTGTTAATATTTGTTCCACTTCCTGTTGCTTACTGTTGT---TGTTGTTGTTCTGGACGAAAGATTAACTTAGATGATTATCTGCTAGTGCTTTTCAGAAAGTAATAATC
hpt06           GTGTTAATATTTGTTCCACTTCCTGTTGCTTACTGTTGT---TGTTGTTGTTCTGGACGAAAGATTAACTTAGATCATTAT---CTAGTGCTTTTCAGAAAGTAATAATC
hpt07           GTGTTAATATTTGTTCCACTTCCTGTTGCTTACTGTTGT---TGTTGTTGTTCTGGACGAAAGATTAACTTAGATGATTATCTACTAGTGCTTTTCAGAAAGTAATAATC
hpt08           GTGTTAATATTTGTTCCACTTCCTGTTGCTTACTGTTGT---TGTTGTTGTTCTGGACGAAAGATTAACTTAGATGATTATCTACTAGTGCTTTTCAGAAAGTAATAATC
hpt09           GTGTTAATATTTGTTCCACTTCCTGTTGCTTACTGTTGT---TGTTGTTGTTCTGGACGAAAGATTAACTTAGATGATTATCTACTAGTGCTTTTCAGAAAGTAATAATC
hpt10           GTGTTAATATTTGTTCCACTTCCTGTTGCTTACTGTTGT---TGTTGTTGTTCTGGACGAAAGATTAACTTAGATGATTATCTACTAGTGCTTTTCAGAAAGTAATAATC
hpt14           GTGTTAATATTTGTTCCACTTCCTGTTGCTTACTGTTGT---TGTTGTTGTTCTGGACGAAAGATTAACTTAGATGATTATCTACTAGTGCTTTTCAGAAAGTAATAATC
hpt11           GTGTTAATATTTGTTCCACTTCCTGTTGCTTACTGTTGT---TGTTGTTGTTCTGGACGAAAGATTAACTTANATGATTATCTACTAGTGCTTTTCAGAAAGTAATAATC
hpt12           GTGTTAATATTTGTTCCACTTCCTGTTGCTTACTGTTGT---TGTTGTTGTTCTGGACGAAAGATTAACTTAAATGATTATCTACTAGTGCTTTTCAGAAAGTAATAATC
hpt13           GTGTTAATATTTGTTCCACTTCCTGTTGCTTACTGTTGT---TGTTGTTGTTCTGGACGAAAGATTAACTTAGATGATTATCTACTAGTGCTTTTCAGAAAGTAATAATC
hpt15           GTGTTAATATTTGTTCCACTTCCTGTTGCTTACTGTTGT---TGTTGTTGTTCTGGACGAAAGATTAACTTAGATGATTATCTACTAGTGCTTTTCAGAAAGTAATAATC
hpt16           GTGTTAATATTTGTTCCACTTCCTGTTGCTTACTGTTGT---TGTTGTTGTTCTGGACGAAAGATTAACTTAGATGATTATCTACTAGTGCTTTTCAGAAAGTAATAATC
hpt17           GTGTTAATATTTGTTCCACTTCCTGTTGCTTACTGTTGT---TGTTGTTGTTCTGGACGAAAGATTAACTTAGATCATTAT---CTAGTGCTTTTCAGAAAGTAATAATC
hpt18           GTGTTAATATTTGTTCCACTTCCTGTTGCTTACTGTTGT---TGTTGTTGTTCTGGACGAAAGATTAACTTAGATGATTATCTACTAGTGCTTTTCAGAAAGTAATAATC

                       750       760       770       780       790       800       810       820       830       840       850
                ....|....|....|....|....|....|....|....|....|....|....|....|....|....|....|....|....|....|....|....|....|....|
HvGAMYB mRNA    --------------------------------------------------------------------------------------------------------------
HvGAMYB_CDS     --------------------------------------------------------------------------------------------------------------
hpt01           AAACTAAAGCTTTAAGCTGGCACATAGTACTGTGTTCAGCTCTGCTATTCTGGTTACTGAAAGCTAGTTGGCCTGACTTCTAAAAGGTTAAAAATTGACGGCTACATATG
hpt02           AAACTAAAGCTTTAAGCTGGCACATAGTACTGTGTTCAGCTCTGCTATTCTTGTTACTGAAAGCTAGTTGGCCTGACTTCTAAAAGGTTAAAAATTGACGGCTACATACG
hpt03           AAACTAAAGCTTTAAGCTGGCACATAGTACTGTGTTCAGCTCTGCTATTCTTGTTACTGAAAGCTAGTTGGCCTGACTTCTAAAAGGTTAAAAATTGACGGCTACATATG
hpt04           AAACTAAAGCTTTAAGCTGGCACATAGTAATGTGTTCAGCTCTGCTATTCTTGTTACTGAAAGCTAGTTGGCCTGACTTCTAAAAGGTTAAAAATTGACGGCTACATATG
hpt05           AAACTAAAGCTTTAAGCTGGCACATAGTACTGTGTTCAGCTCTGCTATTCTTGTTACTGAAAGCTAGTTGGCCCGACTTCTAAAAGGTTAAAAATTGACGGCTACATATG
hpt06           AAACTAAAGCTTTAAGCTGGCACATAGTACTGTGTTCAGCTCTGCTATTCTTGTTACTGAAAGCTAGTTGGCCTGACTTCTAAAAGGTTAAAAATTGACGGCTACATATG
hpt07           AAACTAAAGCTTTAAGCTGGCACATAGTACTGTGTTCAGCTCTGCTATTCTTGTTACTGAAAGCTAGTTGGCCTGACTTCTAAAAGGTTAAAAATTGACGGCTACATATG
hpt08           AAACTAAAGCTTTAAGCTGGCACATAGTACTGTGTTCAGCTCTGCTATTCTTGTTACTGAAAGCTAGTTGGCCTGACTTCTAAAAGGTTAAAAATTGACGGCTACATATG
hpt09           AAACTAAAGCTTTAAGCTGGCACATAGTACTGTGTTCAGCTCTGCTATTCTTGTTACTGAAAGCTAGTTGGCCTGACTTCTAAAAGGTTAAAAATTGACGGCTACATACG
hpt10           AAACTAAAGCTTTAAGATGGCACATAGTAATGTGTTCAGCTCTGCTATTCTTGTTACTGAAAGCTAGTTGGCCTGACTTCTAAAAGGTTAAAAATTGACGGCTACATATG
hpt14           AAACTAAAGCTTTAAGCTGGCACATAGTACTGTGTTCAGCTCTGCTATTCTTGTTACTGAAAGCTAGTTGGCCTGACTTCTAAAAGGTTAAAAATTGACGGCTACATATG
hpt11           AAACTAAAGCTTTAAGCTGGCACATAGTACTGTGTTCAGCTCTGCTATTCTTGTTACTGAAAGCTAGTTGGCCTGACTTCTAAAAGGTTAAAAATTGACGGCTACATATG
hpt12           AAACTAAAGCTTTAAGCTGGCACATAGTAATGTGTTCAGCTCTGCTATTCTTGTTACTGAAAGCTAGTTGGCCTGACTTCTAAAAGGTTAAAAATTGACGGCTACATATG
hpt13           AAACTAAAGCTTTAAGCTGGCACATAGTACTGTGTTCAGCTCTGCTATTCTGGTTACTGAAAGCTAGTTGGCCTGACTTCTAAAAGGTTAAAAATTGACGGCTACATATG
hpt15           AAACTAAAGCTTTAAGCTGGCACATAGTACTGTGTTCAGCTCTGCTATTCTTGTTACTGAAAGCTAGTTGGCCTGACTTCTAAAAGGTTAAAAATTGACGGCTACATACG
hpt16           AAACTAAAGCTTTAAGCTGGCACATAGTACTGTGTTCAGCTCTGCTATTCTTGTTACTGAAAGCTAGTTGGCCTGACTTCTAAAAGGTTAAAAATTGACGGCTACATATG
hpt17           AAACTAAAGCTTTAAGCTGGCACATAGTACTGTGTTCAGCTCTGCTATTCTTGTTACTGAAAGCTAGTTGGCCTGACTTCTAAAAGGTTAAAAATTGACGGCTACATATG
hpt18           AAACTAAAGCTTTAAGCTGGCACATAGTACTGTGTTCAGCTCTGCTATTCTTGTTACTAAAAGCTAGTTGGCCTGACTTCTAAAAGGTTAAAAATTGACGGCTACATACG
```

```
                      860       870       880       890       900       910       920       930       940       950       960
             ....|....|....|....|....|....|....|....|....|....|....|....|....|....|....|....|....|....|....|....|....|....|
HvGAMYB mRNA ------------------------------------------------------------------------------------------------------------
HvGAMYB_CDS  ------------------------------------------------------------------------------------------------------------
hpt01        TATGCCTTAAATAGGTAGTCCATTTTTGACCTTGACTGCCATCAAGGGGTCGCTTGCTACTCTGGCATCTATGTTGACAAATTGACTATTAGTATTAGCTAGGGGCTATT
hpt02        TATGCCTTAAATAGGTAGTCCATTTTTGACCTTGACTGCCATCAAGGGGTCGCTTGCTACTCTGGCATCTATGTTGACAAATTGACTATTAGTATTAGCTAGGGGCTATT
hpt03        TATGCCTTAAATAGGTAGTCCATTTTTGACCTTGACTGCCATCAAGGGGTCGCTTGCTACTCTGGCATCTATGTTGACAAATTGACTATTAGTATTAGCTAGGGGCTATT
hpt04        TATGCCTTAAATAGGTAGTCCATTTTTGACCTTGACTGCCATCAAGGGGTCGCTTGCTACTCTGGCATCTATGTTGACAAATTGACTATTAGTATTAGCTAGGGGCTATT
hpt05        TATGCCTTAAATAGGTAGTCCATTTTTGACCTTGACTGCCATCAAAGGGTCGCTTGCTACTCTGGCATCTATGTTGACAAATTGACTATTAGTATTAGCTAGGGGCTATT
hpt06        TATGCCTTAAATAGGTAGCCCATTTTTGACCTTGACTGCCATCAAGGGGTCGCTTGCTACTCTGGCATCTATGTTGACAAATTGACTATTAGTATTAGCTAGGGGCTATT
hpt07        TATGCCTTAAATAGGTAGTCCATTTTTGACCTTGACTGCCATCAAGGGGTCGCTTGCTACTCTGGCATCTATGTTGACAAATTGACTATTAGTATTAGCTAGGGGCTATT
hpt08        TATGCCTTAAATAGGTAGTCCATTTTTGACCTTGACTGCCATCAAGGGGTCGCTTGCTACTCTGGCATCTATGTTGACAAATTGACTATTAGTATTAGCTAGGGGCTATT
hpt09        TATGCCTTAAATAGGTAGTCCATTTTTGACCTTGACTGCCATCAAGGGGTCGCTTGCTACTCTGGCATCTATGTTGACAAATTGACTATTAGTATTAGCTAGGGGCTATT
hpt10        TATGCCTTAAATAGGTAGTCCATTTTTGACCTTGACTGCCATCAAGGGGTCGCTTGCTACTCTGGCATCTATGTTGACAAATTGACTATTAGTATTAGCTAGGGGCTATT
hpt14        TATGCCTTAAATAGGTAGTCCATTTTTGACCTTGACTGCCATCAAGGGGTCGCTTGCTACTCTGGCATCTATGTTGACAAATTGACTATTAGTATTAGCTAGGGGCTATT
hpt11        TATGCCTTAAATAGGTAGTCCATTTTTGACCTTGACTGCCATCAAGGGGTCGCTTGCTACTCTGGCATCTATGTTGACAAATTGACTATTAGTATTAGCTAGGGGCTATT
hpt12        TATGCCTTAAATAGGTAGTCCATTTTTGACCTTGACTGCCATCAAGGGGTCGCTTGCTACTCTGGCATCTATGTTGACAAATTGACTATTAGTATTAGCTAGGGGCTATT
hpt13        TATGCCTTAAATAGGTAGTCCATTTTTGACCTTGACTGCCATCAAGGGGTCGCTTGCTACTCTGGCATCTATGTTGACAAATTGACTATTAGTATTAGCTAGGGGCTATT
hpt15        TATGCCTTAAATAGGTAGTCCATTTTTGACCTTGACTGCCATCAAGGGGTCGCTTGCTACTCTGGCATCTATGTTGACAAATTGACTATTAGTATTAGCTAGGGGCTATT
hpt16        TATGCCTTAAATAGGTAGTCCATTTTTGACCTTGACTGCCATCAAGGGGTCGCTTGCTACTCTGGCATCTATGTTGACAAATTGACTATTAGTATTAGCTAGGGGCTATT
hpt17        TATGCCTTAAATAGGTAGCCCATTTTTGACCTTGACTGCCATCAAGGGGTCGCTTGCTACTCTGGCATCTATGTTGACAAATTGACTATTAGTATTAGCTAGGGGCTATT
hpt18        TATGCCTTAAATAGGTAGTCCATTTTTGACCTTGACTGCCATCAAGGGGTCGCTTGCTACTCTGGCATCTATGTTGACAAATTGACTATTAGTATTAGCTAGGGGCTATT

                      970       980       990      1000      1010      1020      1030      1040      1050      1060      1070
             ....|....|....|....|....|....|....|....|....|....|....|....|....|....|....|....|....|....|....|....|....|....|
HvGAMYB mRNA ------------------------------------------------------------------------------------------------------------
HvGAMYB_CDS  ------------------------------------------------------------------------------------------------------------
hpt01        TCAGTGTGACCAAGTAGTGTTGTTTTTGTTGACAATCTAGAGCTACTAATCCTGTATGGAATATTATTATGACTCACAGTATTTAGTTTTTTTTT-AATATCATTTAGCA
hpt02        TCAGTGTGACCAAGTAGTGTTGTTTTTGTTGACAATCTAGAGCTACTAATCCTGTATGGAATATTATTATGACTCACAGTATTTAGTTTTTTTTTT-ATATCATTTAGCA
hpt03        TCAGTGTGACCAAGTAGTGTTGTTTTTGTTGACAATCTAGAGCTACTAATCCTGTATGGAATATTATTATGACTCACAGTATTTAGTTTTTTTTT-AATATCATTTAGCA
hpt04        TCAGTGTGACCAAGTAGTGTTGTTTTTGTTGACAATCTAGAGCTACTAATCCTGTATGGAATATTATTATGACTCACAGTATTTAGTTTTTTTT--AATATCATTTAGCA
hpt05        TCAGTGTGACCAAGTAGTGTTGTTTTTGTTGACAATCTAGAGCTACTAATCCTGTATGGAATATTATTATGACTCACAGTATTTAGTTTTTTTTT-AATATCATTTAGCA
hpt06        TCAGTGTGACCAAGTAGTGTTGTTTTTGTTGACAATCTAGAGCTACTAATCCTGTATGGAATATTATTATGACTCACAGTATTTAGTTTTTTTTTTAATATCATTTAGCA
hpt07        TCAGTGTGACCAAGTAGTGTTGTTTTTGTTGACAATCTAGAGCTACTAATCCTGTATGGAATATTATTATGACTCACAGTATTTAGTTTTTTTTT--ATATCATTTAGCA
hpt08        TCAGTGTGACCAAGTAGTGTTGTTTTTGTTGACAATCTAGAGCTACTAATCCTGTATGGAATATTATTATGACTCACAGTATTTAGTTTTTTTTTT-ATATCATTTAGCA
hpt09        TCAGTGTGACCAAGTAGTGTTGTTTTTGTTGACAATCTAGAGCTACTAATCCTGTATGGAATATTATTATGACTCACAGTATTTAGTTTTTTTTTT-ATATCATTTAGCA
hpt10        TCAGTGTGACCAAGTAGTGTTGTTTTTGTTGACAATCTAGAGCTACTAATCCTGTATGGAATATTATTATGACTCACAGTATTTAGTTTTTTTT--AATATCATTTAGCA
hpt14        TCAGTGTGACCAAGTAGTGTTGTTTTTGTTGACAATCTAGAGCTACTAATCCTGTATGGAATATTATTATGACTCACAGTATTTAGTTTTTTTTTTAATATCATTTAGCA
hpt11        TCAGTGTGACCAAGTAGTGTTGTTTTTGTTGACAATCTAGAGCTACTAATCCTGTATGGAATATTATTATGACTCACAGTATTTAGTTTTTTTTTT-ATATCATTTAGCA
hpt12        TCAGTGTGACCAAGTAGTGTTGTTTTTGTTGACAATCTAGAGCTACTAATCCTGTATGGAATATTATTATGACTCACAGTATTTAGTTTTTTTT--AATATCATTTAGCA
hpt13        TCAGTGTGACCAAGTAGTGTTGTTTTTGTTGACAATCTAGAGCTACTAATCCTGTATGGAATATTATTATGACTCACAGTATTTAGTTTTTTTTT-AATATCATTTAGCA
hpt15        TCAGTGTGACCAAGTAGTGTTGTTTTTGTTGACAATCTAGAGCTACTAATCCTGTATGGAATATTATTATGACTCACAGTATTTAGTTTTTTTTTT-ATATCATTTAGCA
hpt16        TCAGTGTGACCAAGTAGTGTTGTTTTTGTTGACAATCTAGAGCTACTAATCCTGTATGGAATATTATTATGACTCACAGTATTTAGTTTTTTTTTTAATATCATTTAGCA
hpt17        TCAGTGTGACCAAGTAGTGTTGTTTTTGTTGACAATCTAGAGCTACTAATCCTGTATGGAATATTATTATGACTCACAGTATTTAGTTTTTTTTTTAATATCATTTAGCA
hpt18        TCAGTGTGACCAAGTAGTGTTGTTTTTGTTGACAATCTAGAGCTACTAATCCTGTATGGAATATTATTATGACTCACAGTATTTAGTTTTTTTTTT-ATATCATTTAGCA
```

```
                   1080      1090      1100      1110      1120      1130      1140      1150      1160      1170      1180
          ....|....|....|....|....|....|....|....|....|....|....|....|....|....|....|....|....|....|....|....|....|....|
HvGAMYB mRNA   --------------------------------------------------------------------------------------------------------------
HvGAMYB_CDS    --------------------------------------------------------------------------------------------------------------
hpt01          TGTACTCCCTCTTTACCTAGTTCTCCTCAACTATAATTATTTAGGTAGAGAAGAAGTACTTGCTACAATTTGGAAATAAACTCTACAATTGATCCATGTTTCGTTCATAC
hpt02          TGTACTCCCTCTTTACCTAGTTCTCCTCAACTATAATTATTTAGGTAGAGAGTAAGTACTTGCTACAATTTGGAAATAAACCCTACAATTGATCCATGTTTCGTTCATAC
hpt03          TGTACTCCCTCTTTACCTAGTTCTCCTCAACTATAATTATTTAGGTAGAGAAGAAGTACTTGCTACAATTTGGAAATAAACTCTACAATTGATCCATGTTTCGTTCATAC
hpt04          TGTACTCCCTCTTTACCTAGTTCTCCTCAACTATAATTATTTAGGTAGAGAGGAAGTACTTGCTACAATTTGGAAATAAACCCTACAATTGATCCATGTTTCGTTCATAC
hpt05          TGTACTCCCTCTTTACCTAGTTCTCCTCAACTATAATTATTTAGGTAGAGAGGAAGTACTTGCTACAATTTGGAAATAAACCCTACAATTGATCCATGTTTCGTTCATAC
hpt06          TGTACTCCCTCTTTACCTAGTTCTCCTCAACTATAATTATTTAGGTAGAGAAGAAGTACTTGCTACAATTTGGAAATAAACTCTACAATTGATCCATGTTTCGTTCATAC
hpt07          TGTACTCCCTCTTTACCTAGTTCTCCTCAACTATAATTATTTAGGTAGAGAGTAAGTACTTGCTACAATTTGGAAATAAACCCTACAATTGATCCATGTTTCGTTCATAC
hpt08          TGTACTCCCTCTTTACCTAGTTCTCCTCAACTATAATTATTTAGGTAGAGAGGAAGTACTTGCTACAATTTGGAAATAAACCCTACAATTGATCCATGTTTCGTTCATAC
hpt09          TGTACTCCCTCTTTACCTAGTTCTCCTCAACTATAATTATTTAGGTAGAGAGTAAGTACTTGCTACAATTTGGAAATAAACCCTACAATTGATCCATGTTTCGTTCATAC
hpt10          TGTACTCCCTCTTTACCTAGTTCTCCTCAACTATAATTATTTAGGTAGAGAGGAAGTACTTGCTACAATTTGGAAATAAACCCTACAATTGATCCATGTTTCGTTCATAC
hpt14          TGTACTCCCTCTTTACCTAGTTCTCCTCAACTATAATTATTTAGGTAGAGAAGAAGTACTTGCTACAATTTGGAAATAAACTCTACAATTGATCCATGTTTCGTTCATAC
hpt11          TGTACTCCCTCTTTACCTAGTTCTCCTCAACTATAATTATTTAGGTAGAGAGGAAGTACTTGCTACAATTTGGAAATAAACCCTACAATTGATCCATGTTTCGTTCATAC
hpt12          TGTACTCCCTCTTTACCTAGTTCTCCTCAACTATAATTATTTAGGTAGAGAGGAAGTACTTGCTACAATTTGGAAATAAACCCTACAATTGATCCATGTTTCGTTCATAC
hpt13          TGTACTCCCTCTTTACCTAGTTCTCCTCAACTATAATTATTTAGGTAGAGAAGAAGTACTTGCTACAATTTGGAAATAAACTCTACAATTGATCCATGTTTCGTTCATAC
hpt15          TGTACTCCCTCTTTACCTAGTTCTCCTCAACTATAATTATTTAGGTAGAGAGTAAGTACTTGCTACAATTTGGAAATAAACCCTACAATTGATCCATGTTTCGTTCATAC
hpt16          TGTACTCCCTCTTTACCTAGTTCTCCTCAACTATAATTATTTAGGTAGAGAAGAAGTACTTGCTACAATTTGGAAATAAACTCTACAATTGATCCATGTTTCGTTCATAC
hpt17          TGTACTCCCTCTTTACCTAGTTCTCCTCAACTATAATTATTTAGGTAGAGAAGAAGTACTTGCTACAATTTGGAAATAAACTCTACAATTGATCCATGTTTCGTTCATAC
hpt18          TGTACTCCCTCTTTACCTAGTTCTCCTCAACTATAATTATTTAGGTAGAGAGTAAGTACTTGCTACAATTTGGAAATAAACCCTACAATTGATCCATGTTTCGTTCATAC

                   1190      1200      1210      1220      1230      1240      1250      1260      1270      1280      1290
          ....|....|....|....|....|....|....|....|....|....|....|....|....|....|....|....|....|....|....|....|....|....|
HvGAMYB mRNA   --------------------------------------------------------------------------------------------------------------
HvGAMYB_CDS    --------------------------------------------------------------------------------------------------------------
hpt01          CGATTCCATTGAAAAATAGGTTATCATGTCCTGTATTAATTTATCTTGTGTATATGATTCTTGTATTTTTTTTCCCACCTCTTGTTGTTATTATTGACTTTGAGCACCTC
hpt02          CGATTCCATTGAAAAATAGGTTATCATGTCCTGTATTAATTTATCCTGTGTATATGATTCTTGTATTTTTTT-CCCACCTCTTGTTGTTATTATTGACTTTGAGCACCTC
hpt03          CGATTCCATTGAAAAGTAGGTTATCATGTCCTGTATTAATTTATCTTGTGTATATGATTCTTGTATTTTTTTTCCCACCTCTTGTTGTTATTATTGACTTTGAGCACCTC
hpt04          CGATTCCATTGAAAAATAGGTTATCATGTCCTGTATTAATTTATCCTGTGTATATGATTCTTGTATTTTTTTTCCCACCTCTTGTTGTTATTATTGACTTTGAGCACCTC
hpt05          CGATTCCATTGAAAAATAGGTTATCATGTCCTGTATTAATTTATCCTGTGTATATGATTCTTGTATTTTTTTTCCCACCTCTTGTTGTTATTATTGACTTTGAGCACCTC
hpt06          CGATTCCATTGAAAAATAGGTTATCATGTCCTGTATTAATTTATCNTGTGTATATGATTCTTGTATTTTTTTTCCCACCTCTTGTTGTTATTATTGACTTTGAGCACCTC
hpt07          CGATTCCATTGAAAAATAGGTTATCATGTCCTGTATTAATTTATCCTGTGTATATGATTCTTGTATTTTTTTTCCCACCTCTTGTTGTTATTATTGACTTTGAGCACCTC
hpt08          CGATTCCATTGAACAATAGGTTATCATGTCCTGTATTAATTTATCCTGTGTATATGATTCTTGTATTTTTTTTCCCACCTCTTGTTGTTATTATTGACTTTGAGCACCTC
hpt09          CGATTCCATTGAAAAATAGGTTATCATGTCCTGTATTACTTTATCCTGTGTATATGATTCTTGTATTTTTTT-CCCACCTCTTGTTGTTATTATTGACTTTGAGCACCTC
hpt10          CGATTCCATTGAAAAATAGGTTATCATGTCCTGTATTAATTTATCCTGTGTATATGATTCTTGTATTTTTTTTCCCACCTCTTGTTGTTATTATTGACTTTGAGCACCTC
hpt14          CGATTCCATTGAAAAATAGGTTATCATGTCCTGTATTAATTTATCNTGTGTATATGATTCTTGTATTTTTTTTCCCACCTCTTGTTGTTATTATTGACTTTGAGCACCTC
hpt11          CGATTCCATTGAACAATAGGTTATCATGTCCTGTATTAATTTATCCTGTGTATATGATTCTTGTATTTTTTTTCCCACCTCTTGTTGTTATTATTGACTTTGAGCACCTC
hpt12          CGATTCCATTGAAAAATAGGTTATCATGTCCTGTATTAATTTATCCTGTGTATATGATTCTTGTATTTTTTTTCCCACCTCTTGTTGTTATTATTGACTTTGAGCACCTC
hpt13          CGATTCCATTGAAAAATAGGTTATCATGTCCTGTATTAATTTATCTTGTGTATATGATTCTTGTATTTTTTTTCCCACCTCTTGTTGTTATTATTGACTTTGAGCACCTC
hpt15          CGATTCCATTGAAAAATAGGTTATCATGTCCTGTATTAATTTATCCTGTGTATATGATTCTTGTATTTTTTT-CCCACCTCTTGTTGTTATTATTGACTTTGAGCACCTC
hpt16          CGATTCCATTGAAAAATAGGTTATCATGTCCTGTATTAATTTATCTTGTGTATATGATTCTTGTATTTTTTTTCCCACCTCTTGTTGTTATTATTGACTTTGAGCACCTC
hpt17          CGATTCCATTGAAAAATAGGTTATCATGTCCTGTATTAATTTATCTTGTGTATATGATTCTTGTATTTTTTT-CCCACCTCTTGTTGTTATTATTGACTTTGAGCACCTC
hpt18          CGATTCCATTGAAAAATAGGTTATCATGTCCTGTATTAATTTATCCTGTGTATATGATTCTTGTATTTTTTTTCCCACCTCTTGTTGTTATTATTGACTTTGAGCACCTC
```

```
                    1300      1310      1320      1330      1340      1350      1360      1370      1380      1390      1400
              ....|....|....|....|....|....|....|....|....|....|....|....|....|....|....|....|....|....|....|....|....|....|
HvGAMYB mRNA  ------~ttgccagggcgtactgataatgaaataaagaattactggaacactcgaataaagagatgtcagcgagccggtttgccaatatatcctgctagtgtatgcaatc
HvGAMYB_CDS   ------~TTGCCAGGGCGTACTGATAACGAAATAAAGAATTACTGGAACACTCGAATAAAGAGATGTCAGCGAGCCGGTTTGCCAATATATCCTGCTAGTGTATGCAATC
hpt01         TTCTCAGTTGCCAGGGCGTACTGATAACGAAATAAAGAATTACTGGAACACTCGAATAAAGAGATGTCAGCGAGCCGGTTTGCCAATATATCCTGCTAGTGTATGCAATC
hpt02         TTCTCAGTTGCCAGGGCGTACTGATAATGAAATAAAGAATTACTGGAACACTCGAATAAAGAGATGTCAGCGAGCCGGTTTGCCAATATATCCTGCTAGTGTATGCAATC
hpt03         TTCTCAGTTGCCAGGGCGTACTGATAACGAAATAAAGAATTACTGGAACACTCGAATAAAGAGATGTCAGCGAGCCGGTTTGCCAATATATCCTGCTAGTGTATGCAATC
hpt04         TTCTCAGTTGCCAGGGCGTACTGATAATGAAATAAAGAATTACTGGAACACTCGAATAAAGAGATGTCAGCGAGCCGGTTTGCCAATATATCCTGCTAGTGTATGCAATC
hpt05         TTCTCAGTTGCCAGGGCGTACTGATAATGAAATAAAGAATTACTGGAACACTCGAATAAAGAGATGTCAGCGAGCCGGTTTGCCAATATATCCTGCTAGTGTATGCAATC
hpt06         TTCTCAGTTGCCAGGGCGTACTGATAACGAAATAAAGAATTACTGGAACACTCGAATAAAGAGATGTCAGCGAGCCGGTTTGCCAATATATCCTGCTAGTGTATGCAATC
hpt07         TTCTCAGTTGCCAGGGCGTACTGATAATGAAATAAAGAATTACTGGAACACTCGAATAAAGAGATGTCAGCGAGCCGGTTTGCCAATATATCCTGCTAGTGTATGCAATC
hpt08         TTCTCAGTTGCCAGGGCGTACTGATAATGAAATAAAGAATTACTGGAACACTCGAATAAAGAGATGTCAGCGAGCCGGTTTGCCAATATATCCTGCTAGTGTATGCAATC
hpt09         TTCTCAGTTGCCAGGGCGTACTGATAATGAAATAAAGAATTACTGGAACACTCGAATAAAGAGATGTCAGCGAGCCGGTTTGCCAATATATCCTGCTAGTGTATGCAATC
hpt10         TTCTCAGTTGCCAGGGCGTACTGATAATGAAATAAAGAATTACTGGAACACTCGAATAAAGAGATGTCAGCGAGCCGGTTTGCCAATATATCCTGCTAGTGTATGCAATC
hpt14         TTCTCAGTTGCCAGGGCGTACTGATAACGAAATAAAGAATTACTGGAACACTCGAATAAAGAGATGTCAGCGAGCCGGTTTGCCAATATATCCTGCTAGTGTATGCAATC
hpt11         TTCTCAGTTGCCAGGGCGTACTGATAATGAAATAAAGAATTACTGGAACACTCGAATAAAGAGATGTCAGCGAGCCGGTTTGCCAATATATCCTGCTAGTGTATGCAATC
hpt12         TTCTCAGTTGCCAGGGCGTACTGATAATGAAATAAAGAATTACTGGAACACTCGAATAAAGAGATGTCAGCGAGCCGGTTTGCCAATATATCCTGCTAGTGTATGCAATC
hpt13         TTCTCAGTTGCCAGGGCGTACTGATAACGAAATAAAGAATTACTGGAACACTCGAATAAAGAGATGTCAGCGAGCCGGTTTGCCAATATATCCTGCTAGTGTATGCAATC
hpt15         TTCTCAGTTGCCAGGGCGTACTGATAATGAAATAAAGAATTACTGGAACACTCGAATAAAGAGATGTCAGCGAGCCGGTTTGCCAATATATCCTGCTAGTGTATGCAATC
hpt16         TTCTCAGTTGCCAGGGCGTACTGATAACGAAATAAAGAATTACTGGAACACTCGAATAAAGAGATGTCAGCGAGCCGGTTTGCCAATATATCCTGCTAGTGTATGCAATC
hpt17         TTCTCAGTTGCCAGGGCGTACTGATAACGAAATAAAGAATTACTGGAACACTCGAATAAAGAGATGTCAGCGAGCCGGTTTGCCAATATATCCTGCTAGTGTATGCAATC
hpt18         TTCTCAGTTGCCAGGGCGTACTGATAATGAAATAAAGAATTACTGGAACACTCGAATAAAGAGATGTCAGCGAGCCGGTTTGCCAATATATCCTGCTAGTGTATGCAATC

                    1410      1420      1430      1440      1450      1460      1470      1480      1490      1500      1510
              ....|....|....|....|....|....|....|....|....|....|....|....|....|....|....|....|....|....|....|....|....|....|
HvGAMYB mRNA  aatcttcaaatgaagatcagcagggctccagcgatttcaactgcggcgagaatctttccagtgacctcctgaatggaaatggtctttatctgccagattttacctgtgac
HvGAMYB_CDS   AATCTTCAAATGAAGATCAGCAGGGCTCCAGCGATTTCAACTGCGGCGAGAATCTTTCCAGTGACCTCCTGAATGGAAATGGTCTTTATCTGCCAGATTTTACCTGTGAC
hpt01         AATCTTCAAATGAAGATCAGCAGGGCTCCAGCGATTTCAACTGCGGCGAGAATCTTTCCAGTGACCTCCTGAATGGAAATGGTCTTTATCTGCCAGATTTTACCTGTGAC
hpt02         AATCTTCAAATGAAGATCAGCAGGGCTCCAGCGATTTCAACTGCGGCGAGAATCTTTCCAGTGACCTCCTGAATGGAAATGGTCTTTATCTGCCAGATTTTACCTGTGAC
hpt03         AATCTTCAAATGAAGATCAGCAGGGCTCCAGCGATTTCAACTGCGGCGAGAATCTTTCCAGTGACCTCCTGAATGGAAATGGTCTTTATCTGCCAGATTTTACCTGTGAC
hpt04         AATCTTCAAATGAAGATCAGCAGGGCTCCAGCGATTTCAACTGCGGCGAGAATCTTTCCAGTGACCTCCTGAATGGAAATGGTCTTTATCTGCCAGATTTTACCTGTGAC
hpt05         AATCTTCAAATGAAGATCAGCAGGGCTCCAGCGATTTCAACTGCGGCGAGAATCTTTCCAGTGACCTCCTGAATGGAAATGGTCTTTATCTGCCAGATTTTACCTGTGAC
hpt06         AATCTTCAAATGAAGATCAGCAGGGCTCCAGCGATTTCAACTGCGGCGAGAATCTTTCCAGTGACCTCCTGAATGGAAATGGTCTTTATCTGCCAGATTTTACCTGTGAC
hpt07         AATCTTCAAATGAAGATCAGCAGGGCTCCAGCGATTTCAACTGCGGCGAGAATCTTTCCAGTGACCTCCTGAATGGAAATGGTCTTTATCTGCCAGATTTTACCTGTGAC
hpt08         AATCTTCAAATGAAGATCAGCAGGGCTCCAGCGATTTCAACTGCGGCGAGAATCTTTCCAGTGACCTCCTGAATGGAAATGGTCTTTATCTGCCAGATTTTACCTGTGAC
hpt09         AATCTTCAAATGAAGATCAGCAGGGCTCCAGCGATTTCAACTGCGGCGAGAATCTTTCCAGTGACCTCCTGAATGGAAATGGTCTTTATCTGCCAGATTTTACCTGTGAC
hpt10         AATCTTCAAATGAAGATCAGCAGGGCTCCAGCGATTTCAACTGCGGCGAGAATCTTTCCAGTGACCTCCTGAATGGAAATGGTCTTTATCTGCCAGATTTTACCTGTGAC
hpt14         AATCTTCAAATGAAGATCAGCAGGGCTCCAGCGATTTCAACTGCGGCGAGAATCTTTCCAGTGACCTCCTGAATGGAAATGGTCTTTATCTGCCAGATTTTACCTGTGAC
hpt11         AATCTTCAAATGAAGATCAGCAGGGCTCCAGCGATTTCAACTGCGGCGAGAATCTTTCCAGTGACCTCCTGAATGGAAATGGTCTTTATCTGCCAGATTTTACCTGTGAC
hpt12         AATCTTCAAATGAAGATCAGCAGGGCTCCAGCGATTTCAACTGCGGCGAGAATCTTTCCAGTGACCTCCTGAATGGAAATGGTCTTTATCTGCCAGATTTTACCTGTGAC
hpt13         AATCTTCAAATGAAGATCAGCAGGGCTCCAGCGATTTCAACTGCGGCGAGAATCTTTCCAGTGACCTCCTGAATGGAAATGGTCTTTATCTGCCAGATTTTACCTGTGAC
hpt15         AATCTTCAAATGAAGATCAGCAGGGCTCCAGCGATTTCAACTGCGGCGAGAATCTTTCCAGTGACCTCCTGAATGGAAATGGTCTTTATCTGCCAGATTTTACCTGTGAC
hpt16         AATCTTCAAATGAAGATCAGCAGGGCTCCAGCGATTTCAACTGCGGCGAGAATCTTTCCAGTGACCTCCTGAATGGAAATGGTCTTTATCTGCCAGATTTTACCTGTGAC
hpt17         AATCTTCAAATGAAGATCAGCAGGGCTCCAGCGATTTCAACTGCGGCGAGAATCTTTCCAGTGACCTCCTGAATGGAAATGGTCTTTATCTGCCAGATTTTACCTGTGAC
hpt18         AATCTTCAAATGAAGATCAGCAGGGCTCCAGCGATTTCAACTGCGGCGAGAATCTTTCCAGTGACCTCCTGAATGGAAATGGTCTTTATCTGCCAGATTTTACCTGTGAC
```

```
                        1520      1530      1540      1550      1560      1570      1580      1590      1600      1610      1620
                ....|....|....|....|....|....|....|....|....|....|....|....|....|....|....|....|....|....|....|....|....|....|
HvGAMYB mRNA    aatttcattgctaattcagaggctctatcttatgcaccacagctttcagctgtttcaataagcagtttgcttggccagagctttgcatccaaaaactgcggcttcatgga
HvGAMYB_CDS     AATTTCATTGCTAATTCAGAGGCTCTATCTTATGCACCACAGCTTTCAGCTGTTTCAATAAGCAGTTTGCTTGGCCAGAGCTTTGCATCCAAAAACTGCGGCTTCATGGA
hpt01           AATTTCATTGCTAATTCAGAGGCTCTATCTTATGCACCACAGCTTTCAGCTGTTTCAATAAGCAGTTTGCTTGGCCAGAGCTTTGCATCCAAAAACTGCGGCTTCATGGA
hpt02           AATTTCATTGCTAATTCAGAGGCTCTATCTTATGCACCACAGCTTTCAGCTGTTTCAATAAGCAGTTTGCTTGGCCAGAGCTTTGCATCCAAAAACTGCGGCTTCATGGA
hpt03           AATTTCATTGCTAATTCAGAGGCTCTATCTTATGCACCACAGCTTTCAGCTGTTTCAATAAGCAGTTTGCTTGGCCAGAGCTTTGCATCCAAAAACTGCGGCTTCATGGA
hpt04           AATTTCATTGCTAATTCAGAGGCTCTATCTTATGCACCACAGCTTTCAGCTGTTTCAATAAGCAGTTTGCTTGGCCAGAGCTTTGCATCCAAAAACTGCGGCTTCATGGA
hpt05           AATTTCATTGCTAATTCAGAGGCTCTATCTTATGCACCACAGCTTTCAGCTGTTTCAATAAGCAGTTTGCTTGGCCAGAGCTTTGCATCCAAAAACTGCGGCTTCATGGA
hpt06           AATTTCATTGCTAATTCAGAGGCTCTATCTTATGCACCACAGCTTTCAGCTGTTTCAATAAGCAGTTTGCTTGGCCAGAGCTTTGCATCCAAAAACTGCGGCTTCATGGA
hpt07           AATTTCATTGCTAATTCAGAGGCTCTATCTTATGCACCACAGCTTTCAGCTGTTTCAATAAGCAGTTTGCTTGGCCAGAGCTTTGCATCCAAAAACTGCGGCTTCATGGA
hpt08           AATTTCATTGCTAATTCAGAGGCTCTATCTTATGCACCACAGCTTTCAGCTGTTTCAATAAGCAGTTTGCTTGGCCAGAGCTTTGCATCCAAAAACTGCGGCTTCATGGA
hpt09           AATTTCATTGCTAATTCAGAGGCTCTATCTTATGCACCACAGCTTTCAGCTGTTTCAATAAGCAGTTTGCTTGGCCAGAGCTTTGCATCCAAAAACTGCGGCTTCATGGA
hpt10           AATTTCATTGCTAATTCAGAGGCTCTATCTTATGCACCACAGCTTTCAGCTGTTTCAATAAGCAGTTTGCTTGGCCAGAGCTTTGCATCCAAAAACTGCGGCTTCATGGA
hpt14           AATTTCATTGCTAATTCAGAGGCTCTATCTTATGCACCACAGCTTTCAGCTGTTTCAATAAGCAGTTTGCTTGGCCAGAGCTTTGCATCCAAAAACTGCGGCTTCATGGA
hpt11           AATTTCATTGCTAATTCAGAGGCTCTATCTTATGCACCACAGCTTTCAGCTGTTTCAATAAGCAGTTTGCTTGGCCAGAGCTTTGCATCCAAAAACTGCGGCTTCATGGA
hpt12           AATTTCATTGCTAATTCAGAGGCTCTATCTTATGCACCACAGCTTTCAGCTGTTTCAATAAGCAGTTTGCTTGGCCAGAGCTTTGCATCCAAAAACTGCGGCTTCATGGA
hpt13           AATGTCATTGCTAATTCAGAGGCTCTATCTTATGCACCACAGCTTTCAGCTGTTTCAATAAGCAGTTTGCTTGGCCAGAGCTTTGCATCCAAAAACTGCGGCTTCATGGA
hpt15           AATTTCATTGCTAATTCAGAGGCTCTATCTTATGCACCACAGCTTTCAGCTGTTTCAATAAGCAGTTTGCTTGGCCAGAGCTTTGCATCCAAAAACTGCGGCTTCATGGA
hpt16           AATTTCATTGCTAATTCAGAGGCTCTATCTTATGCACCACAGCTTTCAGCTGTTTCAATAAGCAGTTTGCTTGGCCAGAGCTTTGCATCCAAAAACTGCGGCTTCATGGA
hpt17           AATTTCATTGCTAATTCAGAGGCTCTATCTTATGCACCACAGCTTTCAGCTGTTTCAATAAGCAGTTTGCTTGGCCAGAGCTTTGCATCCAAAAACTGCGGCTTCATGGA
hpt18           AATTTCATTGCTAATTCAGAGGCTCTATCTTATGCACCACAGCTTTCAGCTGTTTCAATAAGCAGTTTGCTTGGCCAGAGCTTTGCATCCAAAAACTGCGGCTTCATGGA

                        1630      1640      1650      1660      1670      1680      1690      1700      1710      1720      1730
                ....|....|....|....|....|....|....|....|....|....|....|....|....|....|....|....|....|....|....|....|....|....|
HvGAMYB mRNA    tcaagtaaaccaagcagggatgctaaaacagtctgaccctttactccctggattgagcgacaccatcaatggcgcgctctcctcggtcgatcaattctcaaatgactctg
HvGAMYB_CDS     TCAAGTAAACCAAGCAGGGATGCTAAAACAGTCTGACCCTTTACTCCCTGGATTGAGCGACACCATCAATGGCGCGCTCTCCTCGGTCGATCAATTCTCAAATGACTCTG
hpt01           TCAAGTAAACCAAGCAGGGATGCTAAAACAGTCTGACCCTTTACTCCCTGGATTGAGCGACACCATCAATGGCGCGCTCTCCTCGGTCGATCAATTCTCAAATGACTCTG
hpt02           TCAAGTAAACCAAGCAGGGATGCTAAAACAGTCTGACCCTTTACTCCCTGGATTGAGCGACACCATCAATGGCGCGCTCTCCTCGGTCGATCAATTCTCAAATGACTCTG
hpt03           TCAAGTAAACCAAGCAGGGATGCTAAAACAGTCTGACCCTTTACTCCCTGGATTGAGCGACACCATCAATGGCGCGCTCTCCTCGGTCGATCAATTCTCAAATGACTCTG
hpt04           TCAAGTAAACCAAGCAGGGATGCTAAAACAGTCTGACCCTTTACTCCCTGGATTGAGCGACACCATCAATGGCGCGCTCTCCTCGGTCGATCAATTCTCAAATGACTCTG
hpt05           TCAAGTAAACCAAGCAGGGATGCTAAAACAGTCTGACCCTTTACTCCCTGGATTGAGCGACACCATCAATGGCGCGCTCTCCTCGGTCGATCAATTCTCAAATGACTCTG
hpt06           TCAAGTAAACCAAGCAGGGATGCTAAAACAGTCTGACCCTTTACTCCCTGGATTGAGCGACACCATCAATGGCGCGCTCTCCTCGGTCGATCAATTCTCAAATGACTCTG
hpt07           TCAAGTAAACCAAGCAGGGATGCTAAAACAGTCTGACCCTTTACTCCCTGGATTGAGCGACACCATCAATGGCGCGCTCTCCTCGGTCGATCAATTCTCAAATGACTCTG
hpt08           TCAAGTAAACCAAGCAGGGATGCTAAAACAGTCTGACCCTTTACTCCCTGGATTGAGCGACACCATCAATGGCGCGCTCTCCTCGGTCGATCAATTCTCAAATGACTCTG
hpt09           TCAAGTAAACCAAGCAGGGATGCTAAAACAGTCTGACCCTTTACTCCCTGGATTGAGCGACACCATCAATGGCGCGCTCTCCTCGGTCGATCAATTCTCAAATGACTCTG
hpt10           TCAAGTAAACCAAGCAGGGATGCTAAAACAGTCTGACCCTTTACTCCCTGGATTGAGCGACACCATCAATGGCGCGCTCTCCTCGGTCGATCAATTCTCAAATGACTCTG
hpt14           TCAAGTAAACCAAGCAGGGATGCTAAAACAGTCTGACCCTTTACTCCCTGGATTGAGCGACACCATCAATGGCGCGCTCTCCTCGGTCGATCAATTCTCAAATGACTCTG
hpt11           TCAAGTAAACCAAGCAGGGATGCTAAAACAGTCTGACCCTTTACTCCCTGGATTGAGCGACACCATCAATGGCGCGCTCTCCTCGGTCGATCAATTCTCAAATGACTCTG
hpt12           TCAAGTAAACCAAGCAGGGATGCTAAAACAGTCTGACCCTTTACTCCCTGGATTGAGCGACACCATCAATGGCGCGCTCTCCTCGGTCGATCAATTCTCAAATGACTCTG
hpt13           TCAAGTAAACCAAGCAGGGATGCTAAAACAGTCTGACCCTTTACTCCCTGGATTGAGCGACACCATCAATGGCGCGCTCTCCTCGGTCGATCAATTCTCAAATGACTCTG
hpt15           TCAAGTAAACCAAGCAGGGATGCTAAAACAGTCTGACCCTTTACTCCCTGGATTGAGCGACACCATCAATGGCGCGCTCTCCTCGGTCGATCAATTCTCAAATGACTCTG
hpt16           TCAAGTAAACCAAGCAGGGATGCTAAAACAGTCTGACCCTTTACTCCCTGGATTGAGCGACACCATCAATGGCGCGCTCTCCTCGGTCGATCAATTCTCAAATGACTCTG
hpt17           TCAAGTAAACCAAGCAGGGATGCTAAAACAGTCTGACCCTTTACTCCCTGGATTGAGCGACACCATCAATGGCGCGCTCTCCTCGGTCGATCAATTCTCAAATGACTCTG
hpt18           TCAAGTAAACCAAGCAGGGATGCTAAAACAGTCTGACCCTTTACTCCCTGGATTGAGCGACACCATCAATGGCGCGCTCTCCTCGGTCGATCAATTCTCAAATGACTCTG
```

```
                    1740      1750      1760      1770      1780      1790      1800      1810      1820      1830      1840
              ....|....|....|....|....|....|....|....|....|....|....|....|....|....|....|....|....|....|....|....|....|....|
HvGAMYB mRNA  agaagctcaagcaggctcttggttttgactatctccacgaagccaactctagcagcaagattattgcaccatttgggggtgcgcttactggcagccatgcctttttaaat
HvGAMYB_CDS   AGAAGCTCAAGCAGGCTCTTGGTTTTGACTATCTCCACGAAGCCAACTCTAGCAGCAAGATTATTGCACCATTTGGGGGTGCGCTTACTGGCAGCCATGCCTTTTTAAAT
hpt01         AGAAGCTCAAGCAGGCTCTTGGTTTTGACTATCTCCACGAAGCCAACTCTAGCAGCAAGATTATTGCACCATTTGGGGGTGCGCTTACTGGCAGCCATGCCTTTTTAAAT
hpt02         AGAAGCTCAAGCAGGCTCTTGGTTTTGACTATCTCCACGAAGCCAACTCTAGCAGCAAGATTATTGCACCATTTGGGGGTGCGCTTACTGGCAGCCATGCCTTTTTAAAT
hpt03         AGAAGCTCAAGCAGGCTCTTGGTTTTGACTATCTCCACGAAGCCAACTCTAGCAGCAAGATTATTGCACCATTTGGGGGTGCGCTTACTGGCAGCCATGCCTTTTTAAAT
hpt04         AGAAGCTCAAGCAGGCTCTTGGTTTTGACTATCTCCACGAAGCCAACTCTAGCAGCAAGATTATTGCACCATTTGGGGGTGCGCTTACTGGCAGCCATGCCTTTTTAAAT
hpt05         AGAAGCTCAAGCAGGCTCTTGGTTTTGACTATCTCCACGAAGCCAACTCTAGCAGCAAGATTATTGCACCATTTGGGGGTGCGCTTACTGGCAGCCATGCCTTTTTAAAT
hpt06         AGAAGCTCAAGCAGGCTCTTGGTTTTGACTATCTCCACGAAGCCAACTCTAGCAGCAAGATTATTGCACCATTTGGGGGTGCGCTTACTGGCAGCCATGCCTTTTTAAAT
hpt07         AGAAGCTCAAGCAGGCTCTTGGTTTTGACTATCTCCACGAAGCCAACTCTAGCAGCAAGATTATTGCACCATTTGGGGGTGCGCTTACTGGCAGCCATGCCTTTTTAAAT
hpt08         AGAAGCTCAAGCAGGCTCTTGGTTTTGACTATCTCCACGAAGCCAACTCTAGCAGCAAGATTATTGCACCATTTGGGGGTGCGCTTACTGGCAGCCATGCCTTTTTAAAT
hpt09         AGAAGCTCAAGCAGGCTCTTGGTTTTGACTATCTCCACGAAGCCAACTCTAGCAGCAAGATTATTGCACCATTTGGGGGTGCGCTTACTGGCAGCCATGCCTTTTTAAAT
hpt10         AGAAGCTCAAGCAGGCTCTTGGTTTTGACTATCTCCACGAAGCCAACTCTAGCAGCAAGATTATTGCACCATTTGGGGGTGCGCTTACTGGCAGCCATGCCTTTTTAAAT
hpt14         AGAAGCTCAAGCAGGCTCTTGGTTTTGACTATCTCCACGAAGCCAACTCTAGCAGCAAGATTATTGCACCATTTGGGGGTGCGCTTACTGGCAGCCATGCCTTTTTAAAT
hpt11         AGAAGCTCAAGCAGGCTCTTGGTTTTGACTATCTCCACGAAGCCAACTCTAGCAGCAAGATTATTGCACCATTTGGGGGTGCGCTTACTGGCAGCCATGCCTTTTTAAAT
hpt12         AGAAGCTCAAGCAGGCTCTTGGTTTTGACTATCTCCACGAAGCCAACTCTAGCAGCAAGATTATTGCACCATTTGGGGGTGCGCTTACTGGCAGCCATGCCTTTTTAAAT
hpt13         AGAAGCTCAAGCAGGCTCTTGGTTTTGACTATCTCCACGAAGCCAACTCTAGCAGCAAGATTATTGCACCATTTGGGGGTGCGCTTACTGGCAGCCATGCCTTTTTAAAT
hpt15         AGAAGCTCAAGCAGGCTCTTGGTTTTGACTATCTCCACGAAGCCAACTCTAGCAGCAAGATTATTGCACCATTTGGGGGTGCGCTTACTGGCAGCCATGCCTTTTTAAAT
hpt16         AGAAGCTCAAGCAGGCTCTTGGTTTTGACTATCTCCACGAAGCCAACTCTAGCAGCAAGATTATTGCACCATTTGGGGGTGCGCTTACTGGCAGCCATGCCTTTTTAAAT
hpt17         AGAAGCTCAAGCAGGCTCTTGGTTTTGACTATCTCCACGAAGCCAACTCTAGCAGCAAGATTATTGCACCATTTGGGGGTGCGCTTACTGGCAGCCATGCCTTTTTAAAT
hpt18         AGAAGCTCAAGCAGGCTCTTGGTTTTGACTATCTCCACGAAGCCAACTCTAGCAGCAAGATTATTGCACCATTTGGGGGTGCGCTTACTGGCAGCCATGCCTTTTTAAAT


                    1850      1860      1870      1880      1890      1900      1910      1920      1930      1940      1950
              ....|....|....|....|....|....|....|....|....|....|....|....|....|....|....|....|....|....|....|....|....|....|
HvGAMYB mRNA  ggcaccttctctacttctaggaccatcaatggtcctttgaagatggagctcccttcactccaagataccgaatctgatccgaatagctggctcaagtataccgtggctcc
HvGAMYB_CDS   GGCACCTTCTCTACTTCTAGGACCATCAATGGTCCTTTGAAGATGGAGCTCCCTTCACTCCAAGATACCGAATCTGATCCGAATAGCTGGCTCAAGTATACCGTGGCTCC
hpt01         GGCACCTTCTCTACTTCTAGGACCATCAATGGTCCTTTGAAGATGGAGCTCCCTTCACTCCAAGATACCGAATCTGATCCGAATAGCTGGCTCAAGTATACCGTGGCTCC
hpt02         GGCACCTTCTCTACTTCTAGGACCATCAATGGTCCTTTGAAGATGGAGCTCCCTTCACTCCAAGATACCGAATCTGATCCGAATAGCTGGCTCAAGTATACCGTGGCTCC
hpt03         GGCACCTTCTCTACTTCTAGGACCATCAATGGTCCTTTGAAGATGGAGCTCCCTTCACTCCAAGATACCGAATCTGATCCGAATAGCTGGCTCAAGTATACCGTGGCTCC
hpt04         GGCACCTTCTCTACTTCTAGGACCATCAATGGTCCTTTGAAGATGGAGCTCCCTTCACTCCAAGATACCGAATCTGATCCGAATAGCTGGCTCAAGTATACCGTGGCTCC
hpt05         GGCACCTTCTCTACTTCTAGGACCATCAATGGTCCTTTGAAGATGGAGCTCCCTTCACTCCAAGATACCGAATCTGATCCGAATAGCTGGCTCAAGTATACCGTGGCTCC
hpt06         GGCACCTTCTCTACTTCTAGGACCATCAATGGTCCTTTGAAGATGGAGCTCCCTTCACTCCAAGATACCGAATCTGATCCGAATAGCTGGCTCAAGTATACCGTGGCTCC
hpt07         GGCACCTTCTCTACTTCTAGGACCATCAATGGTCCTTTGAAGATGGAGCTCCCTTCACTCCAAGATACCGAATCTGATCCGAATAGCTGGCTCAAGTATACCGTGGCTCC
hpt08         GGCACCTTCTCTACTTCTAGGACCATCAATGGTCCTTTGAAGATGGAGCTCCCTTCACTCCAAGATACCGAATCTGATCCGAATAGCTGGCTCAAGTATACCGTGGCTCC
hpt09         GGCACCTTCTCTACTTCTAGGACCATCAATGGTCCTTTGAAGATGGAGCTCCCTTCACTCCAAGATACCGAATCTGATCCGAATAGCTGGCTCAAGTATACCGTGGCTCC
hpt10         GGCACCTTCTCTACTTCTAGGACCATCAATGGTCCTTTGAAGATGGAGCTCCCTTCACTCCAAGATACCGAATCTGATCCGAATAGCTGGCTCAAGTATACCGTGGCTCC
hpt14         GGCACCTTCTCTACTTCTAGGACCATCAATGGTCCTTTGAAGATGGAGCTCCCTTCACTCCAAGATACCGAATCTGATCCGAATAGCTGGCTCAAGTATACCGTGGCTCC
hpt11         GGCACCTTCTCTACTTCTAGGACCATCAATGGTCCTTTGAAGATGGAGCTCCCTTCACTCCAAGATACCGAATCTGATCCGAATAGCTGGCTCAAGTATACCGTGGCTCC
hpt12         GGCACCTTCTCTACTTCTAGGACCATCAATGGTCCTTTGAAGATGGAGCTCCCTTCACTCCAAGATACCGAATCTGATCCGAATAGCTGGCTCAAGTATACCGTGGCTCC
hpt13         GGCACCTTCTCTACTTCTAGGACCATCAATGGTCCTTTGAAGATGGAGCTCCCTTCACTCCAAGATACCGAATCTGATCCGAATAGCTGGCTCAAGTATACCGTGGCTCC
hpt15         GGCACCTTCTCTACTTCTAGGACCATCAATGGTCCTTTGAAGATGGAGCTCCCTTCACTCCAAGATACCGAATCTGATCCGAATAGCTGGCTCAAGTATACCGTGGCTCC
hpt16         GGCACCTTCTCTACTTCTAGGACCATCAATGGTCCTTTGAAGATGGAGCTCCCTTCACTCCAAGATACCGAATCTGATCCGAATAGCTGGCTCAAGTATACCGTGGCTCC
hpt17         GGCACCTTCTCTACTTCTAGGACCATCAATGGTCCTTTGAAGATGGAGCTCCCTTCACTCCAAGATACCGAATCTGATCCGAATAGCTGGCTCAAGTATACCGTGGCTCC
hpt18         GGCACCTTCTCTACTTCTAGGACCATCAATGGTCCTTTGAAGATGGAGCTCCCTTCACTCCAAGATACCGAATCTGATCCGAATAGCTGGCTCAAGTATACCGTGGCTCC
```

```
                           1960      1970      1980      1990      2000      2010      2020      2030      2040      2050      2060
                     ....|....|....|....|....|....|....|....|....|....|....|....|....|....|....|....|....|....|....|....|....|....|
HvGAMYB mRNA         tgcgatgcagcctacggagttggttgatccgtacctgcagtccccgacagcaactccgtcagtgaaatcggagtgtgcttcgccgaggaacagcggcctcttggaagagc
HvGAMYB_CDS          TGCGATGCAGCCTACGGAGTTGGTTGATCCGTACATGCAGTCCCCGACAGCAACTCCGTCAGTGAAATCGGAGTGTGCTTCGCCGAGGAACAGCGGCCTCTTGGAAGAGC
hpt01                TGCGATGCAGCCTACGGAGTTGGTTGATCCGTACCTGCAGTCCCCGACAGCAACTCCGTCAGTGAAATCGGAGTGTGCTTCGCCGAGGAACAGCGGCCTCTTGGAAGAGC
hpt02                TGCGATGCAGCCTACGGAGTTGGTTGATCCGTACCTGCAGTCCCCGACAGCAACTCCGTCAGTGAAATCGGAGTGTGCTTCGCCGAGGAACAGCGGCCTCTTGGAAGAGC
hpt03                TGCGATGCAGCCTACGGAGTTGGTTGATCCGTACCTGCAGTCCCCGACAGCAACTCCGTCAGTGAAATCGGAGTGTGCTTCGCCGAGGAACAGCGGCCTCTTGGAAGAGC
hpt04                TGCGATGCAGCCTACGGAGTTGGTTGATCCGTACCTGCAGTCCCCGACAGCAACTCCGTCAGTGAAATCGGAGTGTGCTTCGCCGAGGAACAGCGGCCTCTTGGAAGAGC
hpt05                TGCGATGCAGCCTACGGAGTTGGTTGATCCGTACCTGCAGTCCCCGACAGCAACTCCGTCAGTGAAATCGGAGTGTGCTTCGCCGAGGAACAGCGGCCTCTTGGAAGAGC
hpt06                TGCGATGCAGCCTACGGAGTTGGTTGATCCGTACCTGCAGTCCCCGACAGCAACTCCGTCAGTGAAATCGGAGTGTGCTTCGCCGAGGAACAGCGGCCTCTTGGAAGAGC
hpt07                TGCGATGCAGCCTACGGAGTTGGTTGATCCGTACCTGCAGTCCCCGACAGCAACTCCGTCAGTGAAATCGGAGTGTGCTTCGCCGAGGAACAGCGGCCTCTTGGAAGAGC
hpt08                TGCGATGCAGCCTACGGAGTTGGTTGATCCGTACCTGCAGTCCCCGACAGCAACTCCGTCAGTGAAATCGGAGTGTGCTTCGCCGAGGAACAGCGGCCTCTTGGAAGAGC
hpt09                TGCGATGCAGCCTACGGAGTTGGTTGATCCGTACCTGCAGTCCCCGACAGCAACTCCGTCAGTGAAATCGGAGTGTGCTTCGCCGAGGAACAGCGGCCTCTTGGAAGAGC
hpt10                TGCGATGCAGCCTACGGAGTTGGTTGATCCGTACCTGCAGTCCCCGACAGCAACTCCGTCAGTGAAATCGGAGTGTGCTTCGCCGAGGAACAGCGGCCTCTTGGAAGAGC
hpt14                TGCGATGCAGCCTACGGAGTTGGTTGATCCGTACATGCAGTCCCCGACAGCAACTCCGTCAGTGAAATCGGAGTGTGCTTCGCCGAGGAACAGCGGCCTCTTGGAAGAGC
hpt11                TGCGATGCAGCCTACGGAGTTGGTTGATCCGTACCTGCAGTCCCCGACAGCAACTCCGTCAGTGAAATCGGAGTGTGCTTCGCCGAGGAACAGCGGCCTCTTGGAAGAGC
hpt12                TGCGATGCAGCCTACGGAGTTGGTTGATCCGTACCTGCAGTCCCCGACAGCAACTCCGTCAGTGAAATCGGAGTGTGCTTCGCCGAGGAACAGCGGCCTCTTGGAAGAGC
hpt13                TGCGATGCAGCCTACGGAGTTGGTTGATCCGTACCTGCAGTCCCCGACAGCAACTCCGTCAGTGAAATCGGAGTGTGCTTCGCCGAGGAACAGCGGCCTCTTGGAAGAGC
hpt15                TGCGATGCAGCCTACGGAGTTGGTTGATCCGTACCTGCAGTCCCCGACAGCAACTCCGTCAGTGAAATCGGAGTGTGCTTCGCCGAGGAACAGCGGCCTCTTGGAAGAGC
hpt16                TGCGATGCAGCCTACGGAGTTGGTTGATCCGTACCTGCAGTCCCCGACAGCAACTCCGTCAGTGAAATCGGAGTGTGCTTCGCCGAGGAACAGCGGCCTCTTGGAAGAGC
hpt17                TGCGATGCAGCCTACGGAGTTGGTTGATCCGTACCTGCAGTCCCCGACAGCAACTCCGTCAGTGAAATCGGAGTGTGCTTCGCCGAGGAACAGCGGCCTCTTGGAAGAGC
hpt18                TGCGATGCAGCCTACGGAGTTGGTTGATCCGTACCTGCAGTCCCCGACAGCAACTCCGTCAGTGAAATCGGAGTGTGCTTCGCCGAGGAACAGCGGCCTCTTGGAAGAGC

                           2070      2080      2090      2100      2110      2120      2130      2140      2150      2160      2170
                     ....|....|....|....|....|....|....|....|....|....|....|....|....|....|....|....|....|....|....|....|....|....|
HvGAMYB mRNA         tgcttcatgaagctcagggactaagatctgggaagaatcagcagctctccgtgagaagttcaagttcctctgtcagcacgccgtgtgataccacggtggttagcccggag
HvGAMYB_CDS          TGCTTCATGAAGCTCAGGGACTAAGATCTGGGAAGAATCAGCAGCTCTCCGTGAGAAGTTCAAGTTCCTCTGTCAGCACGCCGTGTGATACCACGGTGGTTAGCCCGGAG
hpt01                TGCTTCATGAAGCTCAGGGACTAAGATCTGGGAAGAATCAGCAGCTCTCCGTGAGAAGTTCAAGTTCCTCTGTCAGCACGCCGTGTGATACCACGGTGGTTAGCCCGGAG
hpt02                TGCTTCATGAAGCTCAGGGACTAAGATCTGGGAAGAATCAGCAGCTCTCCGTGAGAAGTTCAAGTTCCTCTGTCAGCACGCCGTGTGATACCACGGTGGTTAGCCCGGAG
hpt03                TGCTTCATGAAGCTCAGGGACTAAGATCTGGGAAGAATCAGCAGCTCTCCGTGAGAAGTTCAAGTTCCTCTGTCAGCACGCCGTGTGATACCACGGTGGTTAGCCCGGAG
hpt04                TGCTTCATGAAGCTCAGGGACTAAGATCTGGGAAGAATCAGCAGCTCTCCGTGAGAAGTTCAAGTTCCTCTGTCAGCACGCCGTGTGATACCACGGTGGTTAGCCCGGAG
hpt05                TGCTTCATGAAGCTCAGGGACTAAGATCTGGGAAGAATCAGCAGCTCTCCGTGAGAAGTTCAAGTTCCTCTGTCAGCACGCCGTGTGATACCACGGTGGTTAGCCCGGAG
hpt06                TGCTTCATGAAGCTCAGGGACTAAGATCTGGGAAGAATCAGCAGCTCTCCGTGAGAAGTTCAAGTTCCTCTGTCAGCACGCCGTGTGATACCACGGTGGTTAGCCCGGAG
hpt07                TGCTTCATGAAGCTCAGGGGCTAAGATCTGGGAAGAATCAGCAGCTCTCCGTGAGAAGTTCAAGTTCCTCTGTCAGCACGCCGTGTGATACCACGGTGGTTAGCCCGGAG
hpt08                TGCTTCATGAAGCTCAGGGACTAAGATCTGGGAAGAATCAGCAGCTCTCCGTGAGAAGTTCAAGTTCCTCTGTCAGCACGCCGTGTGATACCACGGTGGTTAGCCCGGAG
hpt09                TGCTTCATGAAGCTCAGGGACTAAGATCTGGGAAGAATCAGCAGCTCTCCGTGAGAAGTTCAAGTTCCTCTGTCAGCACGCCGTGTGATACCACGGTGGTTAGCCCGGAG
hpt10                TGCTTCATGAAGCTCAGGGACTAAGATCTGGGAAGAATCAGCAGCTCTCCGTGAGAAGTTCAAGTTCCTCTGTCAGCACGCCGTGTGATACCACGGTGGTTAGCCCGGAG
hpt14                TGCTTCATGAAGCTCAGGGACTAAGATCTGGGAAGAATCAGCAGCTCTCCGTGAGAAGTTCAAGTTCCTCTGTCAGCACGCCGTGTGATACCACGGTGGTTAGCCCGGAG
hpt11                TGCTTCATGAAGCTCAGGGACTAAGATCTGGGAAGAATCAGCAGCTCTCCGTGAGAAGTTCAAGTTCCTCTGTCAGCACGCCGTGTGATACCACGGTGGTTAGCCCGGAG
hpt12                TGCTTCATGAAGCTCAGGGACTAAGATCTGGGAAGAATCAGCAGCTCTCCGTGAGAAGTTCAAGTTCCTCTGTCAGCACGCCGTGTGATACCACGGTGGTTAGCCCGGAG
hpt13                TGCTTCATGAAGCTCAGGGACTAAGATCTGGGAAGAATCAGCAGCTCTCCGTGAGAAGTTCAAGTTCCTCTGTCAGCACGCCGTGTGATACCACGGTGGTTAGCCCGGAG
hpt15                TGCTTCATGAAGCTCAGGGACTAAGATCTGGGAAGAATCAGCAGCTCTCCGTGAGAAGTTCAAGTTCCTCTGTCAGCACGCCGTGTGATACCACGGTGGTTAGCCCGGAG
hpt16                TGCTTCATGAAGCTCAGGGACTAAGATCTGGGAAGAATCAGCAGCTCTCCGTGAGAAGTTCAAGTTCCTCTGTCAGCACGCCGTGTGATACCACGGTGGTTAGCCCGGAG
hpt17                TGCTTCATGAAGCTCAGGGACTAAGATCTGGGAAGAATCAGCAGCTCTCCGTGAGAAGTTCAAGTTCCTCTGTCAGCACGCCGTGTGATACCACGGTGGTTAGCCCGGAG
hpt18                TGCTTCATGAAGCTCAGGGACTAAGATCTGGGAAGAATCAGCAGCTCTCCGTGAGAAGTTCAAGTTCCTCTGTCAGCACGCCGTGTGATACCACGGTGGTTAGCCCGGAG
```

```
                    2180      2190      2200      2210      2220      2230      2240      2250      2260      2270      2280
               ....|....|....|....|....|....|....|....|....|....|....|....|....|....|....|....|....|....|....|....|....|....|
HvGAMYB mRNA   tttgatctctgtcaggaatattgggaagaacgtctgaatgaatatgccccattcagtggcaattcactcactggatccaccgctcctatgagtgctgcgtcgcctgatgt
HvGAMYB_CDS    TTTGATCTCTGTCAGGAATATTGGGAAGAACGTCTGAATGAATATGCCCCATTCAGTGGCAATTCACTCACTGGATCCACCGCTCCTATGAGTGCTGCGTCGCCTGATGT
hpt01          TTTGATCTCTGTCAGGAATATTGGGAAGAACGTCTGAATGAATATGCCCCATTCAGTGGCAATTCTCTCACTGGATCCACCGCTCCTATGAGTGCTGCGTCGCCTGATGT
hpt02          TTTGATCTCTGTCAGGAATATTGGGAAGAACGTCTGAATGAATATGCCCCATTCAGTGGCAATTCACTCACTGGATCCACCGCTCCTATGAGTGCTGCGTCGCCTGATGT
hpt03          TTTGATCTCTGTCAGGAATATTGGGAAGAACGTCTGAATGAATATGCCCCATTCAGTGGCAATTCACTCACTGGATCCACCGCTCCTATGAGTGCTGCGTCGCCTGATGT
hpt04          TTTGATCTCTGTCAGGAATATTGGGAAGAACGTCTGAATGAATATGCCCCATTCAGTGGCAATTCACTCACTGGATCCACCGCTCCTATGAGTGCTGCGTCGCCTGATGT
hpt05          TTTGATCTCTGTCAGGAATATTGGGAAGAACGTCTGAATGAATATGCCCCATTCAGTGGCAATTCACTCACTGGATCCACCGCTCCTATGAGTGCTGCGTCGCCTGATGT
hpt06          TTTGATCTCTGTCAGGAATATTGGGAAGAACGTCTGAATGAATATGCCCCATTCAGTGGCAATTCTCTCACTGGATCCACCGCTCCTATGAGTGCTGCGTCGCCTGATGT
hpt07          TTTGATCTCTGTCAGGAATATTGGGAAGAACGTCTGAATGAATATGCCCCATTCAGTGGCAATTCACTCACTGGATCCACCGCTCCTATGAGTGCTGCGTCGCCTGATGT
hpt08          TTTGATCTCTGTCAGGAATATTGGGAAGAACGTCTGAATGAATATGCCCCATTCAGTGGCAATTCACTCACTGGATCCACCGCTCCTATGAGTGCTGCGTCGCCTGATGT
hpt09          TTTGATCTCTGTCAGGAATATTGGGAAGAACGTCTGAATGAATATGCCCCATTCAGTGGCAATTCACTCACTGGATCCACCGCTCCTATGAGTGCTGCGTCGCCTGATGT
hpt10          TTTGATCTCTGTCAGGAATATTGGGAAGAACGTCTGAATGAATATGCCCCATTCAGTGGCAATTCACTCACTGGATCCACCGCTCCTATGAGTGCTGCGTCGCCTGATGT
hpt14          TTTGATCTCTGTCAGGAATATTGGGAAGAACGTCTGAATGAATATGCCCCATTCAGTGGCAATTCTCTCACTGGATCCACCGCTCCTATGAGTGCTGCGTCGCCTGATGT
hpt11          TTTGATCTCTGTCAGGAATATTGGGAAGAACGTCTGAATGAATATGCCCCATTCAGTGGCAATTCACTCACTGGATCCACCGCTCCTATGAGTGCTGCGTCGCCTGATGT
hpt12          TTTGATCTCTGTCAGGAATATTGGGAAGAACGTCTGAATGAATATGCCCCATTCAGTGGCAATTCACTCACTGGATCCACCGCTCCTATGAGTGCTGCGTCGCCTGATGT
hpt13          TTTGATCTCTGTCAGGAATATTGGGAAGAACGTCTGAATGAATATGCCCCATTCAGTGGCAATTCTCTCACTGGATCCACCGCTCCTATGAGTGCTGCGTCGCCTGATGT
hpt15          TTTGATCTCTGTCAGGAATATTGGGAAGAACGTCTGAATGAATATGCCCCATTCAGTGGCAATTCACTCACTGGATCCACCGCTCCTATGAGTGCTGCGTCGCCTGATGT
hpt16          TTTGATCTCTGTCAGGAATATTGGGAAGAACGTCTGAATGAATATGCCCCATTCAGTGGCAATTCTCTCACTGGATCCACCGCTCCTATGAGTGCTGCGTCGCCTGATGT
hpt17          TTTGATCTCTGTCAGGAATATTGGGAAGAACGTCTGAATGAATATGCCCCATTCAGTGGCAATTCTCTCACTGGATCCACCGCTCCTATGAGTGCTGCGTCGCCTGATGT
hpt18          TTTGATCTCTGTCAGGAATATTGGGAAGAACGTCTGAATGAATATGCCCCATTCAGTGGCAATTCACTCACTGGATCCACCGCTCCTATGAGTGCTGCGTCGCCTGATGT

                    2290      2300      2310      2320      2330      2340      2350      2360      2370      2380      2390
               ....|....|....|....|....|....|....|....|....|....|....|....|....|....|....|....|....|....|....|....|....|....|
HvGAMYB mRNA   ttttcagctctccaaaatttctcctg-------------------------------------------------------------------------------cacaa
HvGAMYB_CDS    TTTTCAGCTCTCCAAAATTTCTCCTG-------------------------------------------------------------------------------CACAA
hpt01          TTTTCAGCTCTCCAAAATTTCTCCTGGTGAGTATATTTTAATTGTCGTCCGTTCTTGAGAGTGATAAAACTGTGCTGAGTTACTTATATGAATATCTATTTGCAGCACAA
hpt02          TTTTCAGCTCTCCAAAATTTCTCCTGGTGAGTATATTTTAATTGTCGTCCGTTCTTGAGAGTGATAAAACTGTGCTGAGTTACTTATATGAATATCTATTTGCAGCACAA
hpt03          TTTTCAGCTCTCCAAAATTTCTCCTGGTGAGTATATTTTAATTGTCGTCCGTTCTTGAGAGTGATAAAACTGTGCTGAGTTACTTATATGAATATCTATTTGCAGCACAA
hpt04          TTTTCAGCTCTCCAAAATTTCTCCTGGTGAGTATATTTTAATTGTCGTCCGTTCTTGAGAGTGATAAAACTGTGCTGAGTTACTTATATGAATATCTATTTGCAGCACAA
hpt05          TTTTCAGCTCTCCAAAATTTCTCCTGGTGAGTATATTTTAATTGTCGTCCGTTCTTGAGAGTGATAAAACTGTGCTGAGTTACTTATATGAATATCTATTTGCAGCACAA
hpt06          TTTTCAGCTCTCMAAAATTTCTCCTGGTGAGTATATTTTAATTGTCGTCCGTTCTTGAGAGTGATAAAACTGTGCTGAGTTACTTATATGAATATCTATTTGCAGCACAA
hpt07          TTTTCAGCTCTCCAAAATTTCTCCTGGTGAGTATATTTTAATTGTCGTCCGTTCTTGAGAGTGATAAAACTGTGCTGAGTTACTTATATGAATATCTATTTGCAGCACAA
hpt08          TTTTCAGCTCTCCAAAATTTCTCCTGGTGAGTATATTTTAATTGTCGTCCGTTCTTGAGAGTGATAAAACTGTGCTGAGTTACTTATATGAATATCTATTTGCAGCACAA
hpt09          TTTTCAGCTCTCCAAAATTTCTCCTGGTGAGTATATTTTAATTGTCGTCCGTTCTTGAGAGTGATAAAACTGTGCTGAGTTACTTATATGAATATCTATTTGCAGCACAA
hpt10          TTTTCAGCTCTCCAAAATTTCTCCTGGTGAGTATATTTTAATTGTCGTCCGTTCTTGAGAGTGATAAAACTGTGCTGAGTTACTTATATGAATATCTATTTGCAGCACAA
hpt14          TTTTCAGCTCTCCAAAATTTCTCCTGGTGAGTATATTTTAATTGTCGTCCGTTCTTGAGAGTGATAAAACTGTGCTGAGTTACTTATATGAATATCTATTTGCAGCACAA
hpt11          TTTTCAGCTCTCCAAAATTTCTCCTGGTGAGTATATTTTAATTGTCGTCCGTTCTTGAGAGTGATAAAACTGTGCTGAGTTACTTATATGAATATCTATTTGCAGCACAA
hpt12          TTTTCAGCTCTCCAAAATTTCTCCTGGTGAGTATATTTTAATTGTCGTCCGTTCTTGAGAGTGATAAAACTGTGCTGAGTTACTTATATGAATATCTATTTGCAGCACAA
hpt13          TTTTCAGCTCTCCAAAATTTCTCCTGGTGAGTATATTTTAATTGTCGTCCGTTCTTGAGAGTGATAAAACTGTGCTGAGTTACTTATATGAATATCTATTTGCAGCACAA
hpt15          TTTTCAGCTCTCCAAAATTTCTCCTGGTGAGTATATTTTAATTGTCGTCCGTTCTTGAGAGTGATAAAACTGTGCTGAGTTACTTATATGAATATCTATTTGCAGCACAA
hpt16          TTTTCAGCTCTCCAAAATTTCTCCTGGTGAGTATATTTTAATTGTCGTCCGTTCTTGAGAGTGATAAAACTGTGCTGAGTTACTTATATGAATATCTATTTGCAGCACAA
hpt17          TTTTCAGCTCTCMAAAATTTCTCCTGGTGAGTATATTTTAATTGTCGTCCGTTCTTGAGAGTGATAAAACTGTGCTGAGTTACTTATATGAATATCTATTTGCAGCACAA
hpt18          TTTTCAGCTCTCCAAAATTTCTCCTGGTGAGTATATTTTAATTGTCGTCCGTTCTTGAGAGTGATAAAACTGTGCTGAGTTACTTATATGAATATCTATTTGCAGCACAA
```

```
                       2400      2410      2420      2430      2440      2450      2460      2470      2480      2490      2500
               ....|....|....|....|....|....|....|....|....|....|....|....|....|....|....|....|....|....|....|....|....|....|
HvGAMYB mRNA   agcccttcactgggatctggagagcaggcaatggagcctgcatatgagcccggggcaggggacacttcgtctcatcctgaaaacttgaggccagacgcgttcttctccgg
HvGAMYB_CDS    AGCCCTTCACTGGGATCTGGAGAGCAGGCAATGGAGCCTGCATATGAGCCCGGGGCAGGGGACACTTCGTCTCATCCTGAAAACTTGAGGCCAGACGCGTTCTTCTCCGG
hpt01          AGCCCTTCACTGGGATCTGGAGAGCAGGCAATGGAGCCTGCATATGAGCCCGGGGCAGGGGACACTTCGTCTCATCCTGAAAACTTGAGGCCAGACGCGTTCTTCTCCGG
hpt02          AGCCCTTCACTGGGATCTGGAGAGCAGGCAATGGAGCCTGCATATGAGCCCGGGGCAGGGGACACTTCGTCTCATCCTGAAAACTTGAGGCCAGACGCGTTCTTCTCCGG
hpt03          AGCCCTTCACTGGGATCTGGAGAGCAGGCAATGGAGCCTGCATATGAGCCCGGGGCAGGGGACACTTCGTCTCATCCTGAAAACTTGAGGCCAGACGCGTTCTTCTCCGG
hpt04          AGCCCTTCACTGGGATCTGGAGAGCAGGCAATGGAGCCTGCATATGAGCCCGGGGCAGGGGACACTTCGTCTCATCCTGAAAACTTGAGGCCAGACGCGTTCTTCTCCGG
hpt05          AGCCCTTCACTGGGATCTGGAGAGCAGGCAATGGAGCCTGCATATGAGCCCGGGGCAGGGGACACTTCGTCTCATCCTGAAAACTTGAGGCCAGACGCGTTCTTCTCCGG
hpt06          AGCCCTTCACTGGGATCTGGAGAGCAGGCAATGGAGCCTGCATATGAGCCCGGGGCAGGGGACACTTCGTCTCATCCTGAAAACTTGAGGCCAGACGCGTTCTTCTCCGG
hpt07          AGCCCTTCACTGGGATCTGGAGAGCAGGCAATGGAGCCTGCATATGAGCCCGGGGCAGGGGACACTTCGTCTCATCCTGAAAACTTGAGGCCAGACGCGTTCTTCTCCGG
hpt08          AGCCCTTCACTGGGATCTGGAGAGCAGGCAATGGAGCCTGCATATGAGCCCGGGGCAGGGGACACTTCGTCTCATCCTGAAAACTTGAGGCCAGACGCGTTCTTCTCTGG
hpt09          AGCCCTTCACTGGGATCTGGAGAGCAGGCAATGGAGCCTGCATATGAGCCCGGGGCAGGGGACACTTCGTCTCATCCTGAAAACTTGAGGCCAGACGCGTTCTTCTCCGG
hpt10          AGCCCTTCACTGGGATCTGGAGAGCAGGCAATGGAGCCTGCATATGAGCCCGGGGCAGGGGACACTTCGTCTCATCCTGAAAACTTGAGGCCAGACGCGTTCTTCTCCGG
hpt14          AGCCCTTCACTGGGATCTGGAGAGCAGGCAATGGAGCCTGCATATGAGCCCGGGGCAGGGGACACTTCGTCTCATCCTGAAAACTTGAGGCCAGACGCGTTCTTCTCCGG
hpt11          AGCCCTTCACTGGGATCTGGAGAGCAGGCAATGGAGCCTGCATATGAGCCCGGGGCAGGGGACACTTCGTCTCATCCTGAAAACTTGAGGCCAGACGCGTTCTTCTCTGG
hpt12          AGCCCTTCACTGGGATCTGGAGAGCAGGCAATGGAGCCTGCATATGAGCCCGGGGCAGGGGACACTTCGTCTCATCCTGAAAACTTGAGGCCAGACGCGTTCTTCTCCGG
hpt13          AGCCCTTCACTGGGATCTGGAGAGCAGGCAATGGAGCCTGCATATGAGCCCGGGGCAGGGGACACTTCGTCTCATCCTGAAAACTTGAGGCCAGACGCGTTCTTCTCCGG
hpt15          AGCCCTTCACTGGGATCTGGAGAGCAGGCAATGGAGCCTGCATATGAGCCCGGGGCAGGGGACACTTCGTCTCATCCTGAAAACTTGAGGCCAGACGCGTTCTTCTCCGG
hpt16          AGCCCTTCACTGGGATCTGGAGAGCAGGCAATGGAGCCTGCATATGAGCCCGGGGCAGGGGACACTTCGTCTCATCCTGAAAACTTGAGGCCAGACGCGTTCTTCTCCGG
hpt17          AGCCCTTCACTGGGATCTGGAGAGCAGGCAATGGAGCCTGCATATGAGCCCGGGGCAGGGGACACTTCGTCTCATCCTGAAAACTTGAGGCCAGACGCGTTCTTCTCCGG
hpt18          AGCCCTTCACTGGGATCTGGAGAGCAGGCAATGGAGCCTGCATATGAGCCCGGGGCAGGGGACACTTCGTCTCATCCTGAAAACTTGAGGCCAGACGCGTTCTTCTCCGG
```

```
                       2510      2520      2530      2540      2550      2560      2570      2580      2590      2600      2610
               ....|....|....|....|....|....|....|....|....|....|....|....|....|....|....|....|....|....|....|....|....|....|
HvGAMYB mRNA   gaacacggccgactcgtccgtcttcaacaacgccatagccatgctcctgggcaacgacatgaacacggagtgcaagcctgttttcggcgacggtatcatgtttgatactt
HvGAMYB_CDS    GAACACGGCCGACTCGTCCGTCTTCAACAACGCCATAGCCATGCTCCTGGGCAACGACATGAACACGGAGTGCAAGCCTGTTTTCGGCGACGGTATCATGTTTGATACTT
hpt01          GAACACGGCCGACTCGTCCGTCTTCAACAACGCCATAGCCATGCTCCTGGGCAACGACATGAACACGGAGTGCAAGCCTGTTTTCGGCGACGGTATCATGTTTGATACTT
hpt02          GAACACGGCCGACTCGTCCGTCTTCAACAACGCCATAGCCATGCTCCTGGGCAACGACATGAACACGGAGTGCAAGCCTGTTTTCGGCGACGGTATCATGTTTGATACTT
hpt03          GAACACGGCCGACTCGTCCGTCTTCAACAACGCCATAGCCATGCTCCTGGGCAACGACATGAACACGGAGTGCAAGCCTGTTTTCGGCGACGGTATCATGTTTGATACTT
hpt04          GAACACGGCCGACTCGTCCGTCTTCAACAACGCCATAGCCATGCTCCTGGGCAACGACATGAACACGGAGTGCAAGCCTGTTTTCGGCGACGGTATCATGTTTGATACTT
hpt05          GAACACGGCCGACTCGTCCGTCTTCAACAACGCCATAGCCATGCTCCTGGGCAACGACATGAACACGGAGTGCAAGCCTGTTTTCGGCGACGGTATCATGTTTGATACTT
hpt06          GAACACGGCCGACTCGTCCGTCTTCAACAACGCCATAGCCATGCTCCTGGGCAACGACATGAACACGGAGTGCAAGCCTGTTTTCGGCGACGGTATCATGTTTGATACTT
hpt07          GAACACGGCCGACTCGTCCGTCTTCAACAACGCCATAGCCATGCTCCTGGGCAACGACATGAACACGGAGTGCAAGCCTGTTTTCGGCGACGGTATCATGTTTGATACTT
hpt08          GAACACGGCCGACTCGTCCGTCTTCAACAACGCCATAGCCATGCTCCTGGGCAACGACATGAACACGGAGTGCAAGCCTGTTTTCGGCGACGGTATCATGTTTGATACTT
hpt09          GAACACGGCCGACTCGTCCGTCTTCAACAACGCCATAGCCATGCTCCTGGGCAACGACATGAACACGGAGTGCAAGCCTGTTTTCGGCGACGGTATCATGTTTGATACTT
hpt10          GAACACGGCCGACTCGTCCGTCTTCAACAACGCCATAGCCATGCTCCTGGGCAACGACATGAACACGGAGTGCAAGCCTGTTTTCGGCGACGGTATCATGTTTGATACTT
hpt14          GAACACGGCCGACTCGTCCGTCTTCAACAACGCCATAGCCATGCTCCTGGGCAACGACATGAACACGGAGTGCAAGCCTGTTTTCGGCGACGGTATCATGTTTGATACTT
hpt11          GAACACGGCCGACTCGTCCGTCTTCAACAACGCCATAGCCATGCTCCTGGGCAACGACATGAACACGGAGTGCAAGCCTGTTTTCGGCGACGGTATCATGTTTGATACTT
hpt12          GAACACGGCCGACTCGTCCGTCTTCAACAACGCCATAGCCATGCTCCTGGGCAACGACATGAACACGGAGTGCAAGCCTGTTTTCGGCGACGGTATCATGTTTGATACTT
hpt13          GAACACGGCCGACTCGTCCGTCTTCAACAACGCCATAGCCATGCTCCTGGGCAACGACATGAACACGGAGTGCAAGCCTGTTTTCGGCGACGGTATCATGTTTGATACTT
hpt15          GAACACGGCCGACTCGTCCGTCTTCAACAACGCCATAGCCATGCTCCTGGGCAACGACATGAACACGGAGTGCAAGCCTGTTTTCGGCGACGGTATCATGTTTGATACTT
hpt16          GAACACGGCCGACTCGTCCGTCTTCAACAACGCCATAGCCATGCTCCTGGGCAACGACATGAACACGGAGTGCAAGCCTGTTTTCGGCGACGGTATCATGTTTGATACTT
hpt17          GAACACGGCCGACTCGTCCGTCTTCAACAACGCCATAGCCATGCTCCTGGGCAACGACATGAACACGGAGTGCAAGCCTGTTTTCGGCGACGGTATCATGTTTGATACTT
hpt18          GAACACGGCCGACTCGTCCGTCTTCAACAACGCCATAGCCATGCTCCTGGGCAACGACATGAACACGGAGTGCAAGCCTGTTTTCGGCGACGGTATCATGTTTGATACTT
```

```
                       2620      2630      2640      2650      2660      2670      2680      2690      2700      2710      2720
              ....|....|....|....|....|....|....|....|....|....|....|....|....|....|....|....|....|....|....|....|....|....|
HvGAMYB mRNA  cggtgtggagcaacttgcctcatgcttgtcaaatgtcggaggaattcaaatgagttcctcaccgaacctccagcggagtcgaaggagattcttggtatccctcaccctga
HvGAMYB_CDS   CGGTGTGGAGCAACTTGCCTCATGCTTGTCAAATGTCGGAGGAATTCAAATGA
hpt01         CGGTGTGGAGCAACTTGCCTCATGCTTGTCAAATGTC
hpt02         CGGTGTGGAGCAACTTGCCTCATGCTTGTCAAATGTC
hpt03         CGGTGTGGAGCAACTTGCCTCATGCTTGTCAAATGTC
hpt04         CGGTGTGGAGCAACTTGCCTCATGCTTGTCAAATGTC
hpt05         CGGTGTGGAGCAACTTGCCTCATGCTTGTCAAATGTC
hpt06         CGGTGTGGAGCAACTTGCCTCATGCTTGTCAAATGTC
hpt07         CGGTGTGGAGCAACTTGCCTCATGCTTGTCAAATGTC
hpt08         CGGTGTGGAGCAACTTGCCTCATGCTTGTCAAATGTC
hpt09         CGGTGTGGAGCAACTTGCCTCATGCTTGTCAAATGTC
hpt10         CGGTGTGGAGCAACTTGCCTCATGCTTGTCAAATGTC
hpt14         CGGTGTGGAGCAACTTGCCTCATGCTTGTCAAATGTC
hpt11         CGGTGTGGAGCAACTTGCCTCATGCTTGTCAAATGTC
hpt12         CGGTGTGGAGCAACTTGCCTCATGCTTGTCAAATGTC
hpt13         CGGTGTGGAGCAACTTGCCTCATGCTTGTCAAATGTC
hpt15         CGGTGTGGAGCAACTTGCCTCATGCTTGTCAAATGTC
hpt16         CGGTGTGGAGCAACTTGCCTCATGCTTGTCAAATGTC
hpt17         CGGTGTGGAGCAACTTGCCTCATGCTTGTCAAATGTC
hpt18         CGGTGTGGAGCAACTTGCCTCATGCTTGTCAAATGTC


                       2730      2740      2750      2760      2770      2780      2790      2800      2810      2820      2830
              ....|....|....|....|....|....|....|....|....|....|....|....|....|....|....|....|....|....|....|....|....|....|
HvGAMYB mRNA  ttgttttgaggaaaaattcagagaaagcctcatcgattgtatacgctgctgcgtgcgtacgcggctgctaccagatgcgctctccgttcaactaagccccttttccccaa
HvGAMYB_CDS
hpt01
hpt02
hpt03
hpt04
hpt05
hpt06
hpt07
hpt08
hpt09
hpt10
hpt14
hpt11
hpt12
hpt13
hpt15
hpt16
hpt17
hpt18
```

```
                  2840      2850      2860      2870      2880      2890      2900      2910      2920      2930      2940
              ....|....|....|....|....|....|....|....|....|....|....|....|....|....|....|....|....|....|....|....|....|....|
HvGAMYB mRNA  taaagttggcggagataagccggttatctattttttgtttgtttgtttcgaactagagaacccttttttgtcatctctgtggcatttatttgaacaatgtaagatcagtt
HvGAMYB_CDS
hpt01
hpt02
hpt03
hpt04
hpt05
hpt06
hpt07
hpt08
hpt09
hpt10
hpt14
hpt11
hpt12
hpt13
hpt15
hpt16
hpt17
hpt18

                  2950      2960      2970      2980      2990
              ....|....|....|....|....|....|....|....|....|....|
HvGAMYB mRNA  actgcttaaaaaaaaaaaaaaaaaaaaaaaaaaaaaaaaaaaaaaaaaaa
HvGAMYB_CDS
hpt01
hpt02
hpt03
hpt04
hpt05
hpt06
hpt07
hpt08
hpt09
hpt10
hpt14
hpt11
hpt12
hpt13
hpt15
hpt16
hpt17
hpt18
```
