# Supplementary material for: DNA polymorphisms and haplotype patterns of transcription factors involved in barley endosperm development are associated with key agronomic traits
Source: BMC Plant Biol. 2010 Jan 8;10:5. doi: 10.1186/1471-2229-10-5 (PMC2822787; doi:10.1186/1471-2229-10-5)
Supplement: Additional file 4 — Linkage disequilibrium in the surrounding region of the candidate genes BLZ1 (A), BLZ2 (B), BPBF (C), and HvGAMYB (D). The position 0.0 cM refers to the candidate gene. The symbols × and ◆ indicate significant (P = 0.05) and non significant pairwise comparisons, respectively. [file 1471-2229-10-5-S4.PDF]

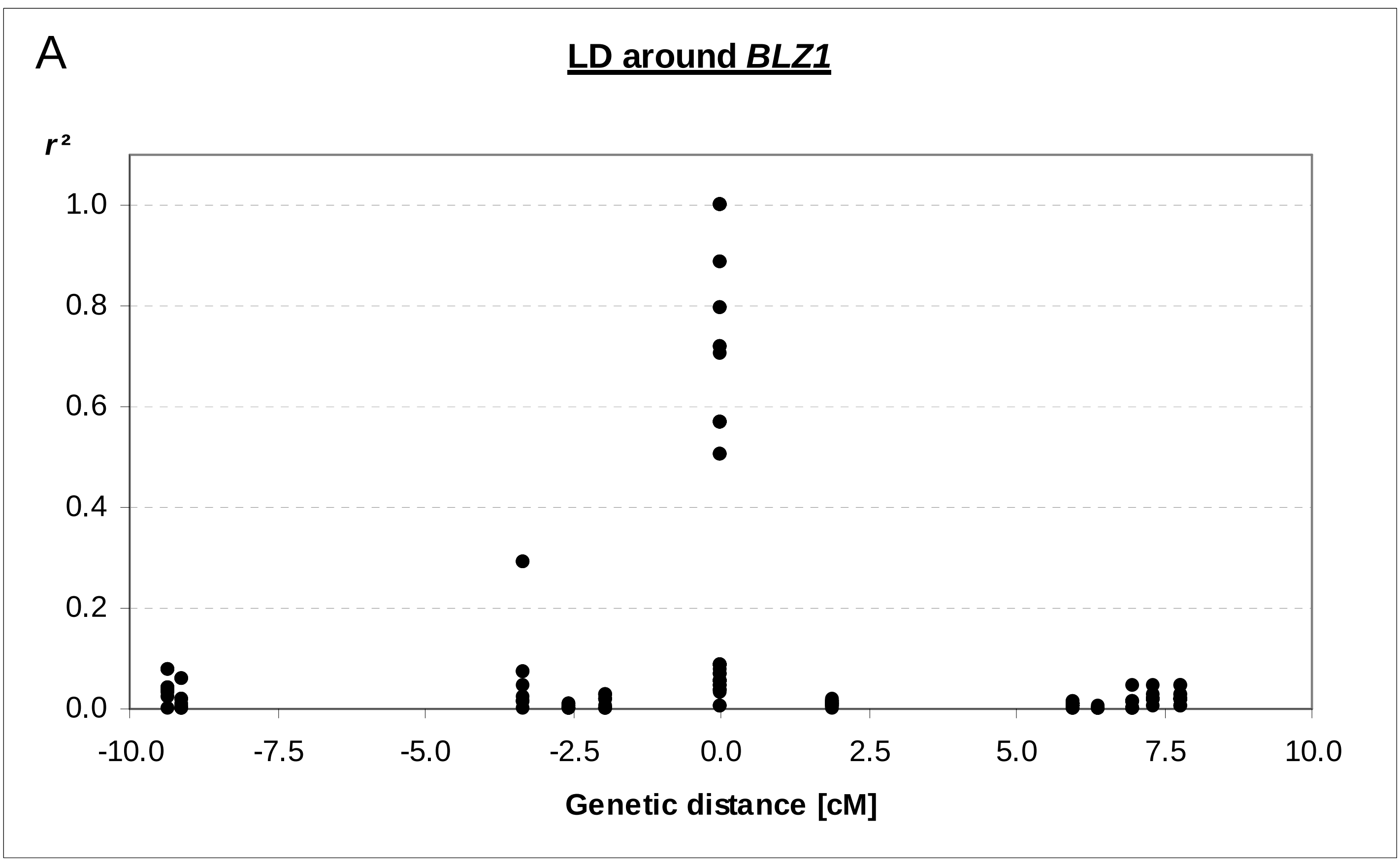

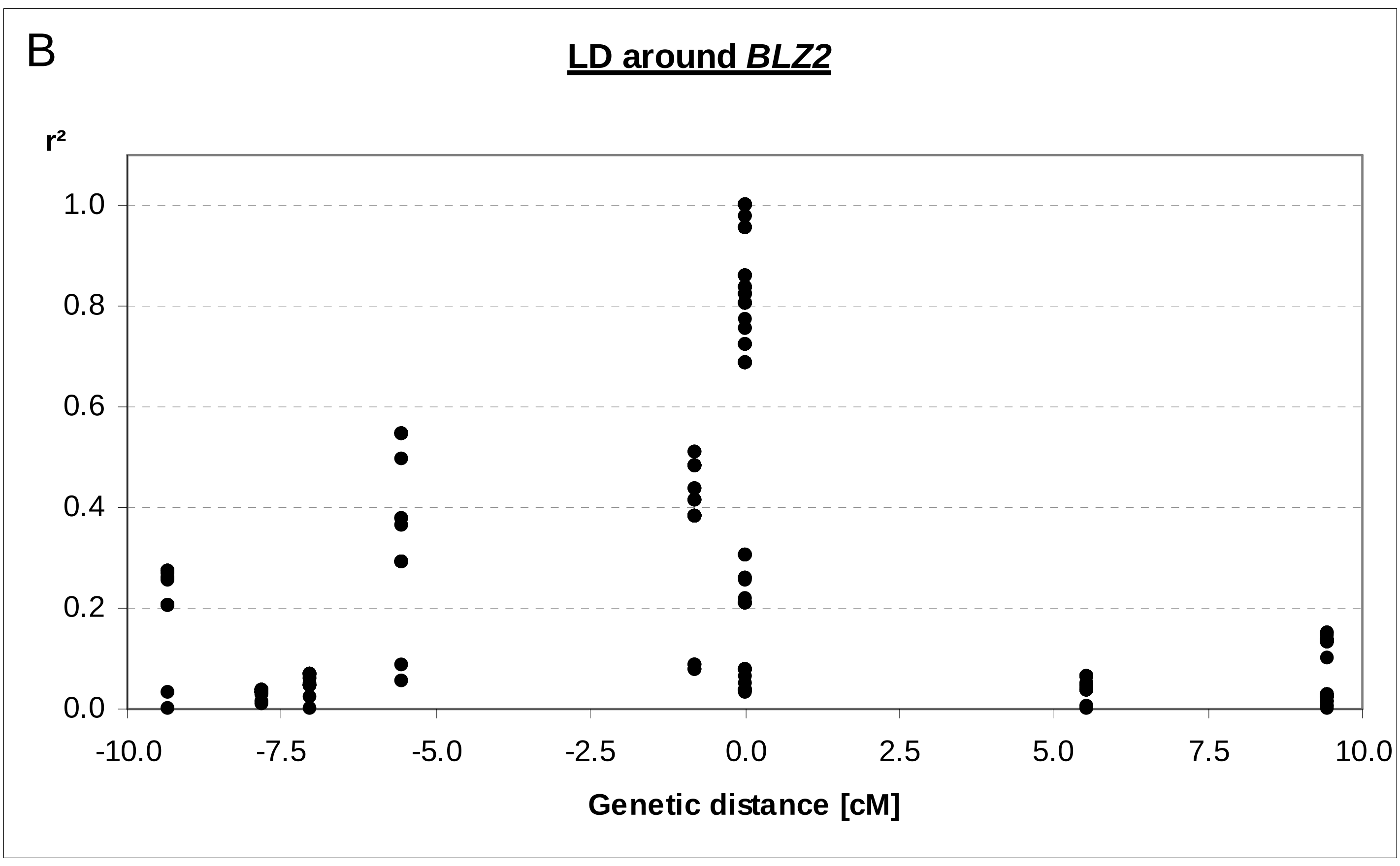

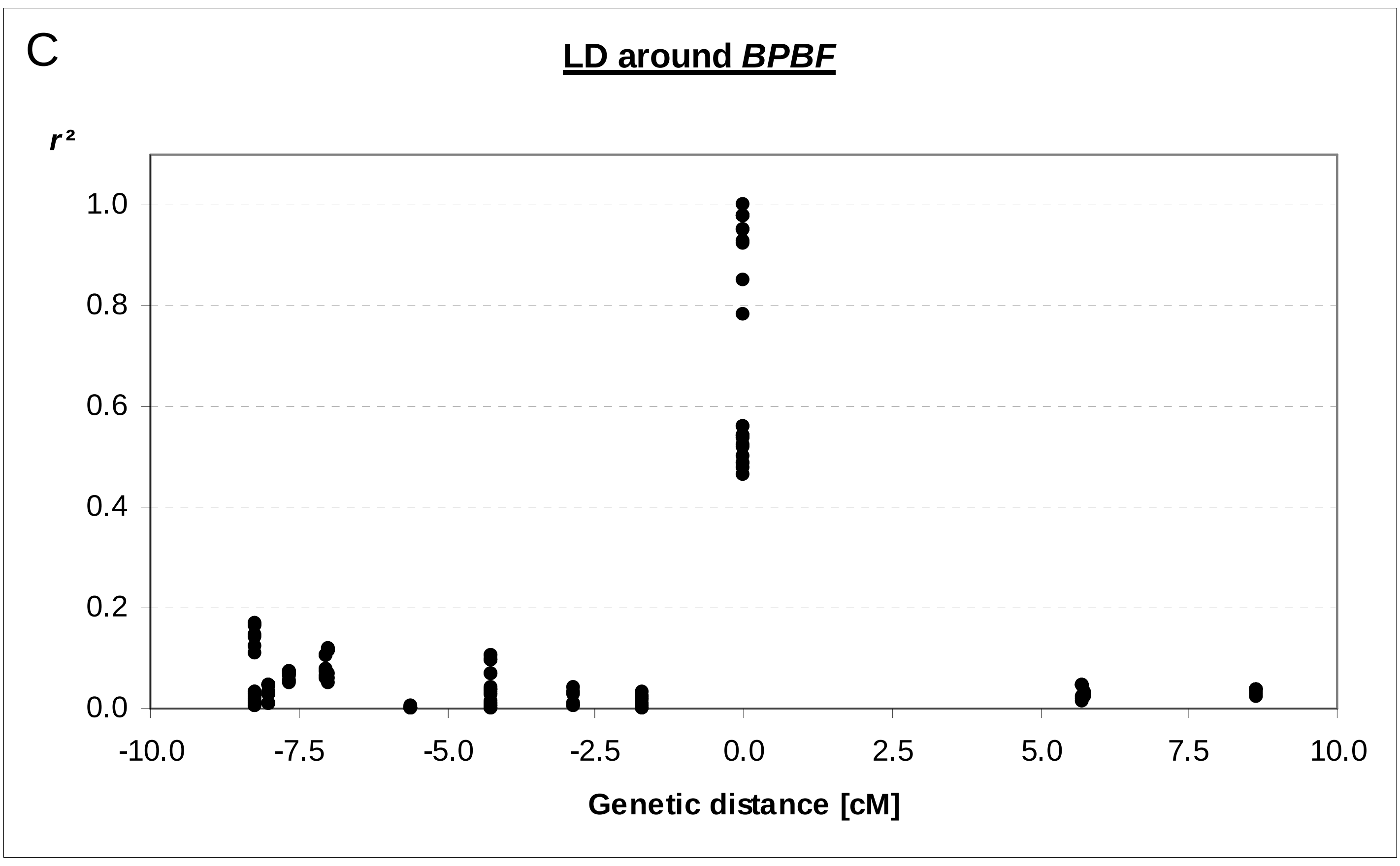

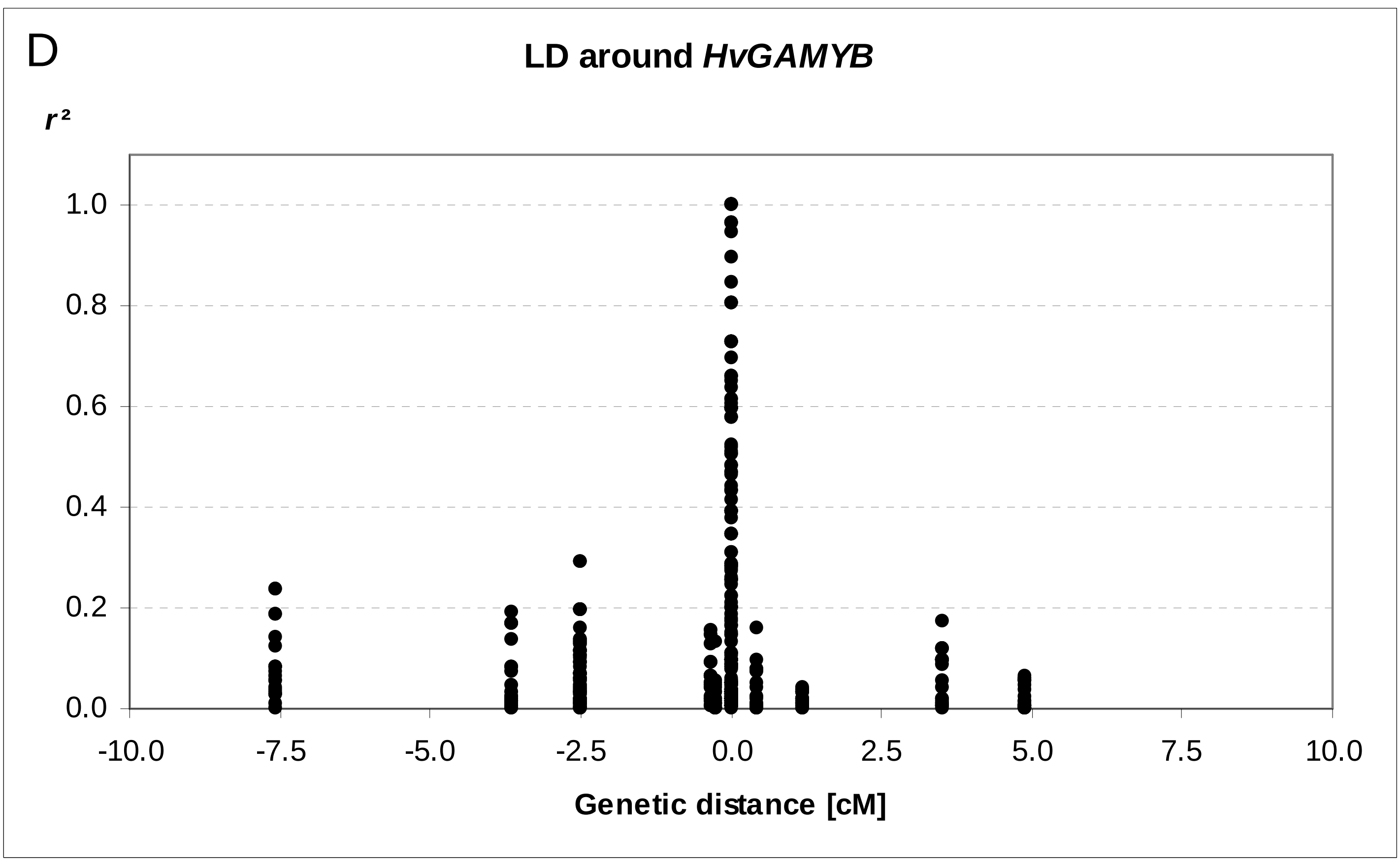

Additional file 5
Linkage disequilibrium in the surrounding region of the candidate genes *BLZ1* (A), *BLZ2* (B), *BPBF* (C), and *HvGAMYB* (D). The position 0.0 cM refers to the candidate gene.
